# Role of adiponectin and its receptors AdipoR1/2 in inflammatory bowel disease

**DOI:** 10.1186/s12964-025-02359-w

**Published:** 2025-07-26

**Authors:** Qiuyan Zhu, Xiaoli Jia, Shupeng Li, Jinxing Feng

**Affiliations:** 1 State Key Laboratory of Chemical Oncogenomics, School of Chemical Biology & Biotechnology, Shenzhen Graduate School, Peking University, Shenzhen, 518055 People’s Republic of China; 2 Department of Psychiatry, University of Toronto, Toronto, ON Canada; 3 Department of Neonatology, Shenzhen Children’s Hospital, Shenzhen, 518055 People’s Republic of China

**Keywords:** Adiponectin, AdipoR1, AdipoR2, Inflammatory bowel disease, Colon, Crohn’s disease, Ulcerative colitis

## Abstract

Intake of nutrients and water from diet to maintain life, a typical physiological function of gut, is highly dependent on the extensive immune network, whose imbalance is easy to induce inflammatory bowel disease (IBD) including Crohn’s disease (CD) and ulcerative colitis (UC). Clinical strategies to completely cure IBD are poor, so it is urgent to develop novel drugs or targets. Adiponectin (APN), an adipokine from adipocytes, regulates energy metabolism and immune response. High levels APN are inversely associated with CD severity UC colonic fibrosis. However, the mechanism by which APN interferes with IBD remains unclear. This review aims to analyze correlation and molecular mechanism between APN and IBD. APN and AdipoR2 proteins are highly expressed in colon which is a primary organ of IBD, and the target intersection of APN and IBD is huge. APN may interfere with lipid metabolism in IBD individuals through AdipoR1/2, but regulates neural and peripheral immune by AdipoR1 but not AdipoR2 and mediates nutritional and energy homeostasis through AdipoR2 rather than AdipoR1. Besides, APN mediates CRP and IL-6 through AdipoR1/2, AMPK and TNF-α through AdipoR1 and PI3K-Akt, PPARA and PPARG through AdipoR2 to affect IBD progression, which depends on direct interaction between APPL1 and AdipoR1/2. Unexpectedly, AMPK and TNF-α may also interact directly with AdipoR1. APN regulates CD through AdipoR1/2-metabolism process and UC through AdipoR1-inflammation axis or AdipoR2-fibrosis process. APN analogues or AdipoRon which is a dual agonist of AdipoR1/2 potentially reduces colonic fibrosis in UC and fistulae in CD, promotes mucosal healing, repairs intestinal microbiota homeostasis and increases autophagy to alleviate IBD symptoms by weakening TNF-α, IL-6, NLRP3, TGFB1 activities and aggrandizing P-AKT, PPARA, PPARG, INS, IRS1/2, IGF-1, TIMP1, NOD2, SIRT1 levels.

**Graphic Abstract:**

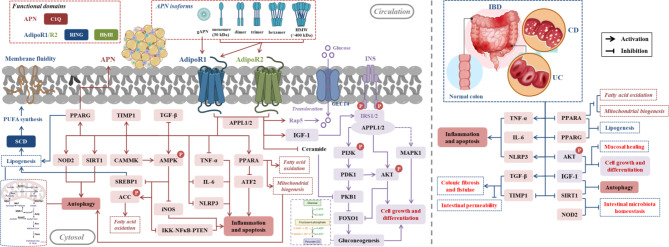

**Supplementary Information:**

The online version contains supplementary material available at 10.1186/s12964-025-02359-w.

## Introduction

Intake of nutrients and water from diet to maintain life is a typical physiological function of gut, which is highly dependent on the extensive network of immune cells [1]. Immune imbalance predisposes to intestinal diseases such as inflammatory bowel disease (IBD) which are characterized by dysregulated monocytes and macrophages [2, 3]. IBD, a chronic, idiopathic and relapsing inflammatory disease of gastrointestinal tract divided into Crohn’s disease (CD) and ulcerative colitis (UC) basing on severe forms with highly heterogeneous conditions [4, 5], affects not only gastrointestinal tract but also a variety of other organs, which is classified as extraintestinal manifestations (EIMs) that are common in either UC or CD [6]. Depression and anxiety are common clinical comorbidities of IBD, with an incidence of 21 ~ 26% in patients with IBD [5, 7] and recognition memory or anxiety-like behavior are also impaired in mice with intestinal inflammation [5]. Importantly, IBD is a truly global disease whose incidence is rising dramatically, which brings a significant burden on health care [8–11]. Periodically inhibiting tumor necrosis factor (TNF), interleukin-12 (IL-12), IL-23 and Janus kinase (JAK) are currently usual strategies for IBD, but these options are far from the clinical goal of curing IBD for its high recurrence rate [2, 12]. Therefore, it is urgent to develop novel IBD drugs or targets.

Adiponectin (APN), an adipokine from adipocytes and an independent risk factor for metabolic syndrome, type 2 diabetes and cardiovascular disease [13, 14] modulates energy metabolism by glucose homeostasis, insulin sensitivity and fatty acid catabolism, and immune response by activating adenosine 5’-monophosphate (AMP)-activated protein kinase (AMPK)/ p70S6 kinase (S6), signal transducer and activator of transcription 3 (STAT3)/ vascular endothelial growth factor (VEGF), nuclear factor-kB (NF-kB), IL-1, IL-6, IL-8, IL-12, TNF-α and C-reactive protein (CRP) through AdipoR1/2 [4,13–15]. Role of APN varies among different IBD subtypes. High levels APN secreted by hypertrophic mesenteric adipocytes in CD patients are inversely associated with disease severity [16] and elevated APN levels in colon of UC patients is positively correlated with colonic fibrosis [17], suggesting that APN shows potential colitis inhibitory and promoted colonic fibrosis effect. Chronic inflammation associated with visceral obesity inhibits APN production and perpetuates inflammation, verifying anti-inflammatory effect of APN and its negative correlation with markers of inflammation. Inversely, APN increases rather than decreases in typical chronic inflammatory or autoimmune diseases such as IBD that have no connection with the increase of adipose tissue, indicating the pro-inflammatory effect of APN and its positive correlation with markers of inflammation [14]. In CD, APN possibly exerts an anti-inflammatory effect by promoting macrophage metabolism of hypertrophied adipose tissue [18–20], while APN possesses a pro-inflammatory effect in UC through various inflammatory signaling pathways for UC may be unrelated to the increase of adipose tissue. APN-AdipoR1 axis aggrandizes the expression of pro-inflammatory factors and neutrophil chemokines, and then recruits neutrophils into colon to exacerbating the deterioration of IBD [4], which is also a common IBD phenotype in mice with short-term AdipoR1 deficiency [21] and AdipoR1 overexpression [4]. Factors targeting ADN-AdipoR1 function may be serviceable candidates for improving IBD, resulting from the dual effect of APN-AdipoR1 axis in IBD. APN deficiency modulates CXCL13 signal transmission of macrophages in colon by attenuating p-AKT, p-P38, p-ERK, p-JNK signaling pathway, and induces colonic fibrosis by increasing a-SMA and COL1Al expression in fibroblasts of mice and human [17].

APN interferes with IBD progression by inflammation [16, 18, 22, 23] but whether APN plays a pro-inflammatory [16, 23] or anti-inflammatory [18, 22] role in IBD is still debated for different models of IBD colitis [24]. What’s more, the anti-inflammatory or pro-inflammatory effects of APN on IBD through which receptor are unclear. Therefore, this review aims to explore how APN disturbs IBD progression by its receptors (AdipoR1 or AdipoR2) and inflammation basing on specific signaling pathways and physiological functions.

## Spatial structure and functional domain of APN and its receptor AdipoR1/2

**Fig. 1:**
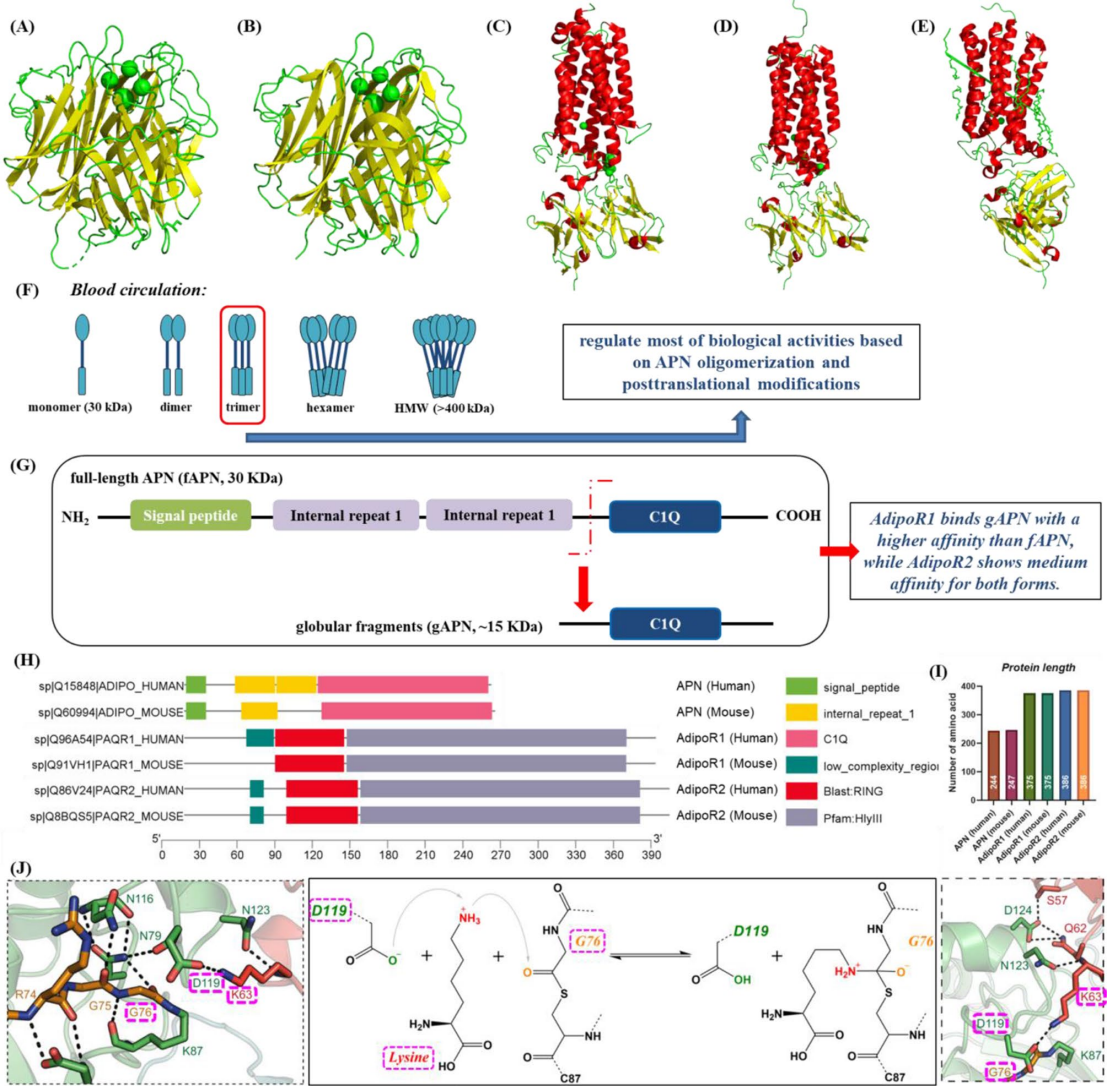
Protein domains and spatial structure of adiponectin (APN), AdipoR1 and AdipoR2. (**A**-**E**) crystal structure of a single-chain trimer of human APN globular domain (PDB: 4DOU), trimeric globular domain of APN (PDB: 6U66), revised crystal structure of the human AdipoR1 in an open conformation (PDB: 5LXG), AdipoR1 (PDB ID: 6KS0), human AdipoR1 (PDB ID: 6KS1); (**F**) presence of APN in the blood circulation; (**G**) full-length APN break into globular fragments (~ 15 KDa) at amino acid 110 site; (**H**) protein domains; (**I**) protein length; (**J**) chemical scheme to activate the acceptor lysine by ubiquitination [35]. (**A**-**E**) red: secondary structure helix (ss h), yellow: secondary structure beta sheet (ss s), green: secondary structure loop and other structures (ss l+). All human and mouse proteins domains are derived from UniProtKB database [Available (November 2024): https://www.uniprot.org/uniprotkb/]. The TBtools-II software was used for data visualization [78]. APN: adiponectin; AdipoR1: adiponectin receptor 1; AdipoR2: adiponectin receptor 2; HMW: high molecular weight

*APN with low and middle molecular weight strongly inhibits proliferative activity of colonic epithelial cells*,* respectively modulating AMPK and PPAR-α pathway by AdipoR1 and AdipoR2.* The spatial structure of APN is mainly beta sheet (Fig. 1A-B), while AdipoR1 (Fig. 1C-D) and AdipoR2 (Fig. 1E) is dominated by helix. APN is present in blood circulation as dimers, trimers or hexamer protein complex with high molecular weight (> 400 kDa), where oligomers including monomers (30 KDa), dimers and trimers (30–180 KDa, low/ middle molecular weight) regulate most of biological activities based on APN oligomerization and posttranslational modifications (Fig. 1F) [23, 25–27]. The complete structure is most common form of APN in plasma, with only a few globular fragments (~ 15 KDa) produced by proteolytic cleavage of full-length APN (fAPN, 30 KDa) at amino acid 110 site (Fig. 1G) [27]. APN circulates at physiological concentration 3 ~ 30 µg/mL accounting for ~ 0.05% of all plasma proteins [25, 28] while the level of fAPN in plasma exceeds 10 µg/mL constituting ~ 0.01% of total amount of plasma protein [27]. AdipoR1 and AdipoR2 are primary receptors of fAPN and globular APN (gAPN), mediating metabolic processes such as fatty acid oxidation, glucose uptake, energy expenditure in vivo through activation of AMPK and peroxisome proliferator-activated receptor (PPAR)-α [25, 27, 29]. The gAPN inhibited proliferative activities of colonic epithelial cells is stronger than that of fAPN for activating AMPK and inhibiting mTOR, which promotes colorectal carcinogenesis [22]. AdipoR1 binds gAPN with a higher affinity than fAPN, while AdipoR2 shows medium affinity for both forms (Fig. 1G) [30, 31]. The gAPN, a strong activator of AMPK, induces AMPK phosphorylation to inhibit acetyl-CoA carboxylase (ACC2) and increase fatty-acid oxidation, glucose uptake, lactate production [32, 33].

**Fig. 2:**
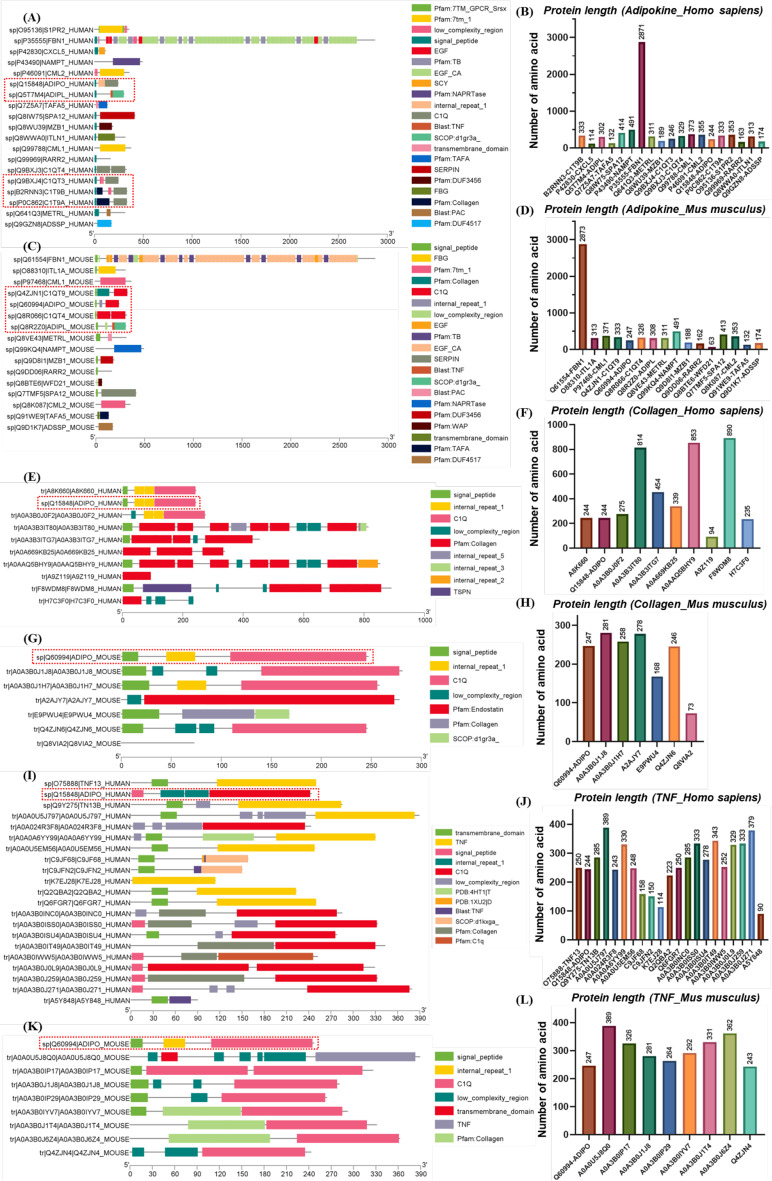
Structural similarity between adiponectin (APN), adipokine, collagen and tumor necrosis factor (TNF). (**A**, **C**, **E**, **G**, **I**, **K**) domains of adipokine, collagen and TNF family from Homo sapiens and Mus musculus; (**B**, **D**, **F**,** H**, **J**, **L**) protein length of adipokine, collagen and TNF family from Homo sapiens and Mus musculus. NAMPT: nicotinamide phosphoribosyltransferase; RARR2: retinoic acid receptor responder protein 2; METRL: Meteorin-like protein; C1QT: complement C1q/TNF-related protein; ADIPL: adipolin; ADIPO: adiponectin (30 kDa adipocyte complement-related protein); C1T: complement C1q and TNF-related protein; ITLN1: intelectin-1; FBN1: fibrillin-1; TAFA5: chemokine-like protein TAFA-5; CXCL5: C-X-C motif chemokine 5; SPA12: serpin A12; MZB1: marginal zone B- and B1-cell-specific protein; ADSSP: adipose-secreted signaling protein; CML: chemerin-like receptor; S1PR2: sphingosine 1-phosphate receptor 2; ITL1A: intelectin-1a; WFD21: protein Wfdc21 (Wdnm1-like protein); A9Z119: collagen type VII alpha 1; A8K660: adiponectin [cDNA FLJ78108, highly similar to Homo sapiens adiponectin, C1Q and collagen domain containing (ADIPOQ), mRNA]; A0A3B0J0F2: adiponectin D; Q15848-ADIPO: adiponectin (30 kDa adipocyte complement-related protein); A0A669KB25: collagen type XIII alpha 1 chain; F8WDM8: collagen type XXIV alpha 1 chain; A0A3B3ITG7: collagen type IV alpha 1 chain; H7C3F0: collagen type XVI alpha 1 chain; A0A3B3IT80: collagen type IV alpha 2 chain; A0AAQ5BHY9: collagen type IV alpha 2 chain; Q8VIA2: A1(XI) collagen; A2AJY7: collagen, type XV, alpha 1; E9PWU4: adiponectin, C1Q and collagen domain containing; Q60994-ADIPO: adiponectin (30 kDa adipocyte complement-related protein); A0A3B0J1H7: adiponectin d; Q4ZJN6: adiponectin m (C1qTNF3); A0A3B0J1J8: adiponectin p; Q9Y275-TN13B: TNF ligand superfamily member 13B; A0A0U5J797: TNF ligand 7 C; A0A0A6YY99: protein TNFSF12-TNFSF13; C9JFN2: TNF superfamily member 13; C9JF68: TNF superfamily member 13; O75888-TNF13: TNF ligand superfamily member 13; K7EJ28: TNF superfamily member 13; Q6FGR7: TNFSF13 protein; A0A0U5EM56: TNF ligand 7B; Q2QBA2: TNF superfamily member 13; A5Y848: TNF ligand superfamily member 13 epsilon; A0A3B0J0L9: adiponectin N; A0A3B0J271: adiponectin P (C1q and TNF related protein 1, isoform CRA_a); A0A3B0ISU4: adiponectin Q; A0A3B0IWW5: adiponectin O (C1q and TNF related protein 8); A0A3B0INC0: adiponectin H; A0A3B0IT49: adiponectin G; A0A024R3F8: adiponectin E; A0A3B0ISS0: adiponectin F2; A0A3B0J259: adiponectin F1; A0A3B0IP17: adiponectin n; A0A3B0J1J8: adiponectin p; A0A3B0IP29: adiponectin q; Q4ZJN4: adiponectin e (C1qTNF5); A0A3B0J6Z4: adiponectin f1; A0A3B0IYV7: adiponectin h; A0A3B0J1T4: adiponectin g; A0A0U5J8Q0: TNF ligand 7c. All human and mouse proteins domains are derived from UniProtKB database [Available (November 2024): https://www.uniprot.org/uniprotkb/]. The TBtools-II software was used for data visualization [78]

*Physiological function of APN is similar to collagen and TNF family for its C1Q and signal peptide domain.* The total length of APN, AdipoR1 and AdipoR2 proteins in human and mouse were 244, 247, 375, 375, 386 and 386 amino acids, respectively (Fig. 1H-I). Human APN protein possesses an N-terminal signal peptide, two internal repeat 1 and a C1Q domain, while that of mouse is short of one internal repeat 1 domain (Fig. 1H). C1Q domain of APN is a collagen-like domain, while internal repeat 1 domains of APN are hypervariable and gAPN formation originates from C-terminal globular domain [24]. The globular region of APN extraordinarily resembles TNF-α [24–26] and its C1Q domain is also a characteristic of collagen family modulating innate humoral immune system [18, 24, 25]. Adjacent branch in phylogenetic tree of adipokine family shows that structure and function of human APN (Q15848, 244 amino acids) is similar to adipolin (ADIPL, 302 amino acids), complement C1q and TNF-related protein 9 A (C1T9A, 333 amino acids), C1T9B (333 amino acids) and complement C1q/TNF-related protein 3 (C1QT3, 246 amino acids) (Fig. 2A-B and Figure S1A), while mouse APN (Q60994, 247 amino acids) approaches to ADIPL (308 amino acids), C1QT4 (326 amino acids) and C1QT9 (333 amino acids) (Fig. 2C-D and Figure S1B). All Homo sapiens APN, C1T9A, C1T9B, C1QT3 and Mus musculus APN, C1QT4, C1QT9 contain a C1Q domain (Fig. 2A and C). Human APN (Q15848, 244 amino acids) approximates to collagen type IV/ VII/ XIII/ XVI/ XXIV alpha 1 (454, 94, 339, 235, 890 amino acids) and collagen type IV alpha 2 chain (A0A3B3IT80: 814 amino acids, A0AAQ5BHY9: 853 amino acids) (Fig. 2E-F and Figure S2), while mouse APN (Q60994, 247 amino acids) is close to A1 (XI) collagen (73 amino acids) and collagen type XV alpha 1 (278 amino acids) (Fig. 2G-H and Figure S3). Common domain of Homo sapiens APN (Q15848), collagen type IV/ XXIV alpha 1 (A0A3B3ITG7, F8WDM8), collagen type IV alpha 2 (A0A3B3IT80, A0AAQ5BHY9) is a signal peptide domain (Fig. 2E). Meanwhile, human APN (Q15848, 244 amino acids) comes near TNF superfamily member 13 (C9JFN2, C9JF68, K7EJ28, Q6FGR7, Q2QBA2; 150, 158, 114, 250, 223 amino acids), TNF ligand superfamily member 13/ 13B/ epsilon (Q9Y275, O75888, A5Y848; 285, 250, 90 amino acids), TNF ligand 7B/ 7 C (A0A0U5EM56, A0A0U5J797; 248, 389 amino acids) (Fig. 2I-J and Figure S4), while mouse APN (Q60994, 247 amino acids) draws near TNF ligand 7c (A0A0U5J8Q0, 389 amino acids) (Fig. 2K-L and Figure S5). APN homologs Homo sapiens adiponectin E/ F1/ F2/ G/ H/ N/ O/ P/ Q/ (A0A024R3F8, A0A3B0J259, A0A3B0ISS0, A0A3B0IT49, A0A3B0INC0, A0A3B0J0L9, A0A3B0IWW5, A0A3B0J271, A0A3B0ISU4) and Mus musculus adiponectin e/ f1/ g/ h/ n/ p/ q (Q4ZJN4, A0A3B0J6Z4, A0A3B0J1T4, A0A3B0IYV7, A0A3B0IP17, A0A3B0J1J8, A0A3B0IP29) are members of TNF family (Fig. 2I-L), and adiponectin D (human: A0A3B0J0F2, mouse: A0A3B0J1H7), adiponectin containing C1Q, collagen domain (E9PWU4), adiponectin m (C1qTNF3, Q4ZJN6) and adiponectin p (A0A3B0J1J8) also belongs to collagen family (Fig. 2E-H).

*AdipoR1 and AdipoR2*,* one of transmembrane proteins containing seven transmembrane domains*,* promote lysine ubiquitination for their RING domain.* Human AdipoR1 has one low complexity region more than mouse AdipoR1, while types, amounts and location of domain in human and mouse AdipoR2 are identical. C-terminal (3’) of AdipoR1 or AdipoR2 is located outside the membrane and their amino terminal (N-terminal, 5’) inside, which attaches them to transmembrane (TM) properties and each possesses seven TM domains [24, 25]. Structure similarities of AdipoR1 and AdipoR2 are Blast: RING and Pfam: HlyIII domains (Fig. 1H), where their seven TM domains are located in HlyIII (Figure S7 and Figure S8). RING (really interesting new gene) domain mediates ubiquitination which successively activates ubiquitin-activating (E1), ubiquitin-conjugating (E2) and ubiquitin-protein ligase enzymes (E3s) [34]. E3s containing RING domain directly promote ubiquitination of substrate lysine by E2 (Fig. 1J) [34–36] and most ubiquitination sites of AdipoR1/2 are located at lysine in RING domains (Figure 1H, Figure S7 and Figure S8; human or mouse AdipoR1 (site 72–127): K79, K86, K126; human AdipoR2 (site 81–138): K90, K97, K116, K137; mouse AdipoR2 (site 81–138): K97, K116, K137). The N-terminal dehydrogenation of lysine promotes hydrolysis of ester group at asparticacid 119 (D119) residues in ubiquitin (Ub) and covalently binds to carbonyl group of glycine 76 (G76) residues in Ub, which is a small, highly conserved, cytoplasmic protein composed of 76 amino acid residues [34–36]. The structure of AdipoR1/2 and membrane-associated RING-cysteine-histidine (MARCH) family are highly similar, with a N-terminal RING domain followed by multiple C-terminal TM domains [36]. In eukaryotes, HlyIII family, also name progestin and adipoQ receptor (PAQR) belonging to the CREST superfamily, consists of seven TM molecules that encode AdipoR1/2 and other functional receptors with a broad spectrum of apparent ligand specificities.

**Fig. 3:**
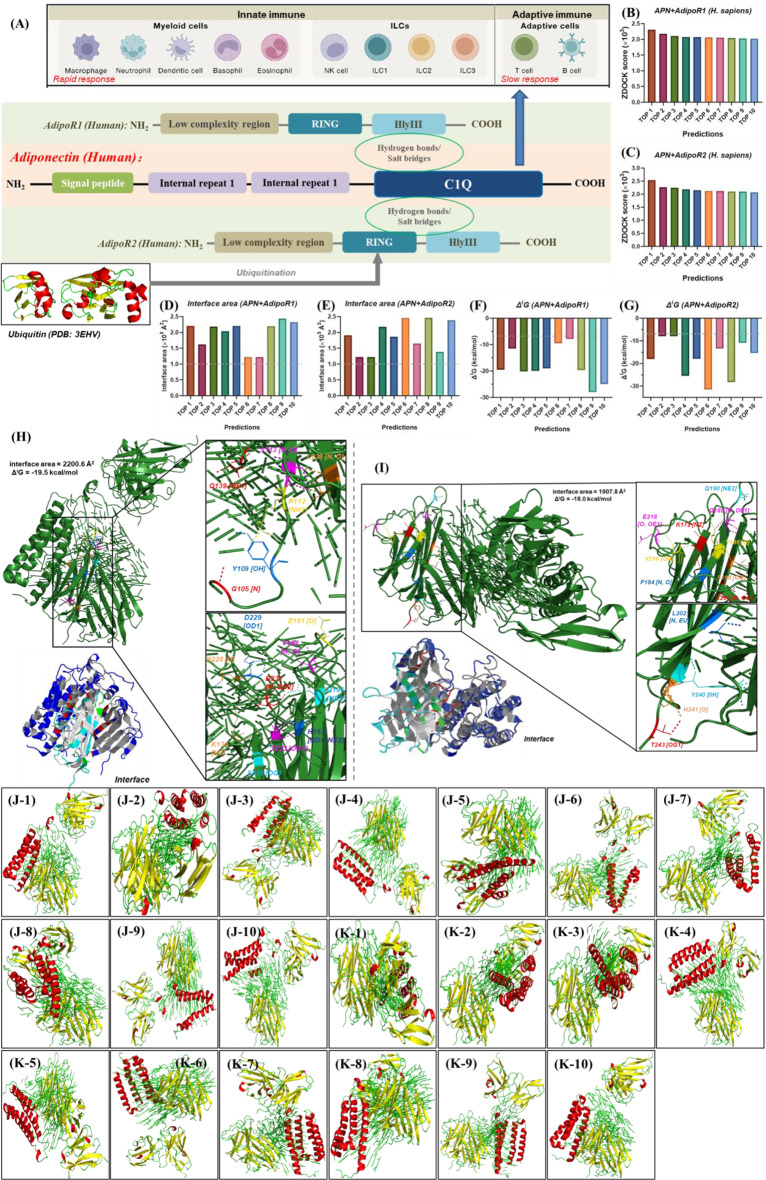
Interaction of adiponectin (APN) and AdipoR1/2. (**A**) diagram between domain and function of APN, AdipoR1 and AdipoR2; (**B**) ZDOCK score of APN and AdipoR1 by molecular docking; (**C**) ZDOCK score of APN and AdipoR2 by molecular docking; (**D**) interface area between APN and AdipoR1 in TOP 1–10 complex by molecular docking; (**E**) interface area between APN and AdipoR2 in TOP 1–10 complex by molecular docking; (**F**) Gibbs free energy (Δ^i^G) of APN + AdipoR1 complex (TOP1-10) by molecular docking; (**G**) Δ^i^G of APN + AdipoR2 complex (TOP1-10) by molecular docking; (**H**-**I**) interactional sites between APN and AdipoR1 or AdipoR2 in TOP 1 complex by molecular docking; (J1-10) top 10 APN + AdipoR1 complex with highest ZDCOK score; (K1-10) top 10 APN + AdipoR2 complex with highest ZDCOK score. The diagrams of immune cells (**I**) are taken from previous study [79]. Interface area > 1000 Å [2] and Δ^i^G<-7.0 kcal/mol are defined as a stable structure. ZDOCK is used for molecular docking (**B**-**C**, **H**-**I**, J1-10, K1-10) and PDBePISA is applied to analyze docking results (**D**-**G**). (J1-10, K1-10) red: secondary structure helix (ss h), yellow: secondary structure beta sheet (ss s), green: secondary structure loop and other structures (ss l+). The PDB ID of APN, AdipoR1 and AdipoR2 used for molecular docking are 6U66, 5LXG and 6KS1. APN: adiponectin; AdipoR1: adiponectin receptor 1; AdipoR2: adiponectin receptor 2. Available (November 2024): https://zdock.wenglab.org/; https://www.ebi.ac.uk/msd-srv/prot_int/

*The C1Q domain endows APN not only with immune function*,* but also binding activities with AdipoR1 or AdipoR2.* The binding mode between APN and its receptor AdipoR1/2 has not been reported previous, so molecule docking is used to explore the binding sites of APN to AdipoR1/2 (Fig. 3, Figure S9 and Figure S10). The structures of top 10 complex of APN + AdipoR1 (Fig. 3J1-10) and APN + AdipoR2 (Fig. 3K1-10) with highest ZDOCK scores (Fig. 3B-C) are stable for their interface area > 1000 Å [2] (Fig. 3D-E) and Gibbs free energy (Δ^i^G) <-7.0 kcal/mol (Fig. 3F-G) [37, 38]. APN binds with AdipoR1 at site G105-L238 (Fig. 3H) and AdipoR2 at site K172-T243 (Fig. 3I), where its C1Q domain is located at P106-Y242 (Fig. 1H). Meanwhile, binging sites of APN + AdipoR1 and APN + AdipoR2 are I139-N244 in AdipoR1 (Figure S9) and W103-S238 in AdipoR2 (Figure S10). HlyIII domain of AdipoR1 is located in S129-V352, which RING domain and HlyIII domain of AdipoR2 is in P81-F138 and S140-V363 (Fig. 1H). Therefore, the C1Q domain endows APN not only with immune function, but also binding activities with AdipoR1 or AdipoR2 (Fig. 3A). Besides, APN and AdipoR1/2 are bonded by hydrogen bonds and salt bridges.

## APN, AdipoR1 and AdipoR2 in different organ tissues and cell types of human

*APN and AdipoR2 proteins are highly expressed in colon which is the primary lesion organ of IBD.* The Human Protein Atlas database is used to determine the distribution of APN and its receptor AdipoR1/2 in different organ tissues and cell types of human. Although APN mRNA expressed in colon is third only to that in adipose tissue and breast (Figure S11A), APN protein is highly expressed in colon (Figure S11B). To further explore the expression of APN in human intestinal tissue, Human Protein Atlas database is again surveyed for single-cell transcriptional analysis. APN is found to be present in distal enterocytes, intestinal goblet cells, enteroendocrine cells, undifferentiated cells and T-cells of colon (Figure S11C). AdipoR1 and AdipoR2 have poor tissue and cell specificity. Although the distribution of AdipoR1 protein has not been reported, its mRNA is widely expressed in various human tissues (Figure S12A-B). Moreover, AdipoR2 mRNA and protein are highly expressed in gastrointestinal tract (Figure S13A-C). AdipoR1 and AdipoR2 are mainly distributed in distal enterocytes, intestinal goblet cells, enteroendocrine cells, undifferentiated cells and T-cells of colon, which is similar to APN (Figure S12C and Figure S13D).

## APN, AdipoR1 and AdipoR2 in mouse colon

*Adult mouse colon is feasible for APN research models.* The expression of APN and its receptor AdipoR1/2 in colon of mice is investigated through Mouse Genome Informatics (MGI) database. Although APN and its receptor AdipoR1/2 are not detected during the embryonic period and post-natal day 0 (P0) ~ P3, they are expressed in colon of mice with age ≥ P4 (Figure S14-S16). Adult mouse colon is feasible as APN target organ. The proliferative activity of colon epithelial cells is enhanced in APN deletion and AdipoR1 deficient mice. AdipoR1 is principally expressed in colon in contrast to AdipoR2 and APN inhibits the proliferation of colon epithelial cells *via* the AdipoR1-mediated AMPK/mTOR pathway under high-fat diet [22]. APN deletion decreases AMPK phosphorylation of intestinal epithelial cells and increases intestinal polyp development in mice [28].

## Molecular dynamics of APN, AdipoR1 and AdipoR2 in IBD

**Fig. 4:**
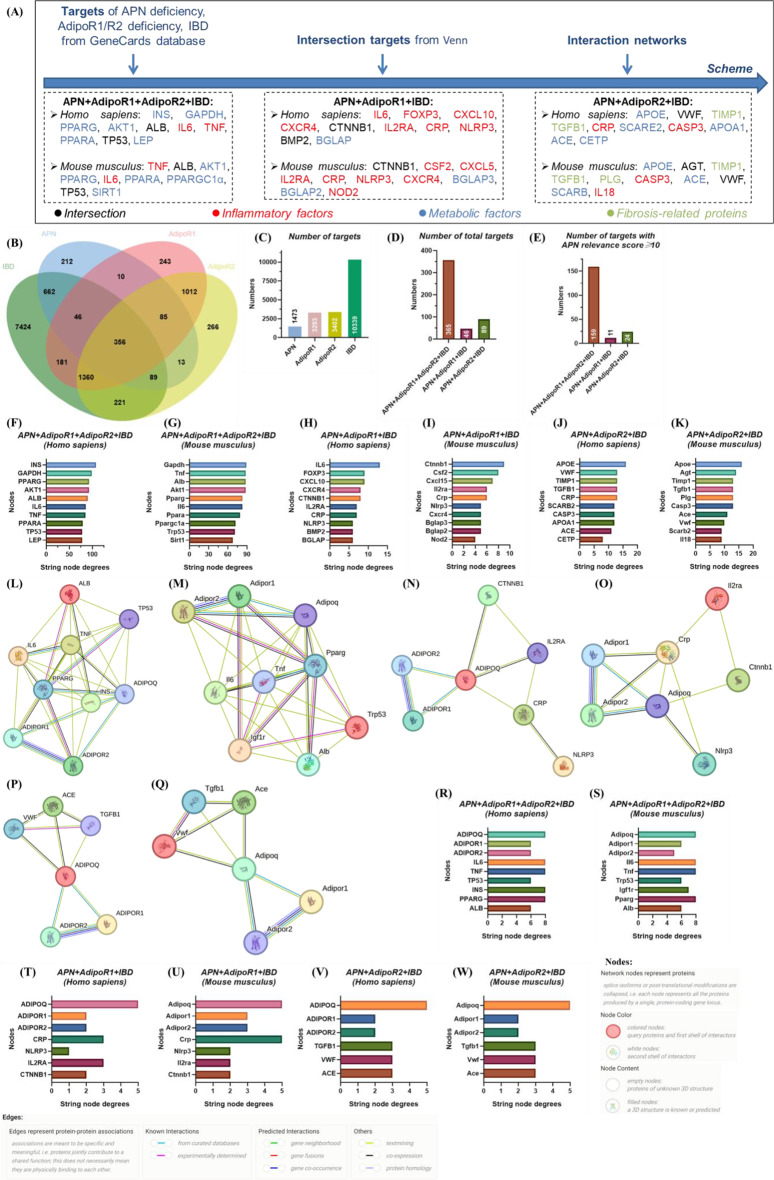
Venn and interaction network of adiponectin, AdipoR1, AdipoR2 and inflammatory bowel disease targets. (**A**) Target screening scheme; (**B**) Venn; (**C**) number of targets; (**D**) number of total targets in APN + AdipoR1 + AdipoR2 + IBD, APN + AdipoR1 + IBD and APN + AdipoR2 + IBD; (**E**) number of targets with APN relevance score ≥ 10 in APN + AdipoR1 + AdipoR2 + IBD, APN + AdipoR1 + IBD and APN + AdipoR2 + IBD; (**F**-**K**) top 10 targets with highest sting node degrees; (**L**, **N**, **P**) interaction network of human APN + AdipoR1 + AdipoR2 + IBD, APN + AdipoR1 + IBD and APN + AdipoR2 + IBD targets with APN relevance score ≥ 10, IBD relevance score ≥ 50 and top 10 highest sting node degrees; (**M**, **O**, **Q**) interaction network of mouse APN + AdipoR1 + AdipoR2 + IBD, APN + AdipoR1 + IBD and APN + AdipoR2 + IBD targets with APN relevance score ≥ 10, IBD relevance score ≥ 50 and top 10 highest sting node degrees; (R-W) sting node degrees of human and mouse APN + AdipoR1 + AdipoR2 + IBD, APN + AdipoR1 + IBD, APN + AdipoR2 + IBD targets with APN relevance score ≥ 10 and IBD relevance score ≥ 50. The targets of IBD, APN deficiency, AdipoR1 deficiency and AdipoR2 deficiency are acquired from GeneCards database. The interaction networks are analyzed by STRING database. APN: adiponectin; AdipoR1: adiponectin receptor 1; AdipoR2: adiponectin receptor 2; IBD: inflammatory bowel disease; INS: insulin; GAPDH: glyceraldehyde-3-phosphate dehydrogenase; PPARG: peroxisome proliferator activated receptor gamma; AKT1: AKT serine/threonine kinase 1; ALB: albumin; IL: interleukin; TNF: tumor necrosis factor; PPARA: peroxisome proliferator activated receptor alpha; TP53: tumor protein P53; LEP: leptin; PPARGC1α: PPARG coactivator 1 alpha; SIRT1: sirtuin 1; FOXP3: forkhead box protein P3; CXCL: C-X-C motif chemokine ligand; CXCR4: C-X-C motif chemokine receptor 4; CTNNB1: catenin beta 1; IL2RA: interleukin 2 receptor subunit alpha; CRP: C-reactive protein; NLRP3: NLR family pyrin domain containing 3; BMP2: bone morphogenetic protein 2; BGLAP: bone gamma-carboxyglutamate protein; CSF2: colony stimulating factor 2; NOD2: nucleotide binding oligomerization domain containing 2; APOE: apolipoprotein E; VWF: Von Willebrand factor; TIMP1: TIMP metallopeptidase inhibitor 1; TGFB1: transforming growth factor beta 1; SCARB: scavenger receptor class B member; CASP3: caspase 3; APOA1: apolipoprotein A1; ACE: angiotensin I converting enzyme; CETP: cholesteryl ester transfer protein; AGT: angiotensinogen; PLG: plasminogen. Available (November 2024): https://www.genecards.org/; https://cn.string-db.org/cgi/input?sessionId=bBSQf0gz7Y6u&input_page_show_search=on

The targets of IBD, APN deficiency, AdipoR1 deficiency and AdipoR2 deficiency are firstly screened from GeneCards database, and then intersection targets between them are determined through Venn diagrams (Fig. 4A). Subsequently, their interaction networks are analyzed by STRING database and functional enrichment is acquired through Gene ontology (GO), canonical pathways and Kyoto Encyclopedia of Genes and Genomes (KEGG) pathways. Finally, interaction sites are predicted by molecular docking.

### Target screening

*Target intersection of APN and IBD is enormous.* The targets of IBD, APN deficiency, AdipoR1 deficiency and AdipoR2 deficiency are acquired from GeneCards database, whose quantities after eliminating duplicate values are 10,339, 1473, 3293 and 3402 (Fig. 4B-C). The numbers of intersection targets of APN + AdipoR1 + AdipoR2 + IBD, APN + AdipoR1 + IBD and APN + AdipoR2 + IBD are 356, 46 and 89 (Fig. 4B-D). Excluding the intersection with AdipoR1 or AdipoR2, the amount of intersection targets between IBD and APN is 662 (Fig. 4B-C). Top 10 targets of APN + AdipoR1 + AdipoR2 + IBD (Table S1) are adiponectin, C1Q and collagen domain containing [ADIPOQ, relevance score (RS) = 149.4171448], acyl-CoA dehydrogenase very long chain (ACADVL, RS = 96.89267731), carnitine palmitoyltransferase 2 (CPT2, RS = 91.03253174), adenosine deaminase (ADA, RS = 77.02828217), solute carrier family 2 member 1 (SLC2A1, RS = 74.02565002), dihydrolipoamide dehydrogenase (DLD, RS = 73.75358582), methylenetetrahydrofolate reductase (MTHFR, RS = 67.14596558), carnitine palmitoyltransferase 1 A (CPT1A, RS = 63.20038605), leptin (LEP, RS = 61.27810669) and arylsulfatase A (ARSA, RS = 54.54859161). Similarly, top 10 targets of APN + AdipoR1 + IBD except APN + AdipoR1 + AdipoR2 + IBD (Table S2) are interleukin 2 receptor subunit alpha (IL2RA, RS = 38.22231674), CRP (RS = 28.32578087), NLR family pyrin domain containing 3 (NLRP3, RS = 16.74045563), C-X-C motif chemokine receptor 4 (CXCR4, RS = 16.13383293), nucleotide binding oligomerization domain containing 2 (NOD2, RS = 15.06386757), TNF receptor superfamily member 11b (TNFRSF11B, RS = 14.31340408), RUNX Family Transcription Factor 1 (RUNX1, RS = 13.18826866), Wolframin ER transmembrane glycoprotein (WFS1, RS = 12.76303864), catenin beta 1 (CTNNB1, RS = 12.62774658) and bone morphogenetic protein 2 (BMP2, RS = 10.8545351). Top 10 targets of APN + AdipoR2 + IBD except APN + AdipoR1 + AdipoR2 + IBD (Table S3) are JAK3 (RS = 66.93528748), lecithin-cholesterol acyltransferase (LCAT, RS = 36.63467789), Von Willebrand factor (VWF, RS = 24.57114792), solute carrier family 17 member 5 (SLC17A5, RS = 24.19079781), lysosomal associated membrane protein 2 (LAMP2, RS = 23.16798019), fibrillin 1 (FBN1, RS = 19.23112297), transforming growth factor beta 1 (TGFB1, RS = 19.12390709), ATP binding cassette subfamily G member 5 (ABCG5, RS = 18.88196945), serpin family F member 1 (SERPINF1, RS = 18.5030632) and microsomal triglyceride transfer protein (MTTP, RS = 18.08280373).

### Construction and analysis of interaction network

*Core proteins of the interaction network are not necessarily in top 10 RS targets.* The APN + AdipoR1 + AdipoR2 + IBD, APN + AdipoR1 + IBD and APN + AdipoR2 + IBD targets with APN RS ≥ 10 are selected for interaction network Table S1-S3). Top 10 targets with highest sting node degrees (SND) of Homo sapiens APN + AdipoR1 + AdipoR2 + IBD (Fig. 4F, Figure S17 and Table S4) are insulin (INS, SND = 108), glyceraldehyde-3-phosphate dehydrogenase (GAPDH, SND = 99), AKT serine/threonine kinase 1 (AKT1, SND = 93), peroxisome proliferator-activated receptor gamma (PPARG, SND = 93), albumin (ALB, SND = 90), IL6 (SND = 86), TNF (SND = 85), peroxisome proliferator-activated receptor alpha (PPARA, SND = 79), leptin (LEP, SND = 78) and tumor protein P53 (TP53, SND = 78), which is differed from PPARG coactivator 1 alpha (Ppargc1a, SND = 72) and sirtuin 1 (Sirt1, SND = 68) of mouse musculus APN + AdipoR1 + AdipoR2 + IBD (Fig. 4G, Figure S18 and Table S5). Top 10 targets with highest SND of Homo sapiens APN + AdipoR1 + IBD excepting APN + AdipoR1 + AdipoR2 + IBD (Fig. 4H, Figure S17 and Table S6) are IL6 (SND = 13), C-X-C motif chemokine ligand 10 (CXCL10, SND = 9), forkhead box protein P3 (FOXP3, SND = 9), CTNNB1 (SND = 8), CXCR4 (SND = 8), CRP (SND = 7), IL2RA (SND = 7), bone gamma-carboxyglutamate protein (BGLAP, SND = 6), BMP2 (SND = 6), NLRP3 (SND = 6) and RUNX1 (SND = 6), which is distinguished from colony stimulating factor 2 (Csf2, SND = 8), C-X-C motif chemokine 15 (Cxcl15, SND = 7), osteocalcin-2 (Bglap2, SND = 5), osteocalcin-3 (Bglap3, SND = 5) and Nod2 (SND = 4) of mouse musculus APN + AdipoR1 + IBD excepting APN + AdipoR1 + AdipoR2 + IBD (Fig. 4I, Figure S18 and Table S7). Top 10 targets with highest SND of Homo sapiens APN + AdipoR2 + IBD excepting APN + AdipoR1 + AdipoR2 + IBD (Fig. 4J, Figure S17 and Table S8) are apolipoprotein E (APOE, SND = 16), CRP (SND = 13), TGFB1 (SND = 13), TIMP metallopeptidase inhibitor 1 (TIMP1, SND = 13), VWF (SND = 13), apolipoprotein A1 (APOA1, SND = 12), Caspase 3 (CASP3, SND = 12), scavenger receptor class B member 2 (SCARB2, SND = 12), angiotensin I converting enzyme (ACE, SND = 12) and cholesteryl ester transfer protein (CETP, SND = 12), which is sifted out from (Agt, SND = 14), (Plg, SND = 13) and Il18 (SND = 13) of mouse musculus APN + AdipoR2 + IBD excepting APN + AdipoR1 + AdipoR2 + IBD (Fig. 4K, Figure S18 and Table S9). To further explore the interaction network between core targets and APN, targets with APN RS ≥ 10, IBD RS ≥ 50 and top 10 highest SND are used for subsequent analysis. IL6, TNF, TP53, INS, PPARG, ALB in APN + AdipoR1 + AdipoR2 + IBD (Fig. 4L-M and R-S), CRP, NLRP3, IL2RA, CTNNB1 in APN + AdipoR1 + IBD excepting APN + AdipoR1 + AdipoR2 + IBD (Fig. 4N-O and T-U) and TGFB1, VWF, ACE in APN + AdipoR2 + IBD excepting APN + AdipoR1 + AdipoR2 + IBD (Fig. 4P-Q and V-W) show highest correlation with APN, AdipoR1 and AdipoR2.

### Preliminary analysis of intersection targets

*APN regulates CD through AdipoR1/2-metabolism process and UC through AdipoR1-inflammation axis or AdipoR2-fibrosis process.* Intersection targets of APN + AdipoR1 + AdipoR2 + IBD are mainly based on metabolic factors INS, GAPDH, PPARG, AKT1, PPARA, PPARA in Homo sapiens and AKT1, PPARG, PPARA, PPARGC1α, SIRT1 in Mouse musculus, where less are inflammatory factors including TNF and IL6 in Homo sapiens or Mouse musculus (Fig. 4A and F-G). Most cross-targets of APN + AdipoR1 + IBD are inflammatory factors including IL6, FOXP3, CXCL10, CX CR4, IL2RA, CRP, NLRP3 in Homo sapiens and CSF2, CXCL5, IL2RA, CRP, NLRP3, CXCR4, NOD2 in Mouse musculus (Fig. 4A and H-I). Surprisingly, intersection targets of APN + AdipoR2 + IBD also involve fibrosis-related proteins TIMP1, TGFB1 in Homo sapiens and TIMP1, TGFB1, PLG in Mouse musculus except for inflammatory factors and metabolic factors (Fig. 4A and J-K). We speculate that APN may modulate metabolic process through AdipoR1/2, regulate immunity by AdipoR1, and adjust metabolic process, immunity and fibrosis process via AdipoR2, thereby interfering with IBD. Moreover, APN perhaps regulate CD through AdipoR1/2-metabolism process and UC through AdipoR1-inflammation process or AdipoR2-fibrosis process, because the symptoms of CD are closely related to metabolism [16, 18–20] and UC is intimately correlated to immunity/ colonic fibrosis [4, 14, 17, 21].

### Functional enrichment

The top 10 intersection targets does not closely match with first 10 core proteins of the interaction network, so functional enrichment is used to further explore the specific physiological functions of these targets. Meanwhile, APN + AdipoR1 + AdipoR2 + IBD, APN + AdipoR1 + IBD and APN + AdipoR2 + IBD targets with APN RS ≥ 10 are selected for Gene ontology (GO), canonical and Kyoto Encyclopedia of Genes and Genomes (KEGG) pathways enrichment.

#### GO enrichment

**Fig. 5:**
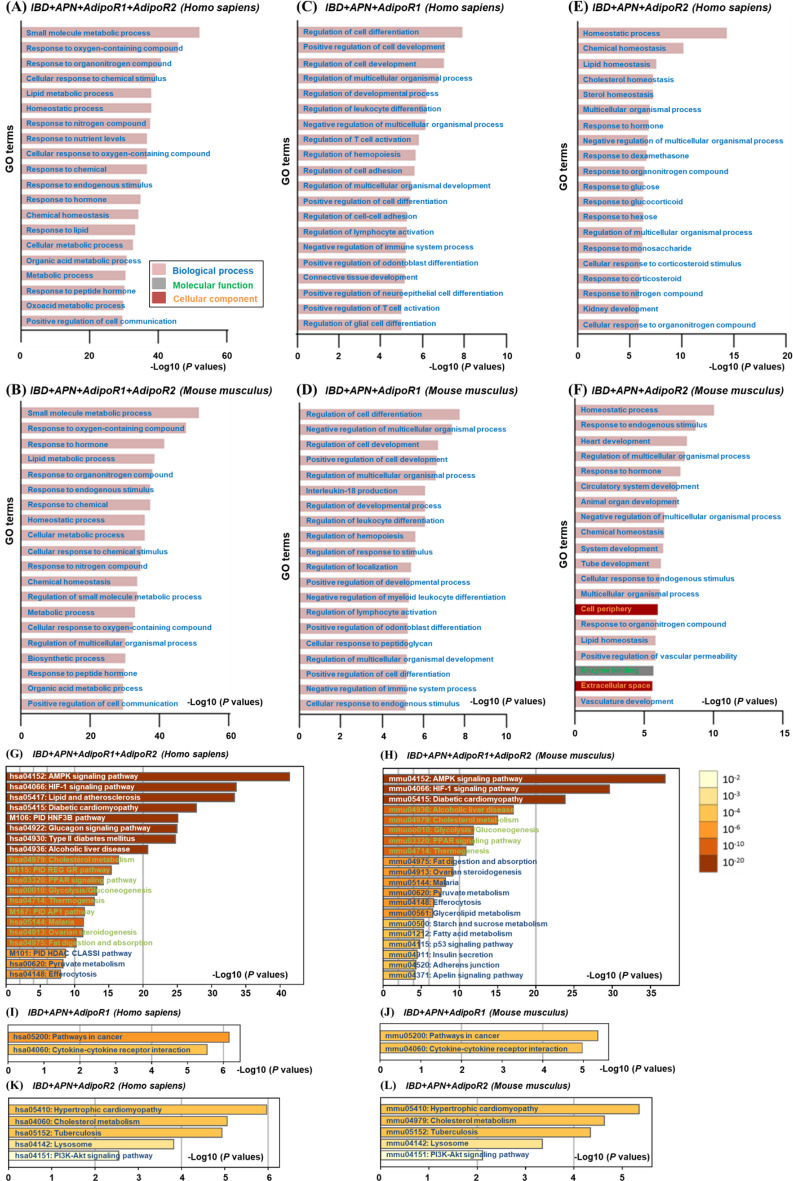
GO (**A**-**F**), canonical pathways and KEGG pathways (**G**-**L**) enrichment of intersection targets. The APN + AdipoR1 + AdipoR2 + IBD (**A**-**B**, **G**-**H**), APN + AdipoR1 + IBD (**C**-**D**, **I**-**J**) and APN + AdipoR2 + IBD (**E**-**F**, **K**-**L**) targets with APN relevance score ≥ 10 are selected for GO, canonical pathways and KEGG enrichment. Top 20 GO enrichments are showed in the figures. GO: Gene ontology; KEGG: Kyoto Encyclopedia of Genes and Genomes; APN: adiponectin; AdipoR1: adiponectin receptor 1; AdipoR2: adiponectin receptor 2; IBD: inflammatory bowel disease. Available (November 2024): https://www.geneontology.org/; https://metascape.org/gp/index.html#/main/step1

*APN perhaps interfere with cell communication*,* lipid*,* organic acid*,* oxygen-containing compounds*,* organonitrogen compounds and peptide hormone metabolism in IBD individuals through AdipoR1 or AdipoR2.* Top 20 GO enrichment of both Homo sapiens (Fig. 5A) and mouse musculus (Fig. 5B) intersection targets of APN + AdipoR1 + AdipoR2 + IBD with APN RS ≥ 10 (Table S1) profoundly influences small molecule, lipid and organic acid metabolic process, cellular response to oxygen-containing compound, organonitrogen compound, chemical stimulus, peptide hormone and endogenous stimulus, homeostatic process, positive regulation of cell communication.

*Mechanism of APN in IBD through AdipoR1 without AdipoR2 may be attributed to neurologic and peripheral immune regulation.* Top 20 GO enrichment of either Homo sapiens (Fig. 5C) or mouse musculus (Fig. 5D) intersection targets of APN + AdipoR1 + IBD with APN RS ≥ 10 (Table S2) excepting APN + AdipoR1 + AdipoR2 + IBD plays a key role on positive regulation of leukocyte, odontoblast, lymphocyte differentiation and activation, multicellular organismal development, hemopoiesis, negative regulation of immune system process. Homo sapiens APN + AdipoR1 + IBD targets also regulate T cell, neuroepithelial cell, glial cell differentiation and activation, cell adhesion, connective tissue development (Fig. 5C), while mouse musculus APN + AdipoR1 + IBD targets extra affect interleukin-18 production, cellular response to endogenous stimulus, localization and negative regulation of myeloid leukocyte differentiation (Fig. 5D).

*APN potentially interfere with nutrition and energy homeostasis through AdipoR2 without AdipoR1*,* thus affecting the peripheral phylogeny of IBD.* Top 20 GO enrichment of both Homo sapiens (Fig. 5E) and mouse musculus (Fig. 5F) intersection targets of APN + AdipoR2 + IBD with APN RS ≥ 10 (Table S3) excepting APN + AdipoR1 + AdipoR2 + IBD play a crucial part in lipid and chemical homeostasis, negative regulation of multicellular organismal process, response to hormone and organonitrogen compound. Moreover, Homo sapiens APN + AdipoR2 + IBD targets modulate cholesterol and sterol homeostasis, cellular response to dexamethasone, glucose, glucocorticoid, hexose, monosaccharide, corticosteroid stimulus and nitrogen compound, kidney development (Fig. 5E), while mouse musculus APN + AdipoR2 + IBD targets regulate cellular response to endogenous stimulus, heart, circulatory system, vasculature, tube, animal organ and system development, cell periphery, vascular permeability, enzyme binding, extracellular space (Fig. 5F). APN likely regulate lipids, glucose, sterols, cholesterol, corticosteroids and nitride homeostasis of IBD through AdipoR2 without AdipoR1, thus affecting peripheral systemic development such as heart, kidney, circulatory system, vasculature and vascular permeability.

#### Canonical and KEGG pathways enrichment

*APN mediates AMPK*,* HIF-1*,* PPAR*,* REG GR*,* AP1*,* HDAC*,* p53 and apelin signaling pathways through AdipoR1 or AdipoR2 to affect IBD progression.* Either Homo sapiens (Fig. 5G) or mouse musculus (Fig. 5H) intersection targets of APN + AdipoR1 + AdipoR2 + IBD with APN RS ≥ 10 regulate AMPK, hypoxia-inducible factor 1 (HIF-1) and peroxisome proliferator-activated receptor (PPAR) signaling pathway, diabetic cardiomyopathy, alcoholic liver disease, cholesterol metabolism, glycolysis/ gluconeogenesis, thermogenesis, fat digestion and absorption, ovarian steroidogenesis, malaria, pyruvate metabolism, efferocytosis. Additionally, Homo sapiens APN + AdipoR1 + AdipoR2 + IBD targets are associated with hepatocyte nuclear factor-3beta gene (HNF3B), glucagon, glucocorticoid receptor (REG GR), activator protein 1 transcription factor (AP1) and histone deacetylase (HDAC) class I pathway, lipid and atherosclerosis, type II diabetes mellitus (Fig. 5G), while that of mouse musculus influence glycerolipid, starch, sucrose and fatty acid metabolism, p53 and apelin signaling pathway, insulin secrenion, adherens junction (Fig. 5H). APN regulates cell growth by activating AMPK, suppressing mTOR, increasing p53-p21 axis and activating ribosomal protein S6, thereby reducing protein translation and insulin sensitivity [24]. Either fAPN or gAPN promote tissue proliferation by blocking protein kinase A/C and adenylate cyclase [24]. AMPK, a highly conserved sensor of low intracellular ATP level [39]regulates mitochondrial homeostasis, autophagy, lipid and glucose metabolism by phosphorylating its downstream proteins mTOR, HDAC and PPAR-γ [39–41]. APN possibly phosphorylate mTOR and HDAC by activating AMPK and PPAR, which promotes energy metabolism and mitochondrial autophagy during IBD.

*APN adjusts PI3K-Akt signaling pathway through AdipoR2 instead of AdipoR1 to influence IBD.* Except for the intersection with AdipoR2, the intersection target enrichment of Homo sapiens (Fig. 5I) and mouse musculus (Fig. 5J) APN + AdipoR1 + IBD is only related to pathways in cancer and cytokine-cytokine receptor interaction. Meanwhile, both Homo sapiens (Fig. 5K) and mouse musculus (Fig. 5L) APN + AdipoR2 + IBD intersection target enrichment include hypertrophic cardiomyopathy, cholesterol metabolism, tuberculosis, lysosome and PI3K-Akt signaling pathway. APN regulates cell growth in two ways, one is to inhibit leptin-induced NF-kB-dependent autocrine IL-6 production and trans-IL-6 signaling to pre-neoplastic colon epithelial cells, and the other is to inhibit IL-6-induced cell proliferation by reducing the phosphorylation and activation of STAT-3 in advanced colon cancer cells [24]. APN deficiency activates PI3K-Akt pathway through upstream protein tyrosine kinase Src and IL-6 to inhibit or phosphorylate downstream targets mTOR, resulting in cell survival, growth and cycle in carcinogenesis [42]. APN also reduces TNF-α secreted by macrophage and anti-TNF-α drugs have become clinical drugs for IBD for its activities on inhibit the progression of colitis-related colon cancer [42], so APN and its receptor agonists are expected to become one of clinical treatment strategies for IBD. PI3K-Akt signaling promotes inflammation and induces immune suppression through its downstream genes NF-kB and TNF-α [43]. Therefore, APN may play an anti-inflammatory role in IBD by inhibiting PI3K-Akt pathways and inactivating NF-kB and TNF-α, which delays the progression of IBD.

#### Molecular Docking

**Fig. 6:**
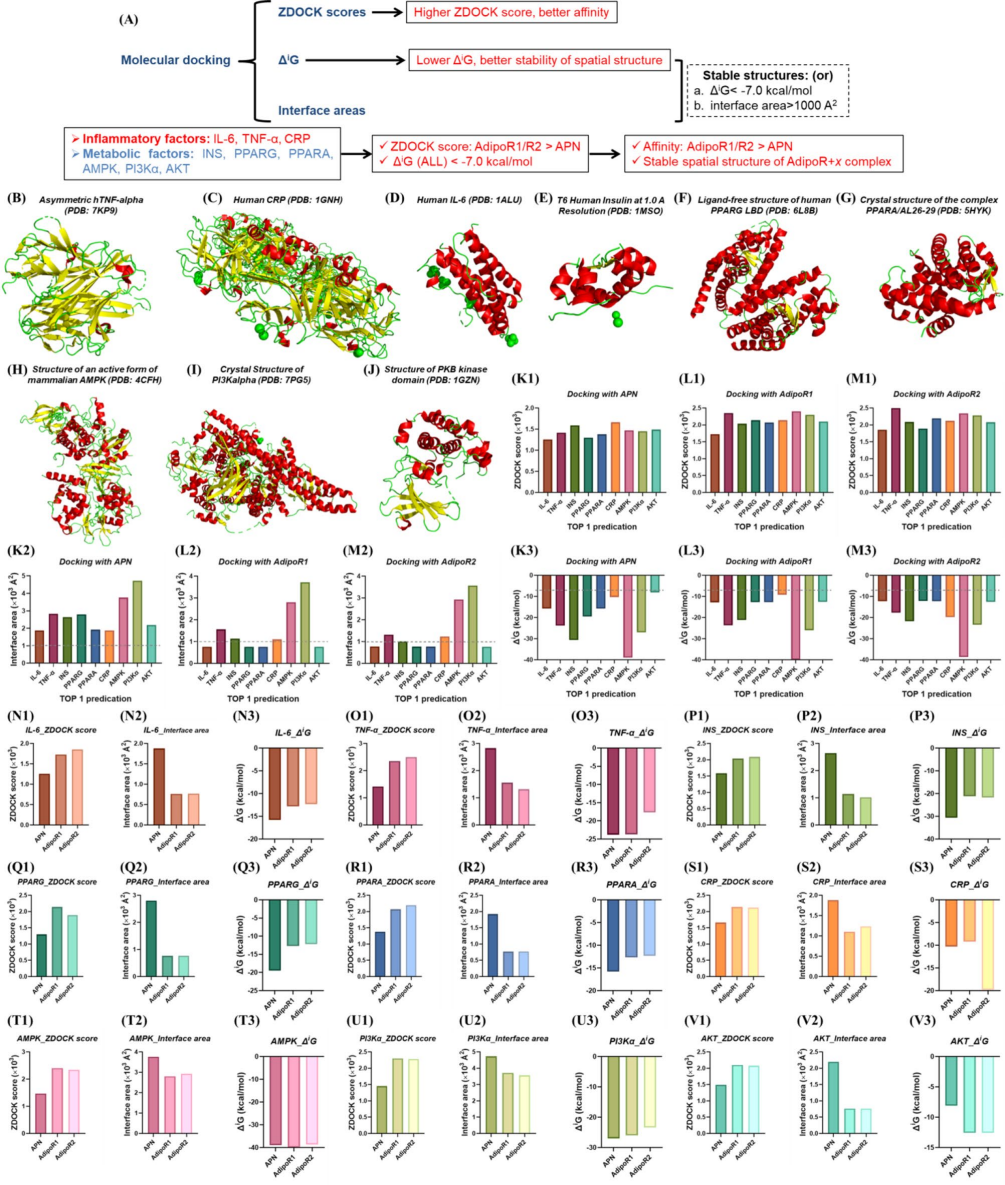
ZDOCK scores, interface area and Δ^i^G of IL-6, TNF-α, INS, PPARG, PPARA, CRP, AMPK, PI3Kα and AKT respectively docking with APN, AdipoR1 and AdipoR2. (**A**) summary of molecular docking; (**B**-**J**) spatial structure of IL-6, TNF-α, INS, PPARG, PPARA, CRP, AMPK, PI3Kα and AKT; (**K1**-**V1**) ZDOCK scores of different factors respectively docking with APN, AdipoR1 and AdipoR2; (**J2**-**U2**) interface area of different factors respectively docking with APN, AdipoR1 and AdipoR2; (**K3**-**V3**) Δ^i^G of different factors respectively docking with APN, AdipoR1 and AdipoR2. TOP 1 complex with highest ZDOCK score are analyzed in the figures by molecular docking. ZDOCK is used for molecular docking and PDBePISA is applied to analyze docking results. (**B**-**J**) red: secondary structure helix (ss h), yellow: secondary structure beta sheet (ss s), green: secondary structure loop and other structures (ss l+). The PDB ID of APN, AdipoR1 and AdipoR2 used for molecular docking are 6U66, 5LXG and 6KS1. APN: adiponectin; AdipoR1: adiponectin receptor 1; AdipoR2: adiponectin receptor 2; Δ^i^G: Gibbs free energy. Available (November 2024): https://zdock.wenglab.org/; https://www.ebi.ac.uk/msd-srv/prot_int/

*IL-6*,* TNF-α*,* INS*,* PPARG*,* PPARA*,* CRP*,* AMPK*,* PI3Kα and AKT are more likely to interact with AdipoR1/2 than with APN.* Intersection targets with APN RS ≥ 10 (Table S2), IBD RS ≥ 50 (Table S10), highest sting node degrees (Fig. 4R-W), significant canonical and KEGG pathways enrichment (Fig. 5G-L) are taken for molecular docking with APN, AdipoR1 and AdipoR2 to explore their affinity. The binding probability and complex stability are evaluated by ZDOCK scores and Δ^i^G, respectively (Fig. 6A). Whether IL-6, TNF-α, INS, PPARG, PPARA, CRP, AMPK, PI3Kα, AKT interacts with APN and AdipoR/2 has not been specifically reported, so their interaction probability are explored through molecular docking. The spatial structures of TNF-α and CRP are mainly beta sheet (Fig. 6B-C), while IL-6, INS, PPARG PPARA, AMPK, PI3Kα and AKT (Fig. 6D-J) are taken helix structures as principal. ZDOCK scores of IL-6, TNF-α, INS, PPARG, PPARA, CRP, AMPK, PI3Kα and AKT docking with AdipoR1 and AdipoR2 are higher than that of APN (Fig. 6K1-M3, N1-V1 and Figure S19-S45), indicating that AdipoR1/2 had better affinity than APN. APN, a kind of secreted protein, may activate downstream signaling molecules mainly through AdipoR1/2 to regulate various physiological processes. Although the binding interface area of each target and AdipoR1/2 is quite different (Fig. 6K1-M3, N2-V2 and Figure S19-S45), Δ^i^G< -7.0 kcal/mol (Fig. 6K1-M3, N3-V3 and Figure S19-S45) belonging to stable structures.

**Fig. 7:**
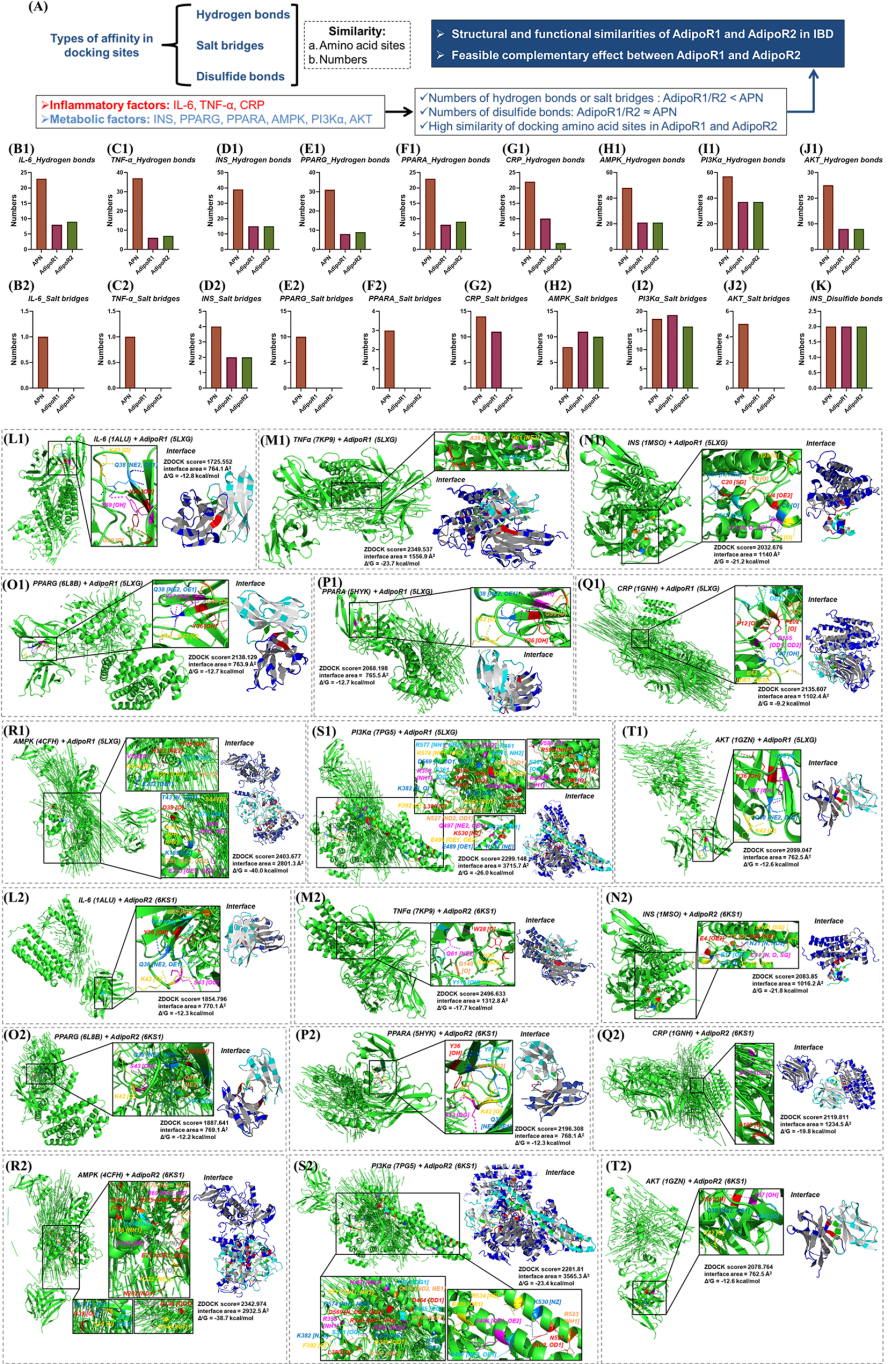
Amino acid sites of AdipoR1 and AdipoR2 interacts with different factors. (**A**) summary and predication of molecular docking; (**B**-**K**) numbers of hydrogen bonds, salt bridges and disulfide bonds of APN, AdipoR1/R2 docking with IL-6, TNF-α, INS, PPARG, PPARA, CRP, AMPK, PI3Kα and AKT; (L1-T1) IL-6, TNF-α, INS, PPARG, PPARA, CRP, AMPK, PI3Kα and AKT respectively docking with AdipoR1; (L2-T2) IL-6, TNF-α, INS, PPARG, PPARA, CRP, AMPK, PI3Kα and AKT respectively docking with AdipoR2. APN: adiponectin; AdipoR1: adiponectin receptor 1; AdipoR2: adiponectin receptor 2; IL-6: interleukin-6; TNF-α: tumor necrosis factor α; INS: insulin; PPARG: peroxisome proliferator-activated receptor gamma; PPARA: peroxisome proliferator-activated receptor alpha; CRP: C-reactive protein; AMPK: adenosine 5’-monophosphate (AMP)-activated protein kinase; PI3Kα: phosphatidylinositol 3-kinase α; AKT: protein kinase B. Available (November 2024): https://zdock.wenglab.org/; https://www.ebi.ac.uk/msd-srv/prot_int/

*AdipoR1 and AdipoR2 may be complementary in IBD.* Although the probability of interaction between APN and IL-6, TNF-α, INS, PPARG, PPARA is lower than that of AdipoR1/2, their interaction areas are larger (Fig. 6K1-M3, N2-R2) and Δ^i^G is lower than that of AdipoR1/2 (Fig. 6K1-M3, N3-R3). Numbers of hydrogen bonds and salt bridges in APN docking with IL-6, TNF-α, INS, PPARG, PPARA, CRP, AMPK, PI3Kα and AKT are more than that of AdipoR1/2 (Fig. 7A-J), although numbers of disulfide bonds in APN, AdipoR1/2 docking with INS are indistinctive (Fig. 7J). Besides, the sites where APN interacts with IL-6, TNF-α, INS, PPARG, PPARA, CRP, AMPK, PI3Kα and AKT are also diverse (Figure S46-S54). Surprisingly, location and mode of action sites of AdipoR1 and IL-6, TNF-α, INS, PPARG, PPARA, AMPK, PI3Kα, AKT are similar to that of AdipoR2 (Fig. 7L-T). Structure and function of AdipoR1 and AdipoR2 are highly similar, so a feasible complementary effect between AdipoR1 and AdipoR2 may exist in IBD. The dual agonist of AdipoR1/2 enhances mitochondrial function by activating AMPK [44]PI3K-AKT [45] and PPARG signaling pathways [46]also indicating that physiological functions of AdipoR1 and AdipoR2 are complementary rather than contradictory.

*TNF-α*,* AMPK may interact directly with AdipoR1.* TOP 1 complex ZDOCK score of TNF-α (2349.537, Fig. 6L1 and 6O1) and AMPK (2403.677, Fig. 6L1 and 6T1) docking with AdopoR1 are higher than that of APN (2099.047, Fig. 3B), so they possess a high probability of interacting directly with AdopoR1. ZDOCK score of TNF-α docking with AdipoR1/2 is higher than that of other targets, possibly resulting from the high structural similarity between TNF-α and APN [24–26]. Meanwhile, TOP 1 complex ZDOCK score of TNF-α (2349.537, 2496.633), INS (2032.676, 2083.85), PPARG (2138.129), PPARA (2068.198, 2196.308), CRP (2135.607, 2119.811), AMPK (2403.677, 2342.974), PI3Kα (2299.148, 2281.81), AKT (2099.047, 2078.764) docking with AdipoR1/2 (Fig. 6L1-V1) are close to that of APN (2303.998, 2535.086, Fig. 3B-C), indicating that they have possibility of direct interaction with AdipoR1/2.

*AdipoR1 and AdipoR2 interact directly with APPL1 to mediate AMPK*,* PPARA*,* PI3K-AKT*,* p38-MAPK*,* IRS1/2 signaling pathway.* APN binds to its receptor AdipoR1/2 and mediates the direct interaction between AdipoR1/2 and pleckstrin homology domain, phosphotyrosine binding domain, and leucine zipper motif 1 (APPL1), which regulates the downstream AMPK, PPARA, PI3K-AKT, p38 MAPK, insulin receptor susbstrate-1/2 (IRS1/2) signaling pathway [47]. TOP 1 complex ZDOCK scores of APPL1 docking with APN, AdipoR1/2 are AdipoR2 > AdipoR1 > APN (Figure S55-S58), and their complex structure are stable (Δ^i^G< -7.0 kcal/mol, Figure S55D) in spite of interface areas of APPL1 docking with AdipoR1/2 are small (Figure S55C-G). Additionally, the affinity of APPL1 to AdipoR2 (ZDOCK score = 3193.088) is higher than AdipoR1 (ZDOCK score = 2779.741).

## Translational perspective

### Potential of targeted interventions

*AdipoRon potentially alleviates CD symptoms by reducing the levels of TNF-α and IL-6.* APN inhibits the phagocytic activity and production of TNF-α and IL-6 in colonic macrophages. In CD, low expression of APN in the colon is accompanied by increase in TNF-α and IL-6 levels [18, 20]. Target intersection analysis shows that APN modulates TNF-α and IL-6 through AdipoR1/2 (Fig. 4A, F-G), so AdipoRon, a dual agonist of AdipoR1/2 [46], potentially plays a crucial part in anti-inflammatory effects and alleviates CD symptoms by reducing the levels of TNF-α and IL-6.

*AdipoRon feasibly relieves IBD by aggrandizing P-AKT*,* PPARA*,* PPARG levels by AdipoR1/2 and weakening NLRP3 activities by AdipoR1.* High expression of TNF-α induces low level phosphorylation of AKT (P-AKT), which aggravates IBD by increasing reactive oxygen species (ROS) and abducting apoptosis [48, 49]. Increased PPARA and PPARG, transcription factors promoting alternatively activated (M2) macrophages polarization, limits chronic inflammation in IBD [46, 50]. Oppositely, NLRP3 inflammasome activity and NF-κB activation abducts classically activated (M1) macrophage phenotype [51], so preventing polarization of pro-inflammatory M1 macrophages and NLRP3 inflammasome responses may restrain IBD progression. APN modulates AKT, PPARA, PPARG pathways through AdipoR1/2 and NLRP3 through AdipoR1 (Fig. 4A, F-I). Meanwhile, AdipoRon enhances P-AKT, PPARA, PPARG levels and weakens NLRP3 activities [46, 52, 53]. Hence, AdipoRon may be a promising drug for treating IBD.

*AdipoR2 agonist possibly reduces colonic fibrosis in UC and fistulae in CD by restoring balance of TIMP1 and TGFB1.* High-level APN is not necessarily beneficial, and the dose-effect relationship between APN and IBD is not simply dose-dependent. High expression of APN promotes colonic fibrosis in UC [17] and fistulae in CD [54, 55], which is related to low expression of TIMP1^54,56^ and high expression of TGFB1 [55, 57]. APN interferes with the expression of TIMP1 and TGFB1 in IBD by targeting AdipoR2 (Fig. 4A, J-K), so emodin succinate monoethyl ester (ESME), an AdipoR2 agonist [58] possibly recedes the severity of colonic fibrosis in UC and fistulae in CD by restoring balance of TIMP1 and TGFB1.

### Intestinal barrier integrity and Microbiome homeostasis

Dysregulated epithelial barrier in IBD leads to an increase in infiltration of pathogenic microorganisms, abnormal inflammatory infiltration, and a decrease in goblet cell numbers, thickness of intestinal muscle, length of intestinal villi, crypt depth. This circulation of intestinal barrier dysfunction and inflammation ulteriorly allows for epithelial deficiency and increased susceptibility to invasion, resulting in impairing intestinal function and intestinal microbiome homeostasis [59, 60]. Maintaining intestinal barrier integrity and managing dysfunctional intestinal epithelium by promoting mucosal healing and regulating intestinal microbiota is a novel therapeutic strategy for IBD [59].

#### Mucosal healing

*APN probably promotes mucosal healing in IBD through INS*,* IRS1/2*,* AKT and mTOR signaling pathways of AdipoR1/2-IGF axis.* A weak mucosal barrier is one of the common pathological features of IBD. Insulin-like growth factor-1 (IGF-1) treatment restores mucosal barrier, characterized by healthy expression of mucin, E-cadherin and β-catenin, structural maintenance of adhesion junction, reduction of immune infiltration and matrix metalloproteinase-2 levels [61]. The characteristic of endogenous IGF-1 desensitization in IBD is the downregulation of IRS1/2, AKT and mTOR signaling cascade reaction [61] which is also the downstream signaling axis regulated by APN through AdipoR1/2 [17, 22, 47]. Exogenous supplementation of APN or dual agonists of AdipoR1/2 may restore physiological equilibrium of IRS1/2, AKT and mTOR in colons of IBD individuals, and reduce IGF-1 endogenous desensitization, which possibly exerts a similar therapeutic effect as IGF-1 treatment. IGF-1 and INS are highly homologous hormones with structural and functional similarity (Figure S59), playing significant roles in metabolism and growth development [62]. Moreover, receptors of IGF-1 and INS (IGF1R and INSR) are also highly homologous, further elaborating on the structural and functional isogeny of IGF-1 and INS. APN disturbs human IBD by AdipoR1/2-INS axis (Fig. 4A and F), so the probability that APN regulates IBD through AdipoR1/2-IGF axis is also not low basing on homology comparison. In other words, APN probably promotes mucosal healing in IBD through INS, IRS1/2, AKT and mTOR signaling pathways of AdipoR1/2-IGF axis.

#### Gut microbiota

*APN restores microbial diversity and beneficial bacteria abundance through AdipoR1-NOD2 axis.* 99% intestinal microbiota is constituted of *Firmicutes*, *Bacteroidetes*, *Actinobacteria* and *Proteobacteria*, among which *Firmicutes* and *Bacteroidetes* account for approximately 90% [63]. Although gut dysbiosis is not the main driving factor for IBD, it can exacerbate immune imbalance and progression of the disease, including incurable wounds, fibrosis, strictures, fistulas formation, abscess, depression and other complications associated with IBD [5, 63]. NOD2, one of major IBD susceptibility genes and bacterial sensor, is a receptor of muramyl dipeptide which is critical constituent of both Gram-positive and Gram-negative bacteria and NOD2 mutations induce damaged epithelial clearance of invasive bacteria, dysfunction of Paneth cells, reduced production of antimicrobial peptide, decreased microbial diversity and beneficial bacteria abundance [64–66]. APN affects NOD2 activities in mouse with IBD through AdipoR1 (Fig. 4A and I), so the strategy of treating gut dysbiosis in IBD through APN has certain promising prospects.

*APN renovates intestinal microbiota homeostasis in IBD through AdipoR1/2-SIRT1 signaling pathway.* IBD is closely related to disruption of intestinal epithelial homeostasis which is maintained through complicated interactions among epithelial cells, commensal gut microbiota and immune cells [67]. SIRT1, a conserved NAD^+^-dependent protein deacetylase in mammals and a vital mediator of host-microbiome interactions, perceives environmental stress to transform intestinal integrity [68]. Intestinal SIRT1-deficient mice with defective gut microbiota exhibit more severe colitis than the control mice when induced by dextran sodium sulfate [69, 70]. Additionally, SIRT1 level in intestines of UC patients is decreased [69]. APN alters SIRT1 expression in murine IBD through AdipoR1/2 (Fig. 4A and G). Therefore, APN may renovate intestinal microbiota homeostasis in IBD through SIRT1-AdipoR1/2 signaling pathway, thereby treating intestinal inflammation.

#### Role of adiporon in AdipoR1/2-IGF and AdipoR1/2-SIRT1 axis in IBD

*AdipoRon increases autophagy induced by IGF-1 and SIRT1 to alleviate IBD.* IGF1R activation and translocation increases autophagic flux and oxidative phosphorylation [71, 72]. IGF-1 supplement activates IGF1R and P-AKT, resulting in rising autophagy and declining ROS [48, 49]. APN promotes mucosal healing in IBD through INS, IRS1/2, AKT and mTOR signaling pathways of AdipoR1/2-IGF axis. Besides, impaired SIRT1-autophagy is one of the pathogenic mechanisms of IBD [68]. AdipoRon activates SIRT1 through AdipoR1/AMPK-dependent nuclear translocation of GAPDH, and subsequently enhances autophagy [73]. APN renovates intestinal microbiota homeostasis in IBD through AdipoR1/2-SIRT1 signaling pathway. Consequently, AdipoRon probabilistically increases autophagy induced by IGF-1 and SIRT1 to promote mucosal healing and intestinal microbiota homeostasis in IBD.

### Unresolved issues and future directions

Although APN potentially possesses multiple targets in IBD treatment, specific mechanism of action and embedded research is needed to clarify its participant signaling pathways and biological effects. Besides, more clinical data are needed to verify the effectiveness and safety of APN in IBD treatment for it has not yet been applied in the clinical strategy of IBD.

Extensive patient heterogeneity is a challenge in IBD management, which is closely related to intersection between gender and social identities. The etiology, incidence rates and risk factors of different IBD subtypes differ significantly between male and female, where CD predominance and severity is higher in female with opposite situation in UC [74, 75]. Nevertheless, overall incidence of UC is higher than CD [76]. Colorectal cancer secondary to highly inflammatory chronic UC is more common in male than in female [75]. Awareness of gender-specificity, gender-related symptoms and disease phenotypes is conducive to achieving tailored treatment including prevention or reduction of complications. Treatment preference of APN for UC and CD remains undetermined and requires further investigation.

Currently, APN analogues or AdipoR agonists such as AdipoRon are mostly administered orally, resulting in an enormously efficient dosage (AdipoRon 50 mg/kg gavage in mice) [46]. Effectively delivering low-dose adiponectin to the sites of intestinal inflammation is also an intricate challenge and nanoparticle delivery systems may be an available solution [77]. Furthermore, APN, an endogenous natural drug with minimal side effects, may have potential to be combined with other treatment methods to enhance IBD therapeutic effect.

## Conclusion and prospect

APN and AdipoR2 proteins are highly expressed in colon which is a primary organ of IBD, and the target intersection of APN and IBD is huge. APN may interfere with cell communication, lipid, organic acid, oxygen-containing compounds, organonitrogen compounds and peptide hormone metabolism in IBD individuals through AdipoR1 or AdipoR2, but regulates neural and peripheral immune by AdipoR1 but not AdipoR2 and mediates nutritional and energy homeostasis through AdipoR2 but not AdipoR1. APN regulates CD through AdipoR1/2-metabolism process and UC through AdipoR1-inflammation axis or AdipoR2-fibrosis process. Additionally, APN influences IBD progression by mediating AMPK, HIF-1, PPAR, HDAC and p53 signaling pathways through AdipoR1 or AdipoR2, while it regulate PI3K-Akt signaling pathway through AdipoR2 rather than AdipoR1. AdipoR1 and AdipoR2 interact directly with APPL1 to mediate AMPK, PPARA, PI3K-AKT, p38-MAPK, IRS1/2 signaling pathway. The APN + AdipoR1 + AdipoR2 + IBD target intersections with the highest confidence are more likely to interact with AdipoR1/2 than with APN, including IL-6, TNF-α, INS, PPARG, PPARA, CRP, AMPK, PI3Kα and AKT. Unexpectedly, AMPK and TNF-α may interact directly with AdipoR1. APN analogues or AdipoRon which is a dual agonist of AdipoR1/2 potentially alleviates CD symptoms by reducing the levels of TNF-α and IL-6, relieves IBD by aggrandizing P-AKT, PPARA, PPARG levels by AdipoR1/2 and weakening NLRP3 activities by AdipoR1, reduces colonic fibrosis in UC and fistulae in CD by restoring balance of TIMP1 and TGFB1, promotes mucosal healing through INS, IRS1/2, AKT and mTOR signaling pathways of AdipoR1/R2-IGF axis, restores microbial diversity and beneficial bacteria abundance through AdipoR1-NOD2 axis and AdipoR1/2-SIRT1 signaling pathway to repair intestinal microbiota homeostasis, and increases autophagy induced by IGF-1 and SIRT1 to alleviate IBD. Above cases are based only on literature reviews and molecular dynamics simulation, so further experiments are required to validate these relevant perspectives and provide direct evidences.

## Electronic supplementary material

Below is the link to the electronic supplementary material.

Supplementary Material 1

## Data Availability

No datasets were generated or analysed during the current study.
