# Supplementary material for: Role of adiponectin and its receptors AdipoR1/2 in inflammatory bowel disease
Source: Cell Commun Signal. 2025 Jul 26;23:356. doi: 10.1186/s12964-025-02359-w (PMC12297855; doi:10.1186/s12964-025-02359-w)
Supplement: Supplementary file 1 — Supplementary Material 1 [file 12964_2025_2359_MOESM1_ESM.docx]

**Role of Adiponectin and Its Receptors AdipoR1/2 in Inflammatory Bowel Disease**

Qiuyan Zhu^1, *^, Xiaoli Jia^1, *^, Shupeng Li^1,^ ^2, #^, Jinxing Feng^3, #^

1. State Key Laboratory of Chemical Oncogenomics, Shenzhen Graduate School, Peking University, Shenzhen 518055, People’s Republic of China.

2. Department of Psychiatry, University of Toronto, Toronto, ON, Canada.

3. Department of Neonatology, Shenzhen Children's Hospital, Shenzhen 518055, People’s Republic of China.

* Z.Q.Y and J.X.L contributed equally to this study.

#Corresponding author: E-mail: lisp@pku.edu.cn; szfjx2013@hotmail.com

***Supporting information***

**Table S1.** Targets of APN+AdipoR1+AdipoR2+IBD with APN relevance score ≥10

| **No.** | **Gene Symbol** | **Description** | **Relevance score** |
| --- | --- | --- | --- |
| **1** | **ADIPOQ** | Adiponectin, C1Q And Collagen Domain Containing | 149.4171448 |
| **2** | **ACADVL** | Acyl-CoA Dehydrogenase Very Long Chain | 96.89267731 |
| **3** | **CPT2** | Carnitine Palmitoyltransferase 2 | 91.03253174 |
| **4** | **ADA** | Adenosine Deaminase | 77.02828217 |
| **5** | **SLC2A1** | Solute Carrier Family 2 Member 1 | 74.02565002 |
| **6** | **DLD** | Dihydrolipoamide Dehydrogenase | 73.75358582 |
| **7** | **MTHFR** | Methylenetetrahydrofolate Reductase | 67.14596558 |
| **8** | **CPT1A** | Carnitine Palmitoyltransferase 1A | 63.20038605 |
| **9** | **LEP** | Leptin | 61.27810669 |
| **10** | **ARSA** | Arylsulfatase A | 54.54859161 |
| **11** | **IKBKB** | Inhibitor Of Nuclear Factor Kappa B Kinase Subunit Beta | 52.07706833 |
| **12** | **LPL** | Lipoprotein Lipase | 50.24864197 |
| **13** | **ABCA1** | ATP Binding Cassette Subfamily A Member 1 | 49.4683075 |
| **14** | **SLC25A20** | Solute Carrier Family 25 Member 20 | 48.52643967 |
| **15** | **IGF1** | Insulin Like Growth Factor 1 | 45.19636154 |
| **16** | **ACAT1** | Acetyl-CoA Acetyltransferase 1 | 44.83299637 |
| **17** | **ARG1** | Arginase 1 | 43.68304825 |
| **18** | **GH1** | Growth Hormone 1 | 43.52623749 |
| **19** | **PTS** | 6-Pyruvoyltetrahydropterin Synthase | 43.04131699 |
| **20** | **G6PC1** | Glucose-6-Phosphatase Catalytic Subunit 1 | 42.05064392 |
| **21** | **INS** | Insulin | 40.69327545 |
| **22** | **GYS1** | Glycogen Synthase 1 | 40.29492569 |
| **23** | **COX10** | Cytochrome C Oxidase Assembly Factor Heme A:Farnesyltransferase COX10 | 39.87785721 |
| **24** | **LIPA** | Lipase A, Lysosomal Acid Type | 38.51563263 |
| **25** | **APOA1** | Apolipoprotein A1 | 38.49344635 |
| **26** | **PLCG2** | Phospholipase C Gamma 2 | 37.55883789 |
| **27** | **NDUFS4** | NADH:Ubiquinone Oxidoreductase Subunit S4 | 37.16767883 |
| **28** | **LEPR** | Leptin Receptor | 36.00936127 |
| **29** | **LIPC** | Lipase C, Hepatic Type | 34.03387451 |
| **30** | **GYS2** | Glycogen Synthase 2 | 32.88444138 |
| **31** | **MAT2A** | Methionine Adenosyltransferase 2A | 31.37300491 |
| **32** | **POMC** | Proopiomelanocortin | 31.3509903 |
| **33** | **IGF1R** | Insulin Like Growth Factor 1 Receptor | 30.73971176 |
| **34** | **TNF** | Tumor Necrosis Factor | 30.53463173 |
| **35** | **CD36** | CD36 Molecule (CD36 Blood Group) | 30.22710609 |
| **36** | **MT-CO2** | Mitochondrially Encoded Cytochrome C Oxidase II | 30.15888596 |
| **37** | **APOB** | Apolipoprotein B | 29.87523651 |
| **38** | **GCK** | Glucokinase | 29.695755 |
| **39** | **PPARG** | Peroxisome Proliferator Activated Receptor Gamma | 29.21384048 |
| **40** | **ATM** | ATM Serine/Threonine Kinase | 29.18803024 |
| **41** | **TPI1** | Triosephosphate Isomerase 1 | 28.71281815 |
| **42** | **CYP19A1** | Cytochrome P450 Family 19 Subfamily A Member 1 | 28.55904579 |
| **43** | **INSR** | Insulin Receptor | 28.37015152 |
| **44** | **CYBB** | Cytochrome B-245 Beta Chain | 28.16389465 |
| **45** | **RETN** | Resistin | 27.40665054 |
| **46** | **TP53** | Tumor Protein P53 | 27.40094948 |
| **47** | **IL6** | Interleukin 6 | 26.8804512 |
| **48** | **SLC2A2** | Solute Carrier Family 2 Member 2 | 26.84040451 |
| **49** | **ALDOA** | Aldolase, Fructose-Bisphosphate A | 25.89560509 |
| **50** | **TTR** | Transthyretin | 24.87352562 |
| **51** | **ECHS1** | Enoyl-CoA Hydratase, Short Chain 1 | 24.81332779 |
| **52** | **CD40** | CD40 Molecule | 24.75733948 |
| **53** | **STAR** | Steroidogenic Acute Regulatory Protein | 24.63085556 |
| **54** | **PCK1** | Phosphoenolpyruvate Carboxykinase 1 | 24.57928085 |
| **55** | **APOC3** | Apolipoprotein C3 | 23.66195107 |
| **56** | **NDUFAF3** | NADH:Ubiquinone Oxidoreductase Complex Assembly Factor 3 | 23.36203957 |
| **57** | **PFKM** | Phosphofructokinase, Muscle | 23.03849792 |
| **58** | **COX5A** | Cytochrome C Oxidase Subunit 5A | 23.01070595 |
| **59** | **LDHA** | Lactate Dehydrogenase A | 22.89772606 |
| **60** | **NAGLU** | N-Acetyl-Alpha-Glucosaminidase | 22.84158325 |
| **61** | **APOC2** | Apolipoprotein C2 | 22.68746758 |
| **62** | **STAT3** | Signal Transducer And Activator Of Transcription 3 | 22.27862358 |
| **63** | **LDLR** | Low Density Lipoprotein Receptor | 21.83319855 |
| **64** | **TNFSF11** | TNF Superfamily Member 11 | 21.381073 |
| **65** | **ALPL** | Alkaline Phosphatase, Biomineralization Associated | 21.35381889 |
| **66** | **APOA5** | Apolipoprotein A5 | 21.28462982 |
| **67** | **KNG1** | Kininogen 1 | 21.10523033 |
| **68** | **APOE** | Apolipoprotein E | 21.07878685 |
| **69** | **GHRL** | Ghrelin And Obestatin Prepropeptide | 21.03521538 |
| **70** | **RBP4** | Retinol Binding Protein 4 | 20.95265961 |
| **71** | **ABCC8** | ATP Binding Cassette Subfamily C Member 8 | 20.35624313 |
| **72** | **XDH** | Xanthine Dehydrogenase | 20.35342979 |
| **73** | **PIK3CA** | Phosphatidylinositol-4,5-Bisphosphate 3-Kinase Catalytic Subunit Alpha | 20.19067383 |
| **74** | **FAS** | Fas Cell Surface Death Receptor | 19.95827675 |
| **75** | **ALB** | Albumin | 19.5243988 |
| **76** | **HMOX1** | Heme Oxygenase 1 | 19.40654564 |
| **77** | **DSP** | Desmoplakin | 19.31038666 |
| **78** | **TSHB** | Thyroid Stimulating Hormone Subunit Beta | 18.9286232 |
| **79** | **AMT** | Aminomethyltransferase | 18.82587814 |
| **80** | **TSC2** | TSC Complex Subunit 2 | 18.6607666 |
| **81** | **VLDLR** | Very Low Density Lipoprotein Receptor | 18.36997223 |
| **82** | **PIK3R1** | Phosphoinositide-3-Kinase Regulatory Subunit 1 | 18.32245636 |
| **83** | **VDR** | Vitamin D Receptor | 18.17501068 |
| **84** | **HNF4A** | Hepatocyte Nuclear Factor 4 Alpha | 18.07590103 |
| **85** | **MTOR** | Mechanistic Target Of Rapamycin Kinase | 18.06875038 |
| **86** | **SMAD5-AS1** | SMAD5 Antisense RNA 1 | 17.84828949 |
| **87** | **NFKB1** | Nuclear Factor Kappa B Subunit 1 | 17.66262245 |
| **88** | **AKT1** | AKT Serine/Threonine Kinase 1 | 17.6061554 |
| **89** | **ACACA** | Acetyl-CoA Carboxylase Alpha | 17.57531929 |
| **90** | **PIK3CD** | Phosphatidylinositol-4,5-Bisphosphate 3-Kinase Catalytic Subunit Delta | 17.37827301 |
| **91** | **COL3A1** | Collagen Type III Alpha 1 Chain | 17.30764198 |
| **92** | **PTEN** | Phosphatase And Tensin Homolog | 17.27806473 |
| **93** | **AR** | Androgen Receptor | 17.08813095 |
| **94** | **COX4I1** | Cytochrome C Oxidase Subunit 4I1 | 16.9908371 |
| **95** | **CREBBP** | CREB Binding Protein | 16.97622681 |
| **96** | **LEPQTL1** | Leptin, Serum Levels Of | 16.80129623 |
| **97** | **NFKBIA** | NFKB Inhibitor Alpha | 16.79353905 |
| **98** | **GPX1** | Glutathione Peroxidase 1 | 16.75167465 |
| **99** | **SERPINA6** | Serpin Family A Member 6 | 16.28858948 |
| **100** | **OCA2** | OCA2 Melanosomal Transmembrane Protein | 16.13784981 |
| **101** | **TXNRD2** | Thioredoxin Reductase 2 | 15.98853588 |
| **102** | **H19** | H19 Imprinted Maternally Expressed Transcript | 15.90372562 |
| **103** | **CYP2R1** | Cytochrome P450 Family 2 Subfamily R Member 1 | 15.87833405 |
| **104** | **IL1B** | Interleukin 1 Beta | 15.81327438 |
| **105** | **PRKAB1** | Protein Kinase AMP-Activated Non-Catalytic Subunit Beta 1 | 15.789958 |
| **106** | **BDNF-AS** | BDNF Antisense RNA | 15.36966228 |
| **107** | **PYY** | Peptide YY | 15.29372215 |
| **108** | **LHCGR** | Luteinizing Hormone/Choriogonadotropin Receptor | 15.23363972 |
| **109** | **UGT1A1** | UDP Glucuronosyltransferase Family 1 Member A1 | 15.21165657 |
| **110** | **PPARGC1A** | PPARG Coactivator 1 Alpha | 15.0242424 |
| **111** | **AGPAT2** | 1-Acylglycerol-3-Phosphate O-Acyltransferase 2 | 14.91242409 |
| **112** | **GPT** | Glutamic--Pyruvic Transaminase | 14.8905735 |
| **113** | **LPIN1** | Lipin 1 | 14.70601845 |
| **114** | **GGT1** | Gamma-Glutamyltransferase 1 | 14.62324429 |
| **115** | **PNLIP** | Pancreatic Lipase | 14.56826973 |
| **116** | **NAMPT** | Nicotinamide Phosphoribosyltransferase | 14.55557728 |
| **117** | **PNPLA2** | Patatin Like Phospholipase Domain Containing 2 | 14.51128387 |
| **118** | **HSD11B1** | Hydroxysteroid 11-Beta Dehydrogenase 1 | 14.45156574 |
| **119** | **CCL2** | C-C Motif Chemokine Ligand 2 | 14.30059433 |
| **120** | **SFTA3** | Surfactant Associated 3 | 14.11212349 |
| **121** | **PPARA** | Peroxisome Proliferator Activated Receptor Alpha | 13.97862434 |
| **122** | **MMP9** | Matrix Metallopeptidase 9 | 13.83238602 |
| **123** | **NR3C1** | Nuclear Receptor Subfamily 3 Group C Member 1 | 13.82389927 |
| **124** | **NOS3** | Nitric Oxide Synthase 3 | 13.81096935 |
| **125** | **CERNA3** | Competing Endogenous LncRNA 3 For MiR-645 | 13.56863594 |
| **126** | **SIRT1** | Sirtuin 1 | 13.56625175 |
| **127** | **SOD2-OT1** | SOD2 Overlapping Transcript 1 | 13.43388176 |
| **128** | **CXCL8** | C-X-C Motif Chemokine Ligand 8 | 13.33070278 |
| **129** | **COL1A1** | Collagen Type I Alpha 1 Chain | 13.323699 |
| **130** | **IRS1** | Insulin Receptor Substrate 1 | 13.13834286 |
| **131** | **PRL** | Prolactin | 13.13256836 |
| **132** | **TCTN2** | Tectonic Family Member 2 | 12.82382774 |
| **133** | **CYP1B1** | Cytochrome P450 Family 1 Subfamily B Member 1 | 12.71220112 |
| **134** | **EP300** | E1A Binding Protein P300 | 12.68213367 |
| **135** | **PCK2** | Phosphoenolpyruvate Carboxykinase 2, Mitochondrial | 12.45251942 |
| **136** | **TLR4** | Toll Like Receptor 4 | 12.42149067 |
| **137** | **STK11** | Serine/Threonine Kinase 11 | 12.41952991 |
| **138** | **PKM** | Pyruvate Kinase M1/2 | 12.28809357 |
| **139** | **CDH13** | Cadherin 13 | 12.00189972 |
| **140** | **ESR1** | Estrogen Receptor 1 | 11.74667549 |
| **141** | **LINC02605** | Long Intergenic Non-Protein Coding RNA 2605 | 11.60106468 |
| **142** | **CSNK2A1** | Casein Kinase 2 Alpha 1 | 11.55945778 |
| **143** | **ABCB7** | ATP Binding Cassette Subfamily B Member 7 | 11.47914982 |
| **144** | **GAPDH** | Glyceraldehyde-3-Phosphate Dehydrogenase | 11.44672203 |
| **145** | **GC** | GC Vitamin D Binding Protein | 11.22716427 |
| **146** | **GJA1** | Gap Junction Protein Alpha 1 | 11.21975327 |
| **147** | **KCNJ11** | Potassium Inwardly Rectifying Channel Subfamily J Member 11 | 11.10448074 |
| **148** | **JAK2** | Janus Kinase 2 | 11.04750633 |
| **149** | **DGAT1** | Diacylglycerol O-Acyltransferase 1 | 11.02456665 |
| **150** | **AOX1** | Aldehyde Oxidase 1 | 10.93191242 |
| **151** | **COMT** | Catechol-O-Methyltransferase | 10.81887722 |
| **152** | **UCP2** | Uncoupling Protein 2 | 10.66944885 |
| **153** | **PON1** | Paraoxonase 1 | 10.60445213 |
| **154** | **LIPE** | Lipase E, Hormone Sensitive Type | 10.55718994 |
| **155** | **ENPP1** | Ectonucleotide Pyrophosphatase/Phosphodiesterase 1 | 10.50633335 |
| **156** | **MAPK1** | Mitogen-Activated Protein Kinase 1 | 10.18360043 |
| **157** | **GMPPB** | GDP-Mannose Pyrophosphorylase B | 10.16575336 |
| **158** | **SELE** | Selectin E | 10.10769749 |
| **159** | **CKM** | Creatine Kinase, M-Type | 10.06750107 |

Available (November 2024): https://www.genecards.org/Search

**Table S2.** Targets of APN+AdipoR1+IBD with APN relevance score ≥10, except APN+AdipoR1+AdipoR2+IBD

| **No.** | **Gene Symbol** | **Description** | **Relevance score** |
| --- | --- | --- | --- |
| **1** | **IL2RA** | Interleukin 2 Receptor Subunit Alpha | 38.22231674 |
| **2** | **CRP** | C-Reactive Protein | 28.32578087 |
| **3** | **NLRP3** | NLR Family Pyrin Domain Containing 3 | 16.74045563 |
| **4** | **CXCR4** | C-X-C Motif Chemokine Receptor 4 | 16.13383293 |
| **5** | **NOD2** | Nucleotide Binding Oligomerization Domain Containing 2 | 15.06386757 |
| **6** | **TNFRSF11B** | TNF Receptor Superfamily Member 11b | 14.31340408 |
| **7** | **RUNX1** | RUNX Family Transcription Factor 1 | 13.18826866 |
| **8** | **WFS1** | Wolframin ER Transmembrane Glycoprotein | 12.76303864 |
| **9** | **CTNNB1** | Catenin Beta 1 | 12.62774658 |
| **10** | **BMP2** | Bone Morphogenetic Protein 2 | 10.8545351 |
| **11** | **FTO** | FTO Alpha-Ketoglutarate Dependent Dioxygenase | 10.1345787 |

Available (November 2024): https://www.genecards.org/Search

**Table S3.** Targets of APN+AdipoR2+IBD with APN relevance score ≥10, except APN+AdipoR1+AdipoR2+IBD

| **No.** | **Gene Symbol** | **Description** | **Relevance score** |
| --- | --- | --- | --- |
| **1** | **JAK3** | Janus Kinase 3 | 66.93528748 |
| **2** | **LCAT** | Lecithin-Cholesterol Acyltransferase | 36.63467789 |
| **3** | **VWF** | Von Willebrand Factor | 24.57114792 |
| **4** | **SLC17A5** | Solute Carrier Family 17 Member 5 | 24.19079781 |
| **5** | **LAMP2** | Lysosomal Associated Membrane Protein 2 | 23.16798019 |
| **6** | **FBN1** | Fibrillin 1 | 19.23112297 |
| **7** | **TGFB1** | Transforming Growth Factor Beta 1 | 19.12390709 |
| **8** | **ABCG5** | ATP Binding Cassette Subfamily G Member 5 | 18.88196945 |
| **9** | **SERPINF1** | Serpin Family F Member 1 | 18.5030632 |
| **10** | **MTTP** | Microsomal Triglyceride Transfer Protein | 18.08280373 |
| **11** | **TNNI3** | Troponin I3, Cardiac Type | 15.64596844 |
| **12** | **SGCB** | Sarcoglycan Beta | 15.59379578 |
| **13** | **CETP** | Cholesteryl Ester Transfer Protein | 15.16499805 |
| **14** | **SCARB2** | Scavenger Receptor Class B Member 2 | 15.11997032 |
| **15** | **MC4R** | Melanocortin 4 Receptor | 14.25977325 |
| **16** | **MIPEP** | Mitochondrial Intermediate Peptidase | 13.4819088 |
| **17** | **SDR9C7** | Short Chain Dehydrogenase/Reductase Family 9C Member 7 | 13.11808681 |
| **18** | **FLCN** | Folliculin | 11.9012146 |
| **19** | **ACE** | Angiotensin I Converting Enzyme | 11.69754982 |
| **20** | **MMP13** | Matrix Metallopeptidase 13 | 10.66612053 |
| **21** | **CRH** | Corticotropin Releasing Hormone | 10.640172 |
| **22** | **CASP3** | Caspase 3 | 10.52865219 |
| **23** | **IL18** | Interleukin 18 | 10.50745678 |
| **24** | **ANGPT1** | Angiopoietin 1 | 10.36938953 |

Available (November 2024): https://www.genecards.org/Search

**Table S4.** String node degrees of APN+AdipoR1+AdipoR2+IBD (Homo sapiens)

| **No.** | **Node** | **Identifier** | **Degree** | **No.** | **Node** | **Identifier** | **Degree** |
| --- | --- | --- | --- | --- | --- | --- | --- |
| **1** | **INS** | 9606.ENSP00000380432 | 108 | **76** | **LIPC** | 9606.ENSP00000299022 | 30 |
| **2** | **GAPDH** | 9606.ENSP00000380070 | 99 | **77** | **PIK3CA** | 9606.ENSP00000263967 | 30 |
| **3** | **AKT1** | 9606.ENSP00000451828 | 93 | **78** | **PON1** | 9606.ENSP00000222381 | 30 |
| **4** | **PPARG** | 9606.ENSP00000287820 | 93 | **79** | **XDH** | 9606.ENSP00000368727 | 30 |
| **5** | **ALB** | 9606.ENSP00000295897 | 90 | **80** | **TTR** | 9606.ENSP00000237014 | 29 |
| **6** | **IL6** | 9606.ENSP00000385675 | 86 | **81** | **KNG1** | 9606.ENSP00000493985 | 28 |
| **7** | **TNF** | 9606.ENSP00000398698 | 85 | **82** | **COL1A1** | 9606.ENSP00000225964 | 26 |
| **8** | **PPARA** | 9606.ENSP00000385523 | 79 | **83** | **TSC2** | 9606.ENSP00000219476 | 26 |
| **9** | **LEP** | 9606.ENSP00000312652 | 78 | **84** | **LPIN1** | 9606.ENSP00000397908 | 24 |
| **10** | **TP53** | 9606.ENSP00000269305 | 78 | **85** | **TPI1** | 9606.ENSP00000229270 | 23 |
| **11** | **IL1B** | 9606.ENSP00000263341 | 76 | **86** | **CPT2** | 9606.ENSP00000360541 | 22 |
| **12** | **ADIPOQ** | 9606.ENSP00000389814 | 73 | **87** | **GYS1** | 9606.ENSP00000317904 | 21 |
| **13** | **SIRT1** | 9606.ENSP00000212015 | 72 | **88** | **ACADVL** | 9606.ENSP00000438689 | 20 |
| **14** | **PPARGC1A** | 9606.ENSP00000264867 | 71 | **89** | **COX4I1** | 9606.ENSP00000457513 | 20 |
| **15** | **IGF1** | 9606.ENSP00000376637 | 69 | **90** | **CYP27B1** | 9606.ENSP00000228606 | 20 |
| **16** | **STAT3** | 9606.ENSP00000264657 | 67 | **91** | **PFKM** | 9606.ENSP00000496597 | 20 |
| **17** | **APOE** | 9606.ENSP00000252486 | 66 | **92** | **RBP4** | 9606.ENSP00000360522 | 20 |
| **18** | **CD36** | 9606.ENSP00000415743 | 66 | **93** | **HSD11B1** | 9606.ENSP00000355995 | 19 |
| **19** | **ESR1** | 9606.ENSP00000405330 | 66 | **94** | **MTHFR** | 9606.ENSP00000365770 | 19 |
| **20** | **GPT** | 9606.ENSP00000378408 | 64 | **95** | **VLDLR** | 9606.ENSP00000371532 | 19 |
| **21** | **IRS1** | 9606.ENSP00000304895 | 64 | **96** | **GC** | 9606.ENSP00000421725 | 18 |
| **22** | **MTOR** | 9606.ENSP00000354558 | 62 | **97** | **PNLIP** | 9606.ENSP00000358223 | 18 |
| **23** | **NFKB1** | 9606.ENSP00000226574 | 62 | **98** | **ALDOA** | 9606.ENSP00000496166 | 17 |
| **24** | **APOB** | 9606.ENSP00000233242 | 58 | **99** | **APOC2** | 9606.ENSP00000466775 | 17 |
| **25** | **CCL2** | 9606.ENSP00000225831 | 56 | **100** | **GJA1** | 9606.ENSP00000282561 | 17 |
| **26** | **TLR4** | 9606.ENSP00000363089 | 55 | **101** | **PIK3CD** | 9606.ENSP00000366563 | 17 |
| **27** | **CXCL8** | 9606.ENSP00000306512 | 53 | **102** | **APOA5** | 9606.ENSP00000445002 | 16 |
| **28** | **PTEN** | 9606.ENSP00000361021 | 53 | **103** | **FAS** | 9606.ENSP00000498466 | 16 |
| **29** | **LPL** | 9606.ENSP00000497642 | 51 | **104** | **PLCG2** | 9606.ENSP00000482457 | 16 |
| **30** | **MMP9** | 9606.ENSP00000361405 | 51 | **105** | **PYY** | 9606.ENSP00000353198 | 16 |
| **31** | **JAK2** | 9606.ENSP00000371067 | 49 | **106** | **TXNRD2** | 9606.ENSP00000383365 | 16 |
| **32** | **POMC** | 9606.ENSP00000384092 | 49 | **107** | **GYS2** | 9606.ENSP00000261195 | 15 |
| **33** | **CPT1A** | 9606.ENSP00000265641 | 48 | **108** | **LHCGR** | 9606.ENSP00000294954 | 15 |
| **34** | **LIPE** | 9606.ENSP00000244289 | 48 | **109** | **DLD** | 9606.ENSP00000205402 | 14 |
| **35** | **ACACA** | 9606.ENSP00000483300 | 46 | **110** | **ENPP1** | 9606.ENSP00000498074 | 14 |
| **36** | **APOA1** | 9606.ENSP00000236850 | 46 | **111** | **GH1** | 9606.ENSP00000312673 | 14 |
| **37** | **G6PC** | 9606.ENSP00000253801 | 46 | **112** | **COMT** | 9606.ENSP00000354511 | 13 |
| **38** | **SLC2A1** | 9606.ENSP00000416293 | 46 | **113** | **ECHS1** | 9606.ENSP00000357535 | 13 |
| **39** | **SLC2A2** | 9606.ENSP00000323568 | 46 | **114** | **UGT1A1** | 9606.ENSP00000304845 | 13 |
| **40** | **EP300** | 9606.ENSP00000263253 | 45 | **115** | **AGPAT2** | 9606.ENSP00000360761 | 12 |
| **41** | **NOS3** | 9606.ENSP00000297494 | 45 | **116** | **ALPL** | 9606.ENSP00000363973 | 12 |
| **42** | **RETN** | 9606.ENSP00000221515 | 44 | **117** | **COL3A1** | 9606.ENSP00000304408 | 12 |
| **43** | **HMOX1** | 9606.ENSP00000216117 | 43 | **118** | **CSNK2A1** | 9606.ENSP00000217244 | 12 |
| **44** | **PCK1** | 9606.ENSP00000319814 | 43 | **119** | **LIPA** | 9606.ENSP00000337354 | 12 |
| **45** | **UCP2** | 9606.ENSP00000499695 | 43 | **120** | **ABCC8** | 9606.ENSP00000494321 | 11 |
| **46** | **ABCA1** | 9606.ENSP00000363868 | 42 | **121** | **SERPINA6** | 9606.ENSP00000342850 | 11 |
| **47** | **IGF1R** | 9606.ENSP00000497069 | 42 | **122** | **STAR** | 9606.ENSP00000276449 | 11 |
| **48** | **NFKBIA** | 9606.ENSP00000216797 | 42 | **123** | **CYP1B1** | 9606.ENSP00000478561 | 10 |
| **49** | **NR3C1** | 9606.ENSP00000231509 | 42 | **124** | **KCNJ11** | 9606.ENSP00000345708 | 10 |
| **50** | **HNF4A** | 9606.ENSP00000312987 | 41 | **125** | **LDLR** | 9606.ENSP00000454071 | 10 |
| **51** | **INSR** | 9606.ENSP00000303830 | 41 | **126** | **PRKAB1** | 9606.ENSP00000441369 | 10 |
| **52** | **LEPR** | 9606.ENSP00000330393 | 41 | **127** | **ADA** | 9606.ENSP00000361965 | 9 |
| **53** | **CREBBP** | 9606.ENSP00000262367 | 40 | **128** | **COX5A** | 9606.ENSP00000317780 | 9 |
| **54** | **PKM** | 9606.ENSP00000320171 | 39 | **129** | **MAT2A** | 9606.ENSP00000303147 | 9 |
| **55** | **MAPK1** | 9606.ENSP00000215832 | 38 | **130** | **MT-CO2** | 9606.ENSP00000354876 | 9 |
| **56** | **GCK** | 9606.ENSP00000223366 | 37 | **131** | **ACAT1** | 9606.ENSP00000265838 | 8 |
| **57** | **LDHA** | 9606.ENSP00000445175 | 37 | **132** | **NDUFS4** | 9606.ENSP00000296684 | 8 |
| **58** | **PIK3R1** | 9606.ENSP00000428056 | 37 | **133** | **SLC25A20** | 9606.ENSP00000326305 | 8 |
| **59** | **CYBB** | 9606.ENSP00000367851 | 36 | **134** | **PTS** | 9606.ENSP00000280362 | 7 |
| **60** | **PNPLA2** | 9606.ENSP00000337701 | 36 | **135** | **AMT** | 9606.ENSP00000273588 | 6 |
| **61** | **PCK2** | 9606.ENSP00000216780 | 34 | **136** | **COX10** | 9606.ENSP00000261643 | 6 |
| **62** | **AR** | 9606.ENSP00000363822 | 33 | **137** | **TSHB** | 9606.ENSP00000256592 | 6 |
| **63** | **ARG1** | 9606.ENSP00000349446 | 33 | **138** | **CDH13** | 9606.ENSP00000268613 | 5 |
| **64** | **ATM** | 9606.ENSP00000278616 | 33 | **139** | **CKM** | 9606.ENSP00000221476 | 5 |
| **65** | **NAMPT** | 9606.ENSP00000222553 | 33 | **140** | **GGT1** | 9606.ENSP00000248923 | 4 |
| **66** | **STK11** | 9606.ENSP00000324856 | 33 | **141** | **OCA2** | 9606.ENSP00000346659 | 4 |
| **67** | **APOC3** | 9606.ENSP00000227667 | 32 | **142** | **DSP** | 9606.ENSP00000369129 | 3 |
| **68** | **IKBKB** | 9606.ENSP00000430684 | 32 | **143** | **ABCB7** | 9606.ENSP00000253577 | 2 |
| **69** | **PRL** | 9606.ENSP00000302150 | 32 | **144** | **AOX1** | 9606.ENSP00000363832 | 2 |
| **70** | **SELE** | 9606.ENSP00000331736 | 32 | **145** | **CYP2R1** | 9606.ENSP00000334592 | 2 |
| **71** | **DGAT1** | 9606.ENSP00000482264 | 31 | **146** | **GMPPB** | 9606.ENSP00000309092 | 2 |
| **72** | **GHRL** | 9606.ENSP00000335074 | 31 | **147** | **NAGLU** | 9606.ENSP00000225927 | 2 |
| **73** | **TNFSF11** | 9606.ENSP00000381775 | 31 | **148** | **NDUFAF3** | 9606.ENSP00000323076 | 2 |
| **74** | **CD40** | 9606.ENSP00000361359 | 30 | **149** | **ARSA** | 9606.ENSP00000216124 | 1 |
| **75** | **CYP19A1** | 9606.ENSP00000379683 | 30 | **150** | **CTCFL** | 9606.ENSP00000415579 | 1 |

Minimum required interaction score: medium confidence 0.4. The nodes with degree>0 are shown in the table.

Available (November 2024):

https://cn.string-db.org/cgi/input?sessionId=bBSQf0gz7Y6u&input_page_show_search=on

**Table S5.** String node degrees of APN+AdipoR1+AdipoR2+IBD (Mouse musculus)

| **No.** | **Node** | **Identifier** | **Degree** | **No.** | **Node** | **Identifier** | **Degree** |
| --- | --- | --- | --- | --- | --- | --- | --- |
| **1** | **Gapdh** | 10090.ENSMUSP00000113942 | 89 | **75** | **Cox4i1** | 10090.ENSMUSP00000138019 | 23 |
| **2** | **Akt1** | 10090.ENSMUSP00000001780 | 88 | **76** | **Lipc** | 10090.ENSMUSP00000034731 | 23 |
| **3** | **Alb** | 10090.ENSMUSP00000031314 | 88 | **77** | **Lpin1** | 10090.ENSMUSP00000070583 | 23 |
| **4** | **Tnf** | 10090.ENSMUSP00000025263 | 88 | **78** | **Ep300** | 10090.ENSMUSP00000066789 | 22 |
| **5** | **Il6** | 10090.ENSMUSP00000026845 | 83 | **79** | **Col1a1** | 10090.ENSMUSP00000001547 | 21 |
| **6** | **Pparg** | 10090.ENSMUSP00000000450 | 83 | **80** | **Hsd11b1** | 10090.ENSMUSP00000016338 | 20 |
| **7** | **Ppara** | 10090.ENSMUSP00000105049 | 79 | **81** | **Acadvl** | 10090.ENSMUSP00000099634 | 19 |
| **8** | **Ppargc1a** | 10090.ENSMUSP00000117040 | 72 | **82** | **Cpt2** | 10090.ENSMUSP00000030345 | 18 |
| **9** | **Trp53** | 10090.ENSMUSP00000104298 | 71 | **83** | **Nos3** | 10090.ENSMUSP00000030834 | 18 |
| **10** | **Sirt1** | 10090.ENSMUSP00000112595 | 68 | **84** | **Tpi1** | 10090.ENSMUSP00000130858 | 18 |
| **11** | **Adipoq** | 10090.ENSMUSP00000023593 | 67 | **85** | **Ttr** | 10090.ENSMUSP00000074783 | 18 |
| **12** | **Il1b** | 10090.ENSMUSP00000028881 | 67 | **86** | **Apoa5** | 10090.ENSMUSP00000113413 | 17 |
| **13** | **Lep** | 10090.ENSMUSP00000067046 | 67 | **87** | **Csnk2a1** | 10090.ENSMUSP00000096829 | 17 |
| **14** | **Stat3** | 10090.ENSMUSP00000120152 | 66 | **88** | **Pik3cd** | 10090.ENSMUSP00000101315 | 17 |
| **15** | **Igf1** | 10090.ENSMUSP00000100937 | 62 | **89** | **Rbp4** | 10090.ENSMUSP00000025951 | 17 |
| **16** | **Cd36** | 10090.ENSMUSP00000133008 | 60 | **90** | **Slc2a1** | 10090.ENSMUSP00000030398 | 17 |
| **17** | **Apoe** | 10090.ENSMUSP00000133302 | 59 | **91** | **Apoc2** | 10090.ENSMUSP00000115173 | 16 |
| **18** | **Esr1** | 10090.ENSMUSP00000101215 | 59 | **92** | **Ugt1a1** | 10090.ENSMUSP00000072803 | 16 |
| **19** | **Mtor** | 10090.ENSMUSP00000099510 | 58 | **93** | **Gja1** | 10090.ENSMUSP00000151620 | 15 |
| **20** | **Irs1** | 10090.ENSMUSP00000063795 | 54 | **94** | **Gc** | 10090.ENSMUSP00000046636 | 14 |
| **21** | **Apob** | 10090.ENSMUSP00000036044 | 53 | **95** | **Plcg2** | 10090.ENSMUSP00000079991 | 14 |
| **22** | **Tlr4** | 10090.ENSMUSP00000045770 | 53 | **96** | **Pnlip** | 10090.ENSMUSP00000056377 | 14 |
| **23** | **Ccl2** | 10090.ENSMUSP00000000193 | 51 | **97** | **Vldlr** | 10090.ENSMUSP00000127329 | 14 |
| **24** | **Pten** | 10090.ENSMUSP00000013807 | 50 | **98** | **Agpat2** | 10090.ENSMUSP00000028286 | 13 |
| **25** | **Jak2** | 10090.ENSMUSP00000064394 | 48 | **99** | **Echs1** | 10090.ENSMUSP00000026538 | 13 |
| **26** | **Mmp9** | 10090.ENSMUSP00000017881 | 48 | **100** | **Fas** | 10090.ENSMUSP00000025691 | 13 |
| **27** | **Gpx1** | 10090.ENSMUSP00000081010 | 45 | **101** | **Gys1** | 10090.ENSMUSP00000003964 | 13 |
| **28** | **Pck1** | 10090.ENSMUSP00000029017 | 44 | **102** | **Pck2** | 10090.ENSMUSP00000038555 | 13 |
| **29** | **Pomc** | 10090.ENSMUSP00000151504 | 44 | **103** | **Pyy** | 10090.ENSMUSP00000135292 | 13 |
| **30** | **Lpl** | 10090.ENSMUSP00000015712 | 43 | **104** | **Aldoa** | 10090.ENSMUSP00000084846 | 12 |
| **31** | **Cpt1a** | 10090.ENSMUSP00000025835 | 42 | **105** | **Col3a1** | 10090.ENSMUSP00000085192 | 12 |
| **32** | **Hmox1** | 10090.ENSMUSP00000005548 | 41 | **106** | **Comt** | 10090.ENSMUSP00000130077 | 12 |
| **33** | **Lipe** | 10090.ENSMUSP00000003207 | 41 | **107** | **Dld** | 10090.ENSMUSP00000106481 | 12 |
| **34** | **Nfkbia** | 10090.ENSMUSP00000021413 | 40 | **108** | **Gys2** | 10090.ENSMUSP00000032371 | 12 |
| **35** | **Acaca** | 10090.ENSMUSP00000099490 | 39 | **109** | **Star** | 10090.ENSMUSP00000033979 | 12 |
| **36** | **Apoa1** | 10090.ENSMUSP00000034588 | 39 | **110** | **Cox5a** | 10090.ENSMUSP00000000090 | 11 |
| **37** | **Slc2a2** | 10090.ENSMUSP00000029240 | 39 | **111** | **Cyp1b1** | 10090.ENSMUSP00000024894 | 11 |
| **38** | **Abca1** | 10090.ENSMUSP00000030010 | 38 | **112** | **Gh** | 10090.ENSMUSP00000099360 | 11 |
| **39** | **Lepr** | 10090.ENSMUSP00000037385 | 37 | **113** | **Lhcgr** | 10090.ENSMUSP00000024916 | 11 |
| **40** | **Cybb** | 10090.ENSMUSP00000015484 | 35 | **114** | **Pfkm** | 10090.ENSMUSP00000059801 | 11 |
| **41** | **Gck** | 10090.ENSMUSP00000099984 | 35 | **115** | **Kcnj11** | 10090.ENSMUSP00000147439 | 10 |
| **42** | **Retn** | 10090.ENSMUSP00000133024 | 35 | **116** | **Ldlr** | 10090.ENSMUSP00000034713 | 10 |
| **43** | **Hnf4a** | 10090.ENSMUSP00000018094 | 34 | **117** | **Mthfr** | 10090.ENSMUSP00000069774 | 9 |
| **44** | **Insr** | 10090.ENSMUSP00000088837 | 34 | **118** | **Prkab1** | 10090.ENSMUSP00000031486 | 9 |
| **45** | **Nr3c1** | 10090.ENSMUSP00000111229 | 34 | **119** | **Serpina6** | 10090.ENSMUSP00000044033 | 9 |
| **46** | **Ghrl** | 10090.ENSMUSP00000145281 | 33 | **120** | **Enpp1** | 10090.ENSMUSP00000114273 | 8 |
| **47** | **Igf1r** | 10090.ENSMUSP00000005671 | 33 | **121** | **Ndufs4** | 10090.ENSMUSP00000022286 | 8 |
| **48** | **Atm** | 10090.ENSMUSP00000156344 | 32 | **122** | **Slc25a20** | 10090.ENSMUSP00000035222 | 8 |
| **49** | **Ldha** | 10090.ENSMUSP00000148107 | 32 | **123** | **Vdr** | 10090.ENSMUSP00000023119 | 8 |
| **50** | **Ucp2** | 10090.ENSMUSP00000120967 | 32 | **124** | **mt-Co2** | 10090.ENSMUSP00000080994 | 8 |
| **51** | **Ikbkb** | 10090.ENSMUSP00000033939 | 31 | **125** | **Alpl** | 10090.ENSMUSP00000030551 | 7 |
| **52** | **Nfkb1** | 10090.ENSMUSP00000029812 | 31 | **126** | **Cox10** | 10090.ENSMUSP00000040138 | 7 |
| **53** | **Pnpla2** | 10090.ENSMUSP00000127149 | 31 | **127** | **Acat1** | 10090.ENSMUSP00000034547 | 6 |
| **54** | **Nampt** | 10090.ENSMUSP00000020886 | 30 | **128** | **Ada** | 10090.ENSMUSP00000017841 | 6 |
| **55** | **Pkm** | 10090.ENSMUSP00000034834 | 30 | **129** | **Abcc8** | 10090.ENSMUSP00000033123 | 5 |
| **56** | **Apoc3** | 10090.ENSMUSP00000113126 | 29 | **130** | **Oca2** | 10090.ENSMUSP00000032633 | 5 |
| **57** | **Arg1** | 10090.ENSMUSP00000020161 | 29 | **131** | **Pts** | 10090.ENSMUSP00000034570 | 5 |
| **58** | **Crebbp** | 10090.ENSMUSP00000023165 | 29 | **132** | **Aox1** | 10090.ENSMUSP00000001027 | 4 |
| **59** | **Cyp19a1** | 10090.ENSMUSP00000034811 | 29 | **133** | **Mat2a** | 10090.ENSMUSP00000087118 | 4 |
| **60** | **Pik3ca** | 10090.ENSMUSP00000029201 | 29 | **134** | **Nckap1** | 10090.ENSMUSP00000107390 | 4 |
| **61** | **Pik3r1** | 10090.ENSMUSP00000056774 | 29 | **135** | **Tshb** | 10090.ENSMUSP00000029450 | 4 |
| **62** | **Tnfsf11** | 10090.ENSMUSP00000022592 | 29 | **136** | **Txnrd2** | 10090.ENSMUSP00000146030 | 4 |
| **63** | **Xdh** | 10090.ENSMUSP00000024866 | 29 | **137** | **Abcb7** | 10090.ENSMUSP00000033695 | 3 |
| **64** | **Dgat1** | 10090.ENSMUSP00000023214 | 28 | **138** | **Amt** | 10090.ENSMUSP00000035230 | 3 |
| **65** | **Cd40** | 10090.ENSMUSP00000017799 | 27 | **139** | **Ckm** | 10090.ENSMUSP00000146972 | 3 |
| **66** | **Pon1** | 10090.ENSMUSP00000002663 | 27 | **140** | **Cyp2r1** | 10090.ENSMUSP00000032908 | 3 |
| **67** | **Ar** | 10090.ENSMUSP00000052648 | 26 | **141** | **Naglu** | 10090.ENSMUSP00000001802 | 3 |
| **68** | **Kng1** | 10090.ENSMUSP00000023589 | 26 | **142** | **Cdh13** | 10090.ENSMUSP00000113527 | 2 |
| **69** | **Mapk1** | 10090.ENSMUSP00000065983 | 26 | **143** | **Dsp** | 10090.ENSMUSP00000115062 | 2 |
| **70** | **Tsc2** | 10090.ENSMUSP00000094986 | 26 | **144** | **Ggt1** | 10090.ENSMUSP00000006508 | 2 |
| **71** | **Gpt** | 10090.ENSMUSP00000023203 | 25 | **145** | **Lipa** | 10090.ENSMUSP00000053270 | 2 |
| **72** | **Sele** | 10090.ENSMUSP00000027874 | 25 | **146** | **Ndufaf3** | 10090.ENSMUSP00000073832 | 2 |
| **73** | **Prl** | 10090.ENSMUSP00000105998 | 24 | **147** | **Arsa** | 10090.ENSMUSP00000127646 | 1 |
| **74** | **Stk11** | 10090.ENSMUSP00000003152 | 24 | **148** | **Itpk1** | 10090.ENSMUSP00000046027 | 1 |

Minimum required interaction score: medium confidence 0.4. The nodes with degree>0 are shown in the table. Available (November 2024):

https://cn.string-db.org/cgi/input?sessionId=bBSQf0gz7Y6u&input_page_show_search=on

**Table S6.** String node degrees of APN+AdipoR1+IBD with APN relevance score ≥10, except APN+AdipoR1+AdipoR2+IBD (Homo sapiens)

| **No.** | **Node** | **Identifier** | **Degree** | **No.** | **Node** | **Identifier** | **Degree** |
| --- | --- | --- | --- | --- | --- | --- | --- |
| **1** | **IL6** | 9606.ENSP00000385675 | 13 | **9** | **BMP2** | 9606.ENSP00000368104 | 6 |
| **2** | **CXCL10** | 9606.ENSP00000305651 | 9 | **10** | **NLRP3** | 9606.ENSP00000337383 | 6 |
| **3** | **FOXP3** | 9606.ENSP00000365380 | 9 | **11** | **RUNX1** | 9606.ENSP00000300305 | 6 |
| **4** | **CTNNB1** | 9606.ENSP00000495360 | 8 | **12** | **DEFB4B** | 9606.ENSP00000424598 | 5 |
| **5** | **CXCR4** | 9606.ENSP00000386884 | 8 | **13** | **NOD2** | 9606.ENSP00000300589 | 5 |
| **6** | **CRP** | 9606.ENSP00000255030 | 7 | **14** | **TNFRSF11B** | 9606.ENSP00000297350 | 3 |
| **7** | **IL2RA** | 9606.ENSP00000369293 | 7 | **15** | **FTO** | 9606.ENSP00000418823 | 1 |
| **8** | **BGLAP** | 9606.ENSP00000357255 | 6 | **16** | **WFS1** | 9606.ENSP00000226760 | 1 |

Minimum required interaction score: medium confidence 0.4. The nodes with degree>0 are shown in the table.

Available (November 2024):

https://cn.string-db.org/cgi/input?sessionId=bBSQf0gz7Y6u&input_page_show_search=on

**Table S7.** String node degrees of APN+AdipoR1+IBD with APN relevance score ≥10, except APN+AdipoR1+AdipoR2+IBD (Mouse musculus)

| **No.** | **Node** | **Identifier** | **Degree** | **No.** | **Node** | **Identifier** | **Degree** |
| --- | --- | --- | --- | --- | --- | --- | --- |
| **1** | **Ctnnb1** | 10090.ENSMUSP00000007130 | 9 | **9** | **Nlrp3** | 10090.ENSMUSP00000098707 | 5 |
| **2** | **Csf2** | 10090.ENSMUSP00000019060 | 8 | **10** | **Nod2** | 10090.ENSMUSP00000113773 | 4 |
| **3** | **Cxcl15** | 10090.ENSMUSP00000031322 | 7 | **11** | **Runx1** | 10090.ENSMUSP00000023673 | 4 |
| **4** | **Crp** | 10090.ENSMUSP00000044665 | 6 | **12** | **Bmp2** | 10090.ENSMUSP00000028836 | 3 |
| **5** | **Il2ra** | 10090.ENSMUSP00000028111 | 6 | **13** | **Tnfrsf11b** | 10090.ENSMUSP00000078705 | 3 |
| **6** | **Bglap2** | 10090.ENSMUSP00000096555 | 5 | **14** | **Nlrp1b** | 10090.ENSMUSP00000104155 | 2 |
| **7** | **Bglap3** | 10090.ENSMUSP00000103166 | 5 | **15** | **Fto** | 10090.ENSMUSP00000068380 | 1 |
| **8** | **Cxcr4** | 10090.ENSMUSP00000053489 | 5 | **16** | **Wfs1** | 10090.ENSMUSP00000048053 | 1 |

Minimum required interaction score: medium confidence 0.4. The nodes with degree>0 are shown in the table.

Available (November 2024):

https://cn.string-db.org/cgi/input?sessionId=bBSQf0gz7Y6u&input_page_show_search=on

**Table S8.** String node degrees of APN+AdipoR2+IBD with APN relevance score ≥10, except APN+AdipoR1+AdipoR2+IBD (Homo sapiens)

| **No.** | **Node** | **Identifier** | **Degree** | **No.** | **Node** | **Identifier** | **Degree** |
| --- | --- | --- | --- | --- | --- | --- | --- |
| **1** | **APOE** | 9606.ENSP00000252486 | 16 | **13** | **ABCG5** | 9606.ENSP00000384513 | 7 |
| **2** | **CRP** | 9606.ENSP00000255030 | 13 | **14** | **MTTP** | 9606.ENSP00000427679 | 7 |
| **3** | **TGFB1** | 9606.ENSP00000221930 | 13 | **15** | **NPC1L1** | 9606.ENSP00000289547 | 7 |
| **4** | **TIMP1** | 9606.ENSP00000218388 | 13 | **16** | **TNNI3** | 9606.ENSP00000341838 | 5 |
| **5** | **VWF** | 9606.ENSP00000261405 | 13 | **17** | **ANGPT1** | 9606.ENSP00000428340 | 3 |
| **6** | **APOA1** | 9606.ENSP00000236850 | 12 | **18** | **CRH** | 9606.ENSP00000276571 | 3 |
| **7** | **CASP3** | 9606.ENSP00000311032 | 12 | **19** | **FBN1** | 9606.ENSP00000325527 | 3 |
| **8** | **SCARB2** | 9606.ENSP00000264896 | 12 | **20** | **LAMP2** | 9606.ENSP00000408411 | 3 |
| **9** | **ACE** | 9606.ENSP00000290866 | 11 | **21** | **MMP13** | 9606.ENSP00000260302 | 3 |
| **10** | **CETP** | 9606.ENSP00000200676 | 8 | **22** | **SERPINF1** | 9606.ENSP00000254722 | 3 |
| **11** | **IL18** | 9606.ENSP00000280357 | 8 | **23** | **JAK3** | 9606.ENSP00000432511 | 2 |
| **12** | **LCAT** | 9606.ENSP00000264005 | 8 | **24** | **MC4R** | 9606.ENSP00000299766 | 1 |

Minimum required interaction score: medium confidence 0.4. The nodes with degree>0 are shown in the table.

Available (November 2024):

https://cn.string-db.org/cgi/input?sessionId=bBSQf0gz7Y6u&input_page_show_search=on

**Table S9.** String node degrees of APN+AdipoR2+IBD with APN relevance score ≥10, except APN+AdipoR1+AdipoR2+IBD (Mouse musculus)

| **No.** | **Node** | **Identifier** | **Degree** | **No.** | **Node** | **Identifier** | **Degree** |
| --- | --- | --- | --- | --- | --- | --- | --- |
| **1** | **Apoe** | 10090.ENSMUSP00000133302 | 16 | **14** | **Abcg5** | 10090.ENSMUSP00000069495 | 6 |
| **2** | **Agt** | 10090.ENSMUSP00000066488 | 14 | **15** | **Apoc1** | 10090.ENSMUSP00000104091 | 6 |
| **3** | **Casp3** | 10090.ENSMUSP00000147767 | 13 | **16** | **Gm44805** | 10090.ENSMUSP00000118305 | 6 |
| **4** | **Plg** | 10090.ENSMUSP00000014578 | 13 | **17** | **Mttp** | 10090.ENSMUSP00000096179 | 6 |
| **5** | **Tgfb1** | 10090.ENSMUSP00000002678 | 13 | **18** | **Fbn1** | 10090.ENSMUSP00000099524 | 4 |
| **6** | **Timp1** | 10090.ENSMUSP00000110999 | 13 | **19** | **Tnni3** | 10090.ENSMUSP00000096458 | 4 |
| **7** | **Ace** | 10090.ENSMUSP00000001963 | 11 | **20** | **Crh** | 10090.ENSMUSP00000061185 | 3 |
| **8** | **Vwf** | 10090.ENSMUSP00000107873 | 10 | **21** | **Lamp2** | 10090.ENSMUSP00000074448 | 3 |
| **9** | **Il18** | 10090.ENSMUSP00000151002 | 9 | **22** | **Serpinf1** | 10090.ENSMUSP00000000769 | 3 |
| **10** | **Scarb2** | 10090.ENSMUSP00000031377 | 9 | **23** | **Jak3** | 10090.ENSMUSP00000060073 | 2 |
| **11** | **Angpt1** | 10090.ENSMUSP00000022921 | 8 | **24** | **Mc4r** | 10090.ENSMUSP00000054776 | 1 |
| **12** | **Lcat** | 10090.ENSMUSP00000038232 | 7 | **25** | **Slc17a5** | 10090.ENSMUSP00000056182 | 1 |
| **13** | **Mmp13** | 10090.ENSMUSP00000015394 | 7 |  |  |  |  |

Minimum required interaction score: medium confidence 0.4. The nodes with degree>0 are shown in the table. Available (November 2024):

https://cn.string-db.org/cgi/input?sessionId=bBSQf0gz7Y6u&input_page_show_search=on

**Table S10.** Targets of inflammatory bowel disease (IBD) with relevance score>50 from GeneCards database

| **No.** | **Gene Symbol** | **Description** | **Relevance score** |
| --- | --- | --- | --- |
| **1** | **NOD2** | Nucleotide Binding Oligomerization Domain Containing 2 | 215.772934 |
| **2** | **IL10RA** | Interleukin 10 Receptor Subunit Alpha | 194.368866 |
| **3** | **IL10RB** | Interleukin 10 Receptor Subunit Beta | 185.1512451 |
| **4** | **IL10** | Interleukin 10 | 162.4203491 |
| **5** | **IFNAR2-IL10RB** | IFNAR2-IL10RB Readthrough | 155.9673157 |
| **6** | **PKHD1** | PKHD1 Ciliary IPT Domain Containing Fibrocystin/Polyductin | 152.9289093 |
| **7** | **IL6** | Interleukin 6 | 136.2720032 |
| **8** | **TGFB1** | Transforming Growth Factor Beta 1 | 136.0959473 |
| **9** | **ADAM17** | ADAM Metallopeptidase Domain 17 | 134.8248291 |
| **10** | **TNF** | Tumor Necrosis Factor | 129.2495117 |
| **11** | **PKD1** | Polycystin 1, Transient Receptor Potential Channel Interacting | 122.0446014 |
| **12** | **GAA** | Alpha Glucosidase | 121.6041794 |
| **13** | **NPC1** | NPC Intracellular Cholesterol Transporter 1 | 112.2392197 |
| **14** | **AGL** | Amylo-Alpha-1, 6-Glucosidase, 4-Alpha-Glucanotransferase | 111.9310989 |
| **15** | **APP** | Amyloid Beta Precursor Protein | 107.3797607 |
| **16** | **LMNA** | Lamin A/C | 100.2545776 |
| **17** | **IL1B** | Interleukin 1 Beta | 95.99531555 |
| **18** | **VWF** | Von Willebrand Factor | 95.99396515 |
| **19** | **STAT3** | Signal Transducer And Activator Of Transcription 3 | 94.07025146 |
| **20** | **GBE1** | 1,4-Alpha-Glucan Branching Enzyme 1 | 92.72264862 |
| **21** | **APOE** | Apolipoprotein E | 91.96401215 |
| **22** | **GBA1** | Glucosylceramidase Beta 1 | 91.73078156 |
| **23** | **PSEN1** | Presenilin 1 | 90.65142822 |
| **24** | **DYNC1H1** | Dynein Cytoplasmic 1 Heavy Chain 1 | 90.61712646 |
| **25** | **RET** | Ret Proto-Oncogene | 90.18618011 |
| **26** | **LRRK2** | Leucine Rich Repeat Kinase 2 | 89.24397278 |
| **27** | **ATP7B** | ATPase Copper Transporting Beta | 88.8192749 |
| **28** | **TERT** | Telomerase Reverse Transcriptase | 88.80545044 |
| **29** | **CYBB** | Cytochrome B-245 Beta Chain | 87.29447937 |
| **30** | **IL23R** | Interleukin 23 Receptor | 87.11198425 |
| **31** | **G6PC1** | Glucose-6-Phosphatase Catalytic Subunit 1 | 86.70644379 |
| **32** | **SMPD1** | Sphingomyelin Phosphodiesterase 1 | 85.43078613 |
| **33** | **GLA** | Galactosidase Alpha | 84.93667603 |
| **34** | **SQSTM1** | Sequestosome 1 | 84.80667114 |
| **35** | **MFN2** | Mitofusin 2 | 84.78187561 |
| **36** | **TLR4** | Toll Like Receptor 4 | 83.05909729 |
| **37** | **MPZ** | Myelin Protein Zero | 82.88692474 |
| **38** | **CTLA4** | Cytotoxic T-Lymphocyte Associated Protein 4 | 82.87289429 |
| **39** | **IFNG** | Interferon Gamma | 82.1277771 |
| **40** | **SLC37A4** | Solute Carrier Family 37 Member 4 | 82.07878113 |
| **41** | **SLC17A5** | Solute Carrier Family 17 Member 5 | 81.19107819 |
| **42** | **SH3TC2** | SH3 Domain And Tetratricopeptide Repeats 2 | 80.92897797 |
| **43** | **CYBA** | Cytochrome B-245 Alpha Chain | 80.71356201 |
| **44** | **NEFL** | Neurofilament Light Chain | 80.62306976 |
| **45** | **RIPK1** | Receptor Interacting Serine/Threonine Kinase 1 | 80.19108582 |
| **46** | **EGFR** | Epidermal Growth Factor Receptor | 78.94490051 |
| **47** | **AARS1** | Alanyl-TRNA Synthetase 1 | 78.60431671 |
| **48** | **CRP** | C-Reactive Protein | 77.71885681 |
| **49** | **COL1A1** | Collagen Type I Alpha 1 Chain | 77.23464203 |
| **50** | **SNCA** | Synuclein Alpha | 77.1419754 |
| **51** | **MEFV** | MEFV Innate Immunity Regulator, Pyrin | 76.7053833 |
| **52** | **NCF2** | Neutrophil Cytosolic Factor 2 | 76.41915894 |
| **53** | **PMP22** | Peripheral Myelin Protein 22 | 76.34007263 |
| **54** | **STXBP2** | Syntaxin Binding Protein 2 | 76.29811096 |
| **55** | **NAGLU** | N-Acetyl-Alpha-Glucosaminidase | 76.23946381 |
| **56** | **PKD2** | Polycystin 2, Transient Receptor Potential Cation Channel | 75.57750702 |
| **57** | **IGHMBP2** | Immunoglobulin Mu DNA Binding Protein 2 | 75.35311127 |
| **58** | **CXCL8** | C-X-C Motif Chemokine Ligand 8 | 74.88278198 |
| **59** | **VCP** | Valosin Containing Protein | 74.34550476 |
| **60** | **ATG16L1** | Autophagy Related 16 Like 1 | 74.15219879 |
| **61** | **NFKB1** | Nuclear Factor Kappa B Subunit 1 | 74.02459717 |
| **62** | **HEXA** | Hexosaminidase Subunit Alpha | 73.24966431 |
| **63** | **HLA-DRB1** | Major Histocompatibility Complex, Class II, DR Beta 1 | 72.8835907 |
| **64** | **PRKN** | Parkin RBR E3 Ubiquitin Protein Ligase | 72.22246552 |
| **65** | **NLRP3** | NLR Family Pyrin Domain Containing 3 | 71.91455078 |
| **66** | **IAH1** | Isoamyl Acetate Hydrolyzing Esterase 1 (Putative) | 71.77783203 |
| **67** | **PYGM** | Glycogen Phosphorylase, Muscle Associated | 71.64316559 |
| **68** | **ATP7A** | ATPase Copper Transporting Alpha | 71.64143372 |
| **69** | **GALC** | Galactosylceramidase | 71.50180054 |
| **70** | **KIF1B** | Kinesin Family Member 1B | 70.84518433 |
| **71** | **GDAP1** | Ganglioside Induced Differentiation Associated Protein 1 | 70.74599457 |
| **72** | **DNM2** | Dynamin 2 | 70.11238098 |
| **73** | **HEXB** | Hexosaminidase Subunit Beta | 70.10643005 |
| **74** | **FIG4** | FIG4 Phosphoinositide 5-Phosphatase | 69.2677002 |
| **75** | **TP53** | Tumor Protein P53 | 68.97905731 |
| **76** | **MAPT** | Microtubule Associated Protein Tau | 68.4057312 |
| **77** | **ABCB1** | ATP Binding Cassette Subfamily B Member 1 | 68.33203125 |
| **78** | **IL1RN** | Interleukin 1 Receptor Antagonist | 67.91143036 |
| **79** | **PTEN** | Phosphatase And Tensin Homolog | 67.69577026 |
| **80** | **IL21** | Interleukin 21 | 67.63804626 |
| **81** | **PIK3CA** | Phosphatidylinositol-4,5-Bisphosphate 3-Kinase Catalytic Subunit Alpha | 67.13624573 |
| **82** | **CCL2** | C-C Motif Chemokine Ligand 2 | 67.0897522 |
| **83** | **PINK1** | PTEN Induced Kinase 1 | 66.91882324 |
| **84** | **ABCA4** | ATP Binding Cassette Subfamily A Member 4 | 66.86011505 |
| **85** | **BDNF-AS** | BDNF Antisense RNA | 66.48675537 |
| **86** | **ACE** | Angiotensin I Converting Enzyme | 66.26078033 |
| **87** | **FOXP3** | Forkhead Box P3 | 65.96025085 |
| **88** | **ITCH** | Itchy E3 Ubiquitin Protein Ligase | 65.91847229 |
| **89** | **XIAP** | X-Linked Inhibitor Of Apoptosis | 65.47042847 |
| **90** | **IL2RA** | Interleukin 2 Receptor Subunit Alpha | 65.1517868 |
| **91** | **LOC130066558** | ATAC-STARR-Seq Lymphoblastoid Silent Region 13254 | 65.08691406 |
| **92** | **STAT1** | Signal Transducer And Activator Of Transcription 1 | 65.08065796 |
| **93** | **CD40LG** | CD40 Ligand | 64.49645996 |
| **94** | **PRNP** | Prion Protein (Kanno Blood Group) | 64.31899261 |
| **95** | **MPO** | Myeloperoxidase | 63.98053741 |
| **96** | **TRPV4** | Transient Receptor Potential Cation Channel Subfamily V Member 4 | 63.96165466 |
| **97** | **LOC106627981** | GBA Recombination Region | 63.40478516 |
| **98** | **INF2** | Inverted Formin 2 | 63.38739014 |
| **99** | **TNFRSF1A** | TNF Receptor Superfamily Member 1A | 63.25193787 |
| **100** | **GJB1** | Gap Junction Protein Beta 1 | 63.22052002 |
| **101** | **EGR2** | Early Growth Response 2 | 63.13110352 |
| **102** | **NCF4** | Neutrophil Cytosolic Factor 4 | 63.05970001 |
| **103** | **IFIH1** | Interferon Induced With Helicase C Domain 1 | 62.88167191 |
| **104** | **MYO5B** | Myosin VB | 62.13356781 |
| **105** | **IL37** | Interleukin 37 | 62.01406479 |
| **106** | **IL4** | Interleukin 4 | 61.9113884 |
| **107** | **ENG** | Endoglin | 61.77528381 |
| **108** | **MTHFR** | Methylenetetrahydrofolate Reductase | 61.71418762 |
| **109** | **NOTCH1** | Notch Receptor 1 | 61.21076202 |
| **110** | **PARK7** | Parkinsonism Associated Deglycase | 61.06502914 |
| **111** | **TLR2** | Toll Like Receptor 2 | 60.9702301 |
| **112** | **IL1A** | Interleukin 1 Alpha | 60.27116394 |
| **113** | **IL17A** | Interleukin 17A | 60.20834351 |
| **114** | **BCKDHB** | Branched Chain Keto Acid Dehydrogenase E1 Subunit Beta | 59.66186523 |
| **115** | **SYNJ1** | Synaptojanin 1 | 59.60185242 |
| **116** | **BCKDHA** | Branched Chain Keto Acid Dehydrogenase E1 Subunit Alpha | 59.5008049 |
| **117** | **H19** | H19 Imprinted Maternally Expressed Transcript | 59.17147064 |
| **118** | **CD40** | CD40 Molecule | 58.88022614 |
| **119** | **TTC7A** | Tetratricopeptide Repeat Domain 7A | 58.66932297 |
| **120** | **CFTR** | CF Transmembrane Conductance Regulator | 58.66273499 |
| **121** | **PTGS2** | Prostaglandin-Endoperoxide Synthase 2 | 58.59539032 |
| **122** | **ABCA1** | ATP Binding Cassette Subfamily A Member 1 | 58.40212631 |
| **123** | **NDRG1** | N-Myc Downstream Regulated 1 | 58.22132111 |
| **124** | **IBD5** | Inflammatory Bowel Disease 5 | 57.8926506 |
| **125** | **FLNA** | Filamin A | 57.72956848 |
| **126** | **HLA-B** | Major Histocompatibility Complex, Class I, B | 57.67527771 |
| **127** | **DBT** | Dihydrolipoamide Branched Chain Transacylase E2 | 57.54059219 |
| **128** | **NFKBIA** | NFKB Inhibitor Alpha | 57.10494995 |
| **129** | **RPL36A-HNRNPH2** | RPL36A-HNRNPH2 Readthrough | 56.97210693 |
| **130** | **PSEN2** | Presenilin 2 | 56.87879944 |
| **131** | **MARS1** | Methionyl-TRNA Synthetase 1 | 56.71995926 |
| **132** | **CYP27A1** | Cytochrome P450 Family 27 Subfamily A Member 1 | 56.68696594 |
| **133** | **COL1A2** | Collagen Type I Alpha 2 Chain | 56.65833282 |
| **134** | **GUCY2D** | Guanylate Cyclase 2D, Retinal | 56.55353165 |
| **135** | **IL2** | Interleukin 2 | 56.53403854 |
| **136** | **PSAP** | Prosaposin | 56.43883133 |
| **137** | **INS** | Insulin | 55.97502518 |
| **138** | **IL18** | Interleukin 18 | 55.95170593 |
| **139** | **ADA** | Adenosine Deaminase | 55.832798 |
| **140** | **PPARG** | Peroxisome Proliferator Activated Receptor Gamma | 55.4983139 |
| **141** | **TNFAIP3** | TNF Alpha Induced Protein 3 | 55.47537994 |
| **142** | **AR** | Androgen Receptor | 55.34187698 |
| **143** | **MED25** | Mediator Complex Subunit 25 | 55.24654007 |
| **144** | **NPC2** | NPC Intracellular Cholesterol Transporter 2 | 54.96887207 |
| **145** | **IKBKG** | Inhibitor Of Nuclear Factor Kappa B Kinase Regulatory Subunit Gamma | 54.92127609 |
| **146** | **IRF5** | Interferon Regulatory Factor 5 | 54.89757538 |
| **147** | **FAS** | Fas Cell Surface Death Receptor | 54.82550049 |
| **148** | **IL13** | Interleukin 13 | 54.81859589 |
| **149** | **ICAM1** | Intercellular Adhesion Molecule 1 | 54.75559998 |
| **150** | **TTR** | Transthyretin | 54.74320221 |
| **151** | **HSPB1** | Heat Shock Protein Family B (Small) Member 1 | 54.59003448 |
| **152** | **HLA-DQB1** | Major Histocompatibility Complex, Class II, DQ Beta 1 | 54.48911285 |
| **153** | **NOS3** | Nitric Oxide Synthase 3 | 54.46019745 |
| **154** | **MMP9** | Matrix Metallopeptidase 9 | 54.42345428 |
| **155** | **GFAP** | Glial Fibrillary Acidic Protein | 54.39175034 |
| **156** | **TLR3** | Toll Like Receptor 3 | 54.38482285 |
| **157** | **CEP290** | Centrosomal Protein 290 | 54.33508682 |
| **158** | **TGFBR2** | Transforming Growth Factor Beta Receptor 2 | 54.33243179 |
| **159** | **ELANE** | Elastase, Neutrophil Expressed | 54.23564148 |
| **160** | **CTNNB1** | Catenin Beta 1 | 54.19369125 |
| **161** | **BSCL2** | BSCL2 Lipid Droplet Biogenesis Associated, Seipin | 54.01826096 |
| **162** | **PTPN22** | Protein Tyrosine Phosphatase Non-Receptor Type 22 | 53.86174774 |
| **163** | **CYLD-AS1** | CYLD Antisense RNA 1 | 53.68192291 |
| **164** | **CASP8** | Caspase 8 | 53.55035782 |
| **165** | **LRSAM1** | Leucine Rich Repeat And Sterile Alpha Motif Containing 1 | 53.45860291 |
| **166** | **POLG** | DNA Polymerase Gamma, Catalytic Subunit | 53.43714523 |
| **167** | **LIPA** | Lipase A, Lysosomal Acid Type | 53.418396 |
| **168** | **GYS1** | Glycogen Synthase 1 | 53.27164841 |
| **169** | **GATA3** | GATA Binding Protein 3 | 53.24220657 |
| **170** | **CBS** | Cystathionine Beta-Synthase | 53.20590973 |
| **171** | **ASPA** | Aspartoacylase | 53.16976547 |
| **172** | **IL21R** | Interleukin 21 Receptor | 53.03694153 |
| **173** | **LAMP2** | Lysosomal Associated Membrane Protein 2 | 52.98726654 |
| **174** | **MTMR2** | Myotubularin Related Protein 2 | 52.8513298 |
| **175** | **MVK** | Mevalonate Kinase | 52.81099319 |
| **176** | **F2** | Coagulation Factor II, Thrombin | 52.74520493 |
| **177** | **PFKM** | Phosphofructokinase, Muscle | 52.72679138 |
| **178** | **F8** | Coagulation Factor VIII | 52.68416595 |
| **179** | **HMOX1** | Heme Oxygenase 1 | 52.62273788 |
| **180** | **TNFRSF11B** | TNF Receptor Superfamily Member 11b | 52.57676315 |
| **181** | **NLRP12** | NLR Family Pyrin Domain Containing 12 | 52.5217514 |
| **182** | **ALB** | Albumin | 52.48821259 |
| **183** | **NFAT5** | Nuclear Factor Of Activated T Cells 5 | 52.48316956 |
| **184** | **SOD1** | Superoxide Dismutase 1 | 52.3216629 |
| **185** | **PDGFRA** | Platelet Derived Growth Factor Receptor Alpha | 52.26938248 |
| **186** | **CARD8** | Caspase Recruitment Domain Family Member 8 | 52.15636826 |
| **187** | **MIR21** | MicroRNA 21 | 52.15202332 |
| **188** | **SH2D1A** | SH2 Domain Containing 1A | 52.07228088 |
| **189** | **IRGM** | Immunity Related GTPase M | 51.99317932 |
| **190** | **BDNF** | Brain Derived Neurotrophic Factor | 51.88354111 |
| **191** | **PLA2G6** | Phospholipase A2 Group VI | 51.77427673 |
| **192** | **HBB** | Hemoglobin Subunit Beta | 51.65487671 |
| **193** | **ARPC1B** | Actin Related Protein 2/3 Complex Subunit 1B | 51.5916748 |
| **194** | **CLCN1** | Chloride Voltage-Gated Channel 1 | 51.55345535 |
| **195** | **RYR1** | Ryanodine Receptor 1 | 51.40711975 |
| **196** | **IL19** | Interleukin 19 | 51.211483 |
| **197** | **LRP5** | LDL Receptor Related Protein 5 | 51.16999054 |
| **198** | **PRF1** | Perforin 1 | 50.8973999 |
| **199** | **IKBKB** | Inhibitor Of Nuclear Factor Kappa B Kinase Subunit Beta | 50.83678055 |
| **200** | **GDNF** | Glial Cell Derived Neurotrophic Factor | 50.64051819 |
| **201** | **MMP1** | Matrix Metallopeptidase 1 | 50.60256958 |
| **202** | **GNE** | Glucosamine (UDP-N-Acetyl)-2-Epimerase/N-Acetylmannosamine Kinase | 50.5302887 |
| **203** | **LITAF** | Lipopolysaccharide Induced TNF Factor | 50.46647263 |
| **204** | **ELP1** | Elongator Acetyltransferase Complex Subunit 1 | 50.35388184 |
| **205** | **PIK3CD** | Phosphatidylinositol-4,5-Bisphosphate 3-Kinase Catalytic Subunit Delta | 50.30455017 |
| **206** | **LINC01672** | Long Intergenic Non-Protein Coding RNA 1672 | 50.27324677 |
| **207** | **IBD2** | Inflammatory Bowel Disease 2 | 50.09247589 |

Available (November 2024): https://www.genecards.org/Search


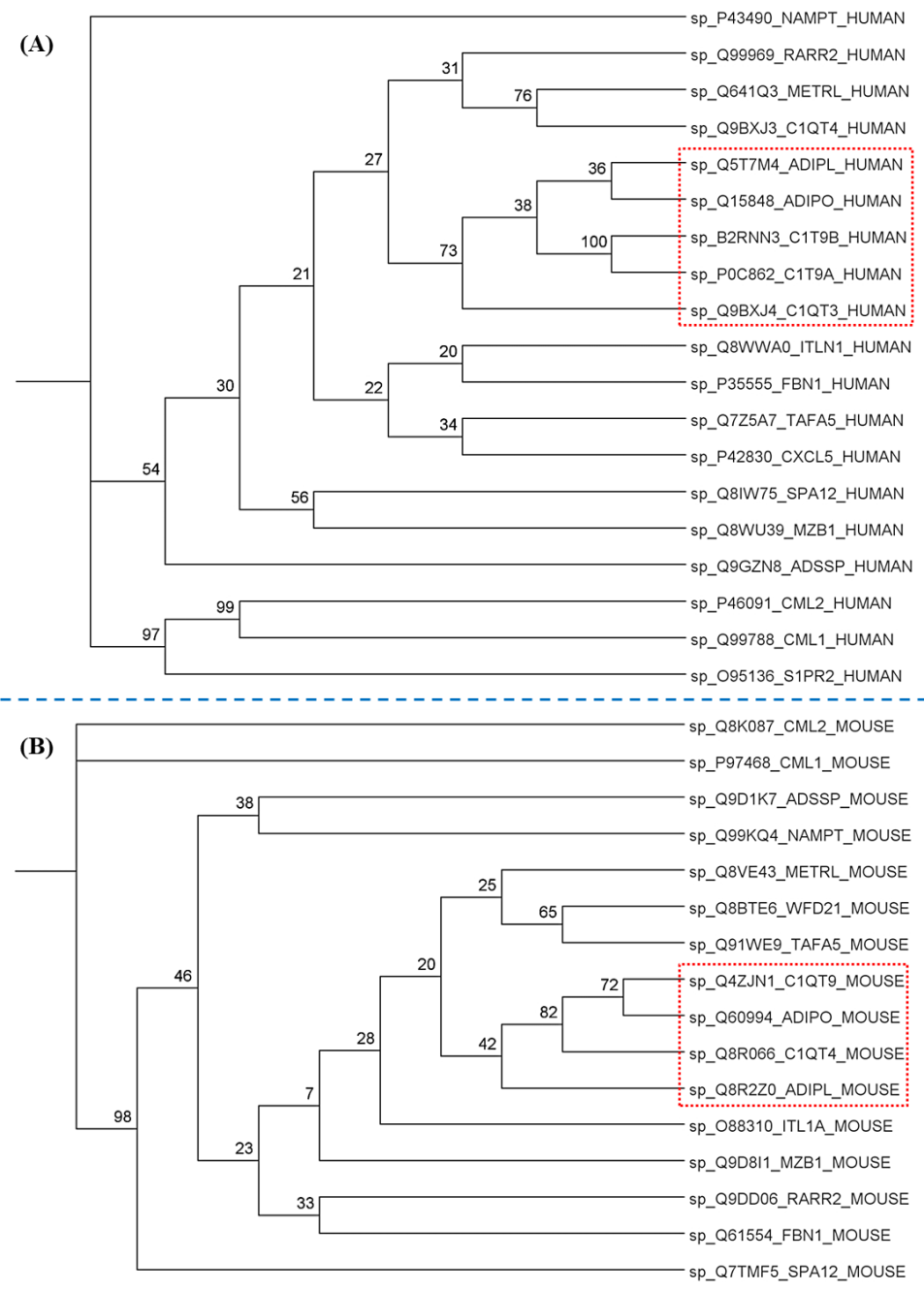


**Figure S1.** Phylogenetic tree of adipokine family

(A) Homo sapiens (human); (B) Mus musculus (Mouse). NAMPT: nicotinamide phosphoribosyltransferase; RARR2: retinoic acid receptor responder protein 2; METRL: Meteorin-like protein; C1QT: complement C1q/ tumor necrosis factor-related protein; ADIPL: adipolin; ADIPO: adiponectin (30 kDa adipocyte complement-related protein); C1T: complement C1q and tumor necrosis factor-related protein (C1q/TNF-related protein); ITLN1: intelectin-1; FBN1: fibrillin-1; TAFA5: chemokine-like protein TAFA-5; CXCL5: C-X-C motif chemokine 5; SPA12: serpin A12; MZB1: marginal zone B- and B1-cell-specific protein; ADSSP: adipose-secreted signaling protein; CML: chemerin-like receptor; S1PR2: sphingosine 1-phosphate receptor 2; ITL1A: intelectin-1a; WFD21: protein Wfdc21 (Wdnm1-like protein). All human and mouse proteins domains are derived from UniProtKB database [Available (November 2024): https://www.uniprot.org/uniprotkb/]. The TBtools-II software was used for data visualization^1^.


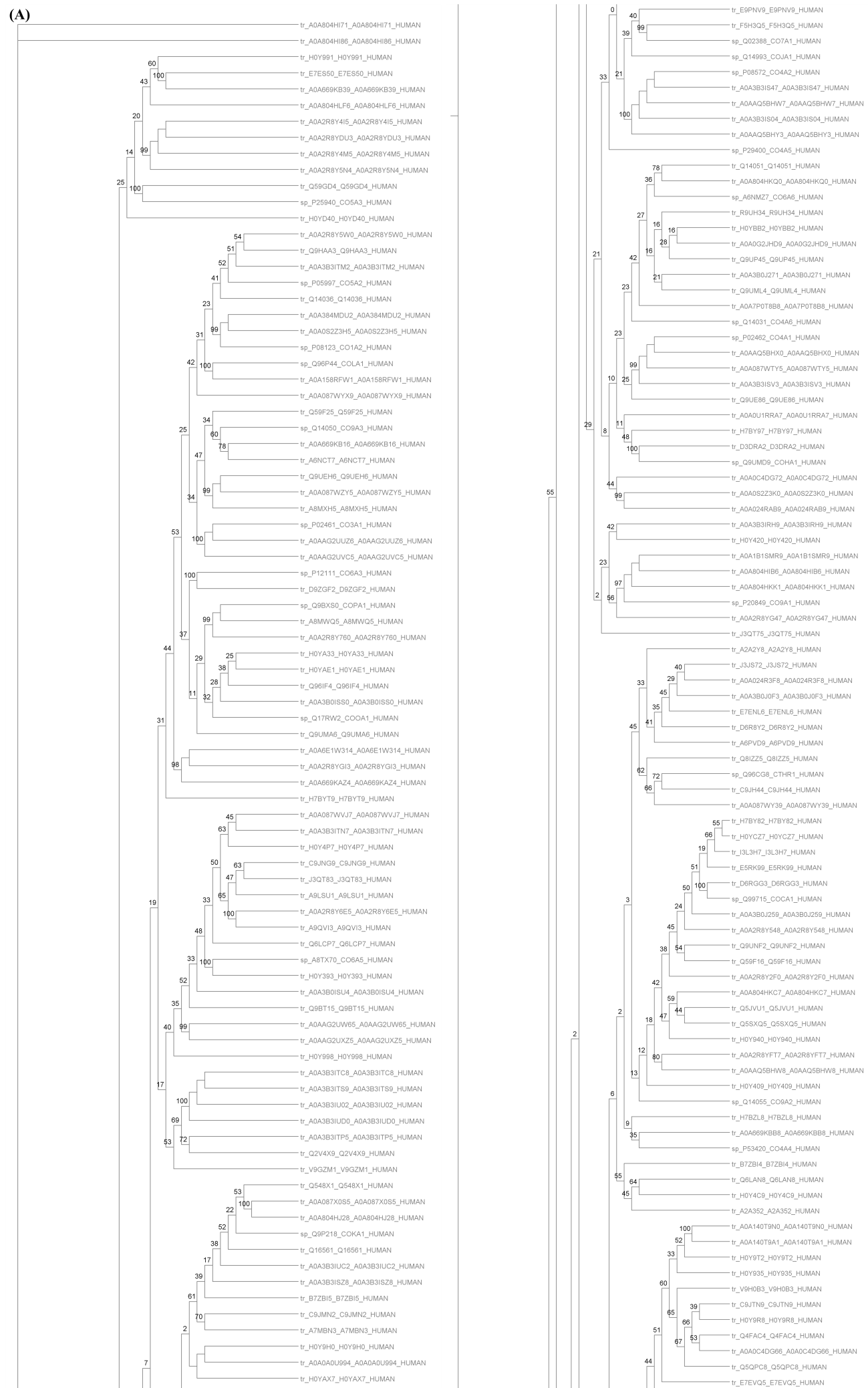


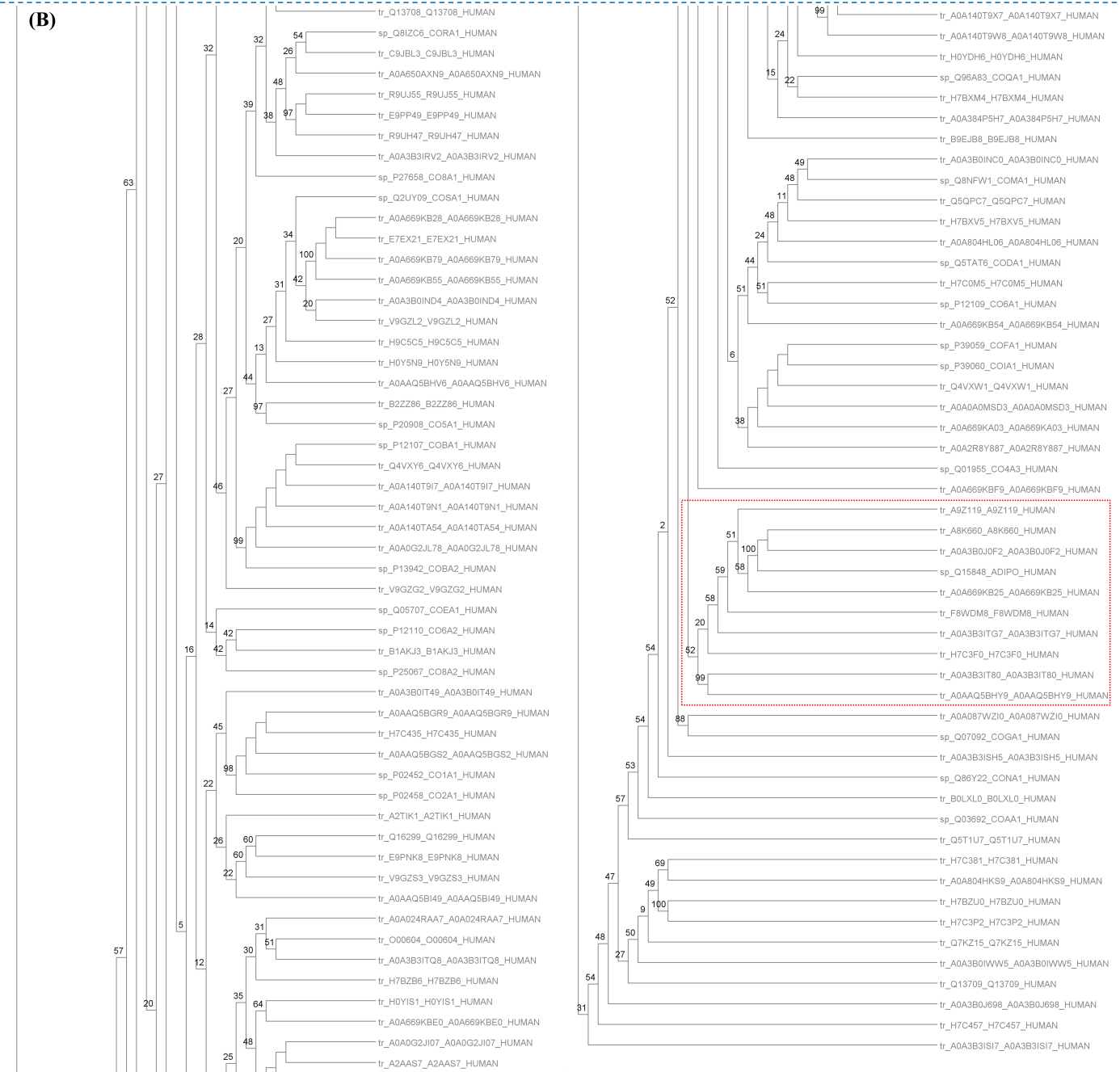


**Figure S2.** Phylogenetic tree of collagen (Homo sapiens) family

(B) is continuation of (A). A9Z119: collagen type VII alpha 1; A8K660: adiponectin [cDNA FLJ78108, highly similar to Homo sapiens adiponectin, C1Q and collagen domain containing (ADIPOQ), mRNA]; A0A3B0J0F2: adiponectin D; Q15848: adiponectin (30 kDa adipocyte complement-related protein); A0A669KB25: collagen type XIII alpha 1 chain; F8WDM8: collagen type XXIV alpha 1 chain; A0A3B3ITG7: collagen type IV alpha 1 chain; H7C3F0: collagen type XVI alpha 1 chain; A0A3B3IT80: collagen type IV alpha 2 chain; A0AAQ5BHY9: collagen type IV alpha 2 chain. All human and mouse proteins domains are derived from UniProtKB database [Available (November 2024): https://www.uniprot.org/uniprotkb/]. The TBtools-II software was used for data visualization^1^.


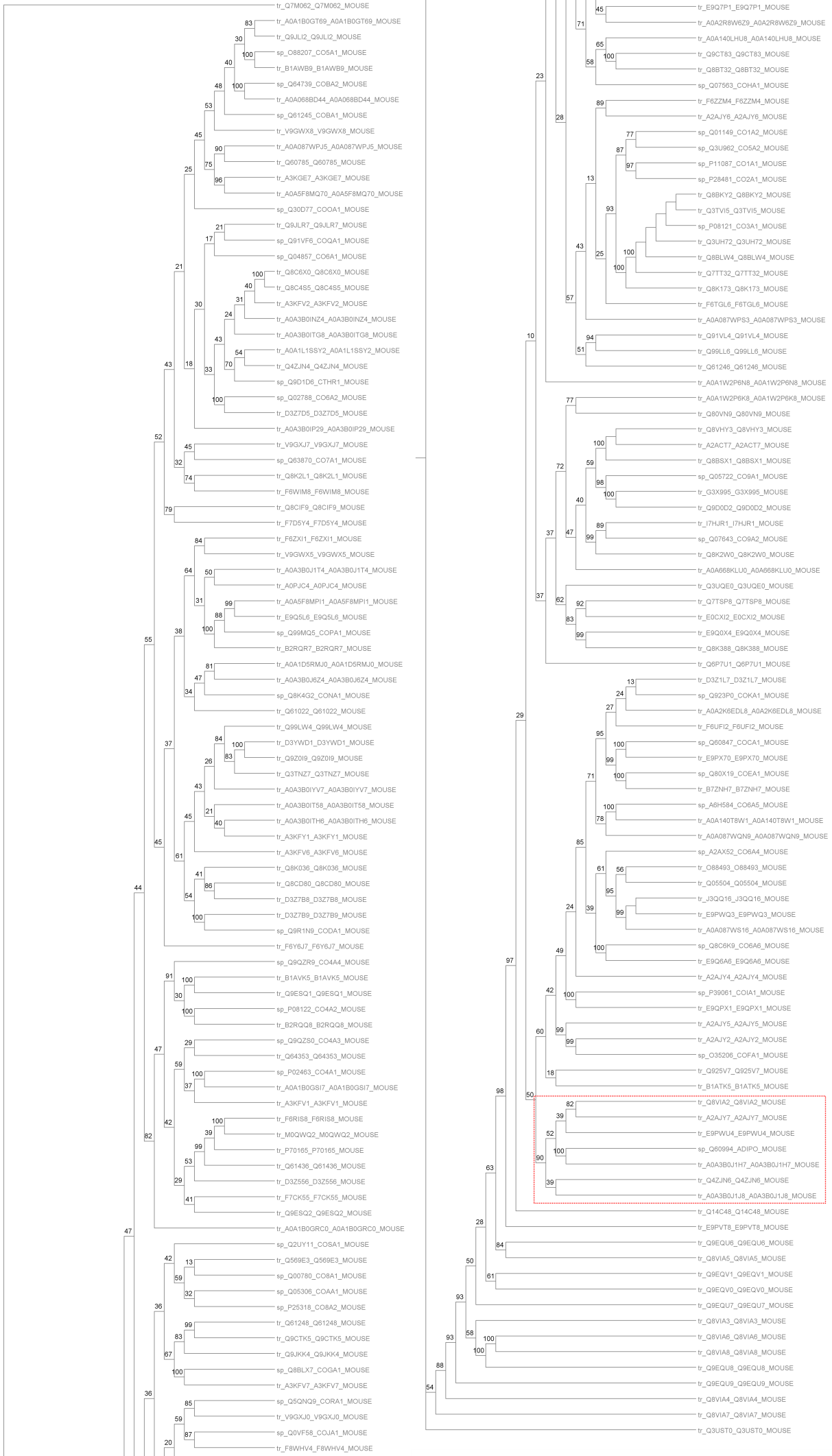


**Figure S3.** Phylogenetic tree of collagen (Mus musculus) family

Q8VIA2: A1(XI) collagen; A2AJY7: collagen, type XV, alpha 1; E9PWU4: adiponectin, C1Q and collagen domain containing; Q60994-ADIPO: adiponectin (30 kDa adipocyte complement-related protein); A0A3B0J1H7: adiponectin d; Q4ZJN6: adiponectin m (C1qTNF3); A0A3B0J1J8: adiponectin p. All human and mouse proteins domains are derived from UniProtKB database [Available (November 2024): https://www.uniprot.org/uniprotkb/]. The TBtools-II software was used for data visualization^1^.


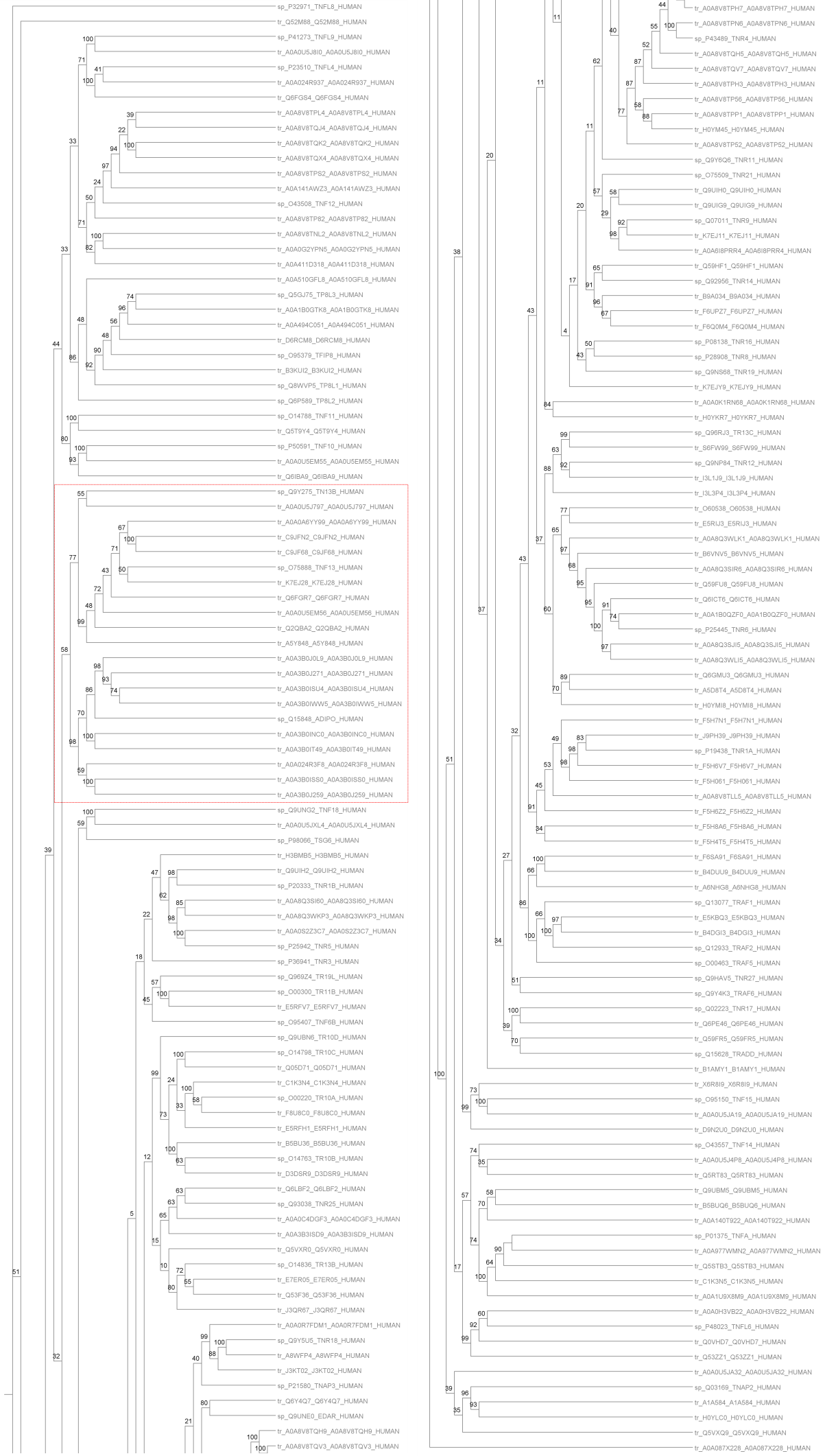


**Figure S4.** Phylogenetic tree of tumor necrosis factor (TNF) family (Homo sapiens)

Q9Y275-TN13B: tumor necrosis factor ligand superfamily member 13B; A0A0U5J797: tumor necrosis factor ligand 7C; A0A0A6YY99: protein TNFSF12-TNFSF13; C9JFN2: TNF superfamily member 13; C9JF68: TNF superfamily member 13; O75888-TNF13: tumor necrosis factor ligand superfamily member 13; K7EJ28: TNF superfamily member 13; Q6FGR7: TNFSF13 protein; A0A0U5EM56: tumor necrosis factor ligand 7B; Q2QBA2: TNF superfamily member 13; A5Y848: tumor necrosis factor ligand superfamily member 13 epsilon; A0A3B0J0L9: adiponectin N; A0A3B0J271: adiponectin P (C1q and tumor necrosis factor related protein 1, isoform CRA_a); A0A3B0ISU4: adiponectin Q; A0A3B0IWW5: adiponectin O (C1q and tumor necrosis factor related protein 8); Q15848-ADIPO: adiponectin (30 kDa adipocyte complement-related protein); A0A3B0INC0: adiponectin H; A0A3B0IT49: adiponectin G; A0A024R3F8: adiponectin E; A0A3B0ISS0: adiponectin F2; A0A3B0J259: adiponectin F1. All human and mouse proteins domains are derived from UniProtKB database [Available (November 2024): https://www.uniprot.org/uniprotkb/]. The TBtools-II software was used for data visualization^1^.


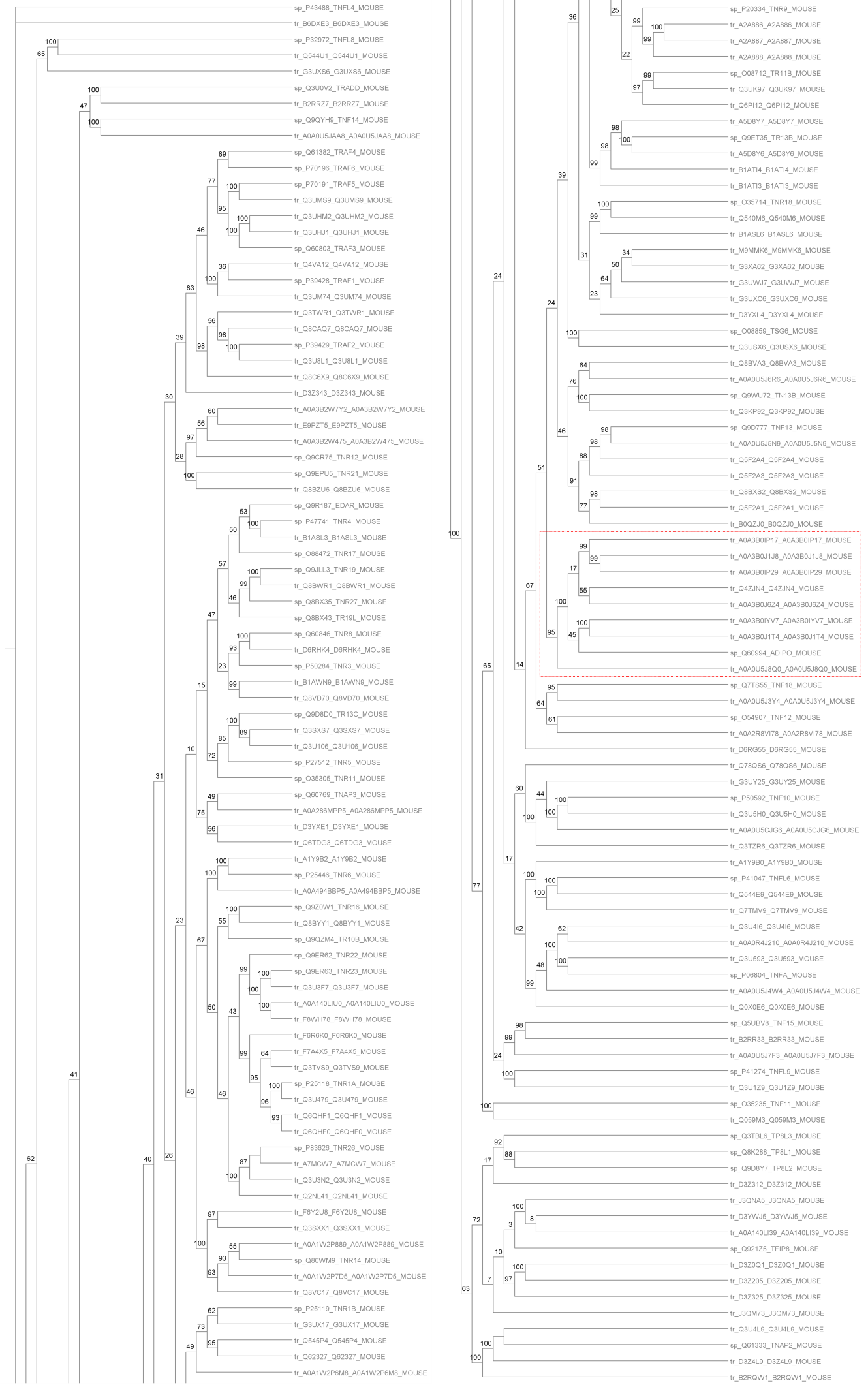


**Figure S5.** Phylogenetic tree of tumor necrosis factor (TNF) family (Mus musculus)

A0A3B0IP17: adiponectin n; A0A3B0J1J8: adiponectin p; A0A3B0IP29: adiponectin q; Q4ZJN4: adiponectin e (C1qTNF5); A0A3B0J6Z4: adiponectin f1; A0A3B0IYV7: adiponectin h; A0A3B0J1T4: adiponectin g; Q60994-ADIPO: adiponectin (30 kDa adipocyte complement-related protein); A0A0U5J8Q0: tumor necrosis factor ligand 7c. All human and mouse proteins domains are derived from UniProtKB database [Available (November 2024): https://www.uniprot.org/uniprotkb/]. The TBtools-II software was used for data visualization^1^.

**
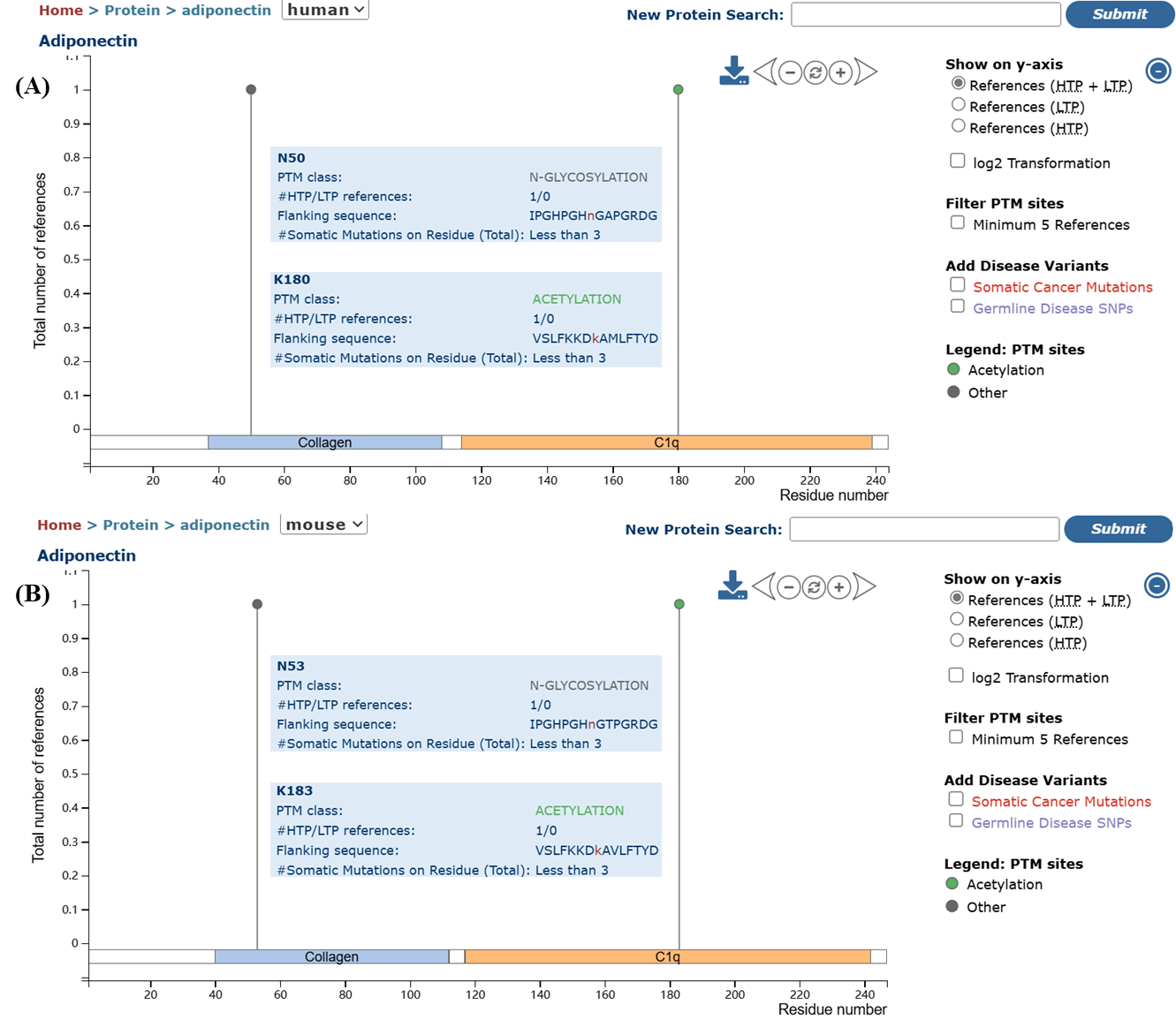
**

**Figure S6.** Post-translational modification sites of adiponectin from phosphoSitePlus (v.6.7.5)

APN: adiponectin; AdipoR1: adiponectin receptor 1; AdipoR2: adiponectin receptor 2; IBD: inflammatory bowel disease. Available (November 2024): https://www.phosphosite.org/homeAction

**
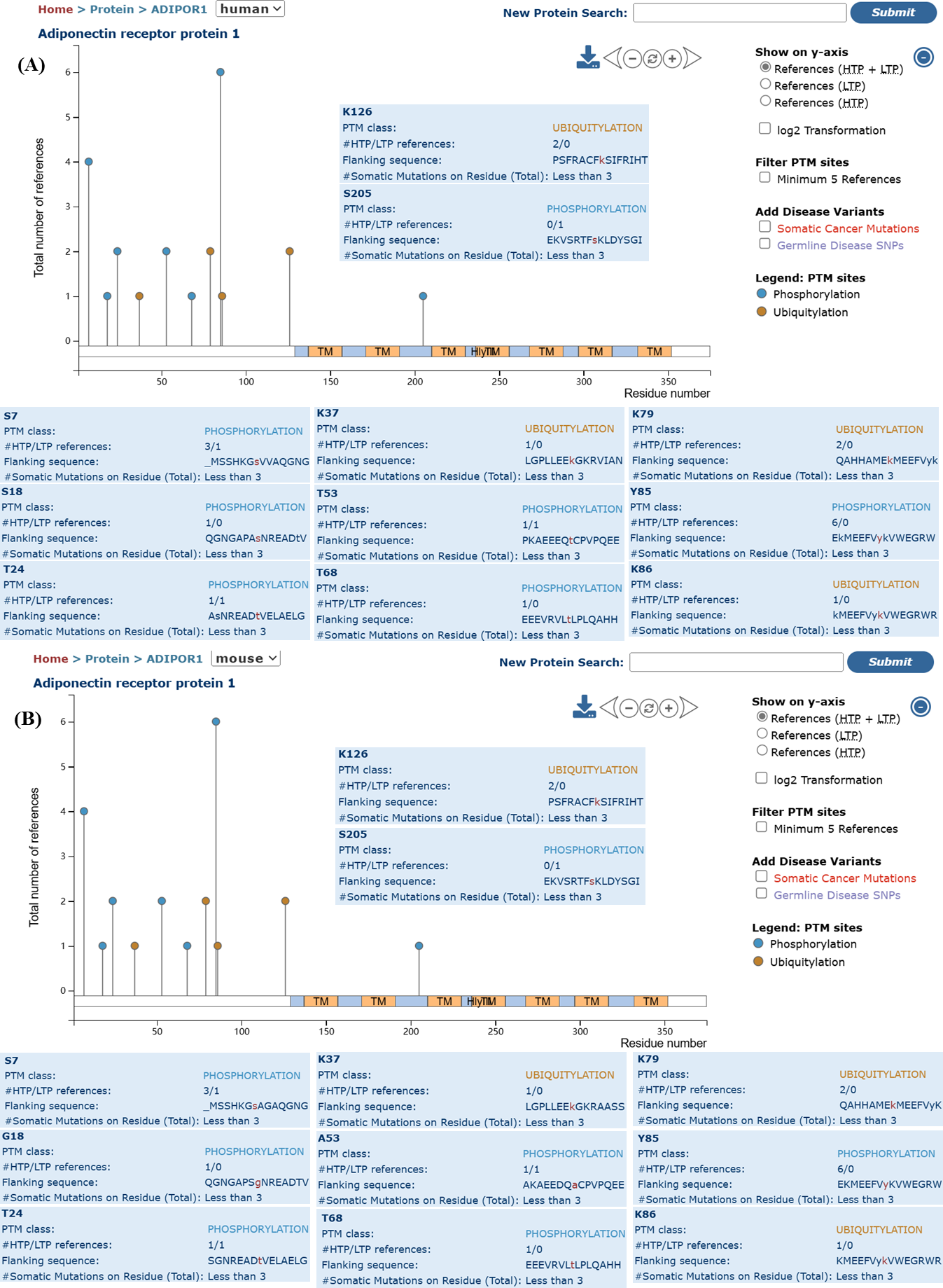
**

**Figure S7.** Post-translational modification sites of AdipoR1 from phosphoSitePlus (v.6.7.5)

APN: adiponectin; AdipoR1: adiponectin receptor 1; AdipoR2: adiponectin receptor 2; IBD: inflammatory bowel disease. Available (November 2024): https://www.phosphosite.org/homeAction

**
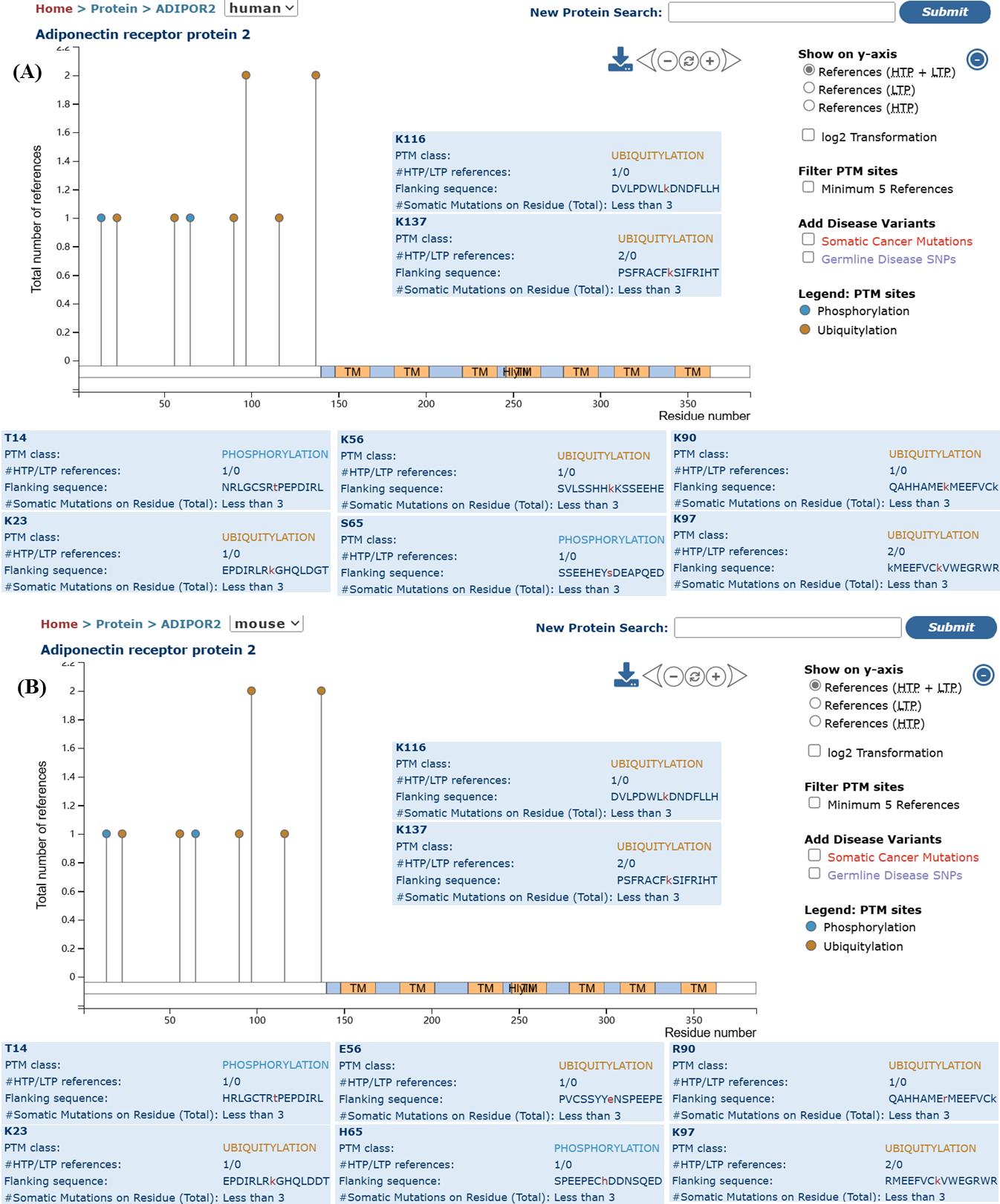
**

**Figure S8.** Post-translational modification sites of AdipoR2 from phosphoSitePlus (v.6.7.5)

APN: adiponectin; AdipoR1: adiponectin receptor 1; AdipoR2: adiponectin receptor 2; IBD: inflammatory bowel disease. Available (November 2024): https://www.phosphosite.org/homeAction

**
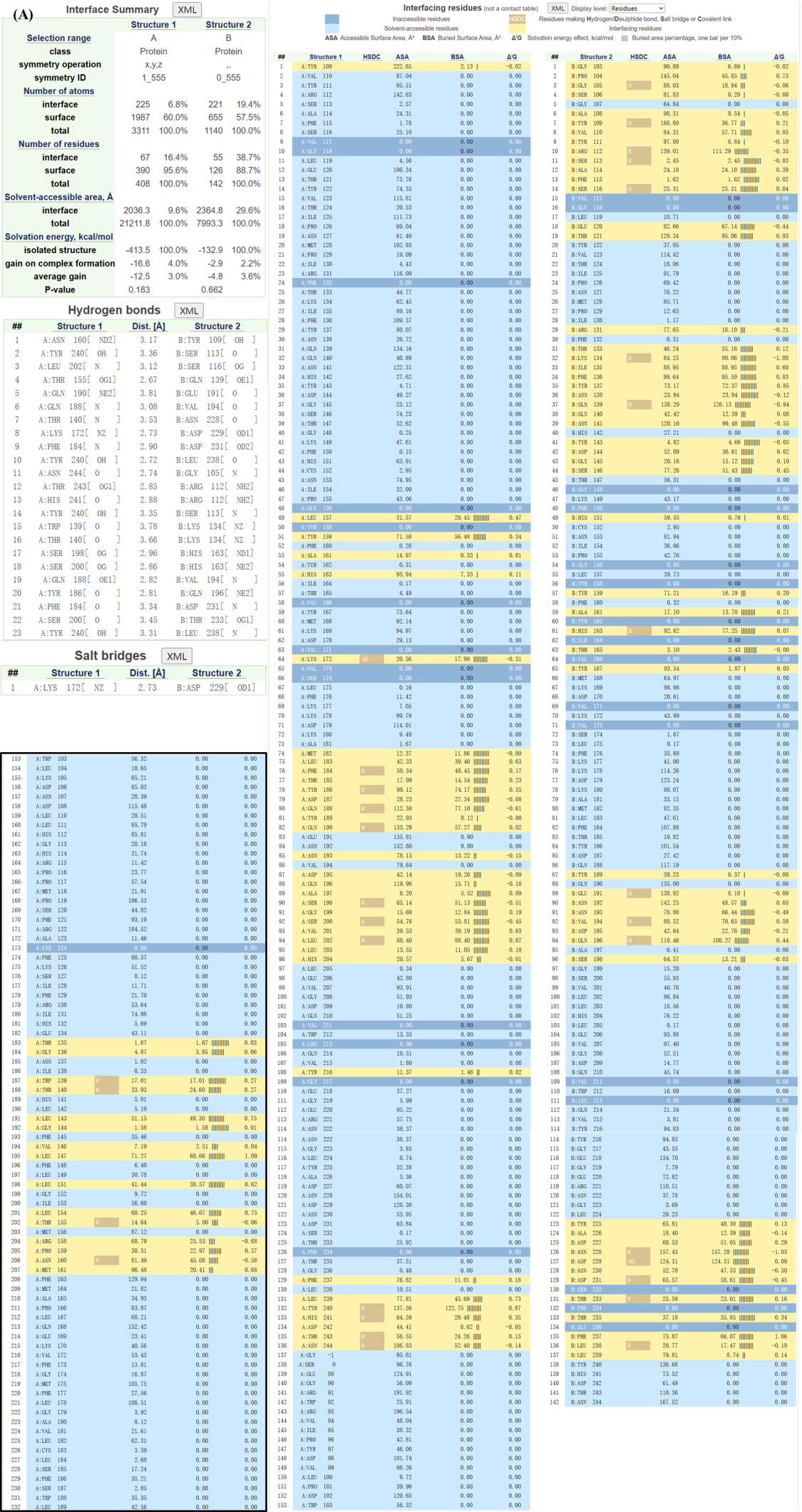
**

**
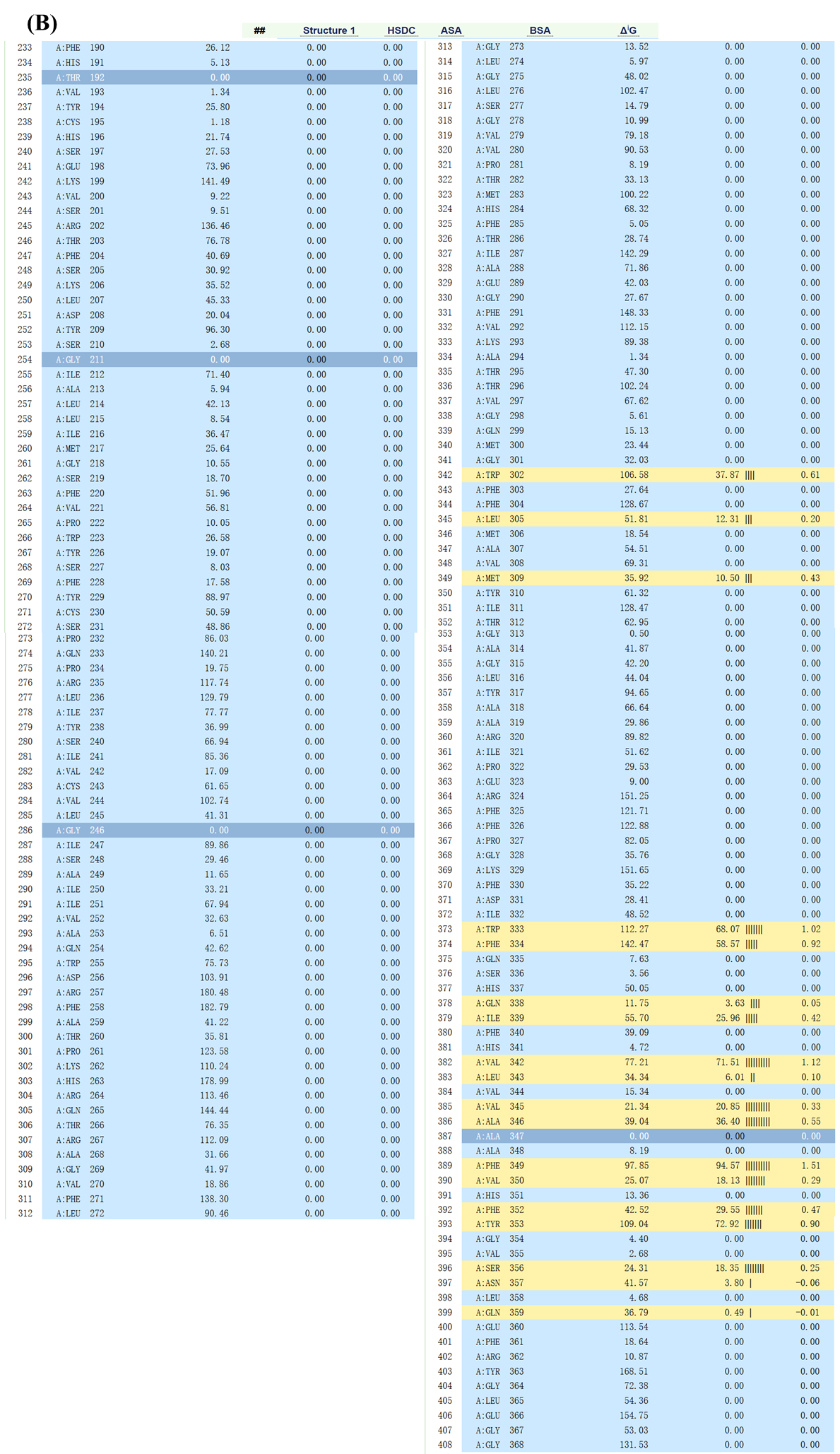
**

**Figure S9.** Molecular docking parameters of APN (6U66) + AdipoR1 (5LXG)

(B) is continuation of (A). ZDOCK is used for docking and PDBePISA is applied to analyze docking results. Available (November 2024): https://zdock.wenglab.org/; https://www.ebi.ac.uk/msd-srv/prot_int/

**
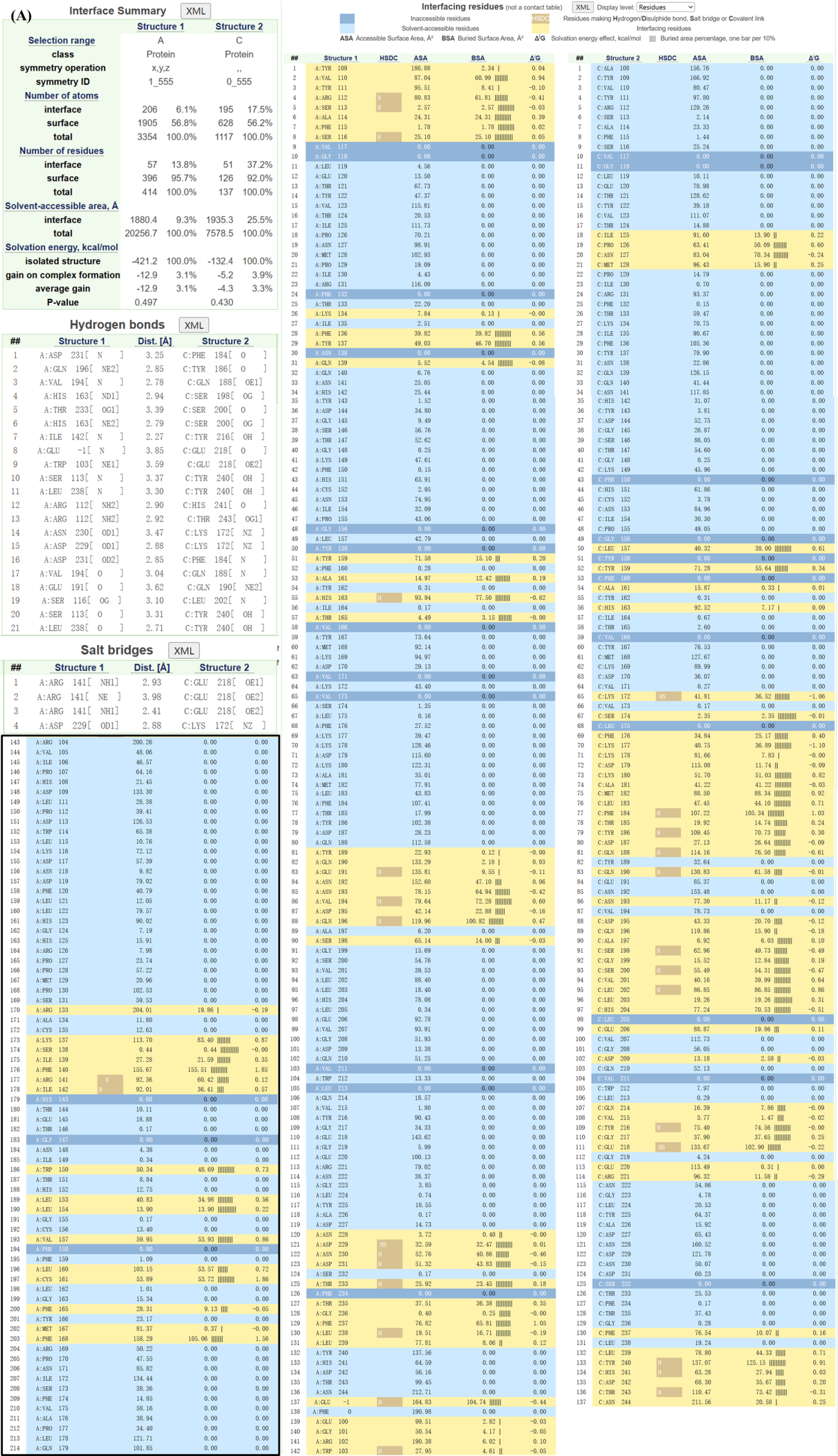
**

**
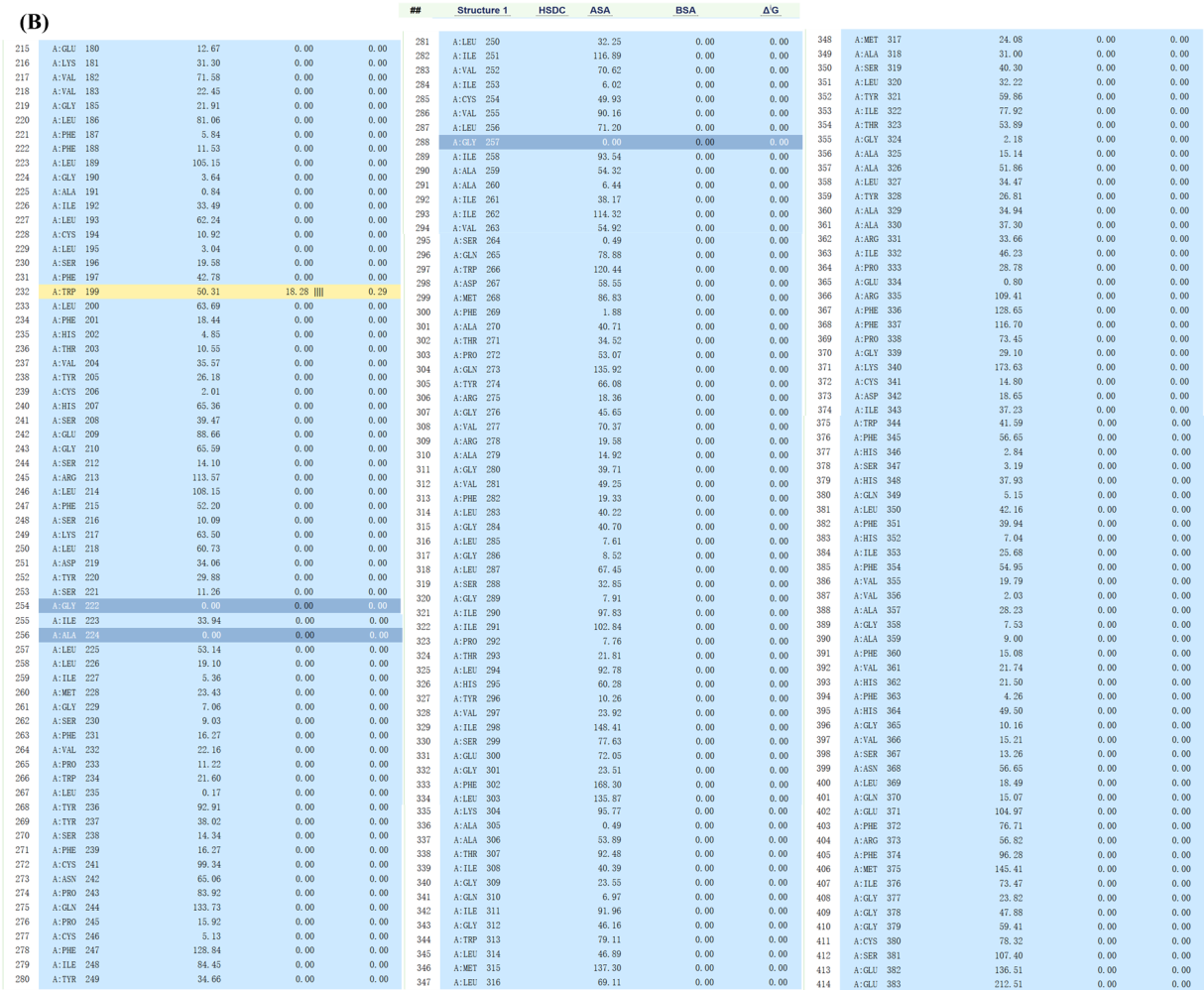
**

**Figure S10.** Molecular docking parameters of APN (6U66) + AdipoR2 (6KS1)

(B) is continuation of (A). ZDOCK is used for docking and PDBePISA is applied to analyze docking results. Available (November 2024): https://zdock.wenglab.org/; https://www.ebi.ac.uk/msd-srv/prot_int/


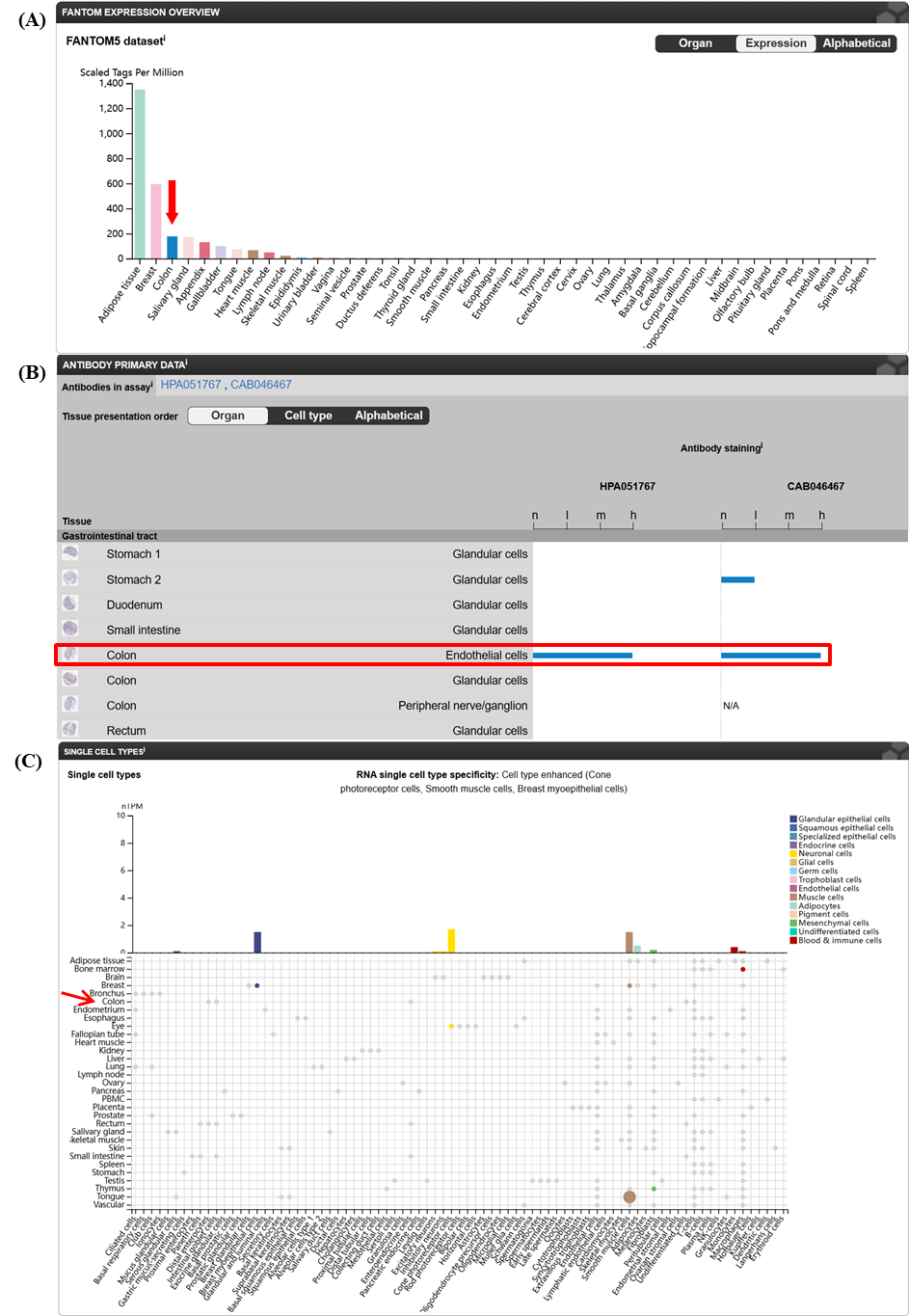


**Figure S11.** Expression of adiponectin in gastrointestinal tract

Available (November 2024): https://www.proteinatlas.org/


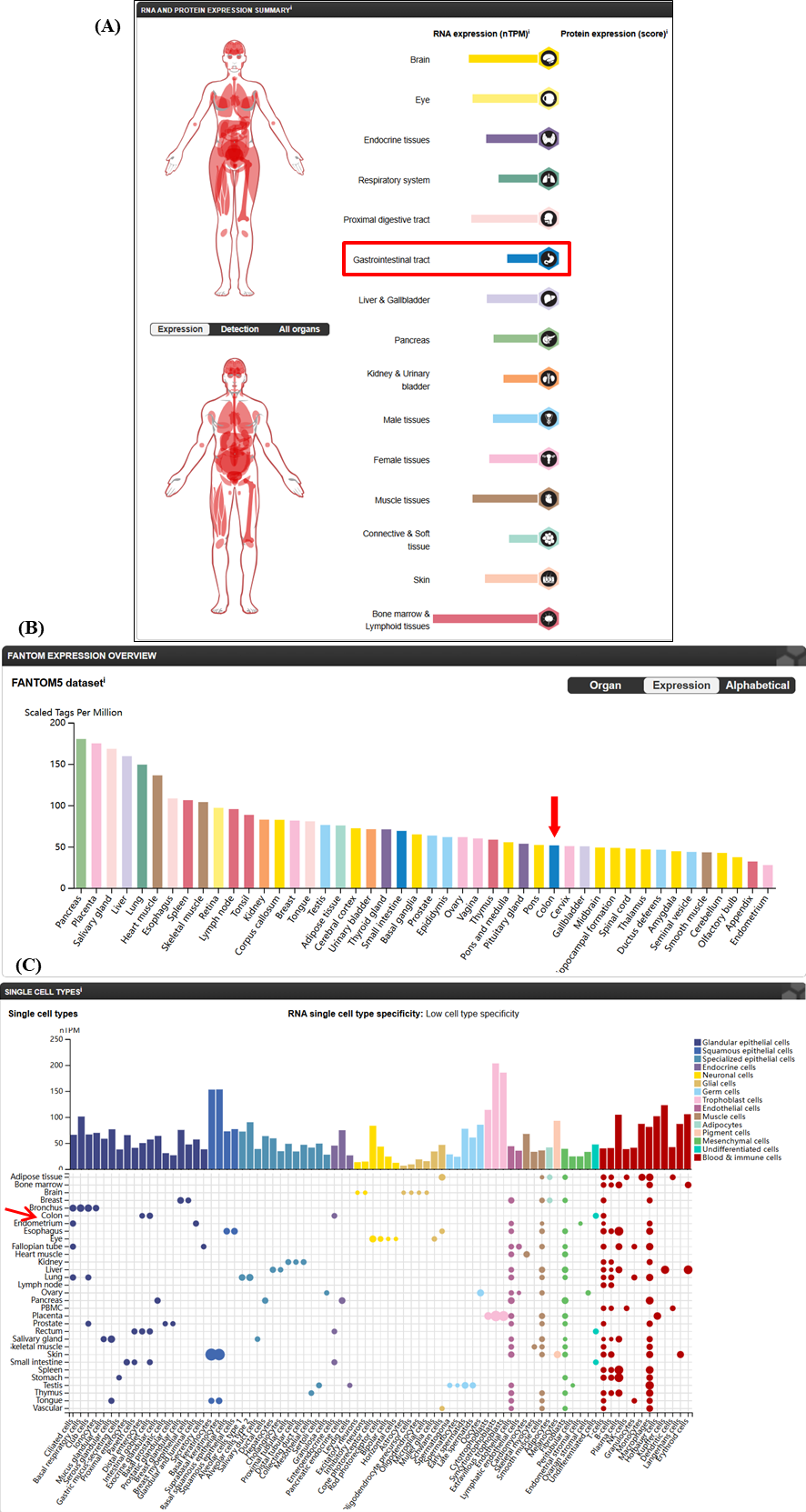


**Figure S12.** Expression of AdipoR1 in human tissue

Available (November 2024): https://www.proteinatlas.org/


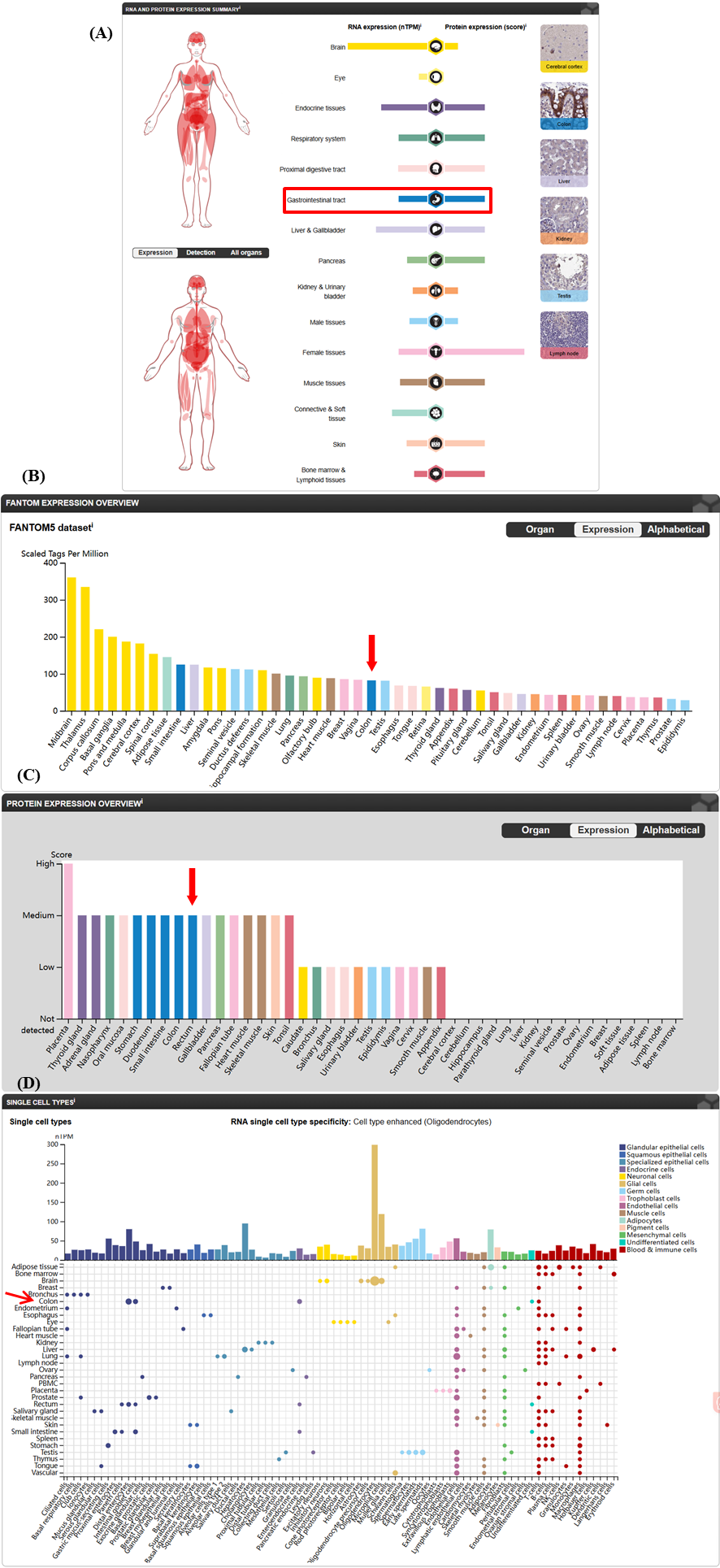


**Figure S13.** Expression of AdipoR2 in human tissue

Available (November 2024): https://www.proteinatlas.org/

**
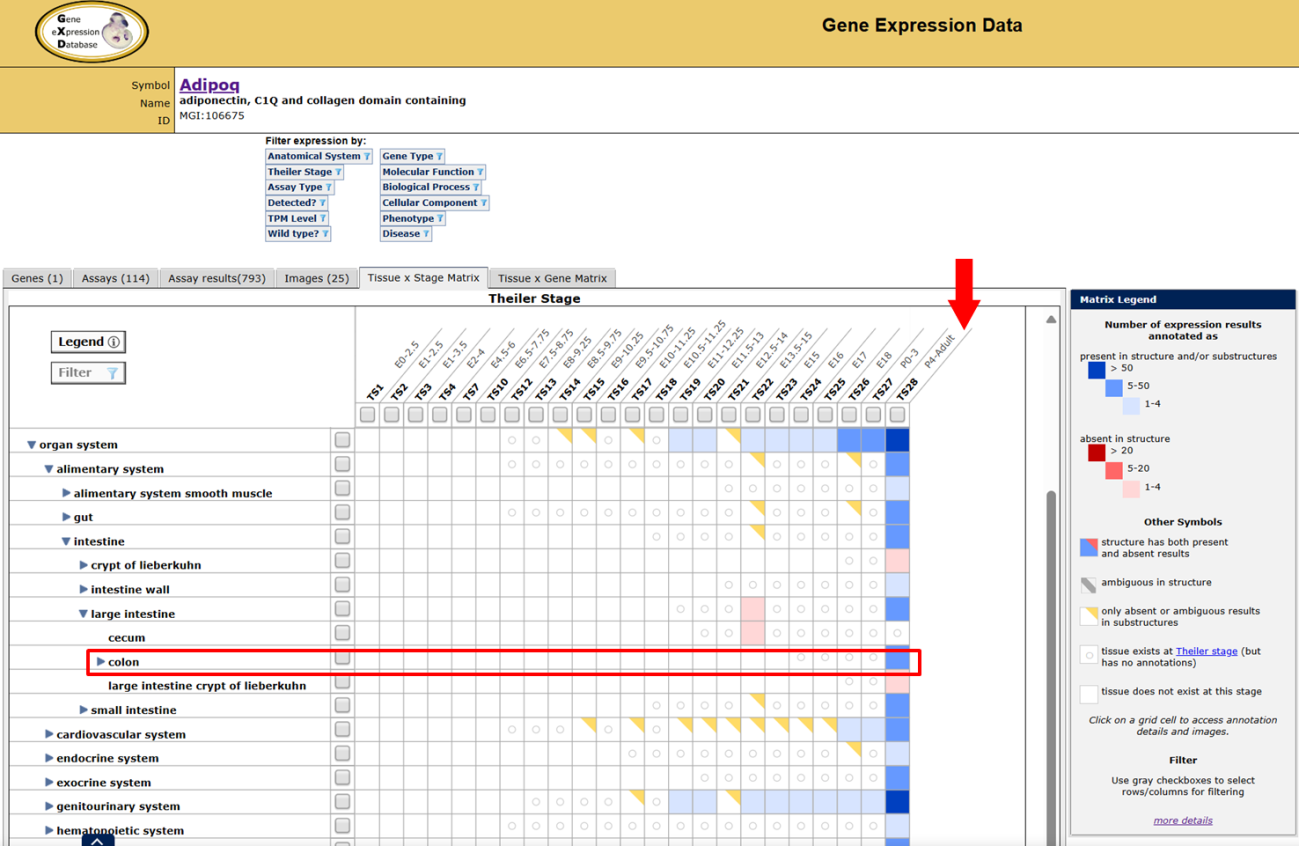
**

**Figure S14.** Expression of adiponectin in mouse colon

Available (November 2024): https://www.informatics.jax.org/

**
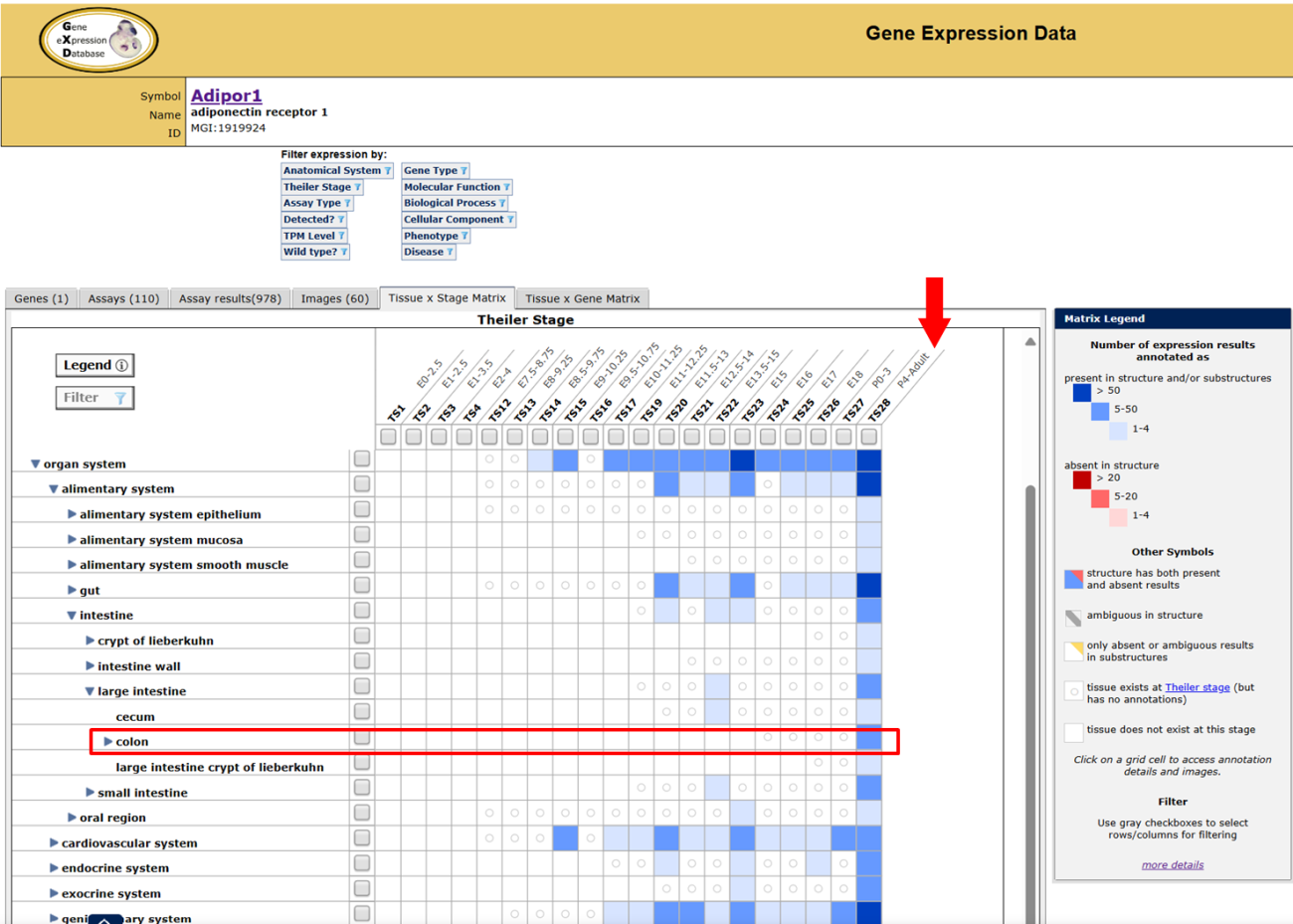
**

**Figure S15.** Expression of AdipoR1 in mouse colon

Available (November 2024): https://www.informatics.jax.org/

**
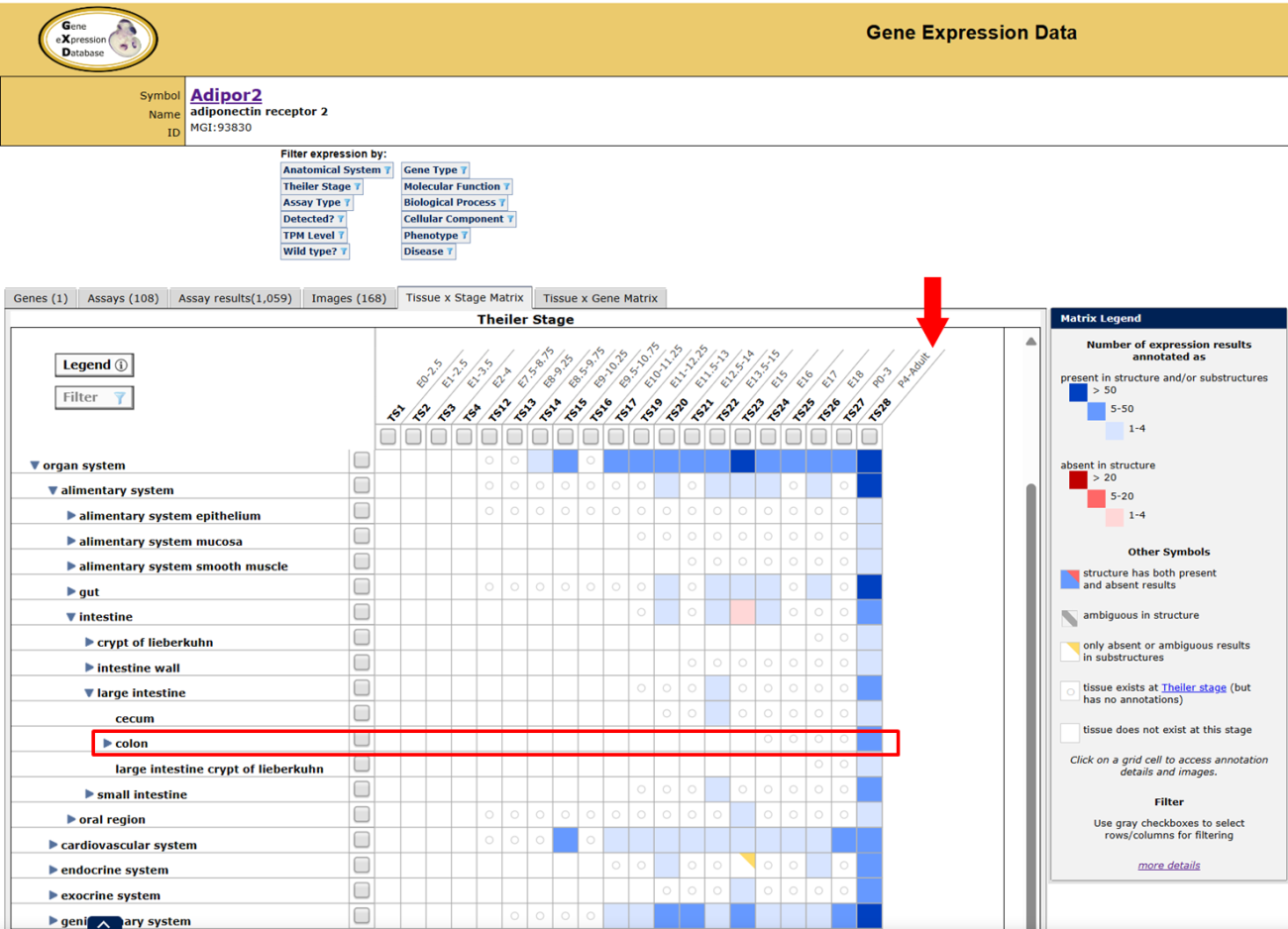
**

**Figure S16.** Expression of AdipoR2 in mouse colon

Available (November 2024): https://www.informatics.jax.org/

**
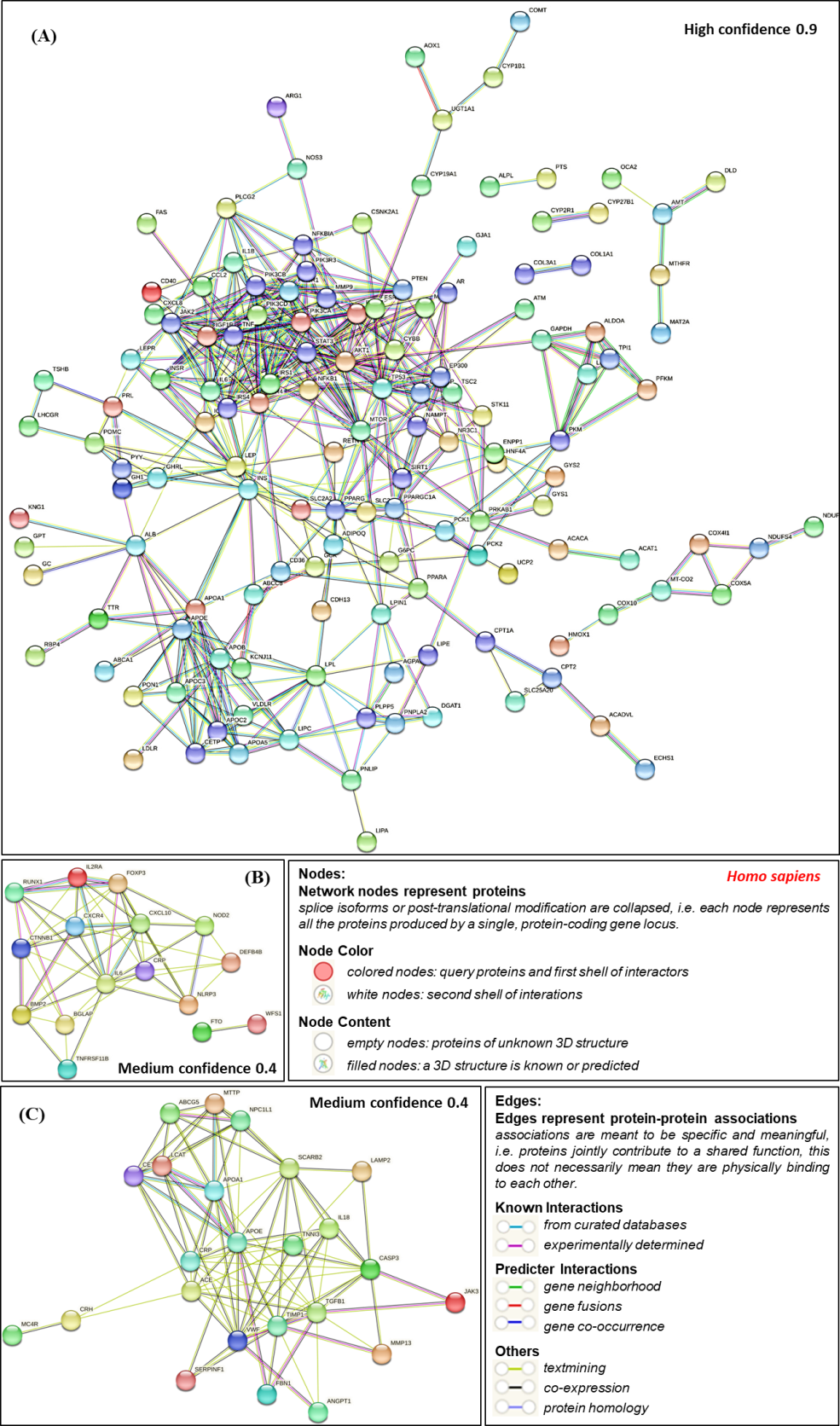
**

**Figure S17.** Interaction network of Homo sapiens APN+AdipoR1+AdipoR2+IBD (A), APN+AdipoR1+IBD (B) and APN+ AdipoR2+IBD (C)

The APN+AdipoR1+AdipoR2+IBD, APN+AdipoR1+IBD and APN+ AdipoR2+IBD targets with APN relevance score≥10 are selected for interaction network analysis by STRING database. APN: adiponectin; AdipoR1: adiponectin receptor 1; AdipoR2: adiponectin receptor 2; IBD: inflammatory bowel disease. Available (November 2024): https://cn.string-db.org/cgi/input?sessionId=bBSQf0gz7Y6u&input_page_show_search=on


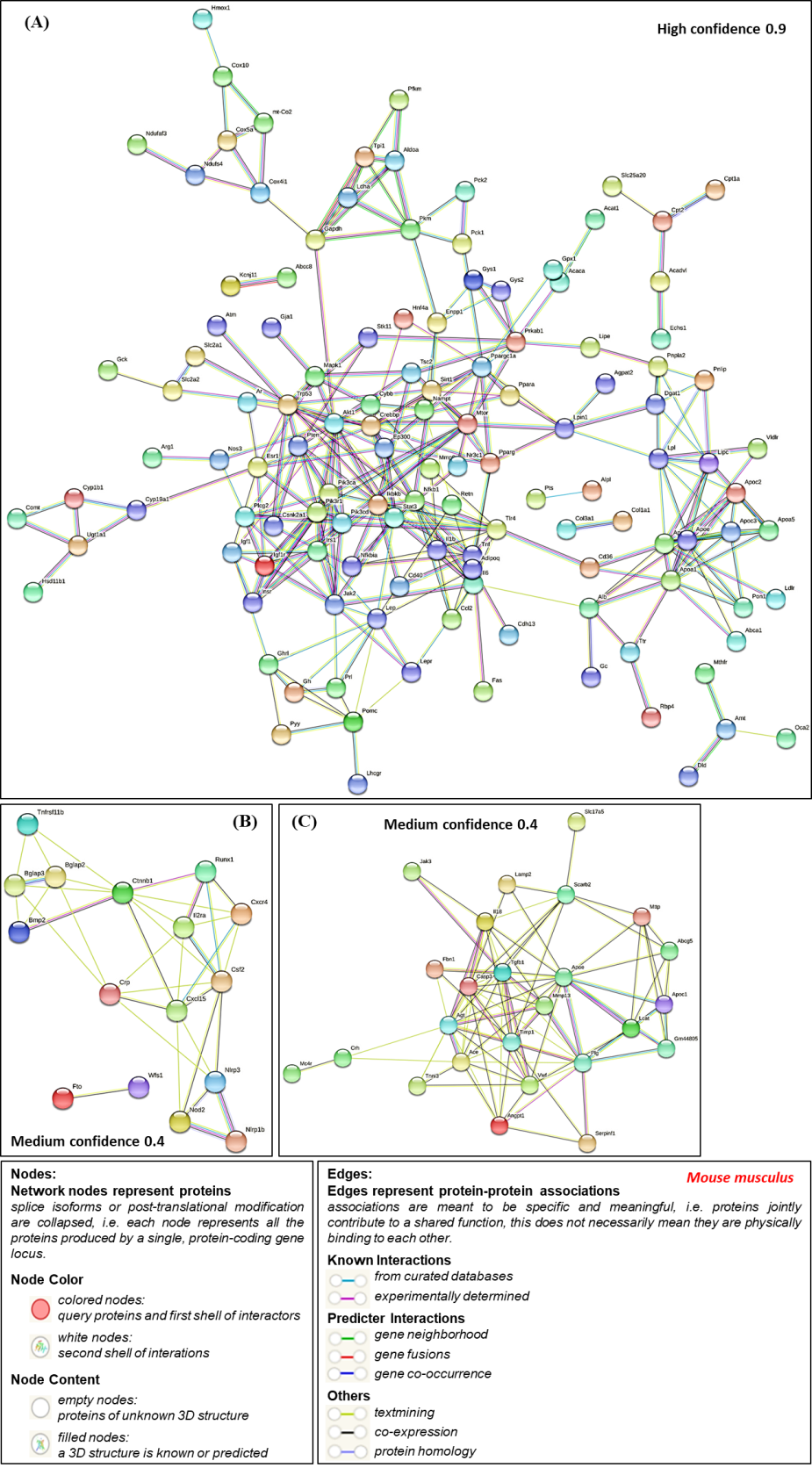


**Figure S18.** Interaction network of mouse musculus APN+AdipoR1+AdipoR2+IBD (A), APN+AdipoR1+IBD (B) and APN+ AdipoR2+IBD (C)

The APN+AdipoR1+AdipoR2+IBD, APN+AdipoR1+IBD and APN+ AdipoR2+IBD with APN relevance score≥10 are selected for interaction network analysis by STRING database. APN: adiponectin; AdipoR1: adiponectin receptor 1; AdipoR2: adiponectin receptor 2; IBD: inflammatory bowel disease. Available (November 2024): https://cn.string-db.org/cgi/input?sessionId=bBSQf0gz7Y6u&input_page_show_search=on

**
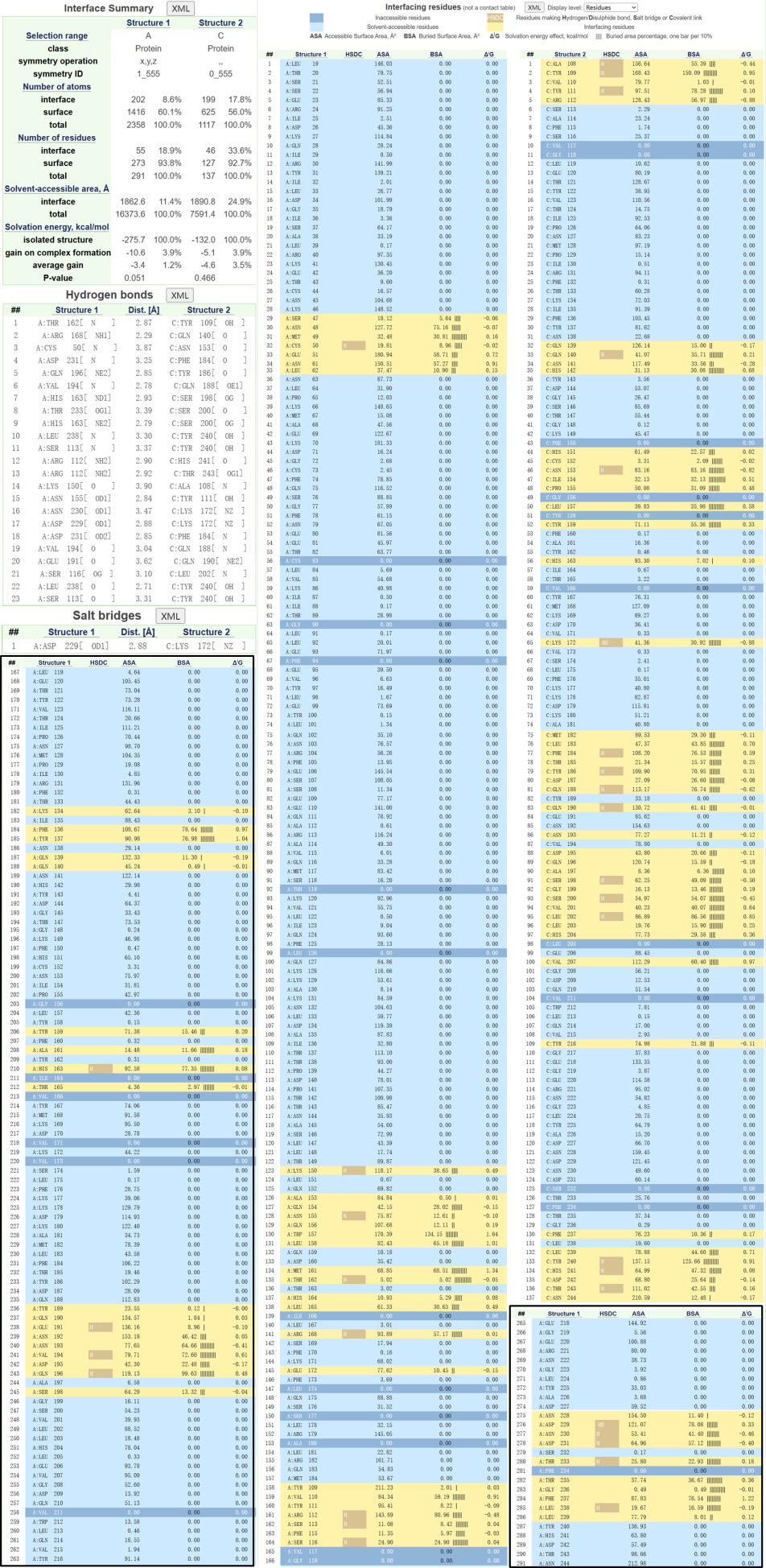
**

**Figure S19.** Molecular docking parameters of APN (6U66) + IL-6 (1ALU)

ZDOCK is used for docking and PDBePISA is applied to analyze docking results. Available (November 2024): https://zdock.wenglab.org/; https://www.ebi.ac.uk/msd-srv/prot_int/

**
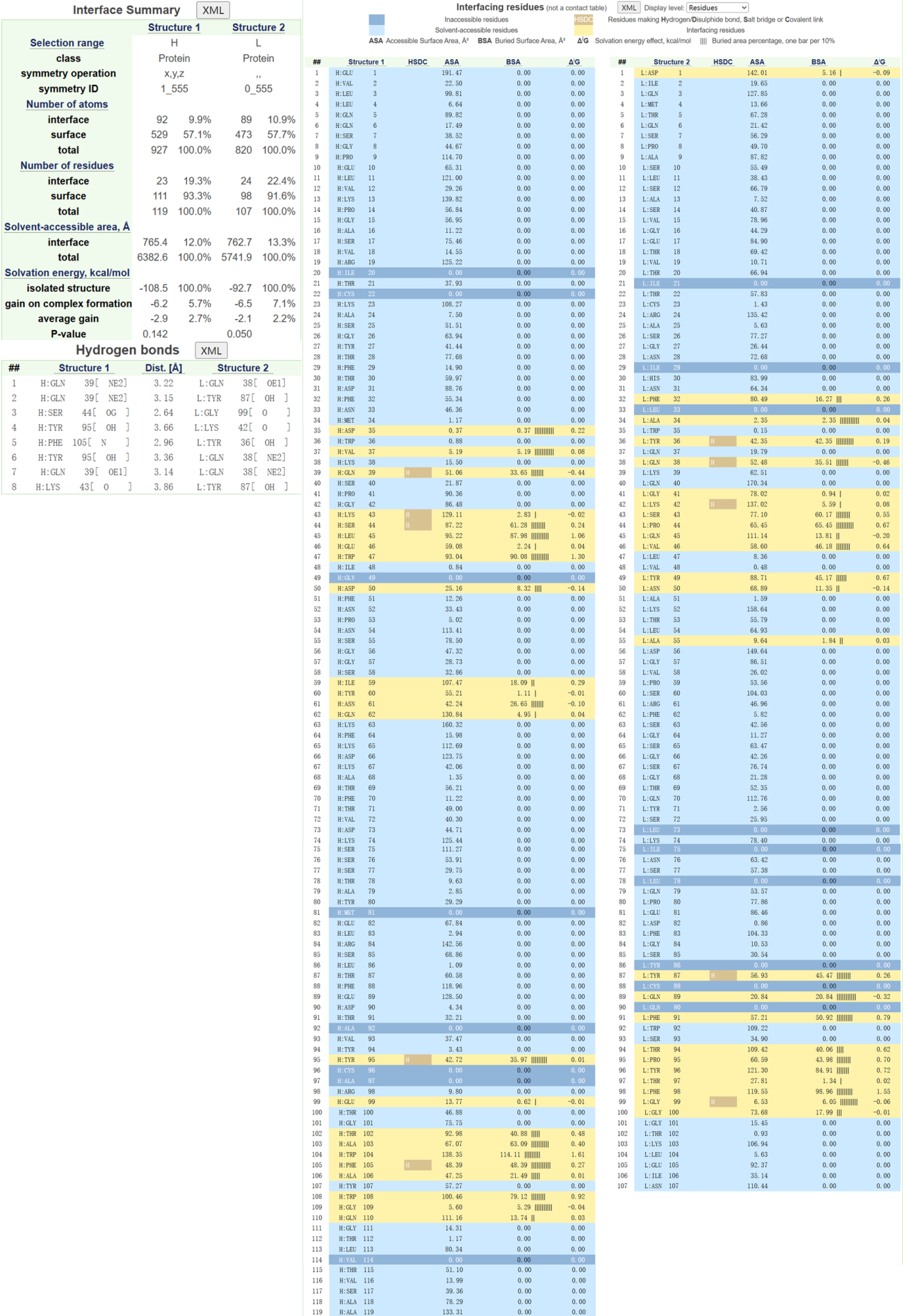
**

**Figure S20.** Molecular docking parameters of AdipoR1 (5LXG) + IL-6 (1ALU)

ZDOCK is used for docking and PDBePISA is applied to analyze docking results. Available (November 2024): https://zdock.wenglab.org/; https://www.ebi.ac.uk/msd-srv/prot_int/

**
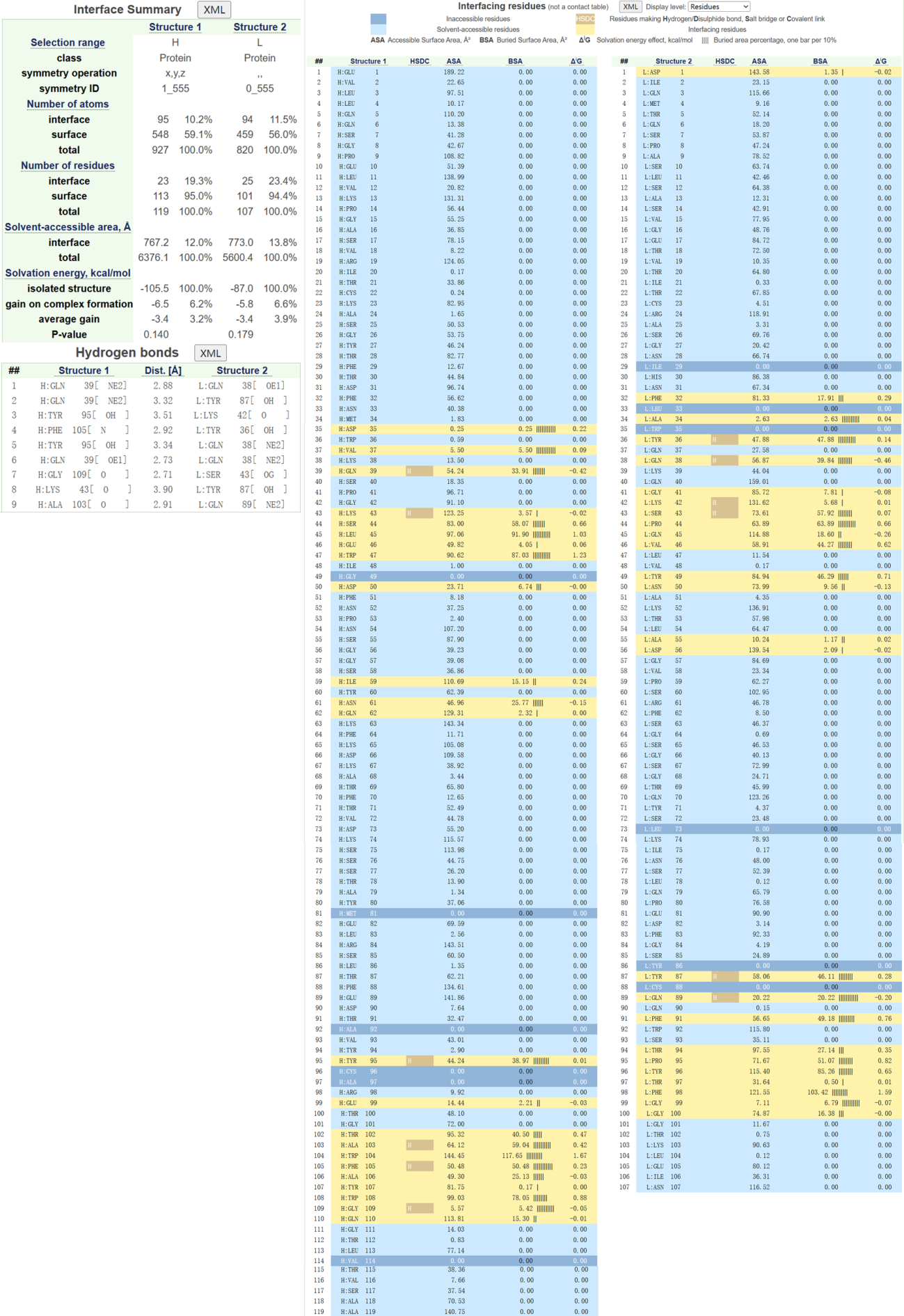
**

**Figure S21.** Molecular docking parameters of AdipoR2 (6KS1) + IL-6 (1ALU)

ZDOCK is used for docking and PDBePISA is applied to analyze docking results. Available (November 2024): https://zdock.wenglab.org/; https://www.ebi.ac.uk/msd-srv/prot_int/

**
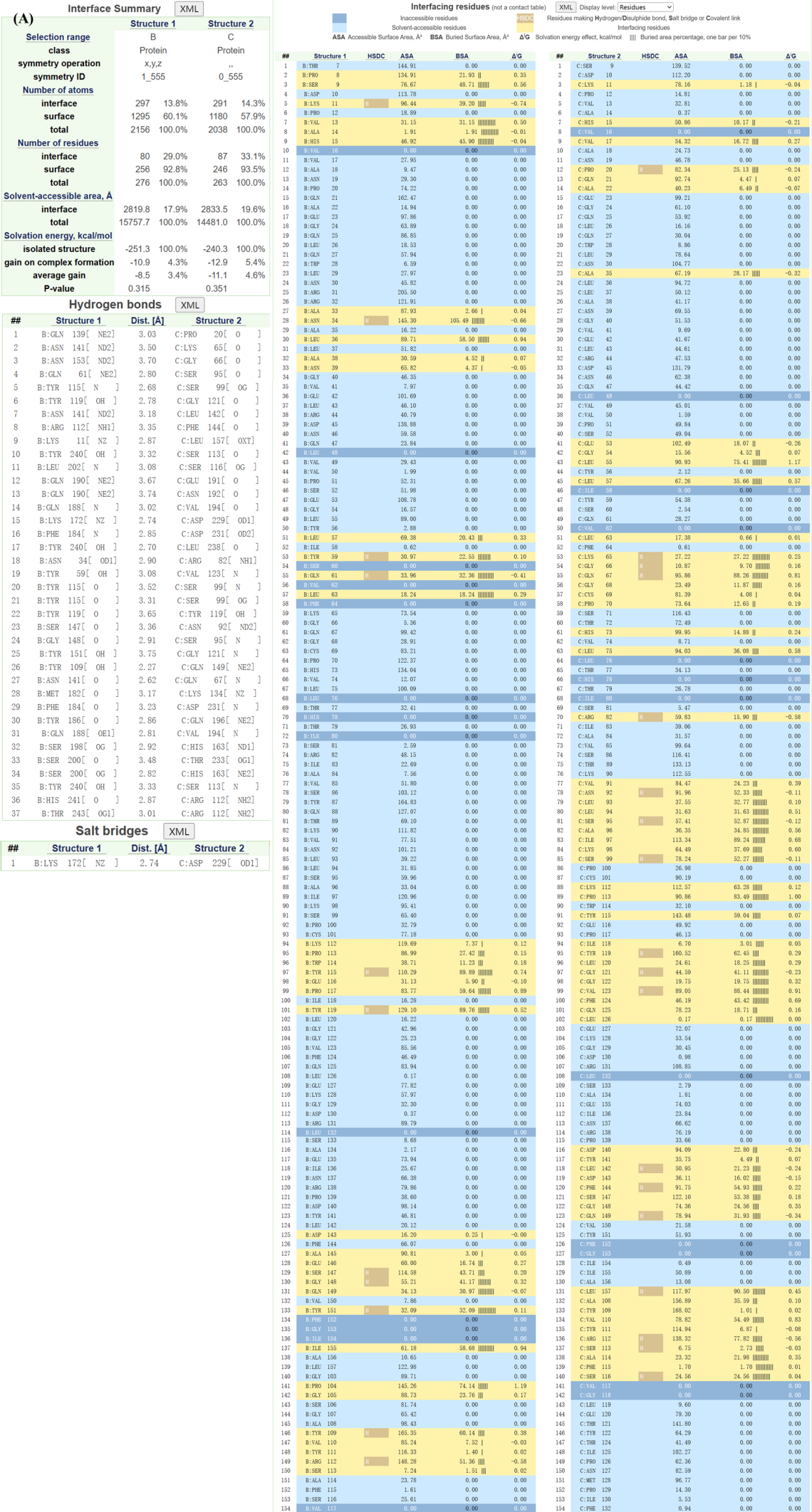
**

**
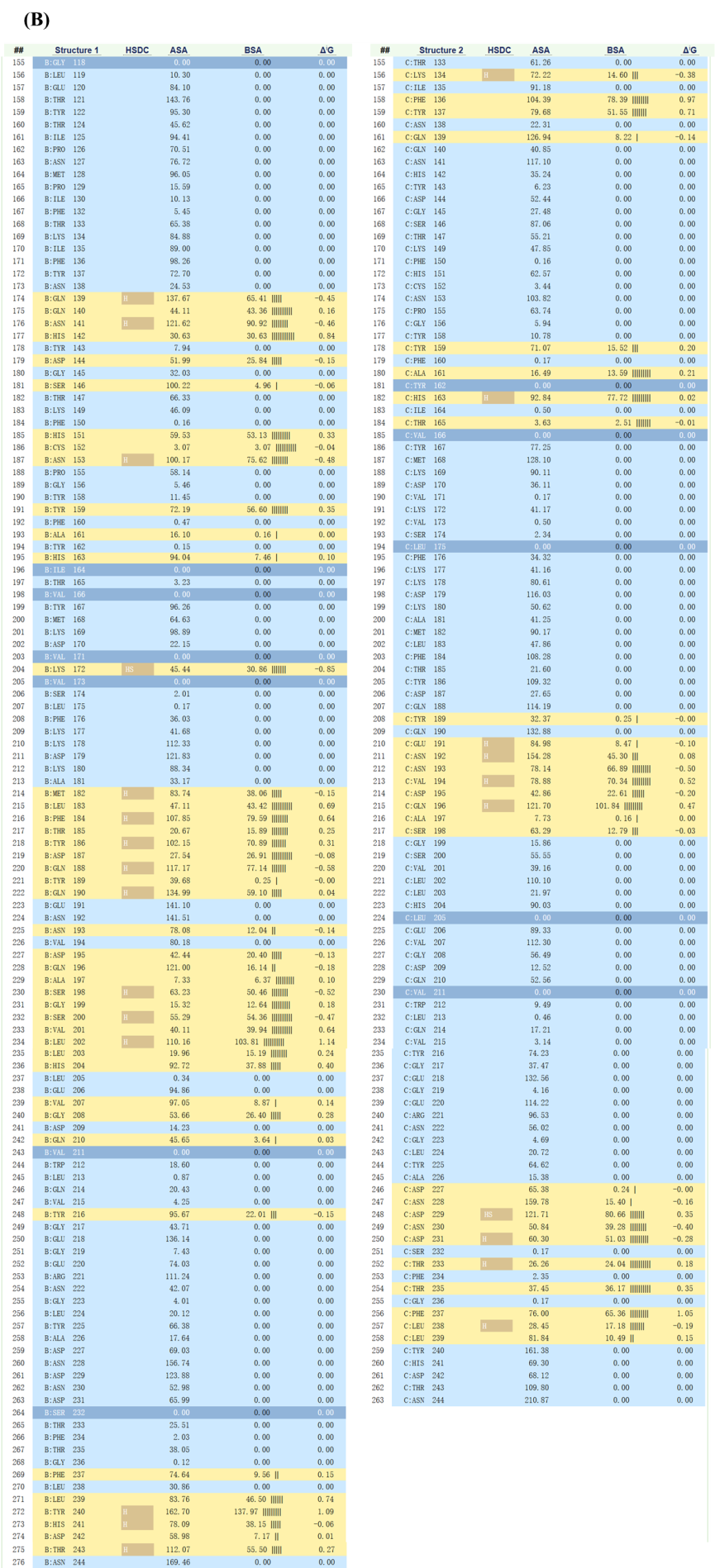
**

**Figure S22.** Molecular docking parameters of APN (6U66) + TNF-α (7KP9)

(B) is continuation of (A). ZDOCK is used for docking and PDBePISA is applied to analyze docking results. Available (November 2024): https://zdock.wenglab.org/; https://www.ebi.ac.uk/msd-srv/prot_int/

**
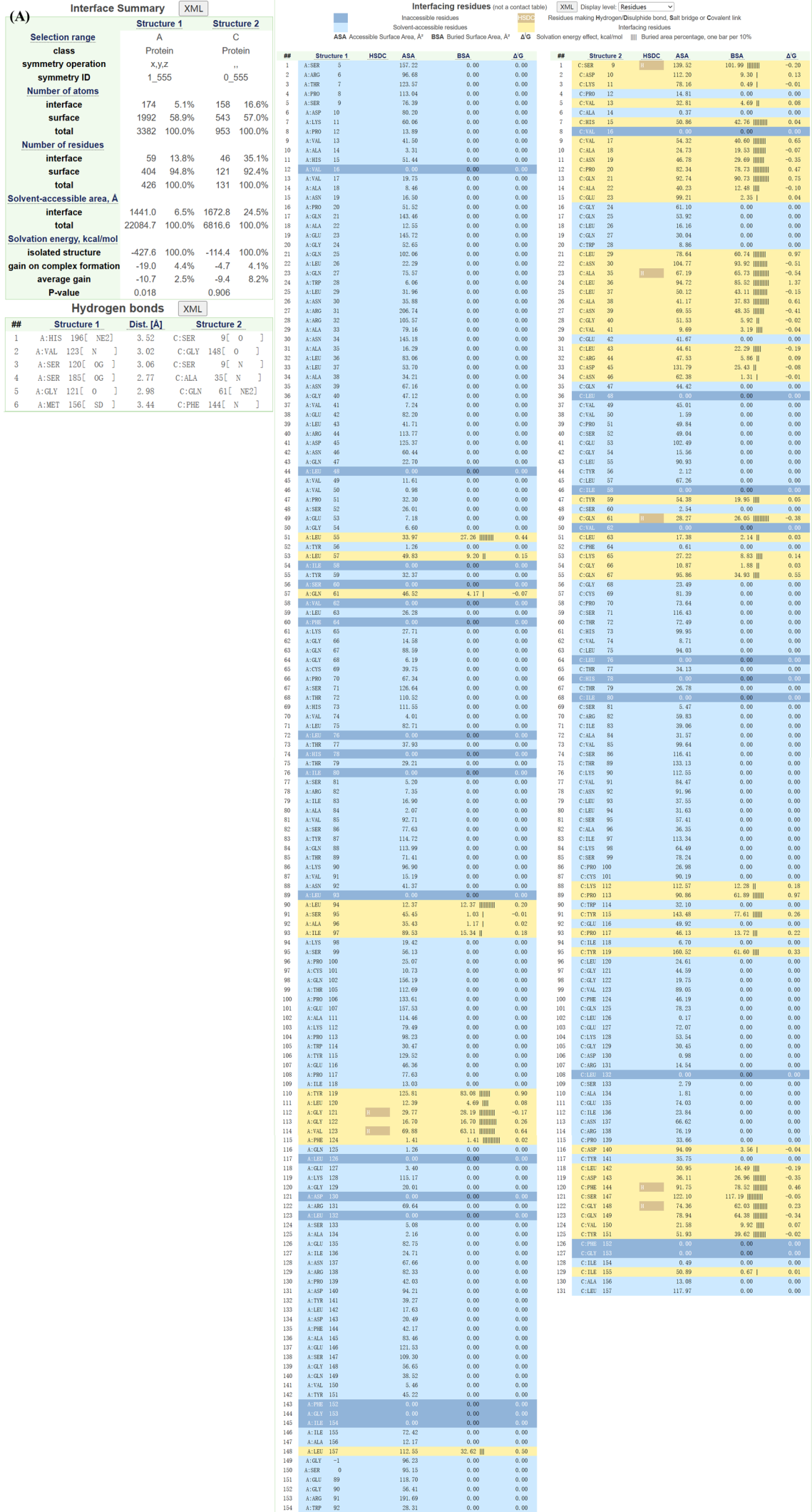
**

**
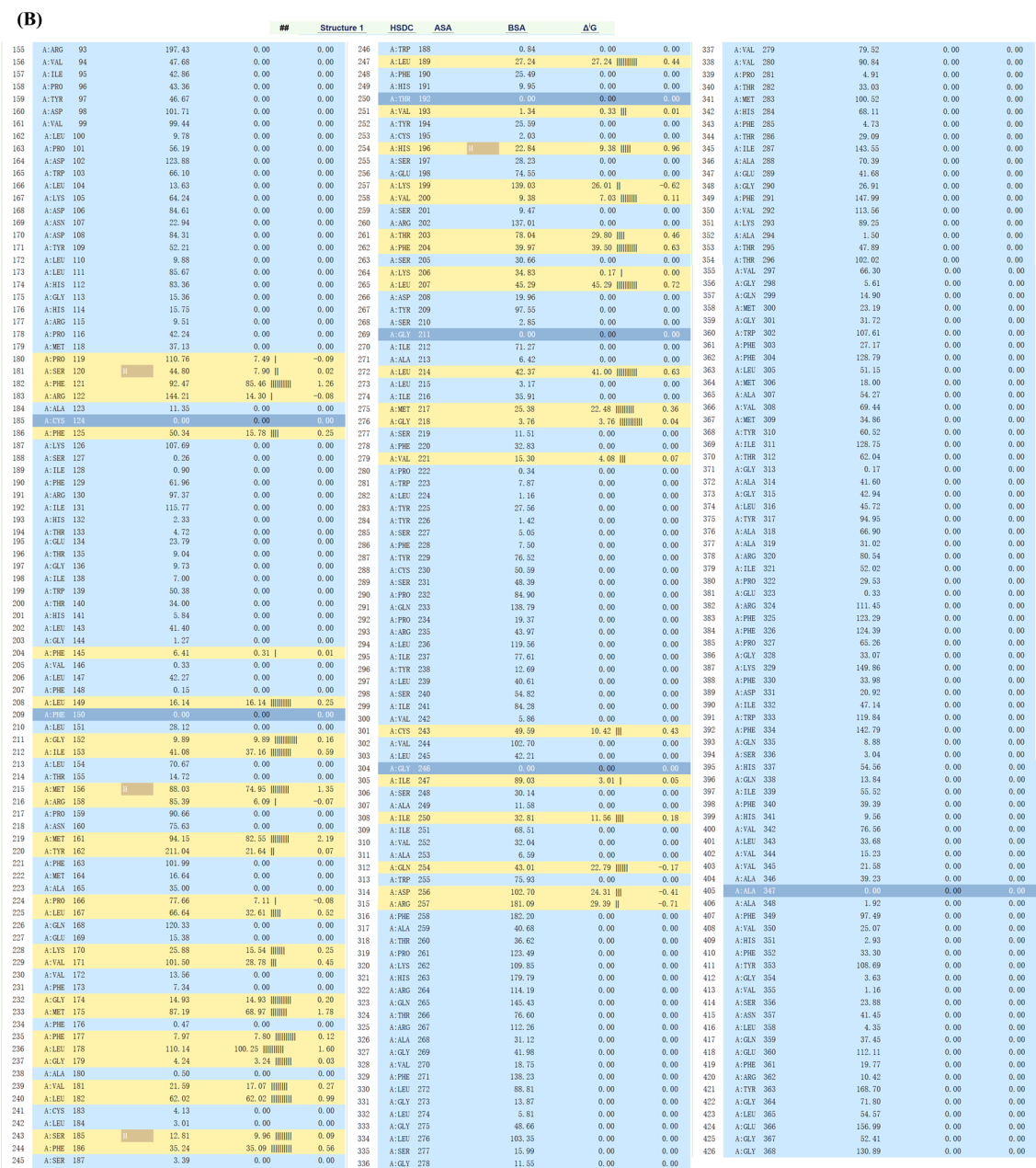
**

**Figure S23.** Molecular docking parameters of AdipoR1 (5LXG) + TNF-α (7KP9)

(B) is continuation of (A). ZDOCK is used for docking and PDBePISA is applied to analyze docking results. Available (November 2024): https://zdock.wenglab.org/; https://www.ebi.ac.uk/msd-srv/prot_int/

**
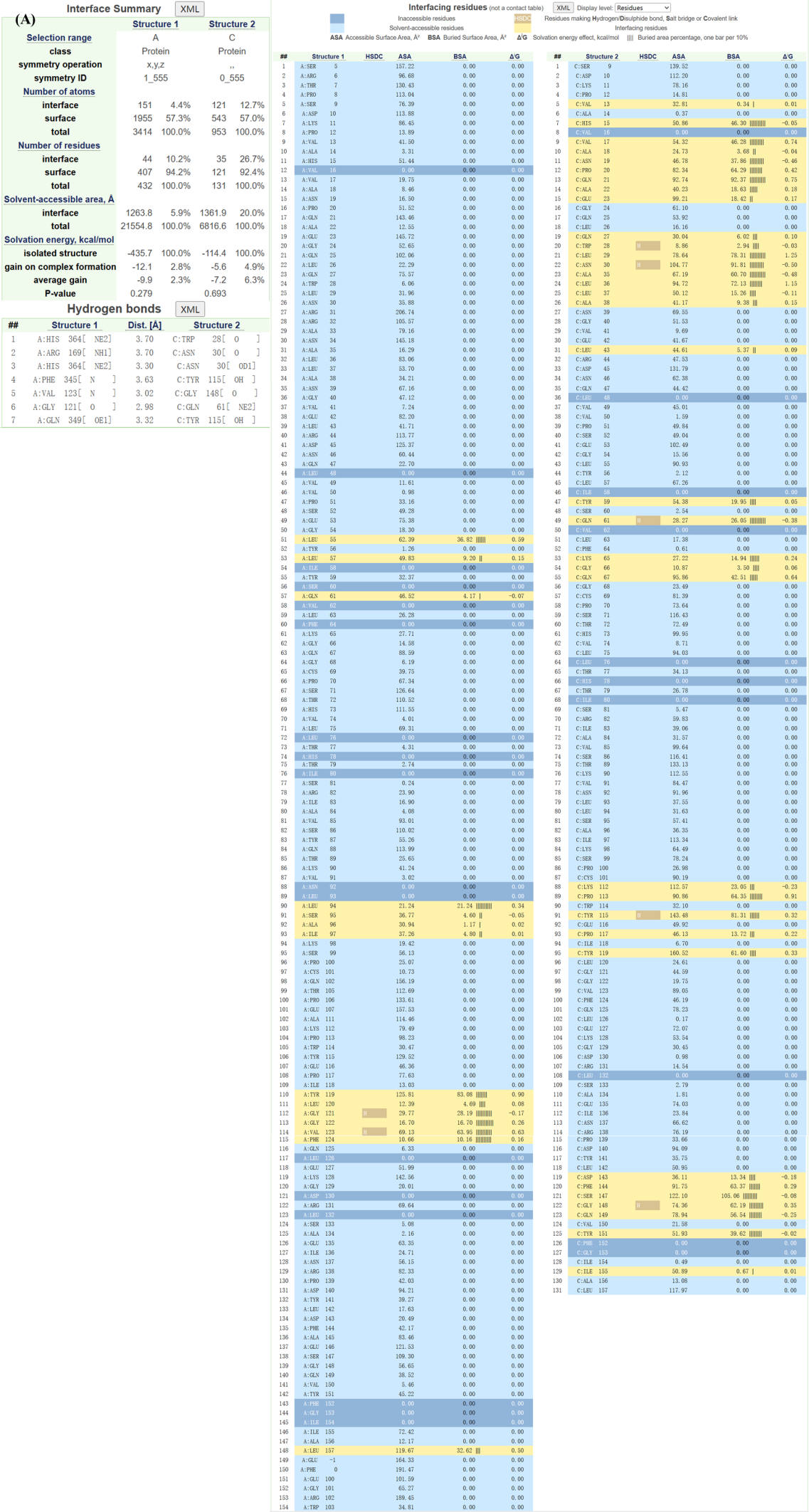
**

**
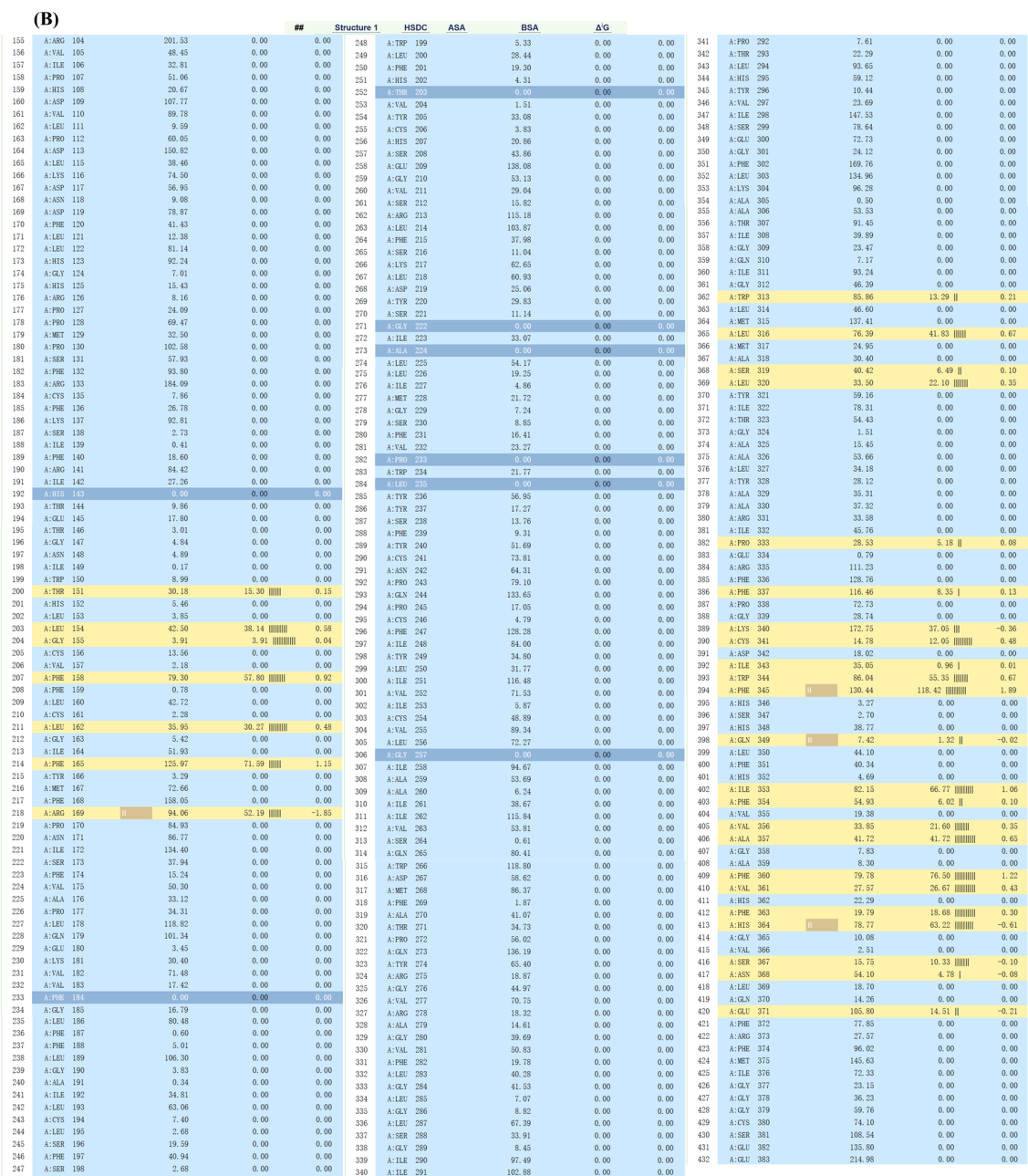
**

**Figure S24.** Molecular docking parameters of AdipoR2 (6KS1) + TNF-α (7KP9)

(B) is continuation of (A). ZDOCK is used for docking and PDBePISA is applied to analyze docking results. Available (November 2024): https://zdock.wenglab.org/; https://www.ebi.ac.uk/msd-srv/prot_int/

**
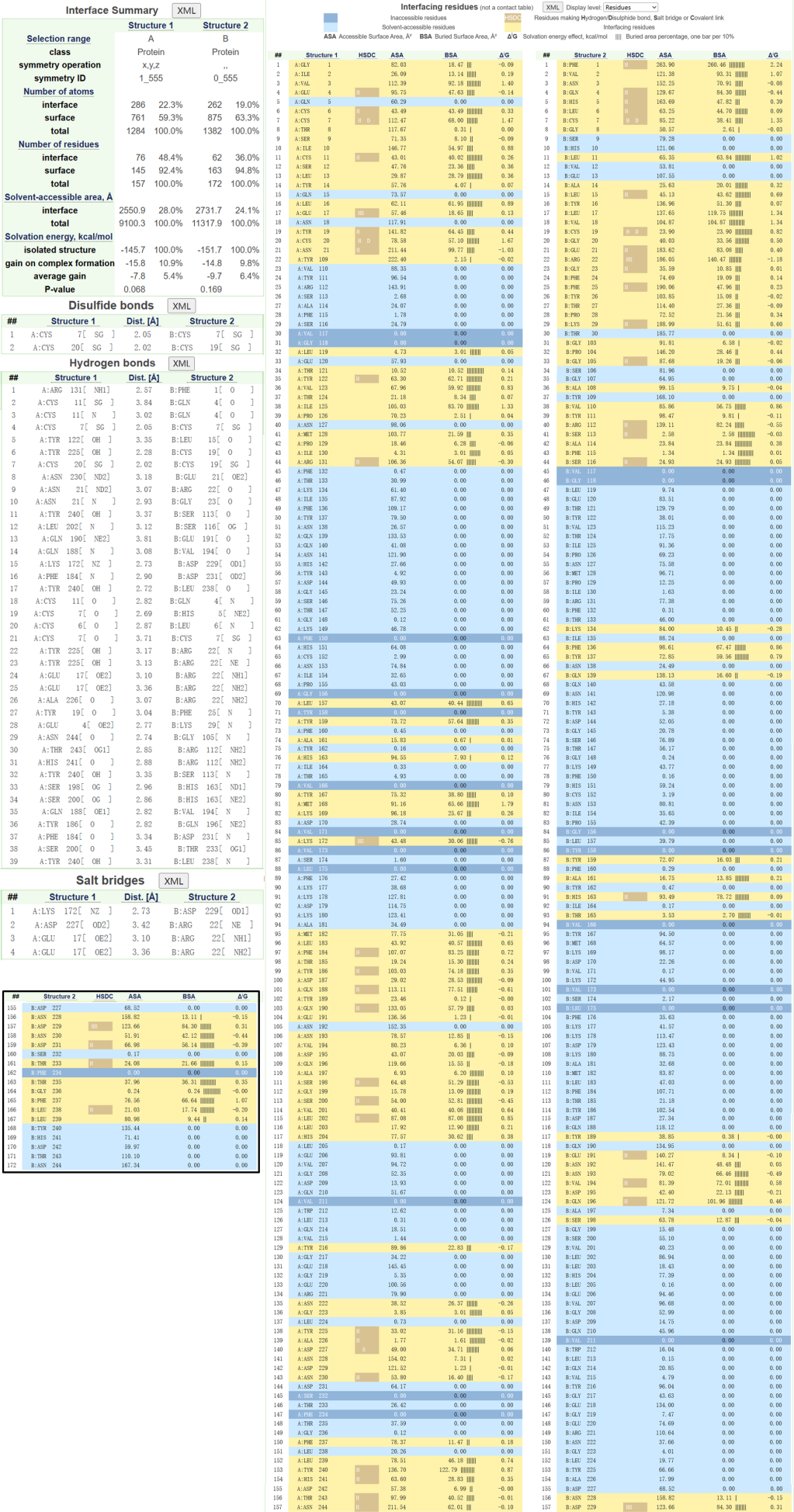
**

**Figure S25.** Molecular docking parameters of APN (6U66) + insulin (1MSO)

ZDOCK is used for docking and PDBePISA is applied to analyze docking results. Available (November 2024): https://zdock.wenglab.org/; https://www.ebi.ac.uk/msd-srv/prot_int/

**
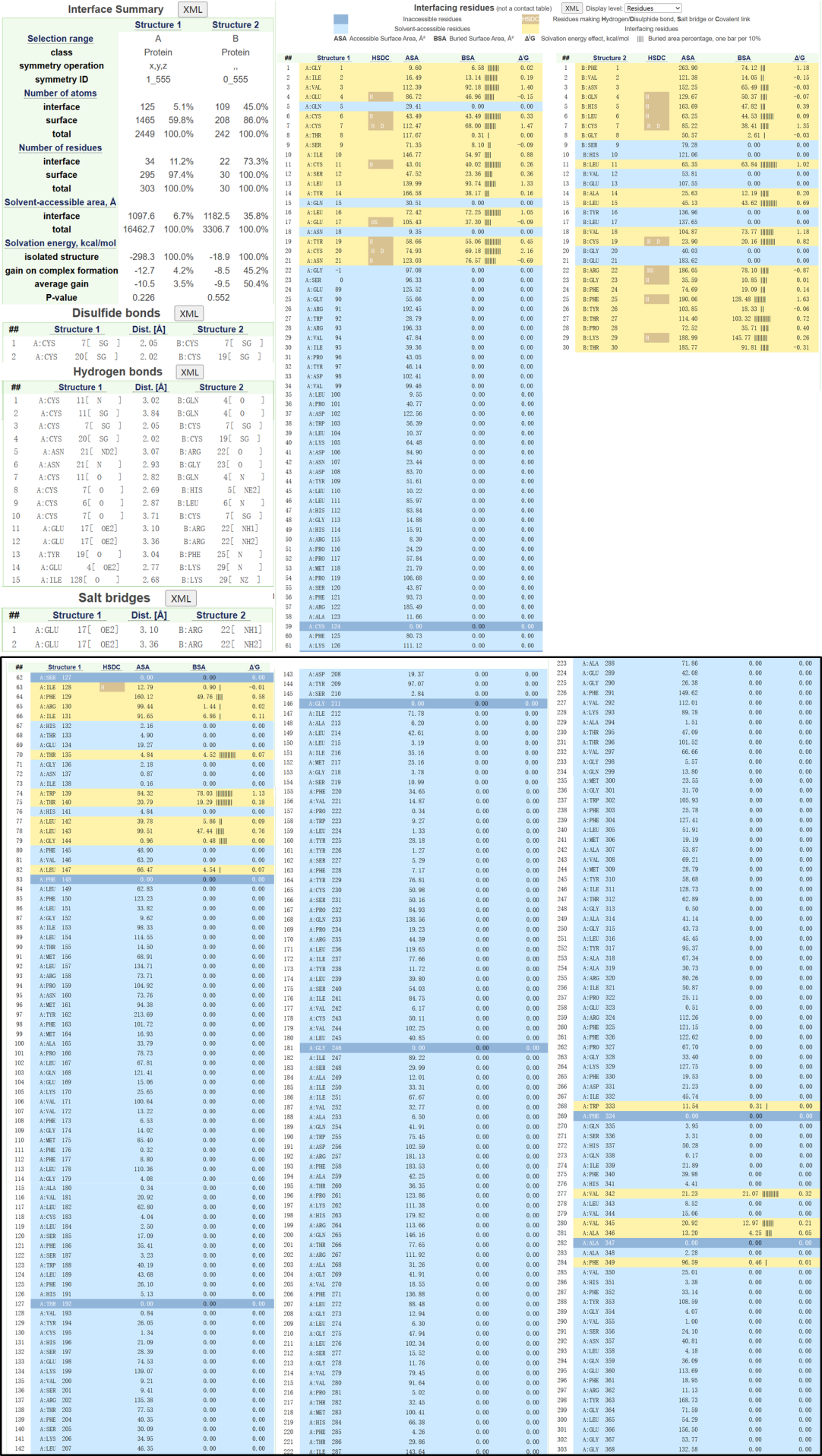
**

**Figure S26.** Molecular docking parameters of AdipoR1 (5LXG) + insulin (1MSO)

ZDOCK is used for docking and PDBePISA is applied to analyze docking results. Available (November 2024): https://zdock.wenglab.org/; https://www.ebi.ac.uk/msd-srv/prot_int/

**
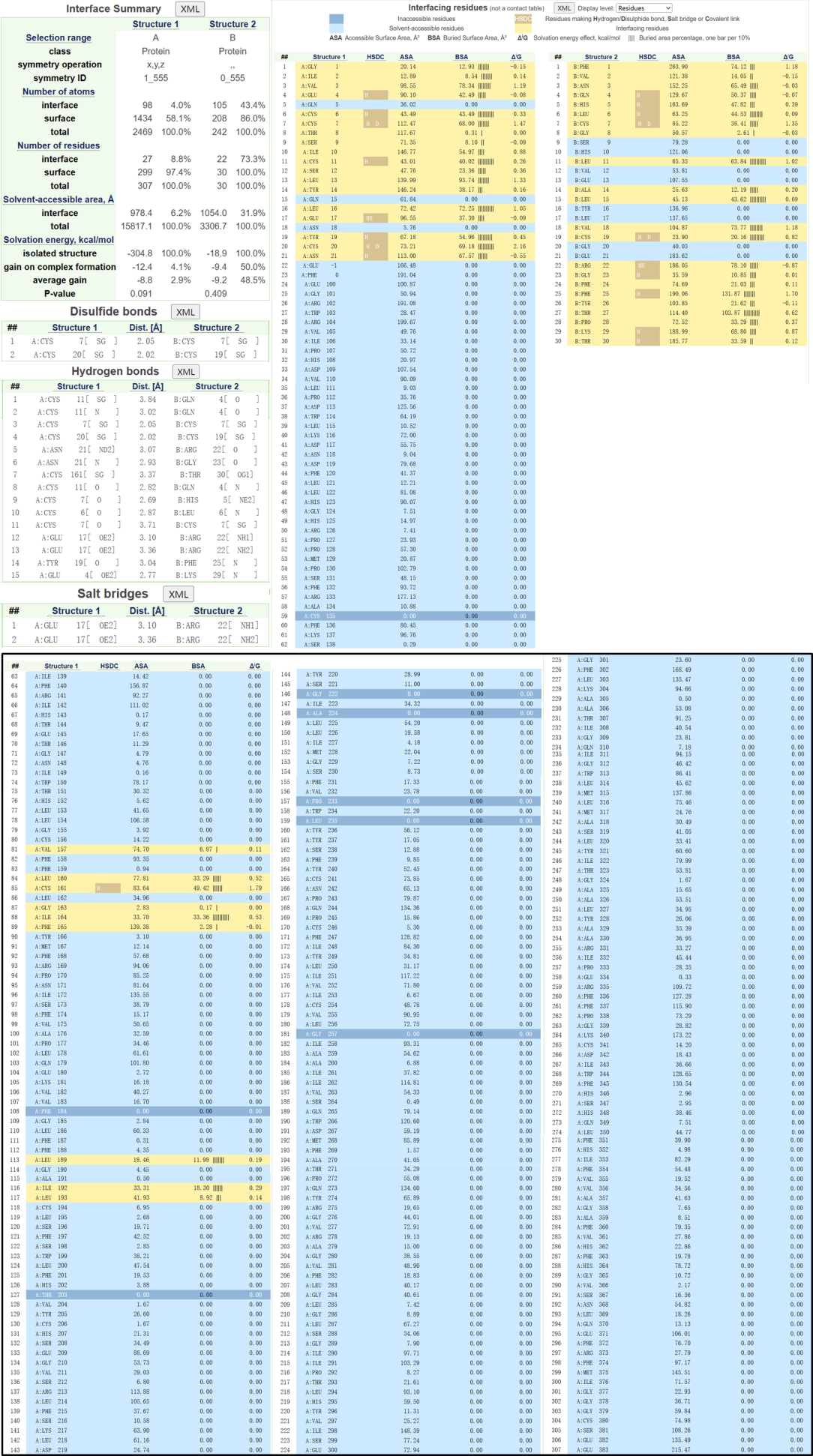
**

**Figure S27.** Molecular docking parameters of AdipoR2 (6KS1) + insulin (1MSO)

ZDOCK is used for docking and PDBePISA is applied to analyze docking results. Available (November 2024): https://zdock.wenglab.org/; https://www.ebi.ac.uk/msd-srv/prot_int/

**
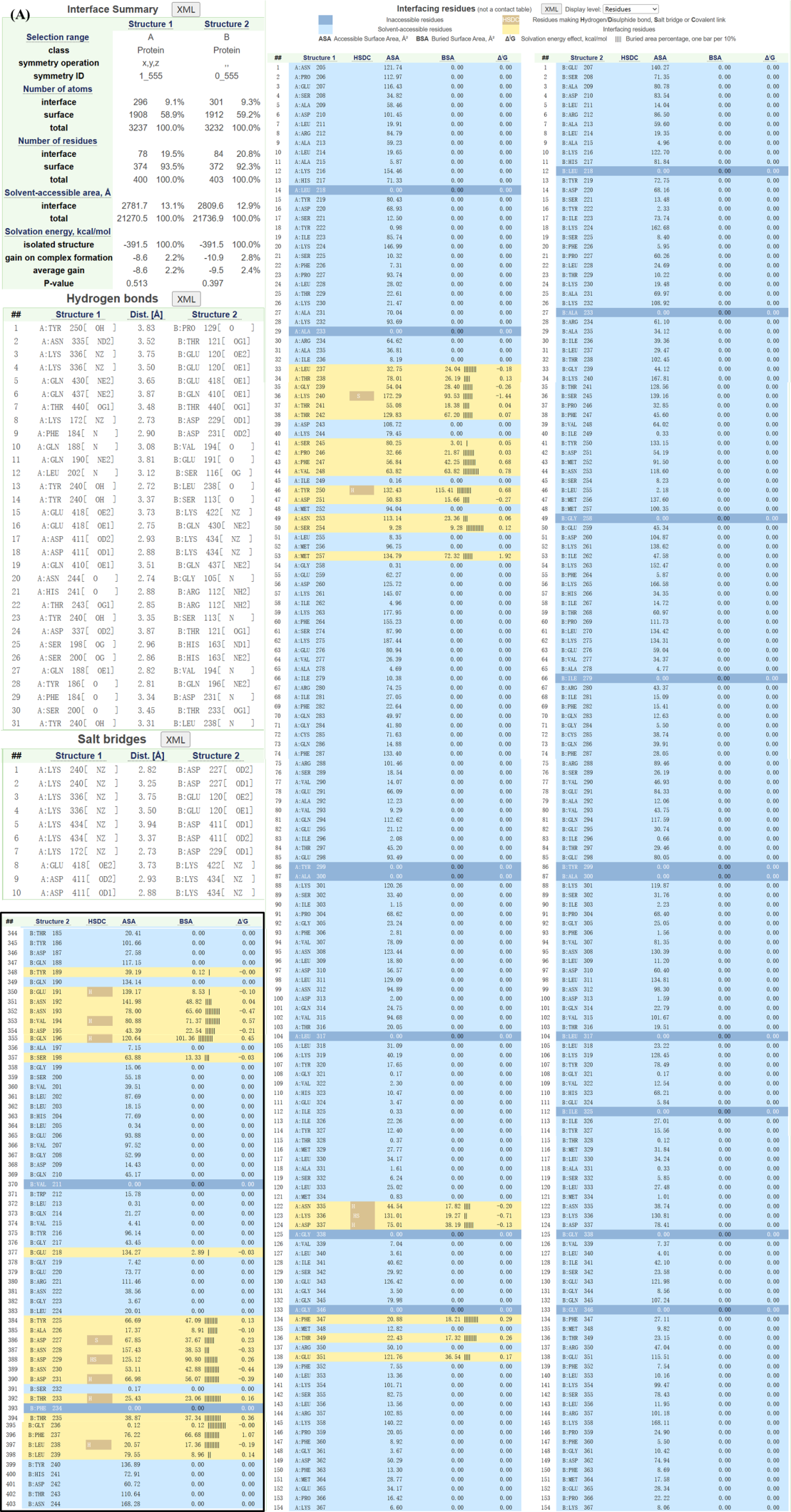
**

**
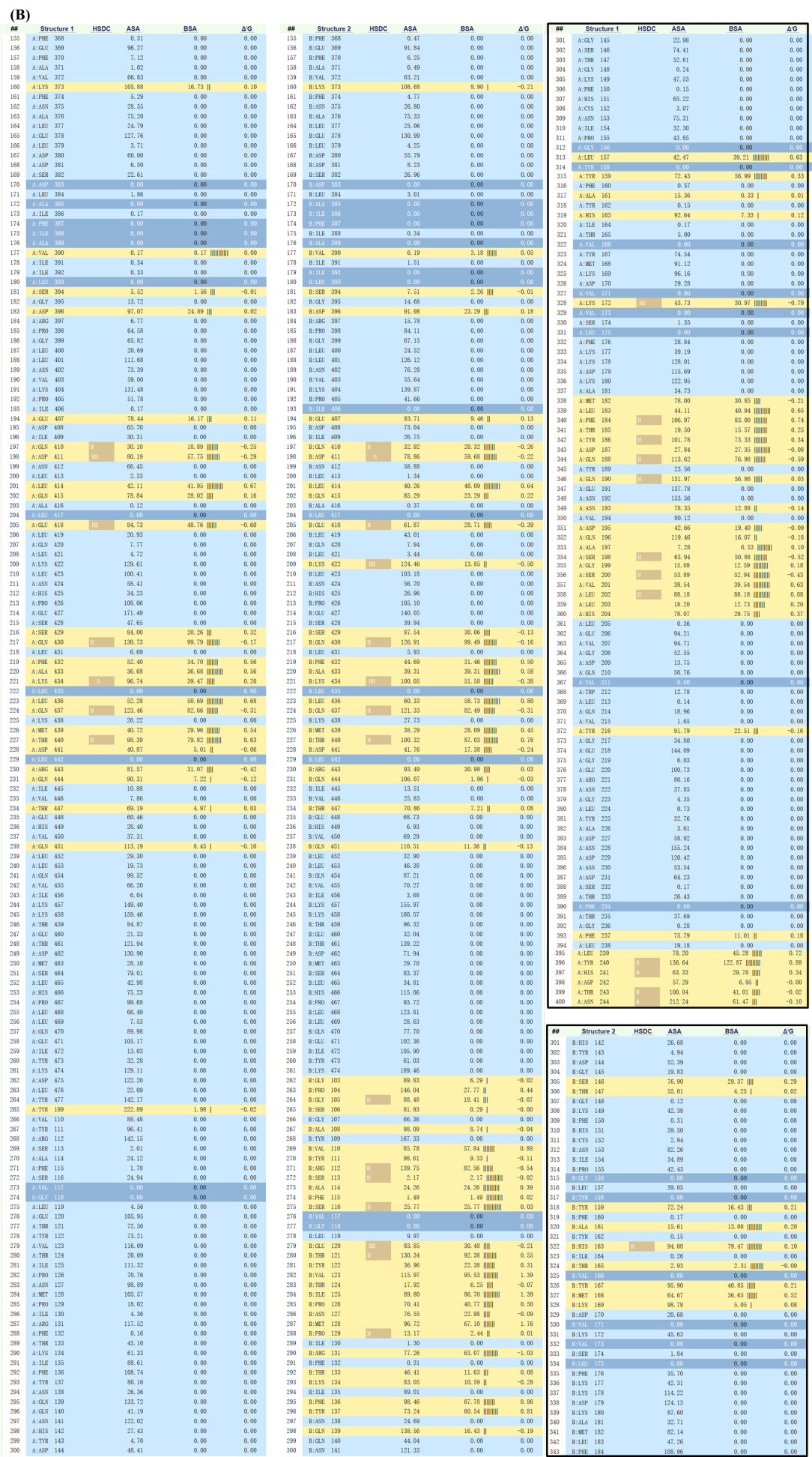
**

**Figure S28.** Molecular docking parameters of APN (6U66) + PPARG (6L8B)

(B) is continuation of (A). ZDOCK is used for docking and PDBePISA is applied to analyze docking results. Available (November 2024): https://zdock.wenglab.org/; https://www.ebi.ac.uk/msd-srv/prot_int/

**
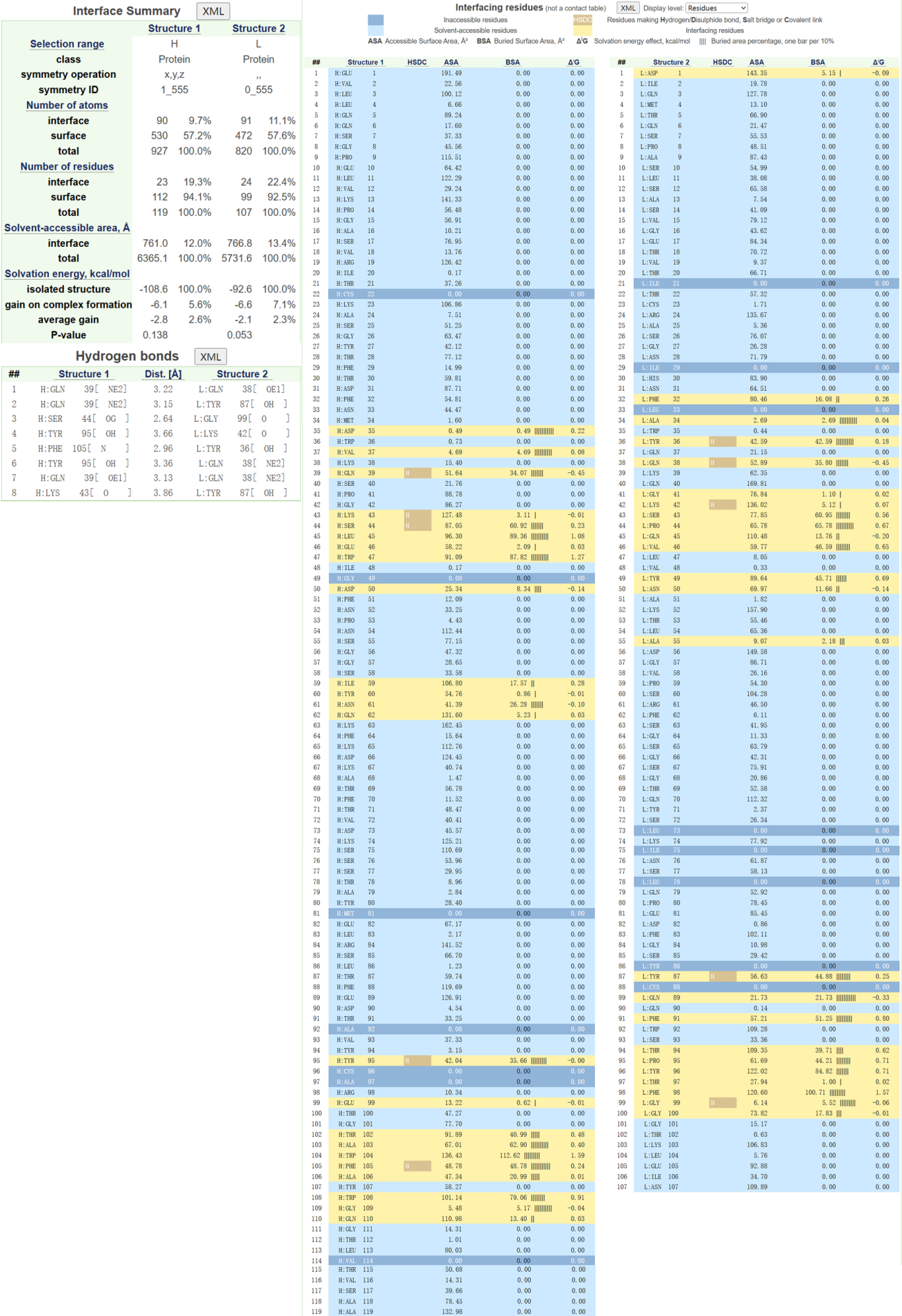
**

**Figure S29.** Molecular docking parameters of AdipoR1 (5LXG) + PPARG (6L8B)

ZDOCK is used for docking and PDBePISA is applied to analyze docking results. Available (November 2024): https://zdock.wenglab.org/; https://www.ebi.ac.uk/msd-srv/prot_int/

**
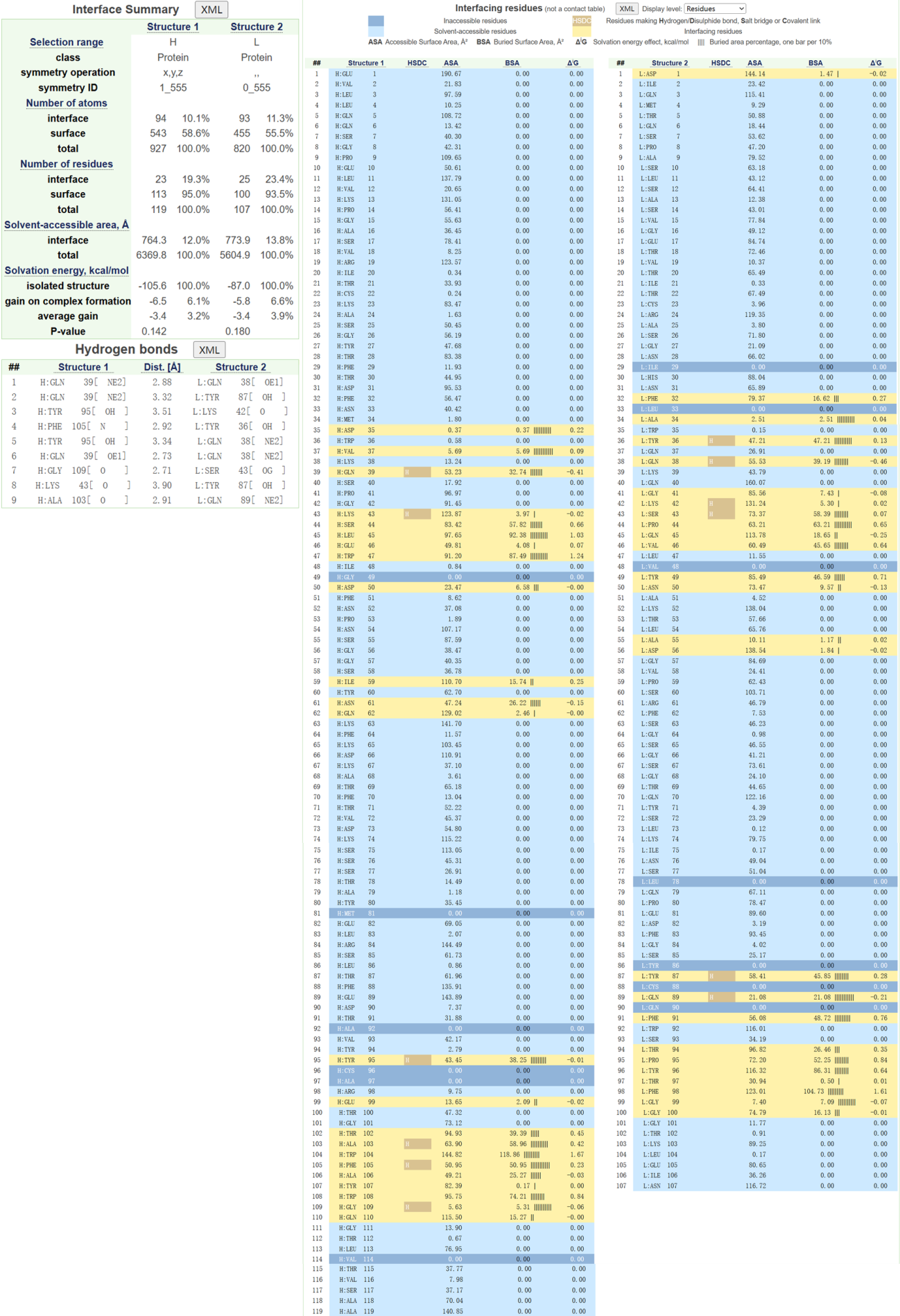
**

**Figure S30.** Molecular docking parameters of AdipoR2 (6KS1) + PPARG (6L8B)

(B) is continuation of (A). ZDOCK is used for docking and PDBePISA is applied to analyze docking results. Available (November 2024): https://zdock.wenglab.org/; https://www.ebi.ac.uk/msd-srv/prot_int/

**
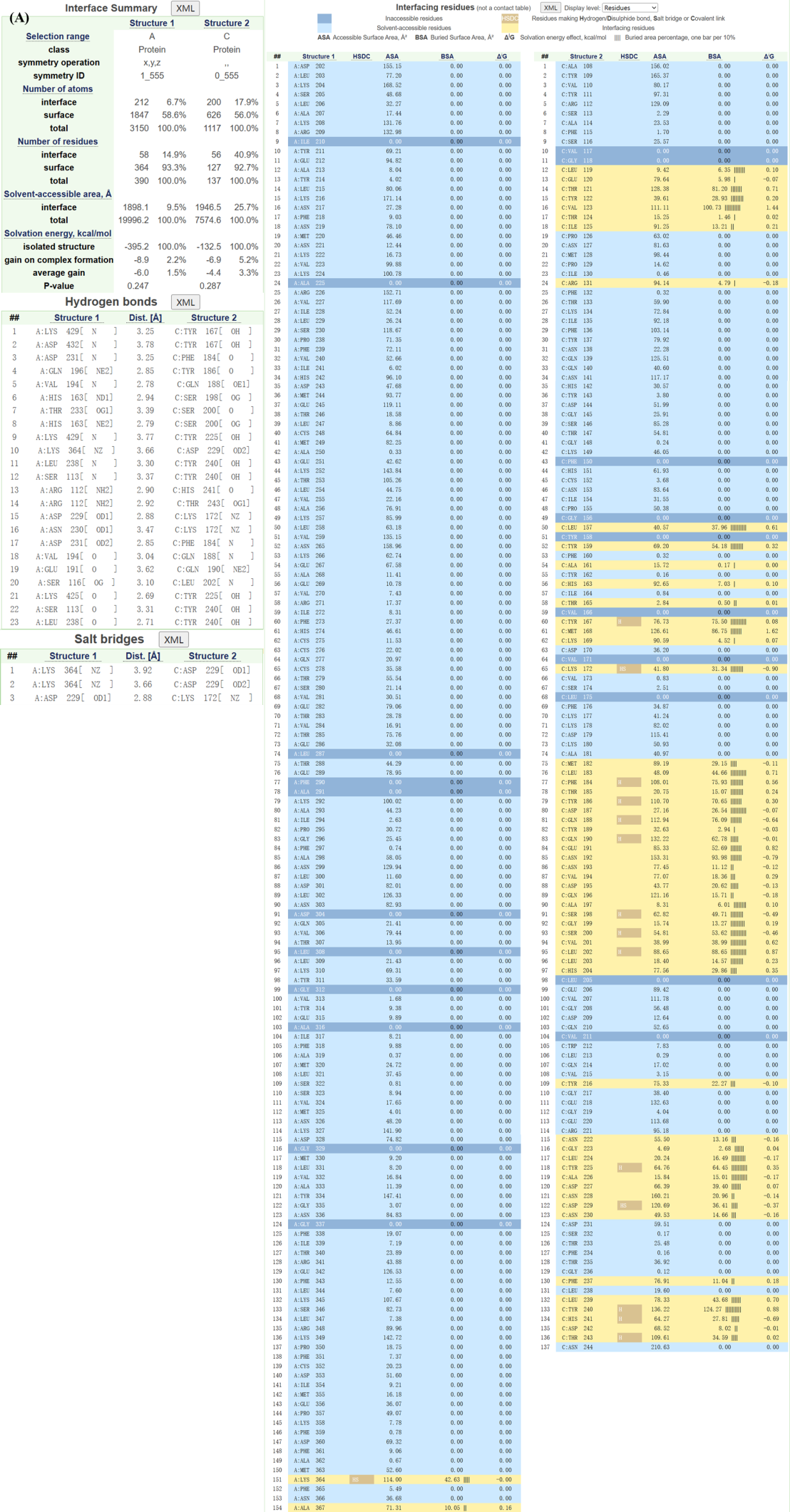
**

**
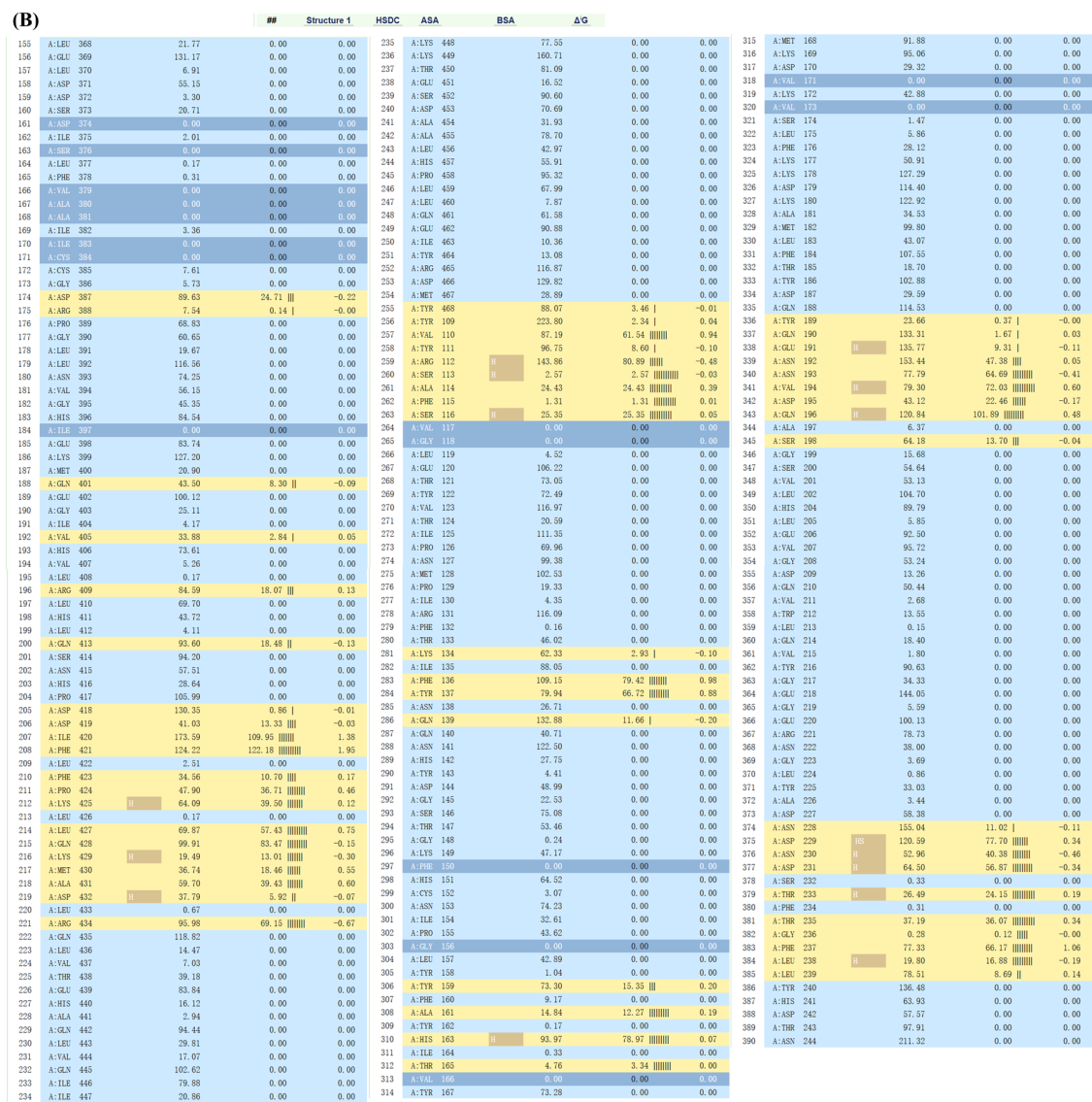
**

**Figure S31.** Molecular docking parameters of APN (6U66) + PPARA/AL26-29 (5HYK)

(B) is continuation of (A). ZDOCK is used for docking and PDBePISA is applied to analyze docking results. Available (November 2024): https://zdock.wenglab.org/; https://www.ebi.ac.uk/msd-srv/prot_int/

**
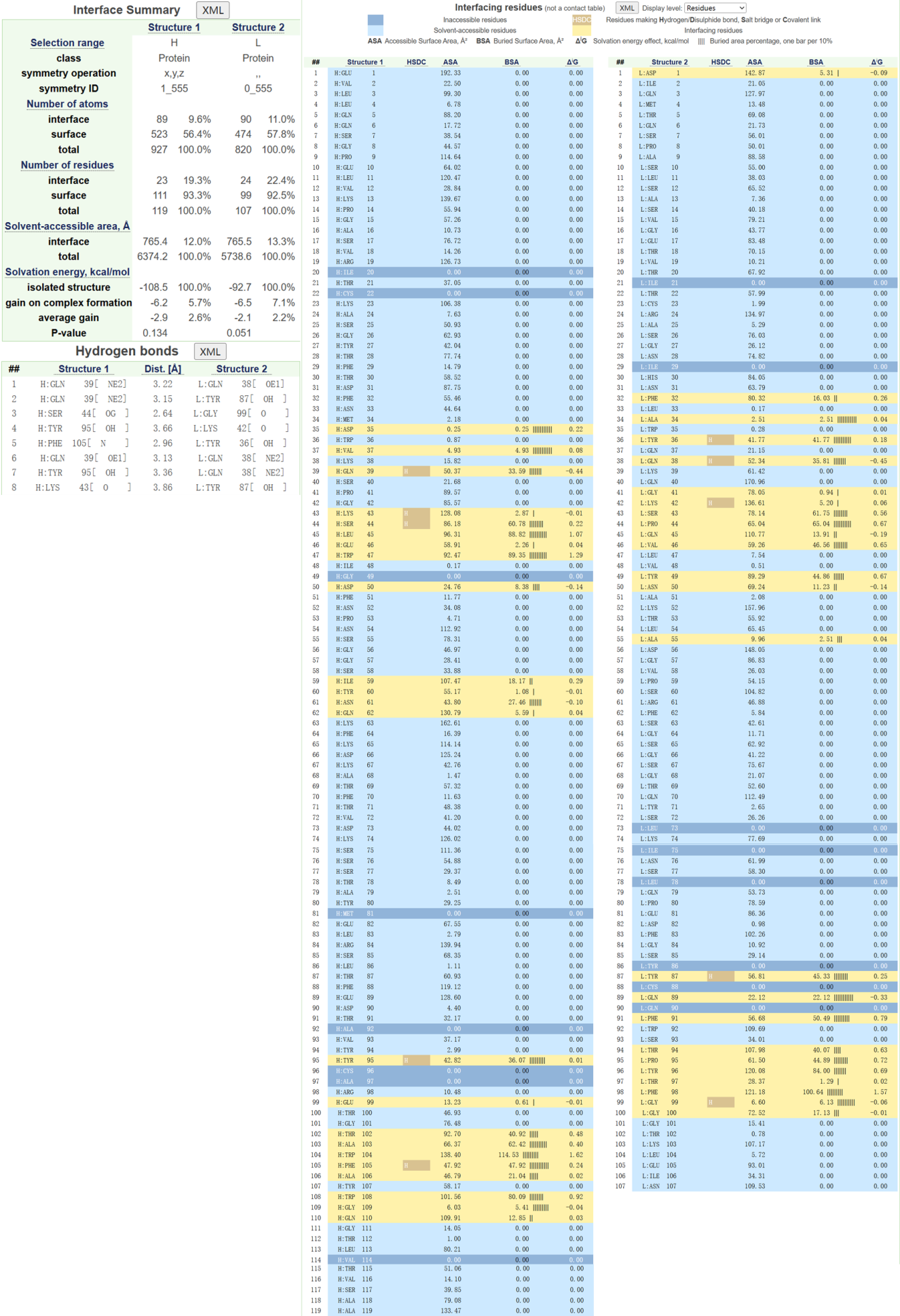
**

**Figure S32.** Molecular docking parameters of AdipoR1 (5LXG) + PPARA/AL26-29 (5HYK)

(B) is continuation of (A). ZDOCK is used for docking and PDBePISA is applied to analyze docking results. Available (November 2024): https://zdock.wenglab.org/; https://www.ebi.ac.uk/msd-srv/prot_int/

**
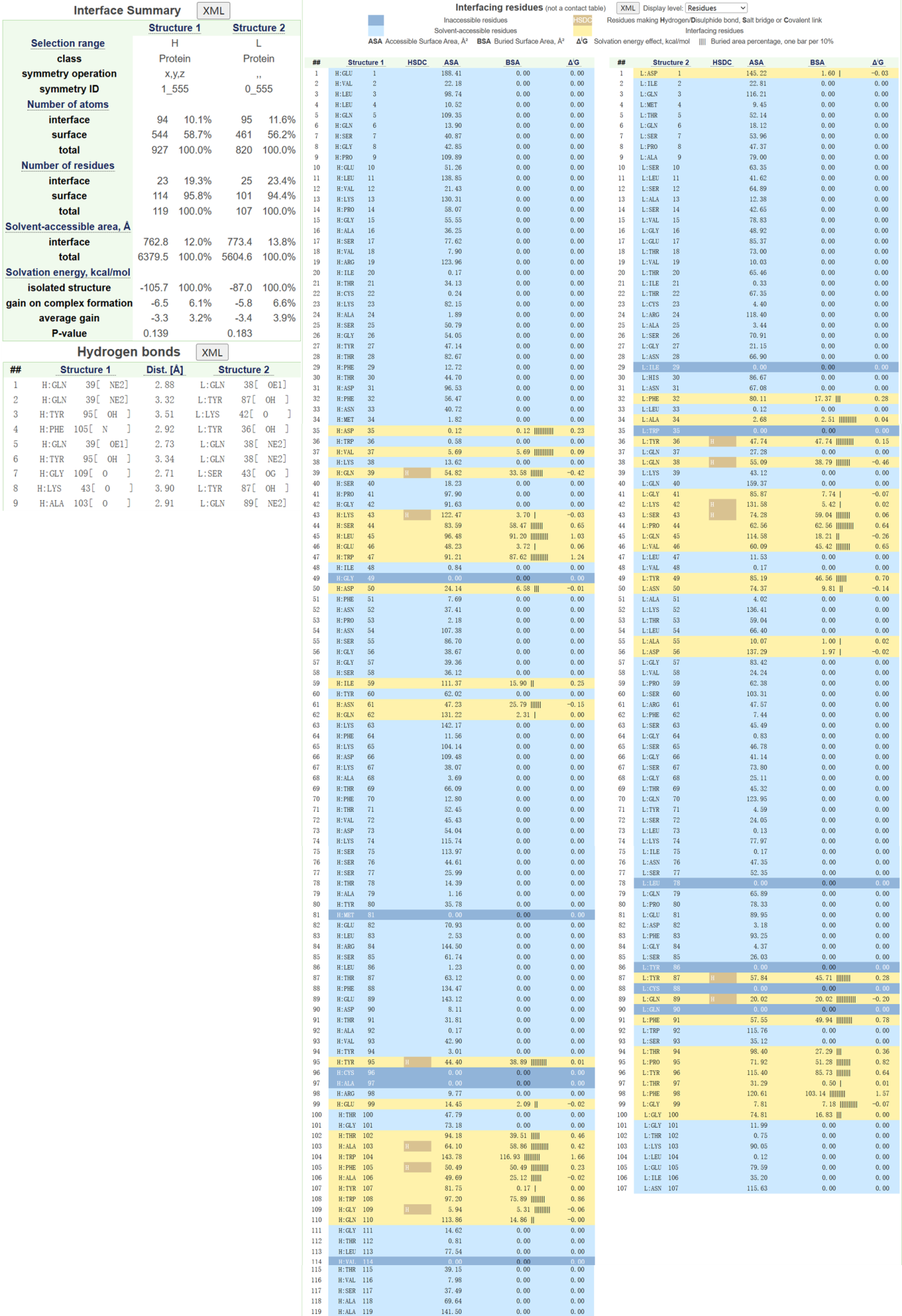
**

**Figure S33.** Molecular docking parameters of AdipoR2 (6KS1) + PPARA/AL26-29 (5HYK)

(B) is continuation of (A). ZDOCK is used for docking and PDBePISA is applied to analyze docking results. Available (November 2024): https://zdock.wenglab.org/; <https://www.ebi.ac.uk/msd-srv/prot_int/>


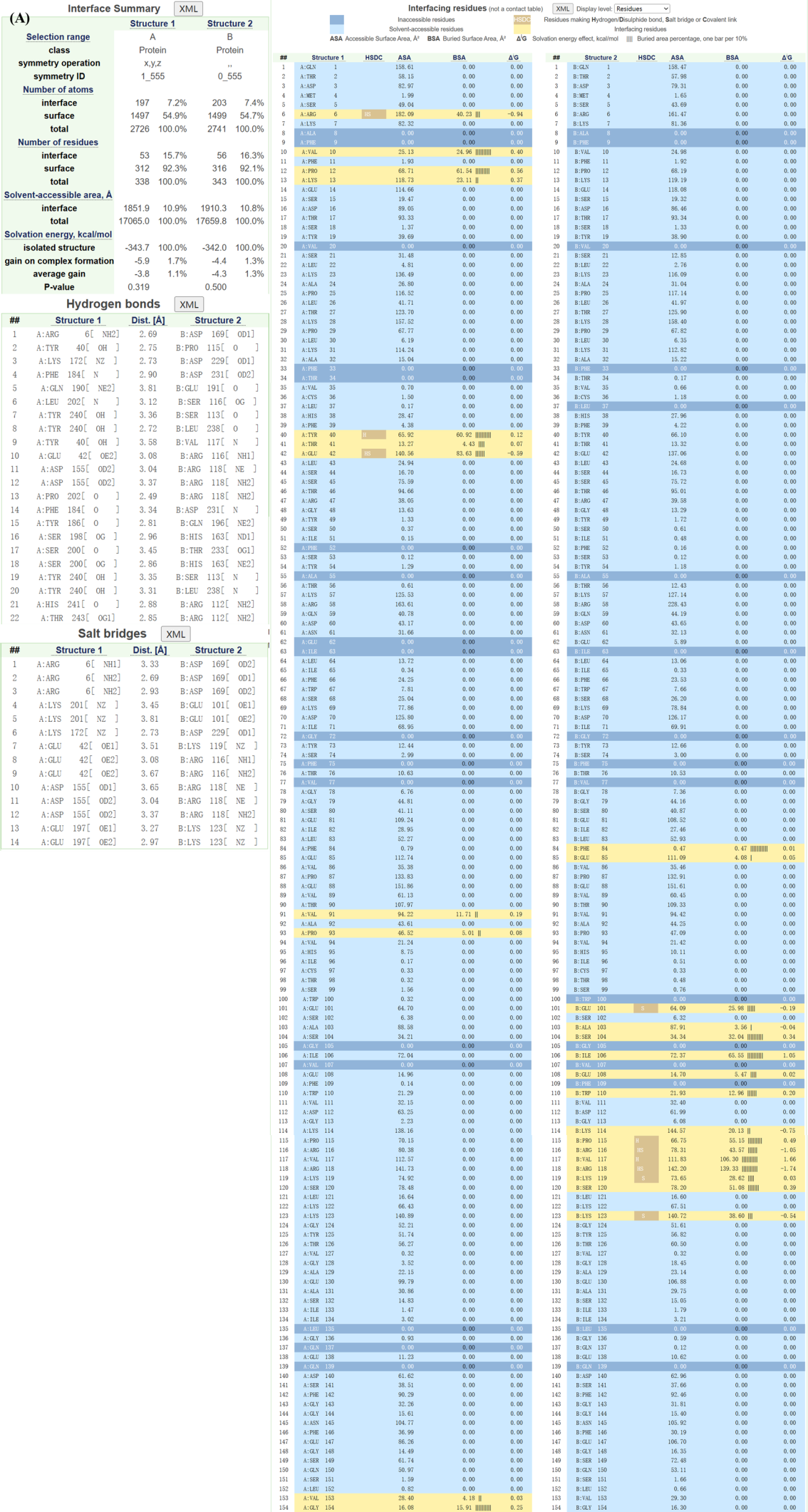


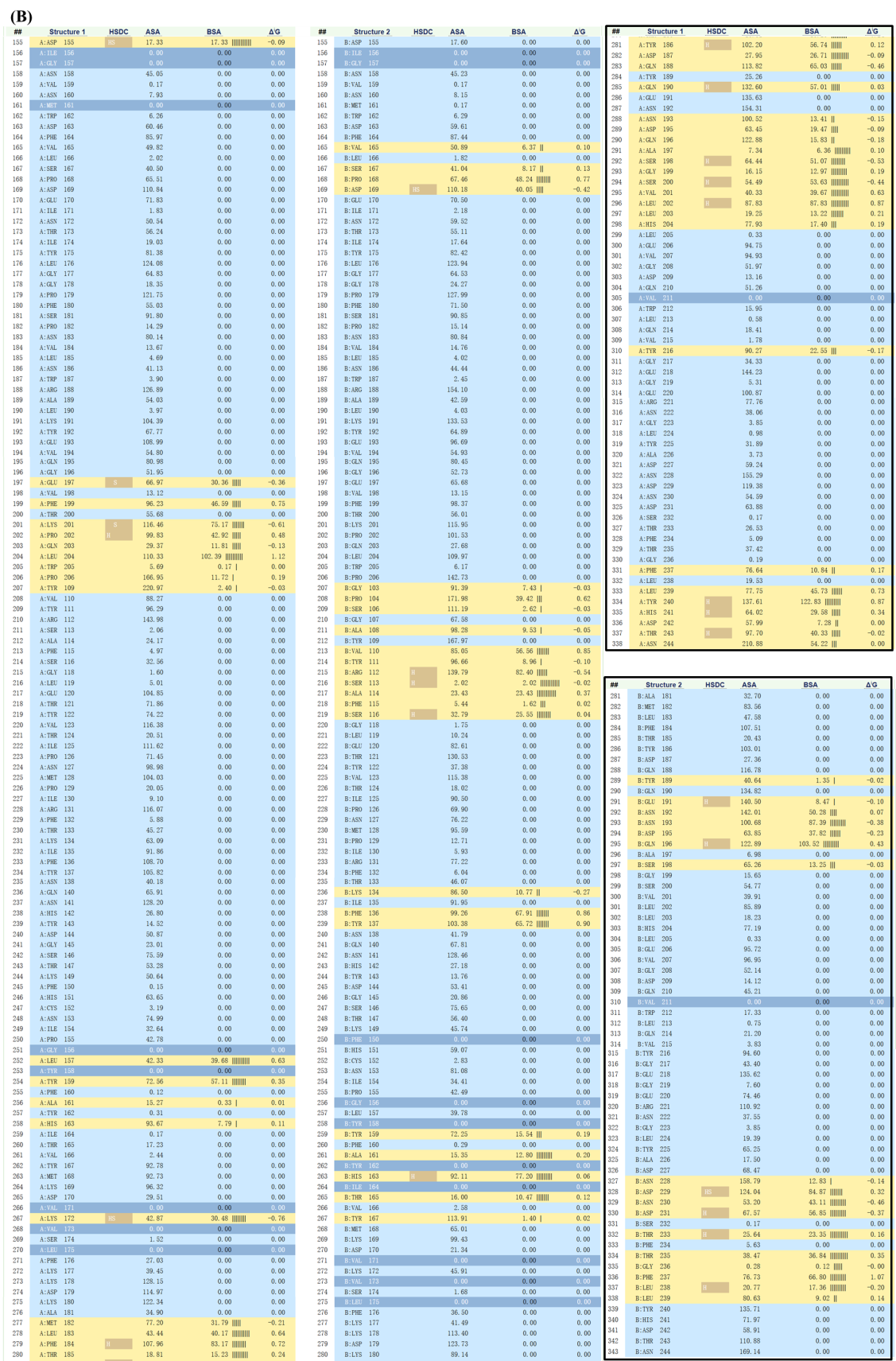


**Figure S34.** Molecular docking parameters of APN (6U66) + CRP (1GNH)

(B) is continuation of (A). ZDOCK is used for docking and PDBePISA is applied to analyze docking results. Available (November 2024): https://zdock.wenglab.org/; https://www.ebi.ac.uk/msd-srv/prot_int/

**
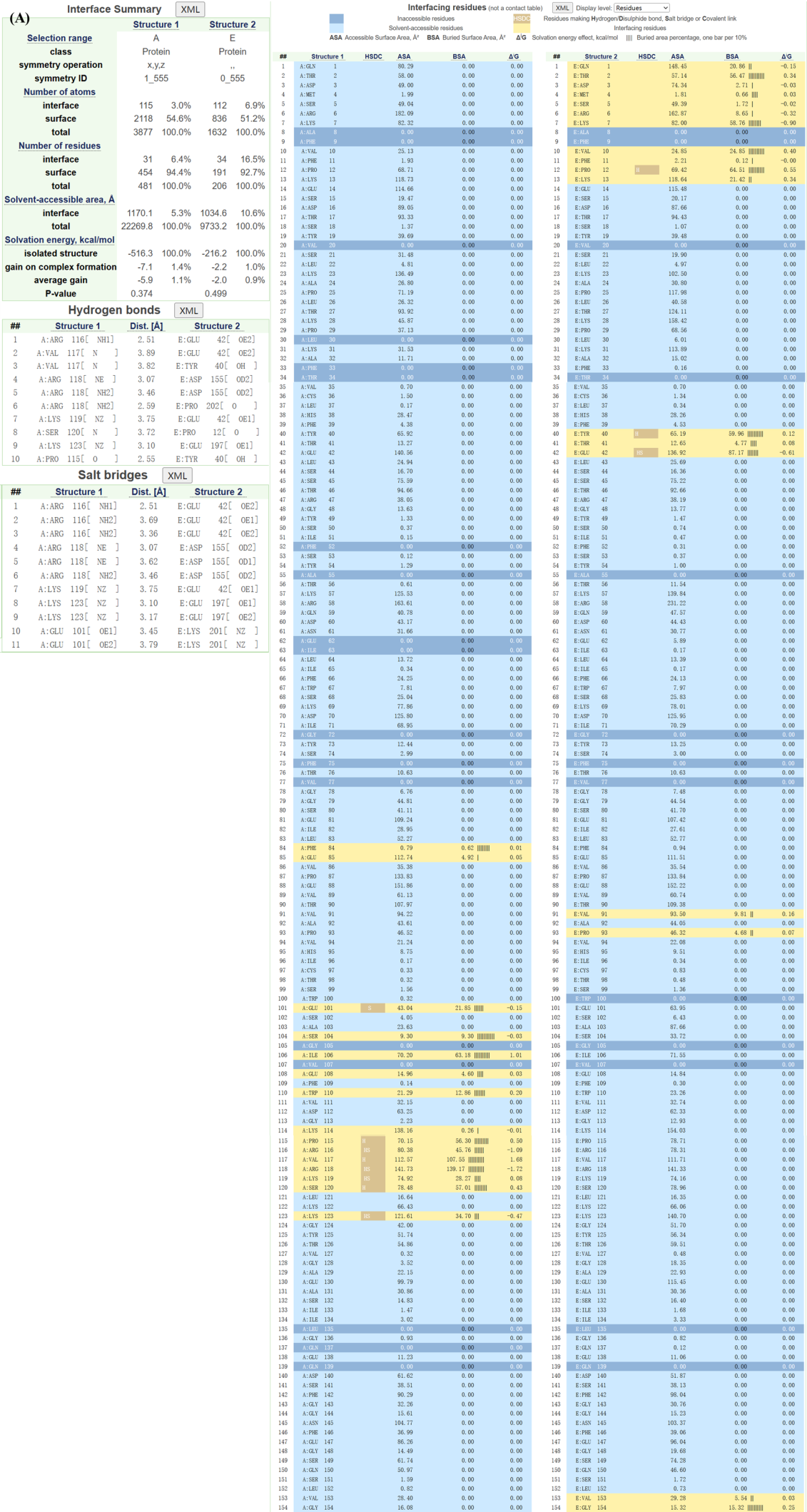
**

**
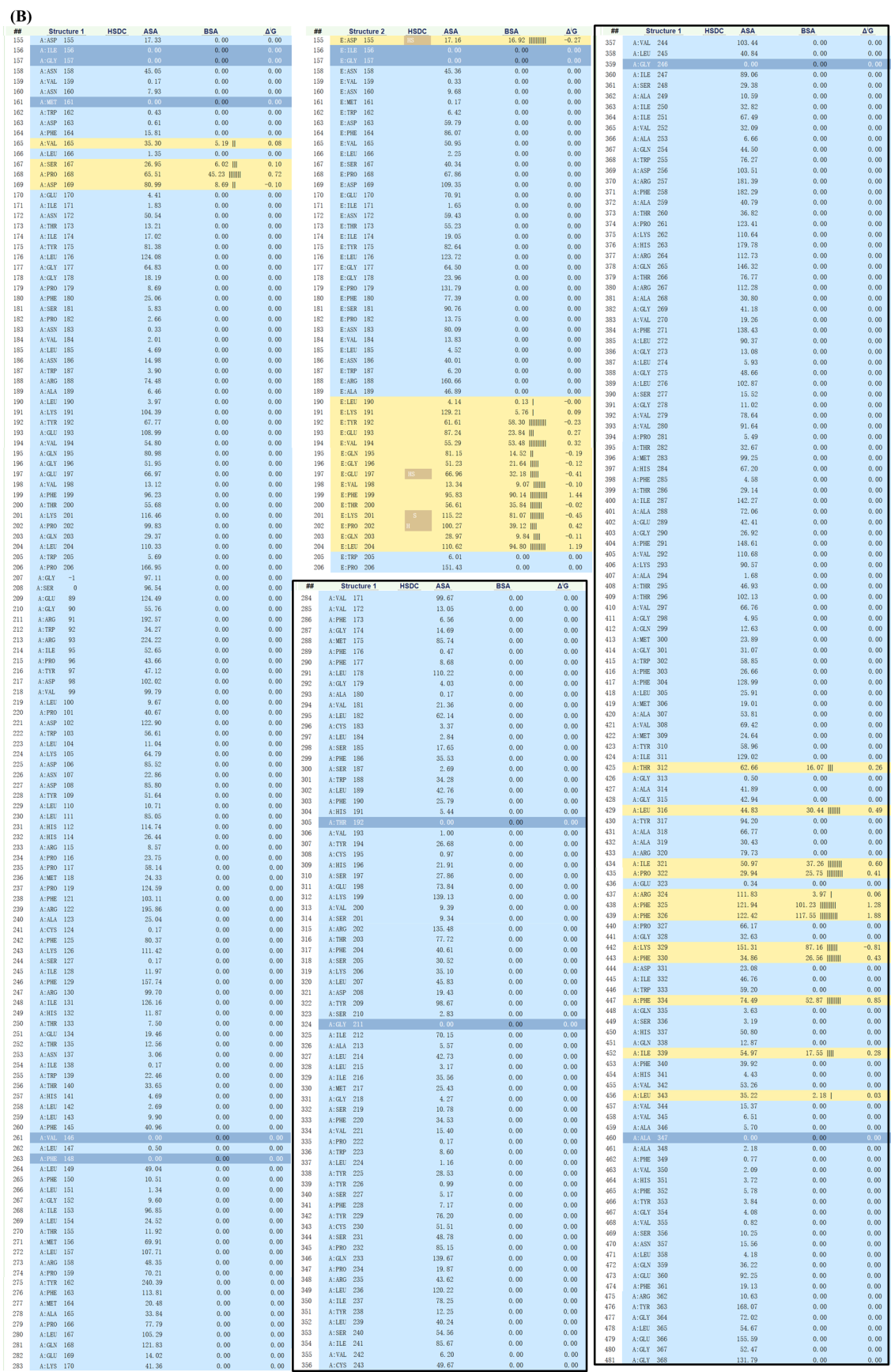
**

**Figure S35.** Molecular docking parameters of AdipoR1 (5LXG) + CRP (1GNH))

(B) is continuation of (A). ZDOCK is used for docking and PDBePISA is applied to analyze docking results. Available (November 2024): https://zdock.wenglab.org/; https://www.ebi.ac.uk/msd-srv/prot_int/

**
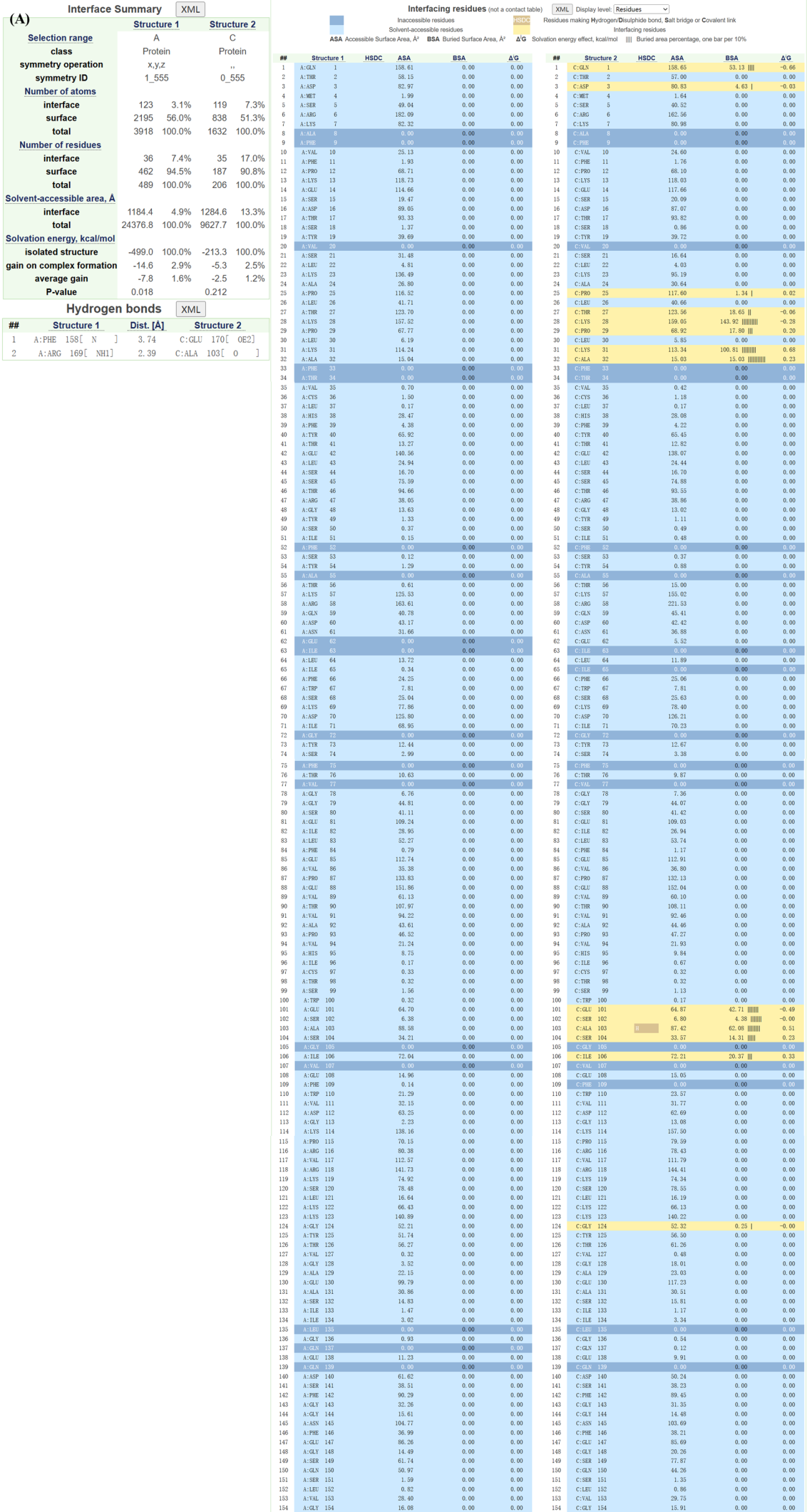
**

**
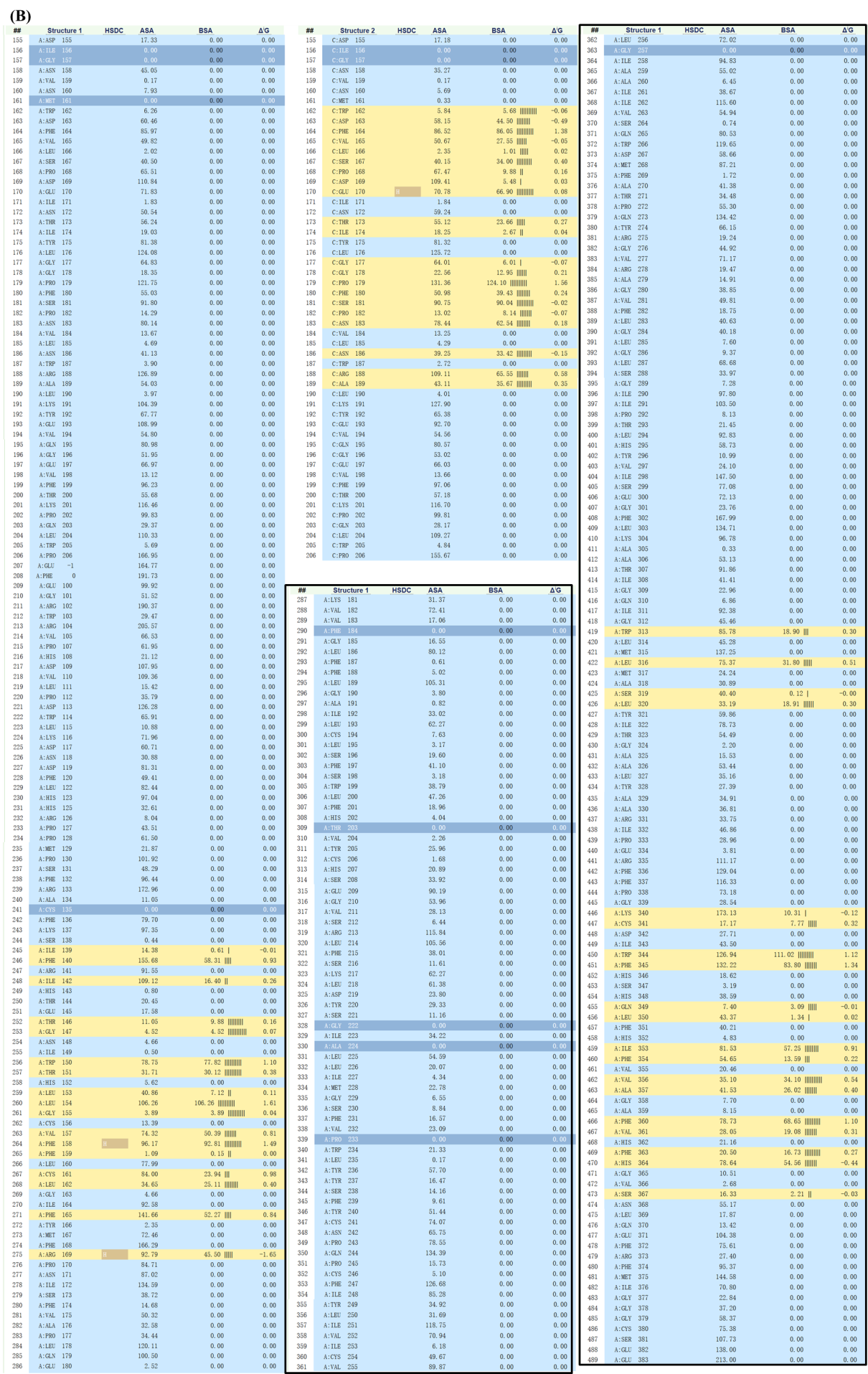
**

**Figure S36.** Molecular docking parameters of AdipoR2 (6KS1) + CRP (1GNH)

(B) is continuation of (A). ZDOCK is used for docking and PDBePISA is applied to analyze docking results. Available (November 2024): https://zdock.wenglab.org/; https://www.ebi.ac.uk/msd-srv/prot_int/


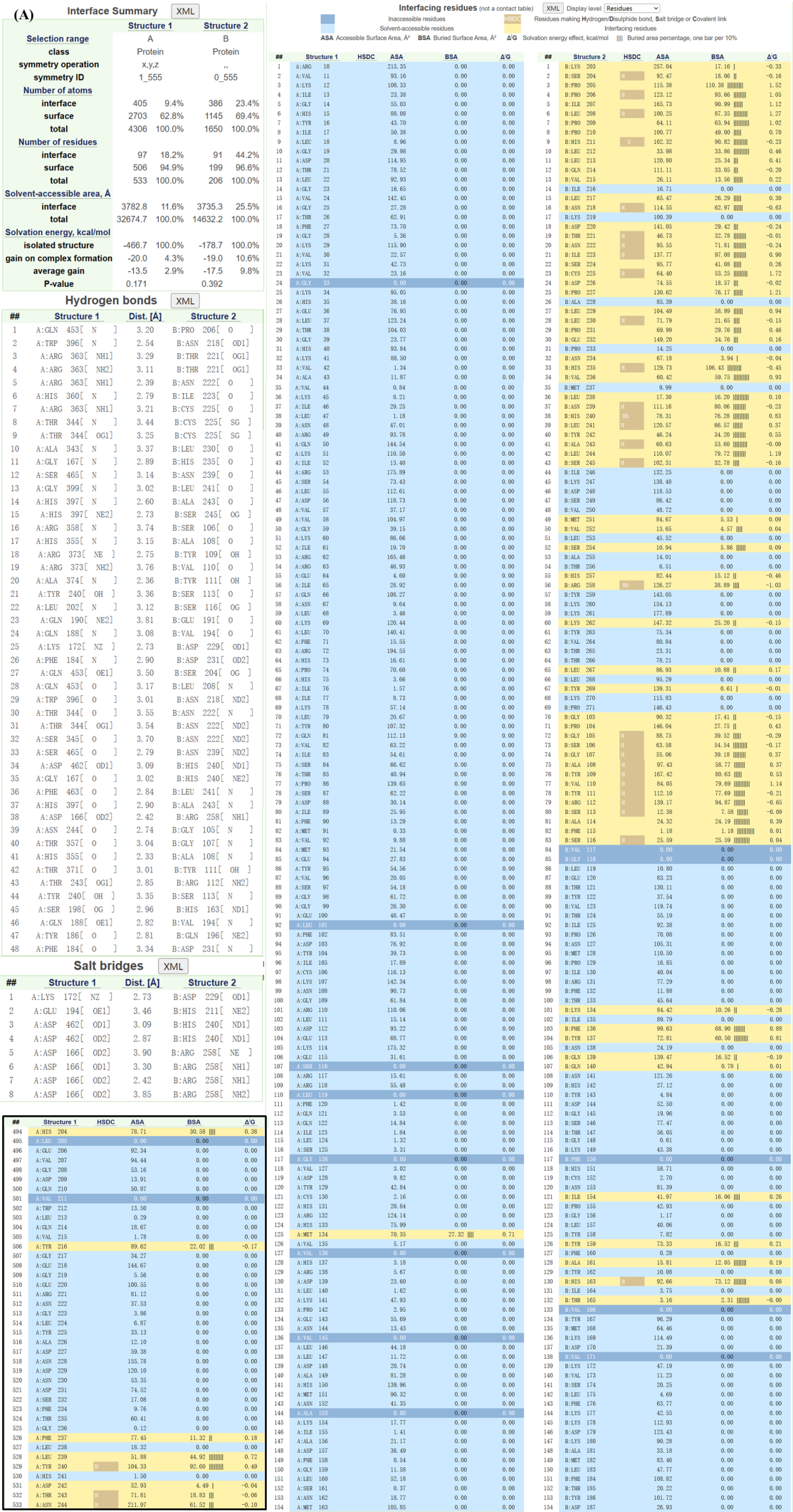


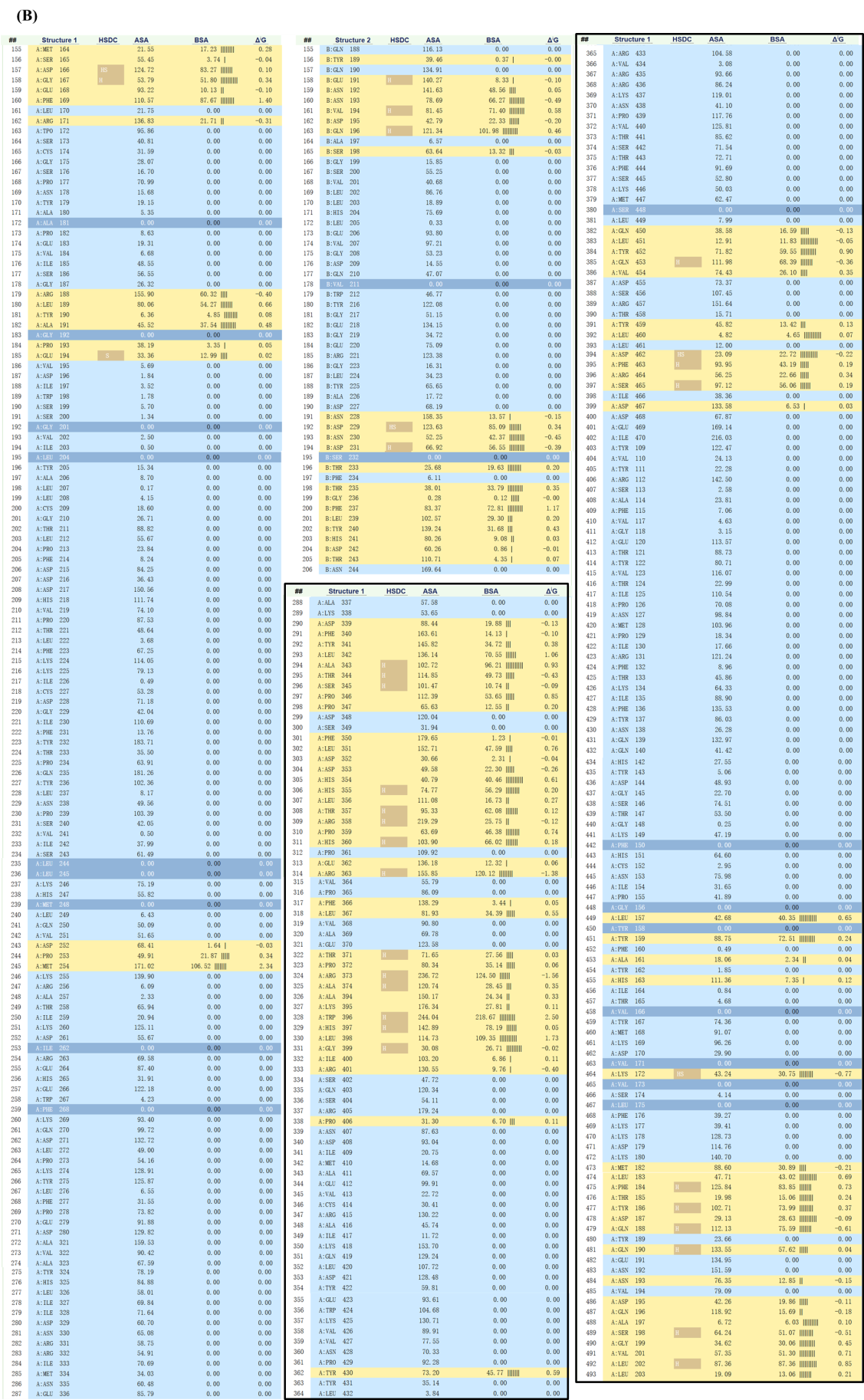


**Figure S37.** Molecular docking parameters of APN (6U66) + AMPK (4CFH)

(B) is continuation of (A). ZDOCK is used for docking and PDBePISA is applied to analyze docking results. Available (November 2024): https://zdock.wenglab.org/; https://www.ebi.ac.uk/msd-srv/prot_int/

**
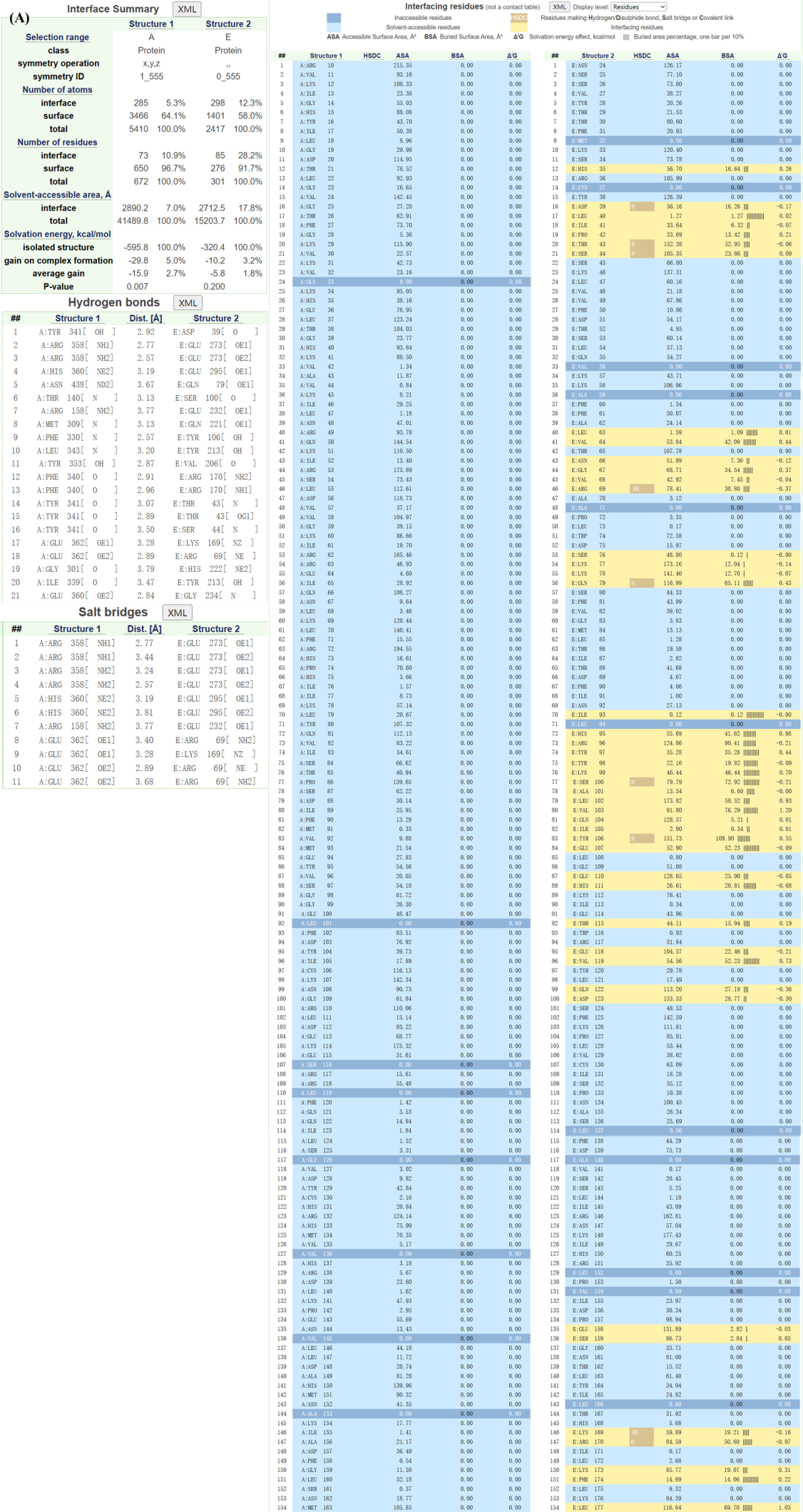
**

**
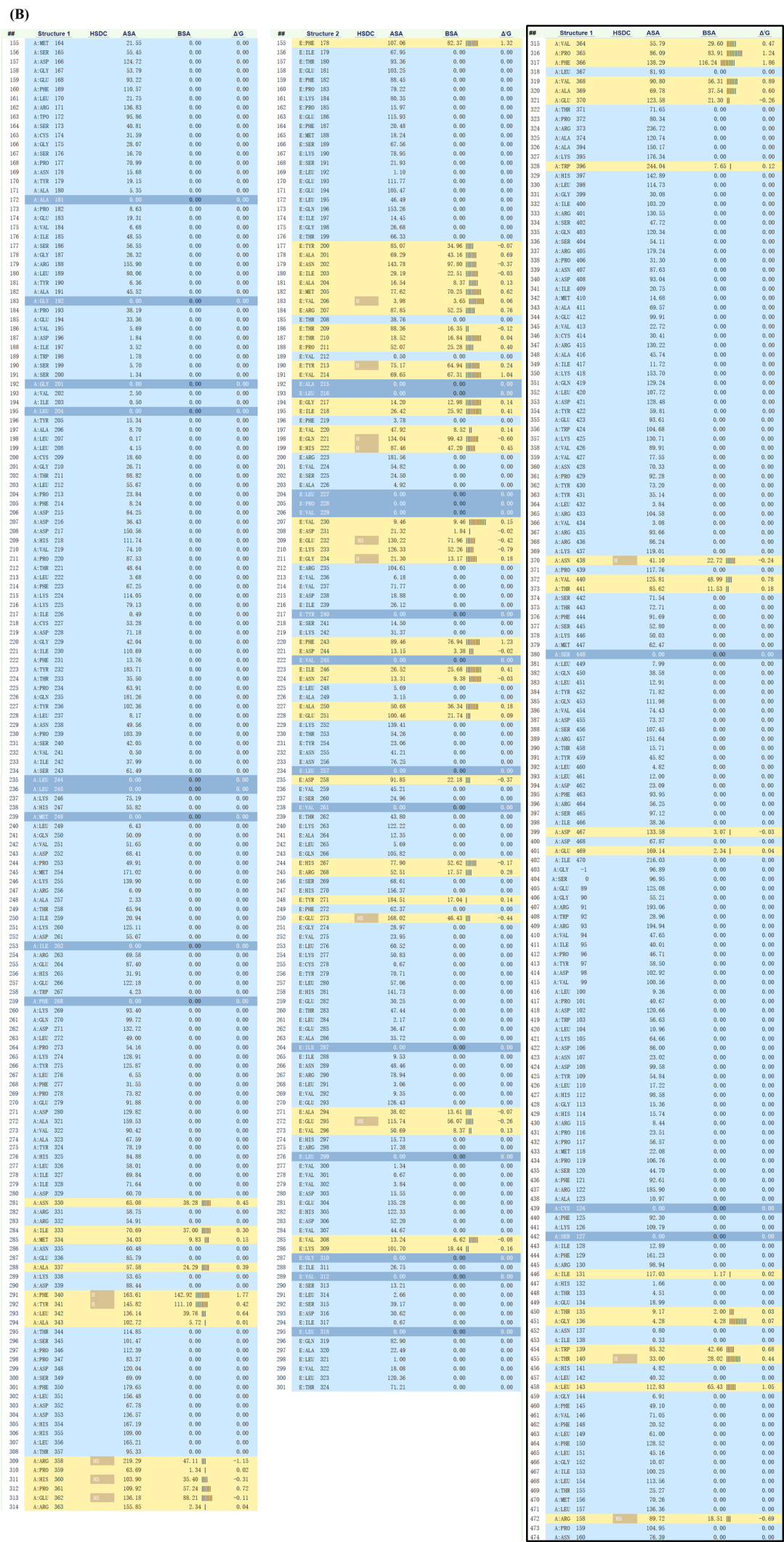
**

**
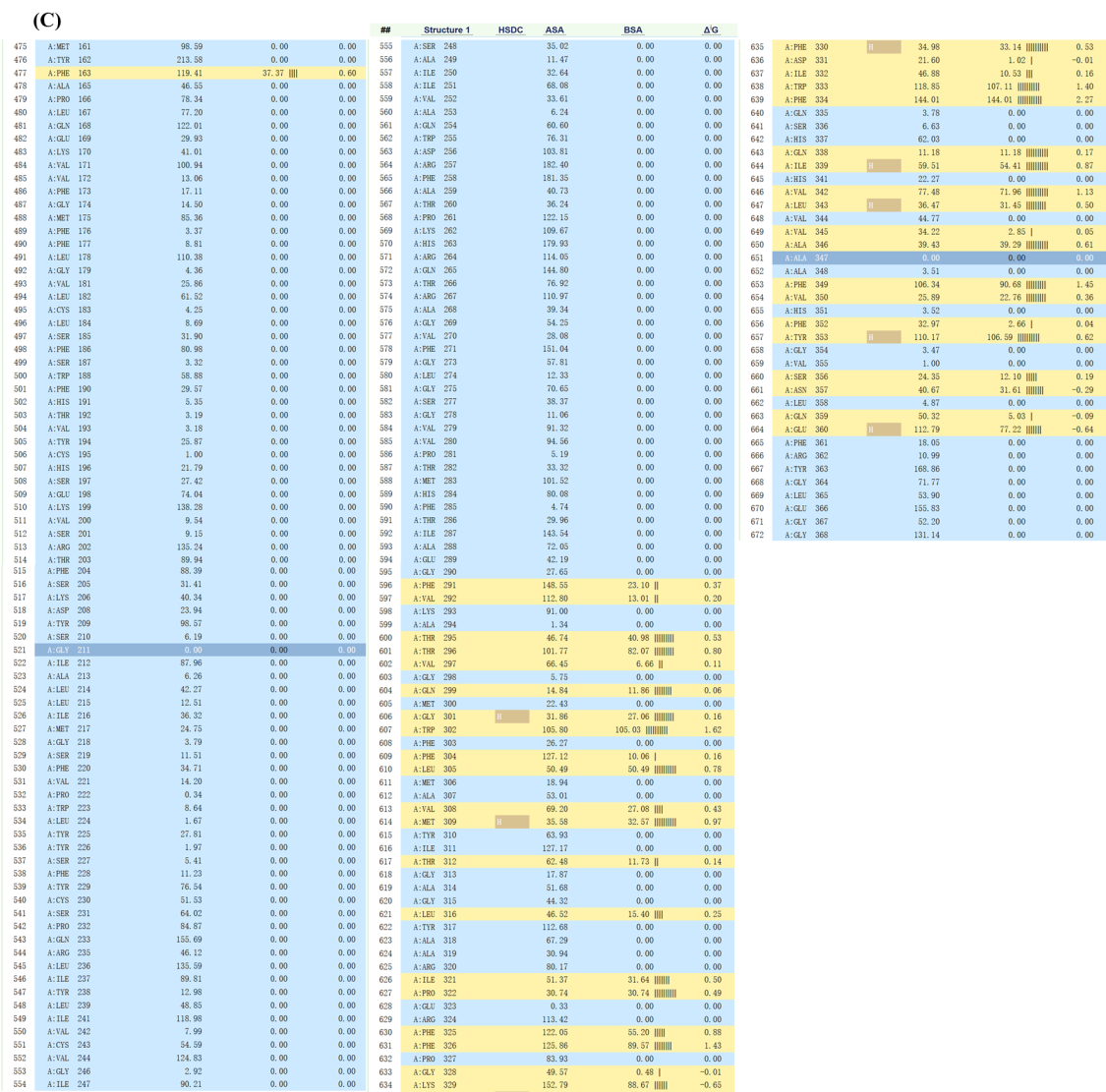
**

**Figure S38.** Molecular docking parameters of AdipoR1 (5LXG) + AMPK (4CFH)

(B-C) is continuation of (A). ZDOCK is used for docking and PDBePISA is applied to analyze docking results. Available (November 2024): https://zdock.wenglab.org/; https://www.ebi.ac.uk/msd-srv/prot_int/

**
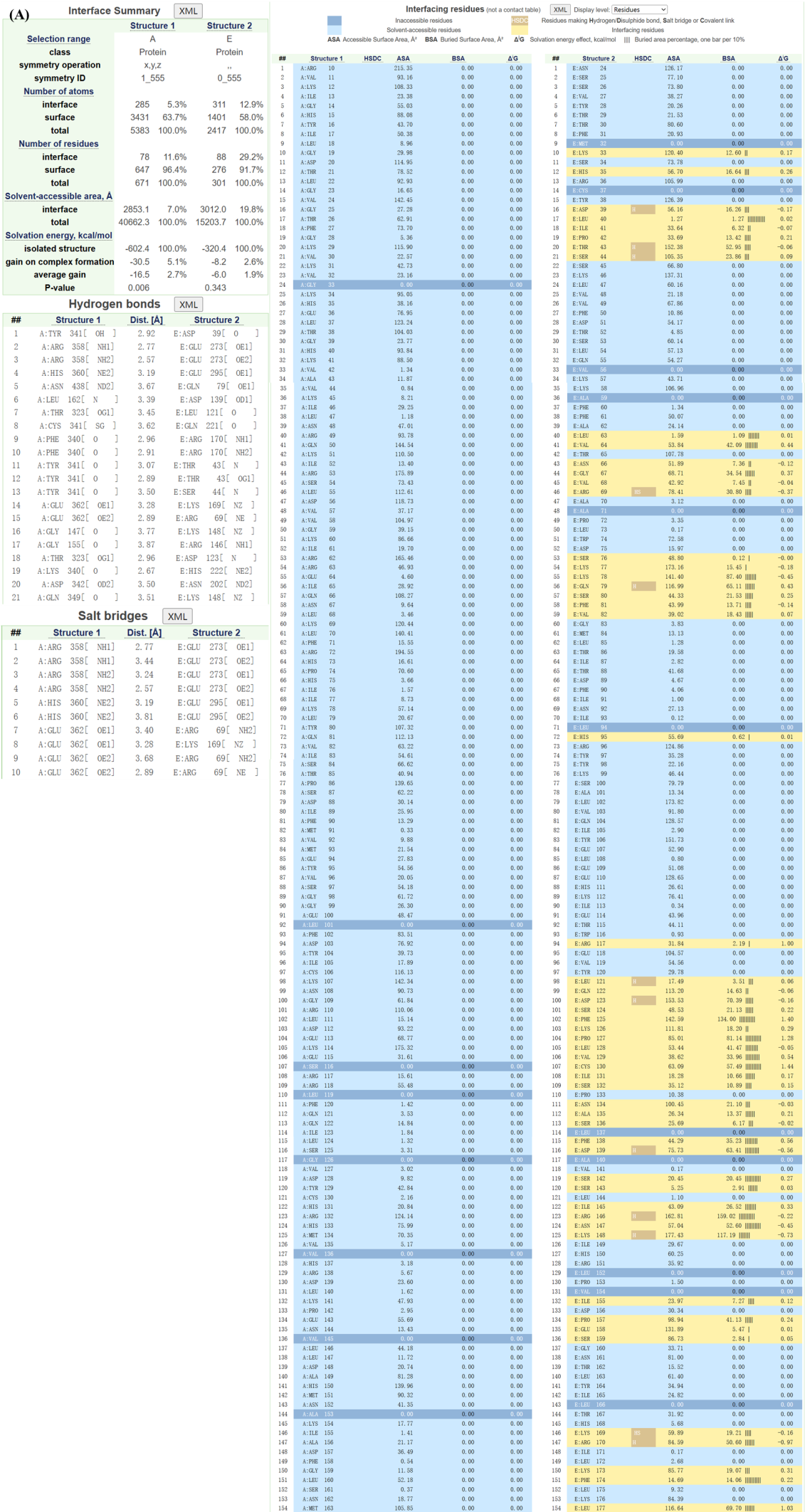
**

**
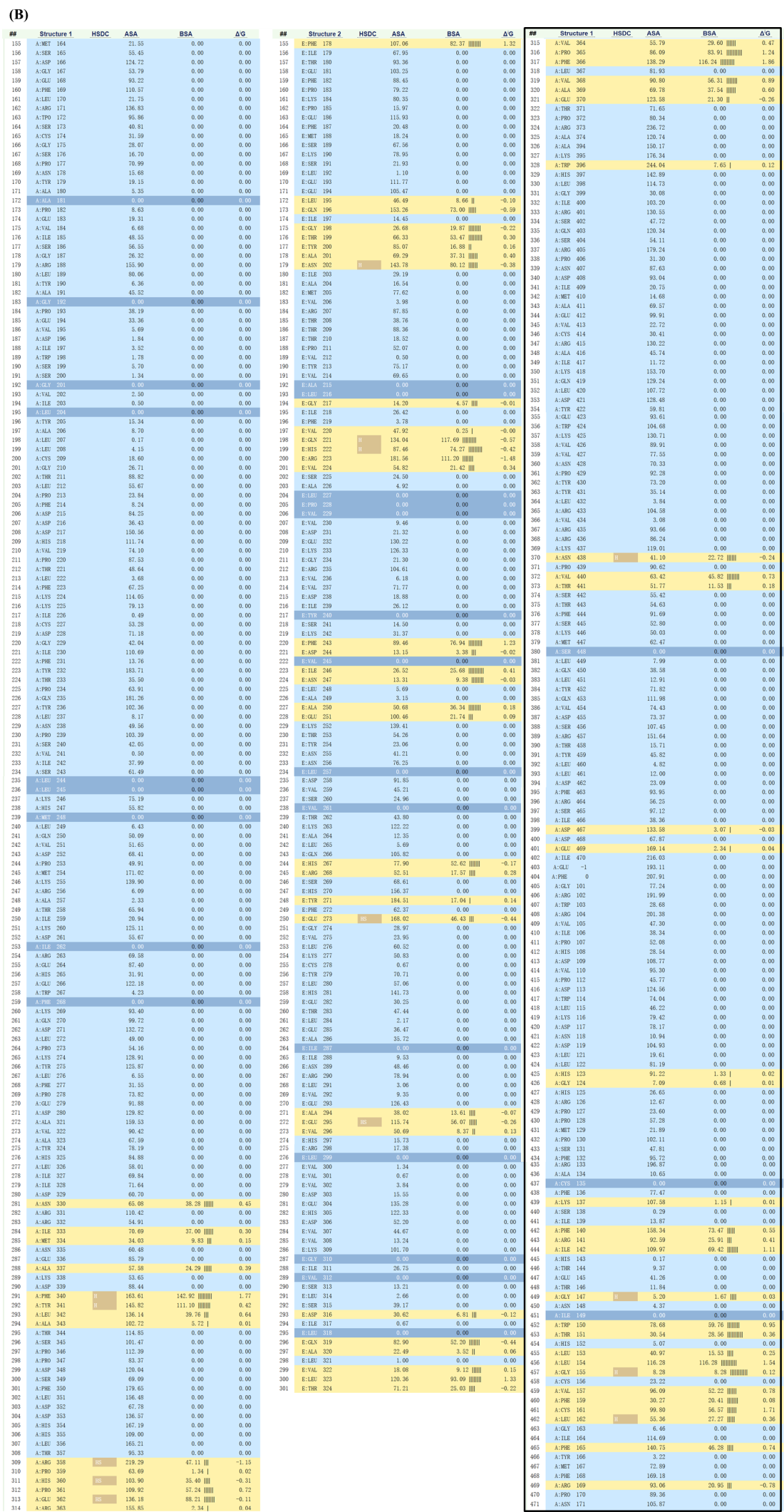
**

**
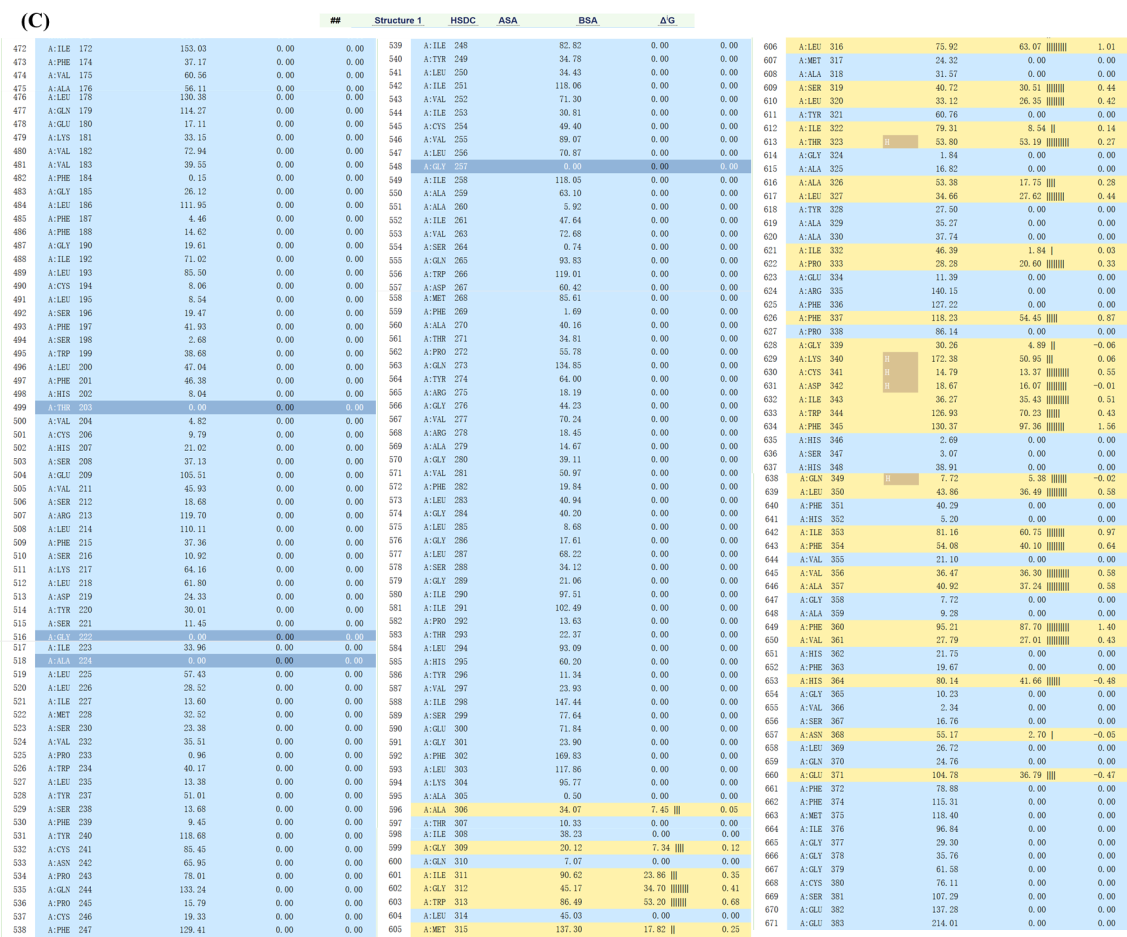
**

**Figure S39.** Molecular docking parameters of AdipoR2 (6KS1) + AMPK (4CFH)

(B-C) is continuation of (A). ZDOCK is used for docking and PDBePISA is applied to analyze docking results. Available (November 2024): https://zdock.wenglab.org/; https://www.ebi.ac.uk/msd-srv/prot_int/


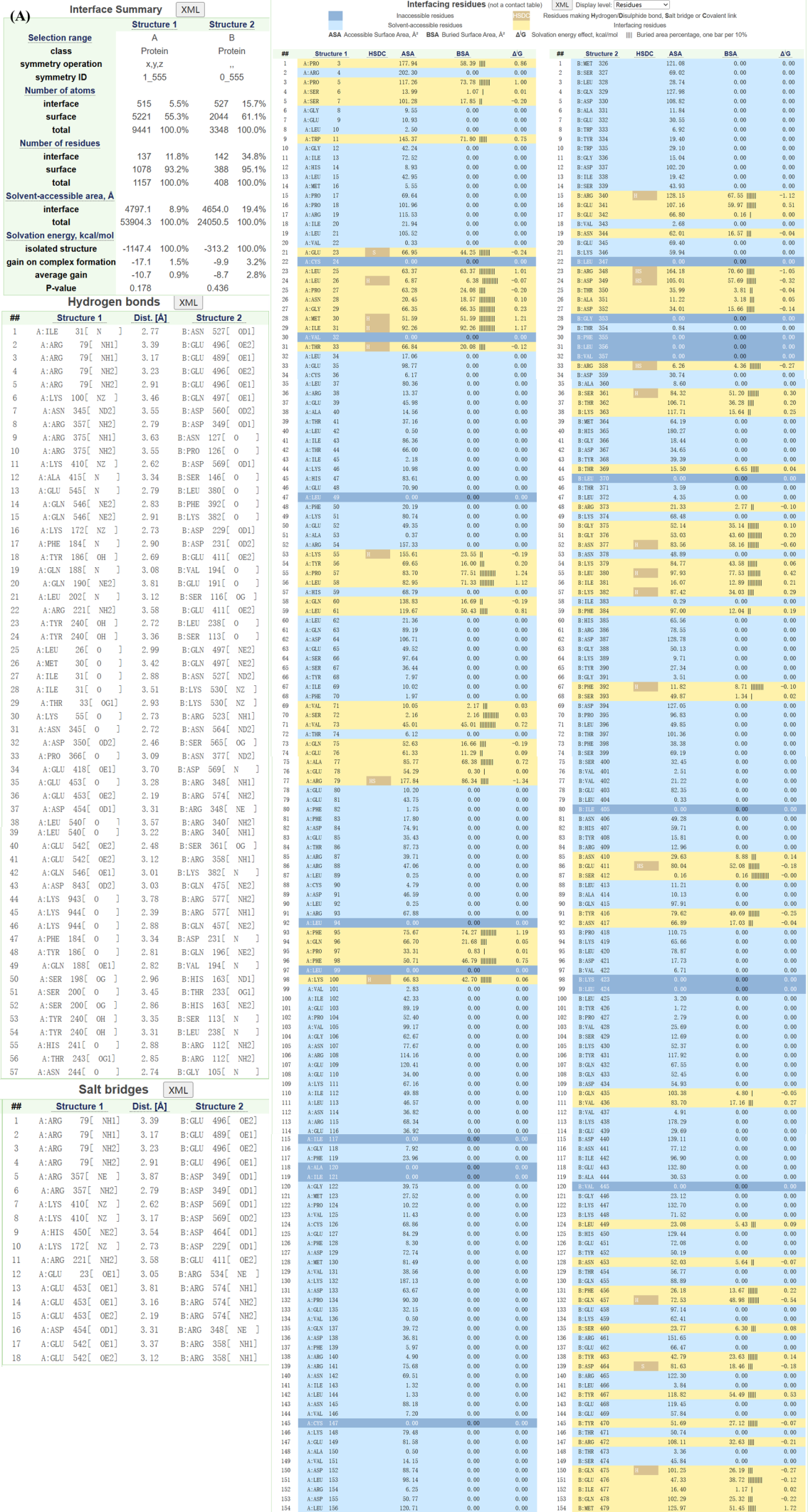


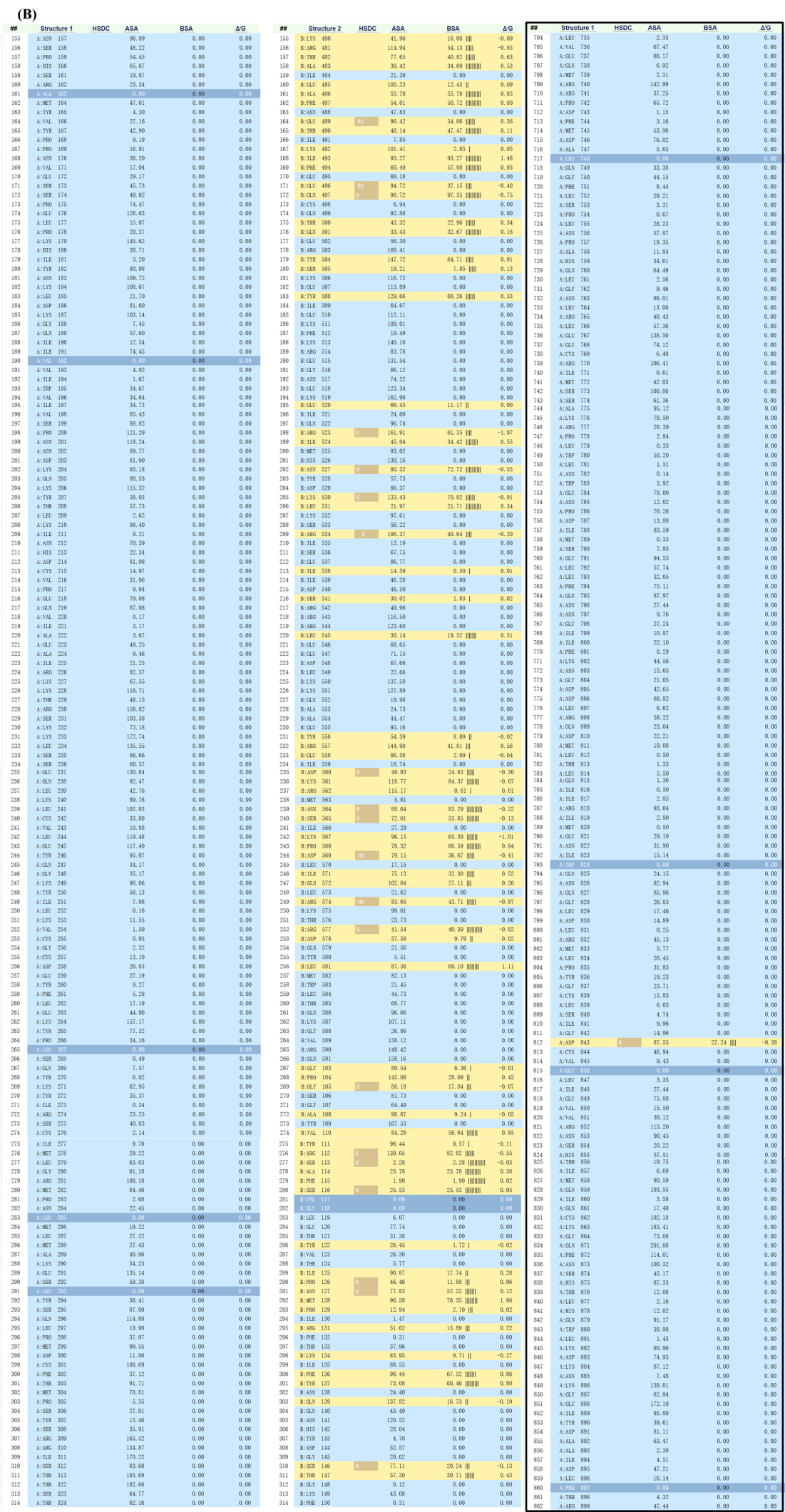


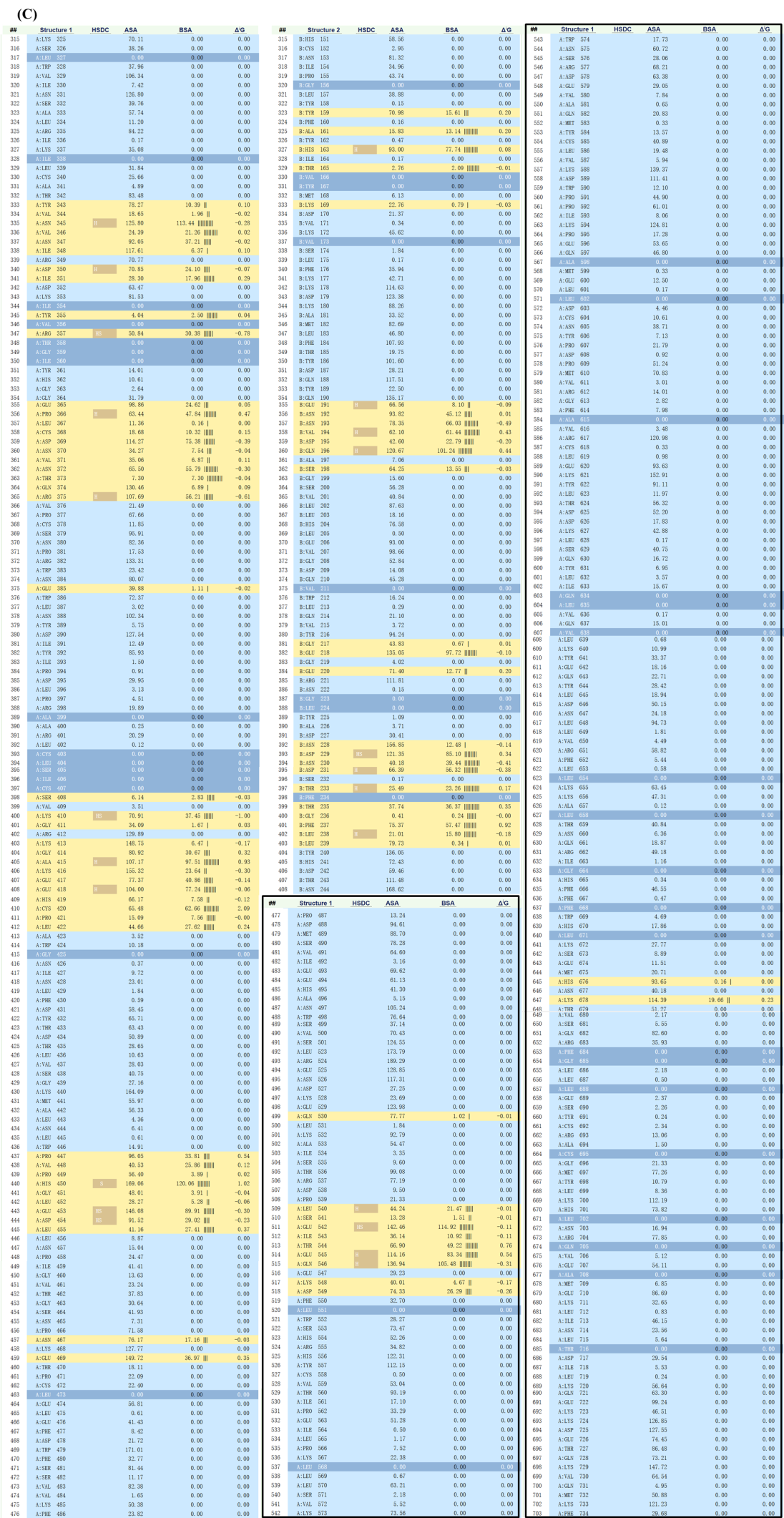


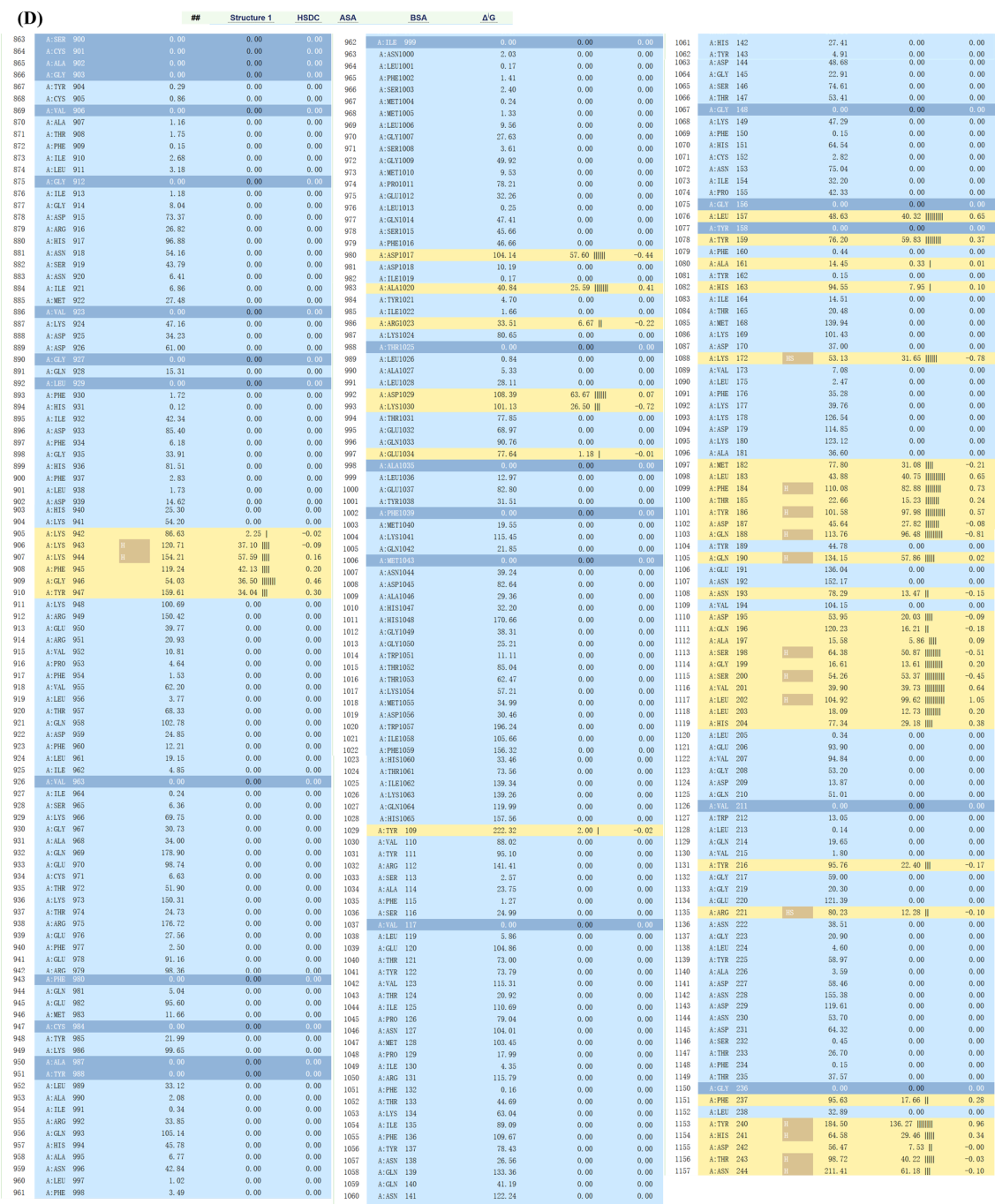


**Figure S40.** Molecular docking parameters of APN (6U66) + PI3Kα (7PG5)

(B-D) is continuation of (A). ZDOCK is used for docking and PDBePISA is applied to analyze docking results. Available (November 2024): https://zdock.wenglab.org/; https://www.ebi.ac.uk/msd-srv/prot_int/

**
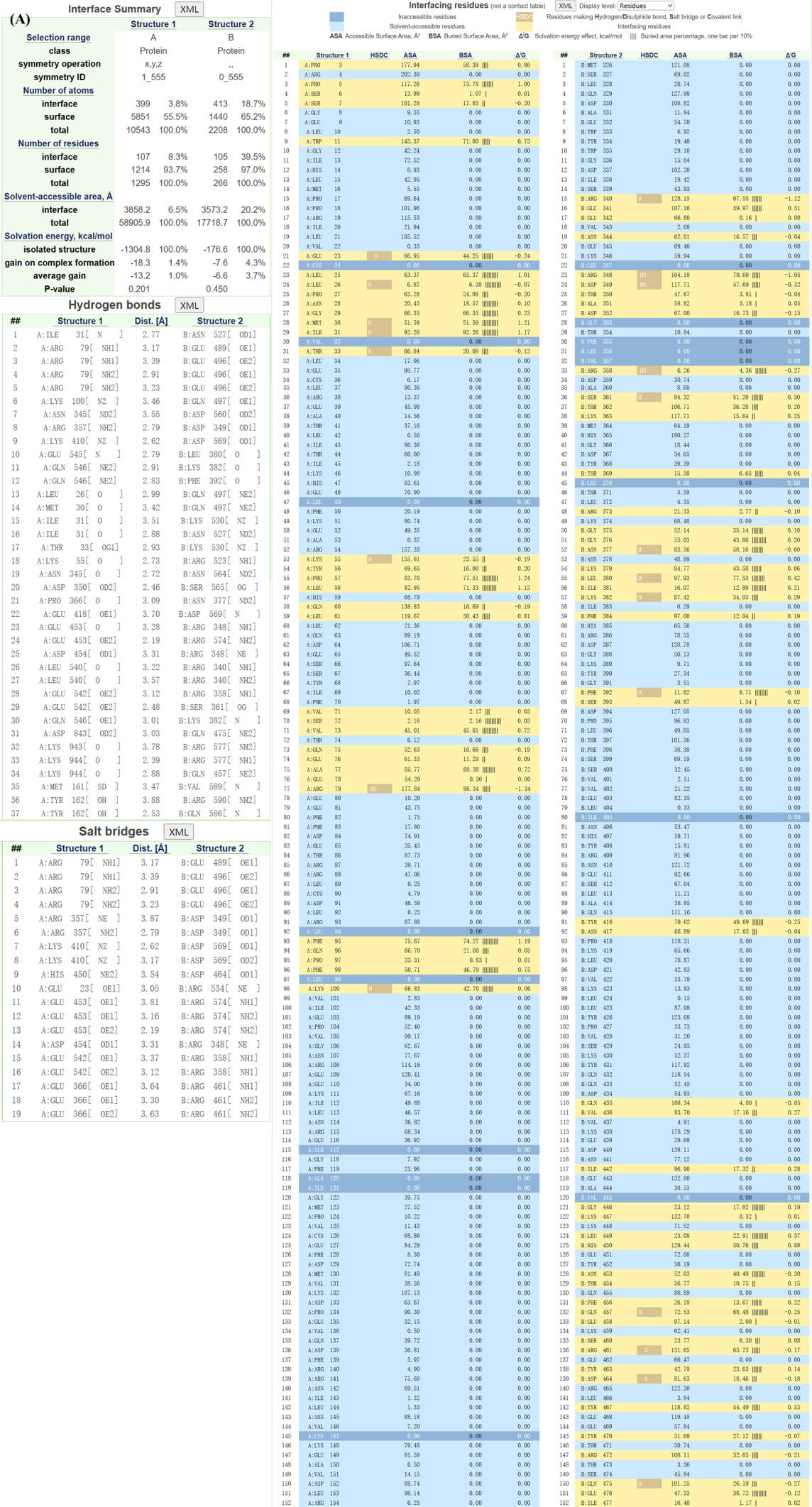
**

**
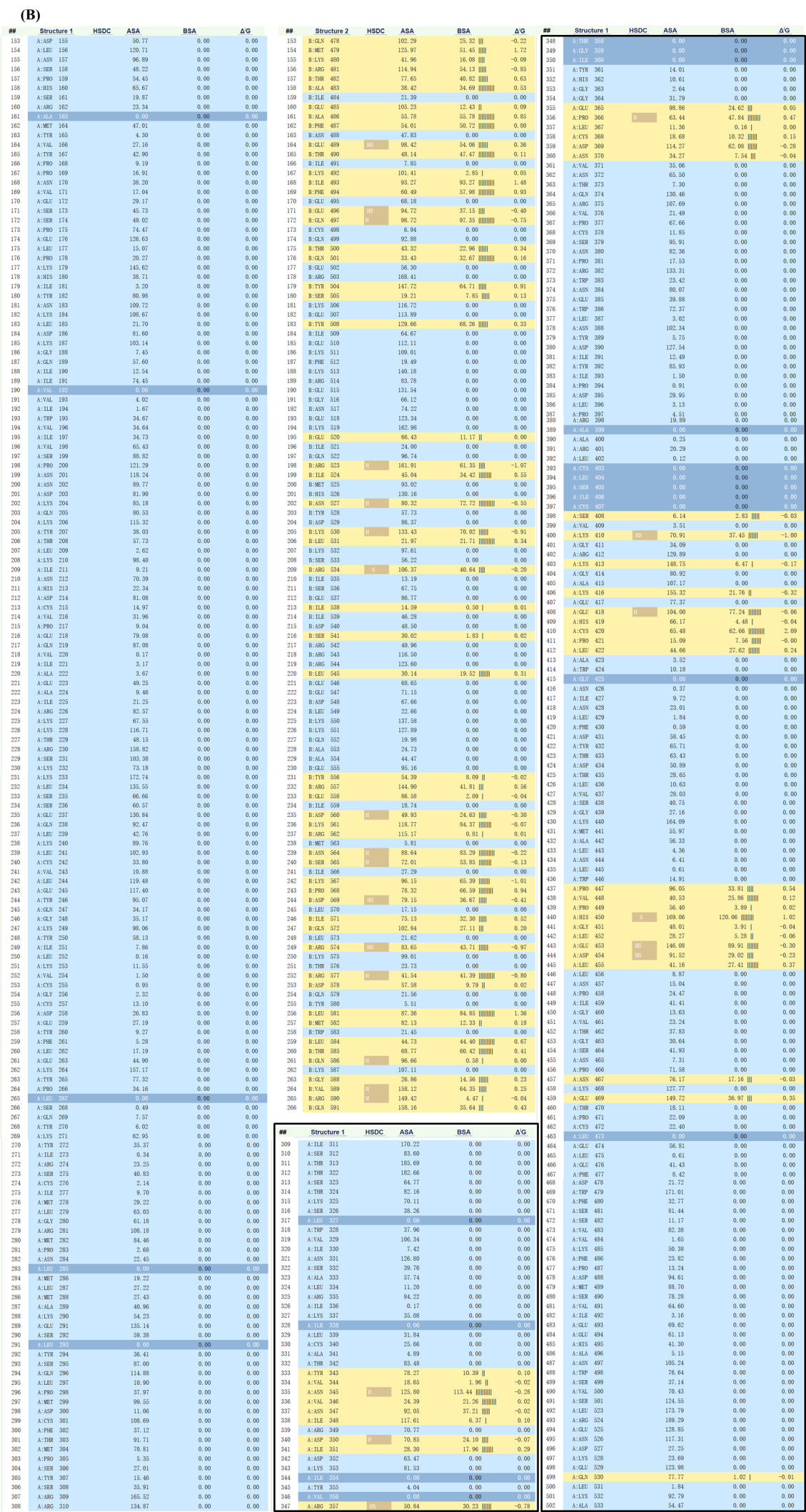
**

**
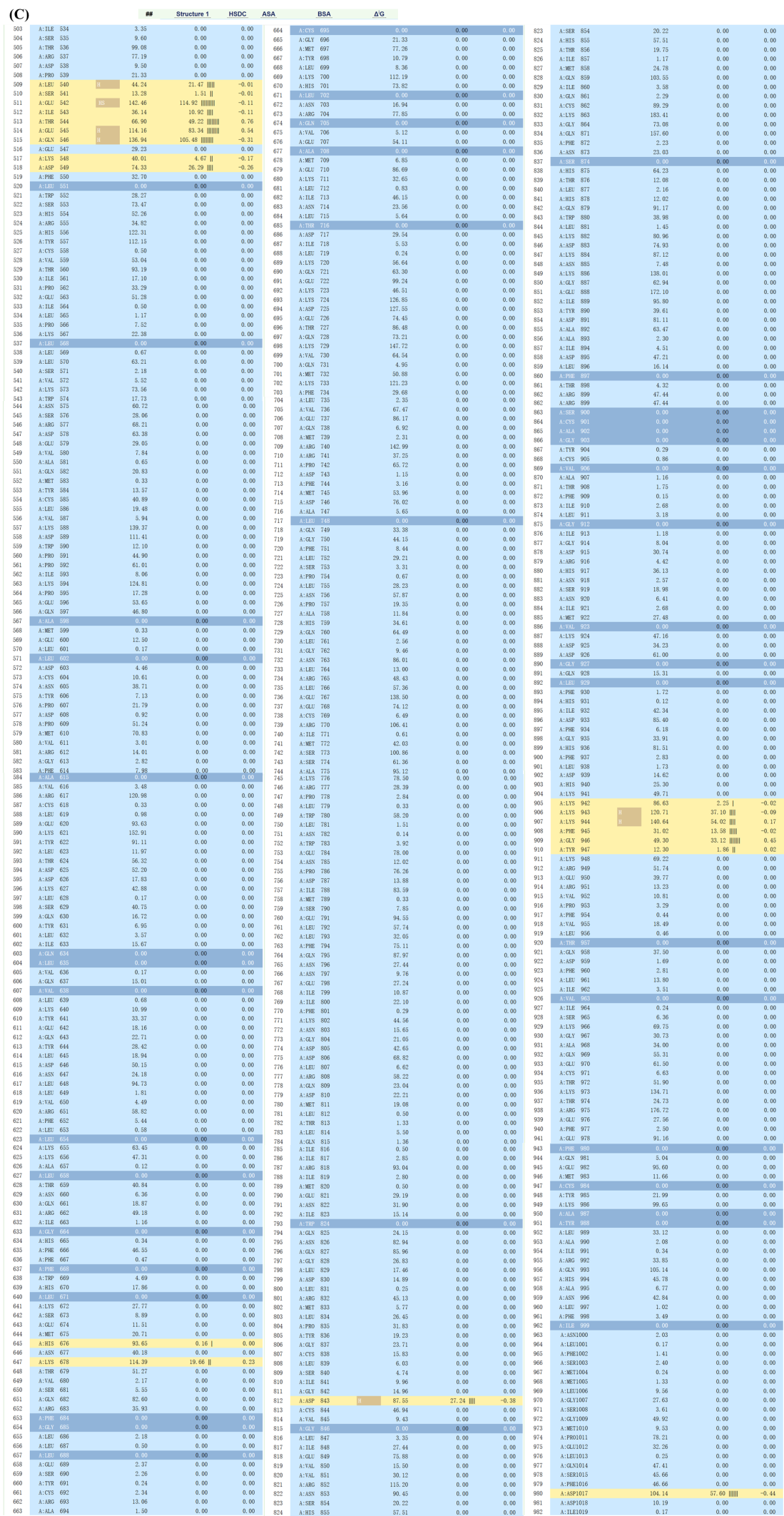
**

**
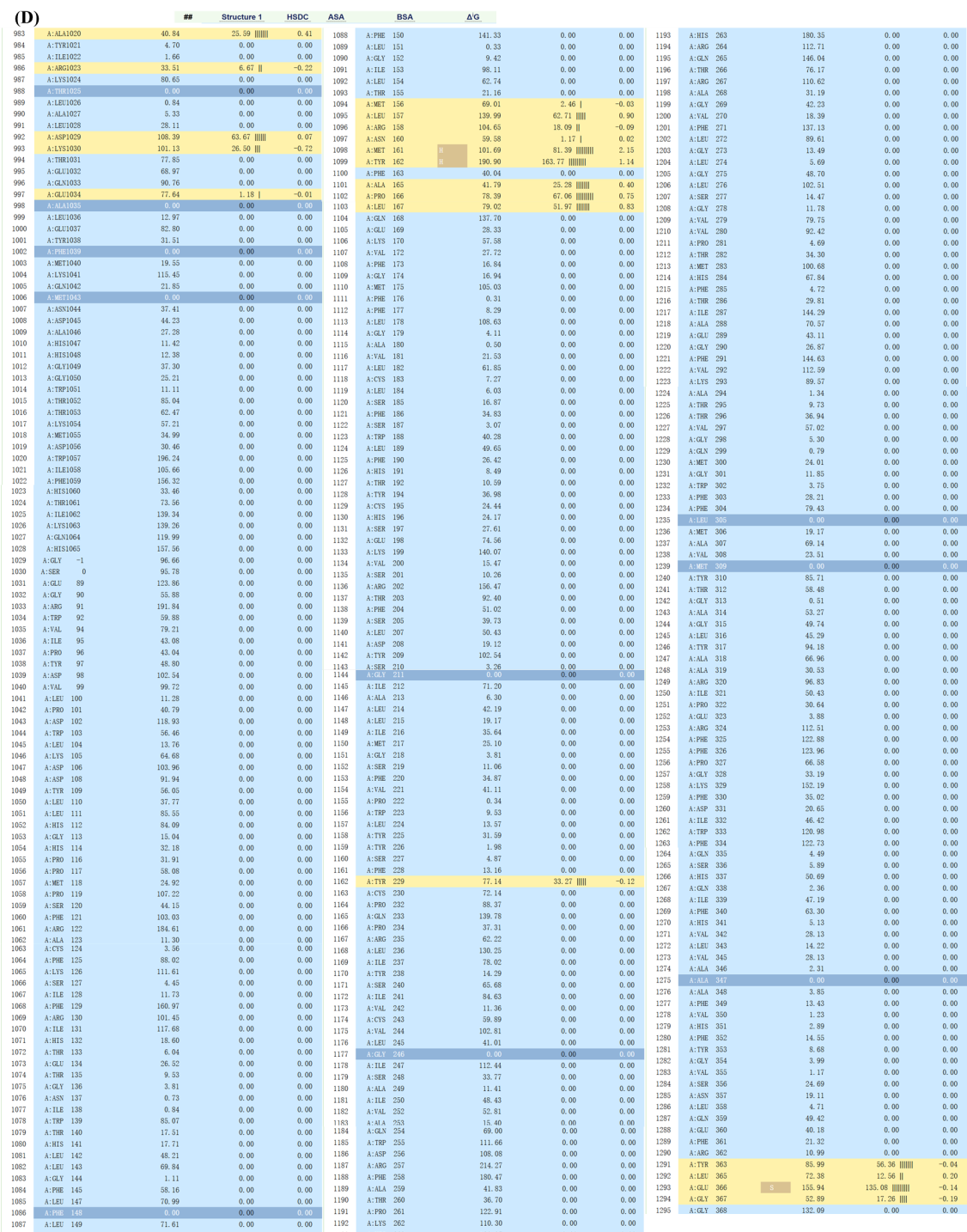
**

**Figure S41.** Molecular docking parameters of AdipoR1 (5LXG) + PI3Kα (7PG5)

(B-D) is continuation of (A). ZDOCK is used for docking and PDBePISA is applied to analyze docking results. Available (November 2024): https://zdock.wenglab.org/; https://www.ebi.ac.uk/msd-srv/prot_int/

**
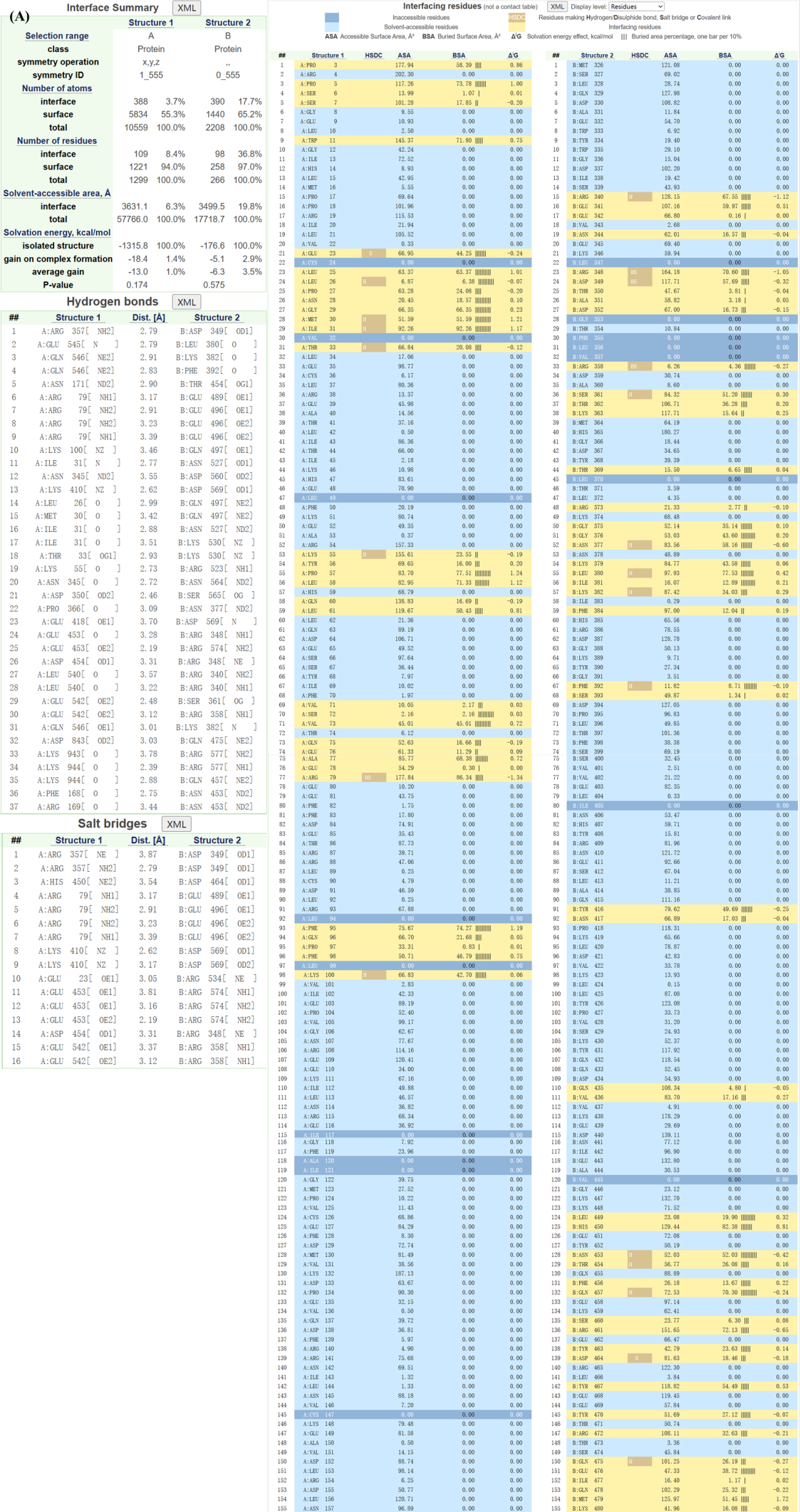
**

**
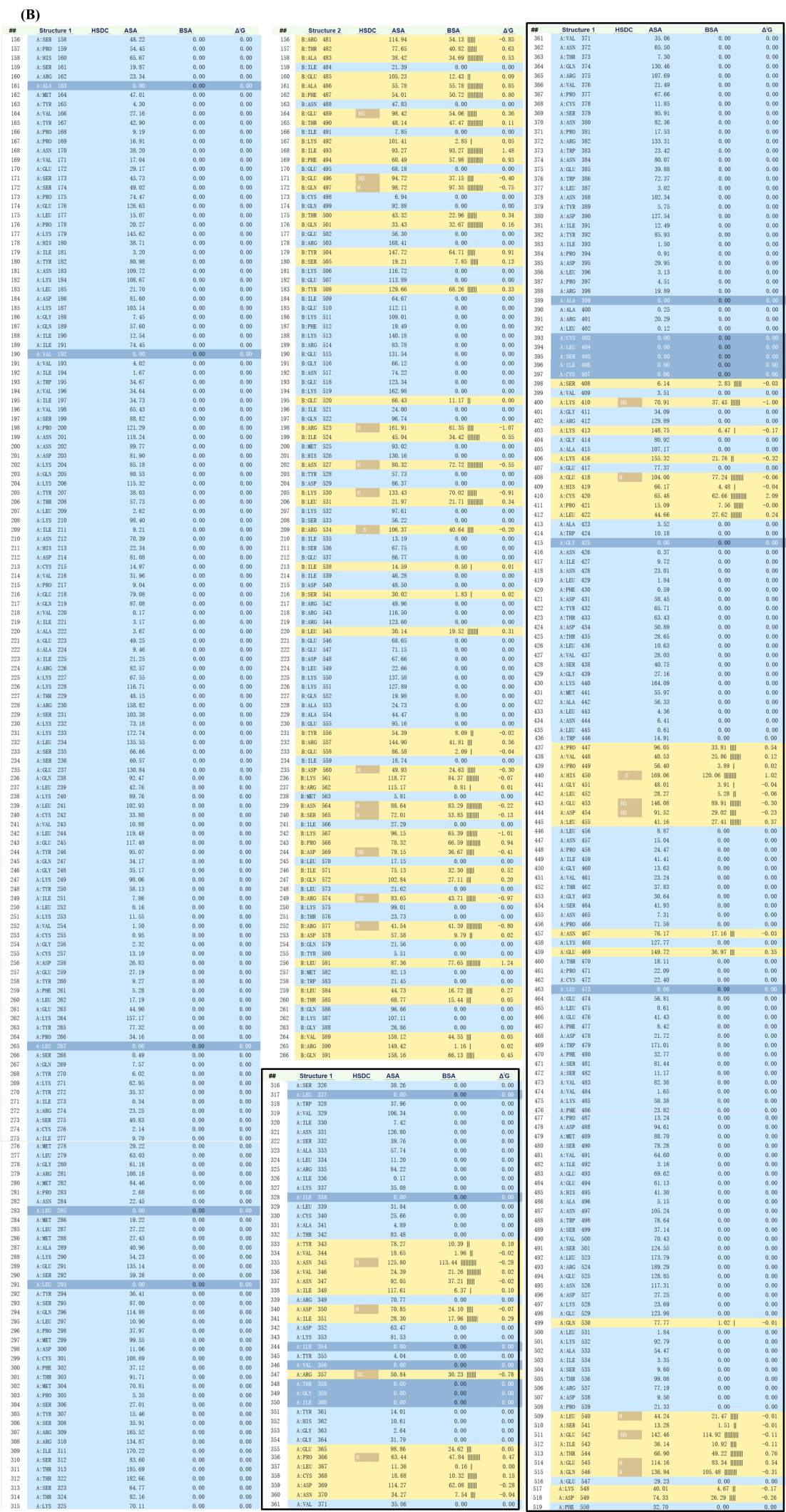
**

**
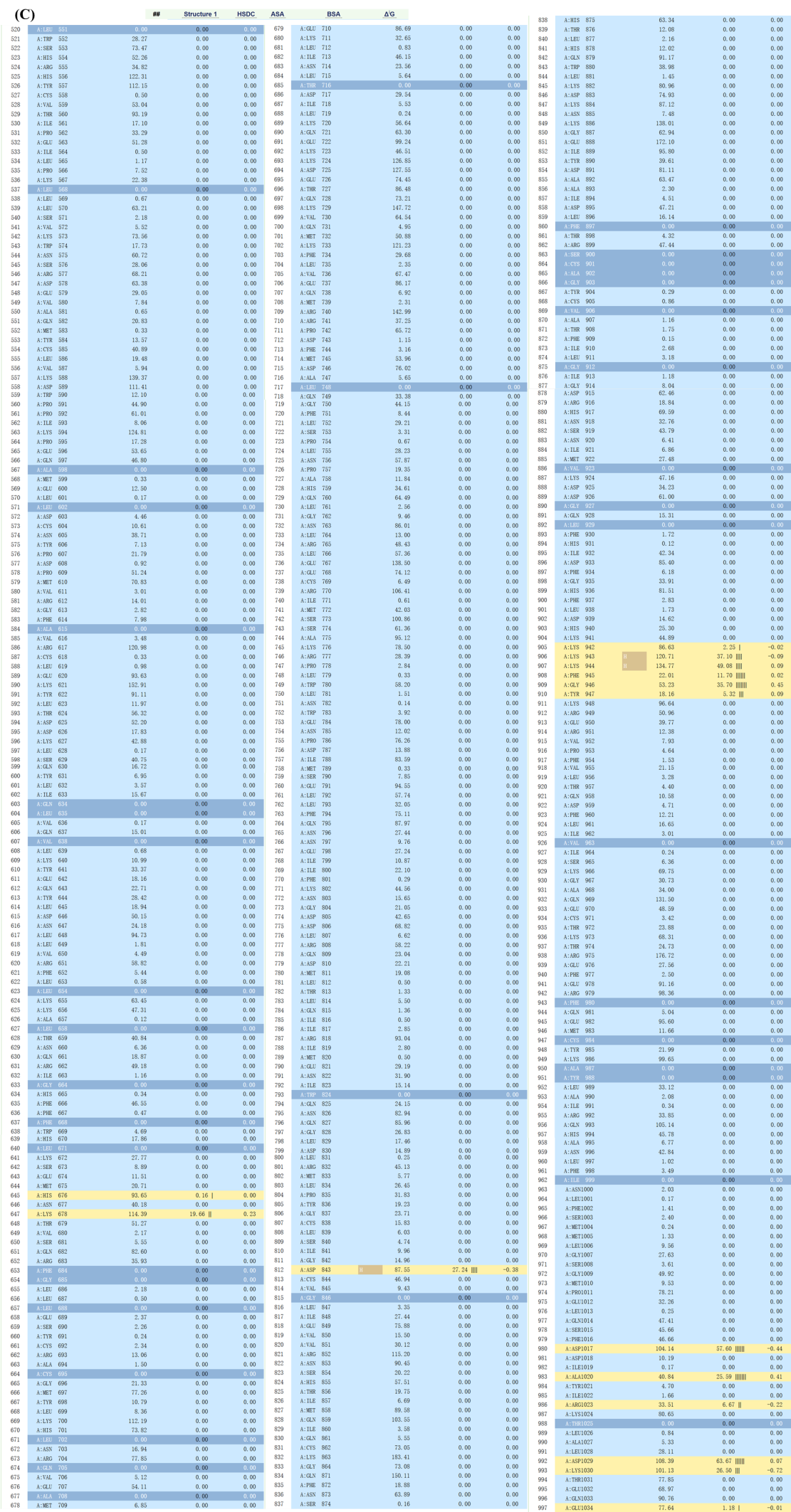
**

**
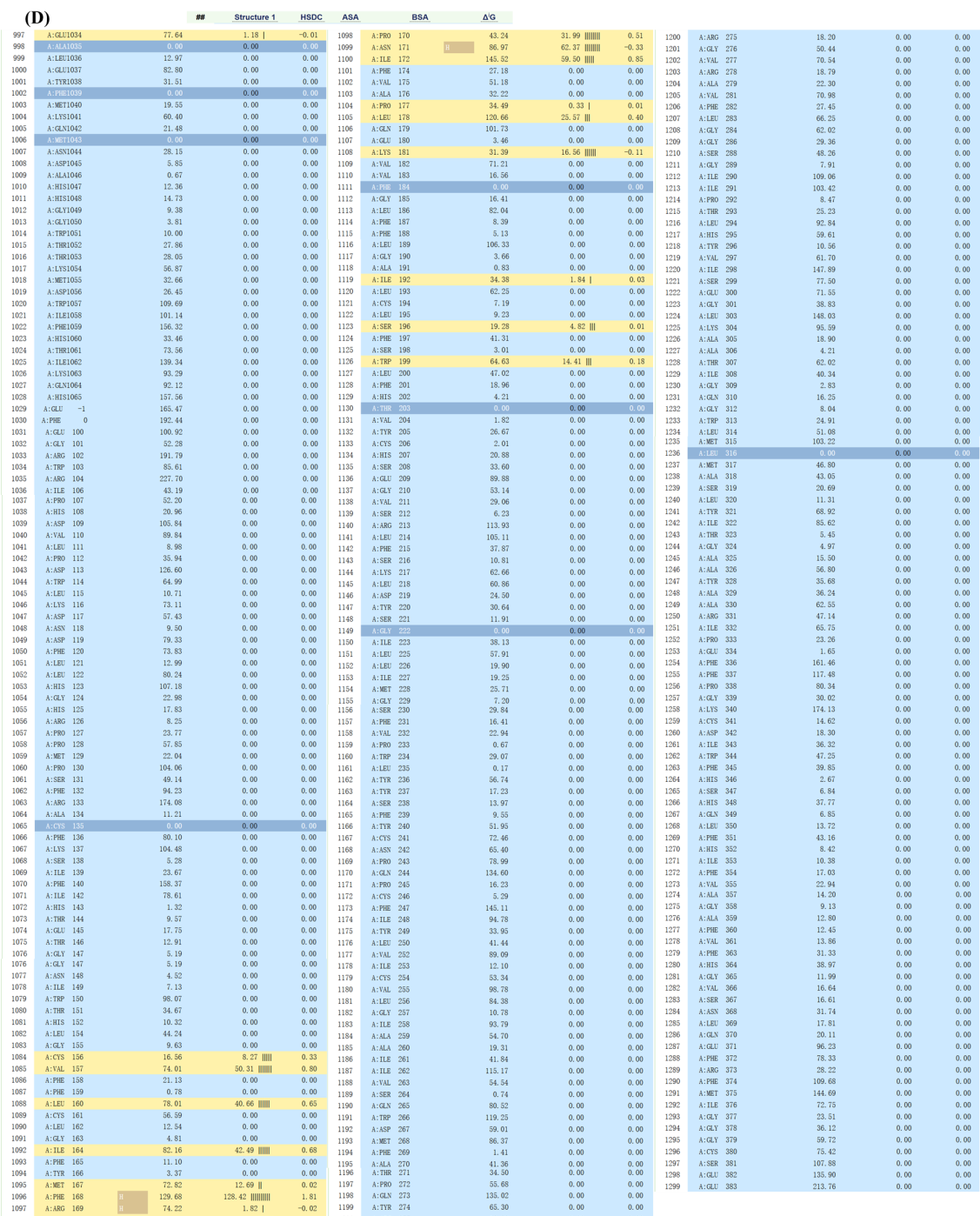
**

**Figure S42.** Molecular docking parameters of AdipoR2 (6KS1) + PI3Kα (7PG5)

(B-D) is continuation of (A). ZDOCK is used for docking and PDBePISA is applied to analyze docking results. Available (November 2024): https://zdock.wenglab.org/; https://www.ebi.ac.uk/msd-srv/prot_int/

**
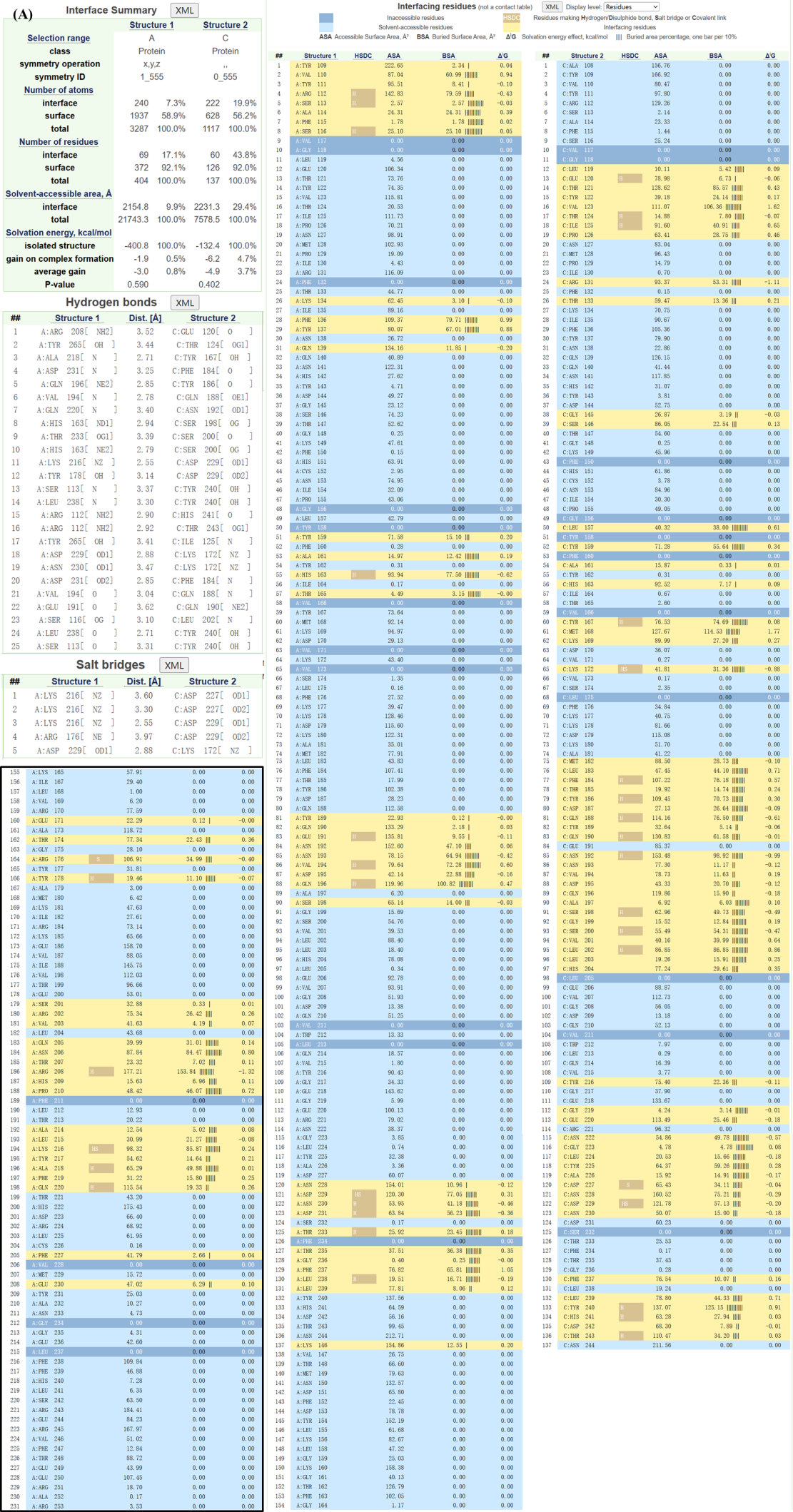
**

**
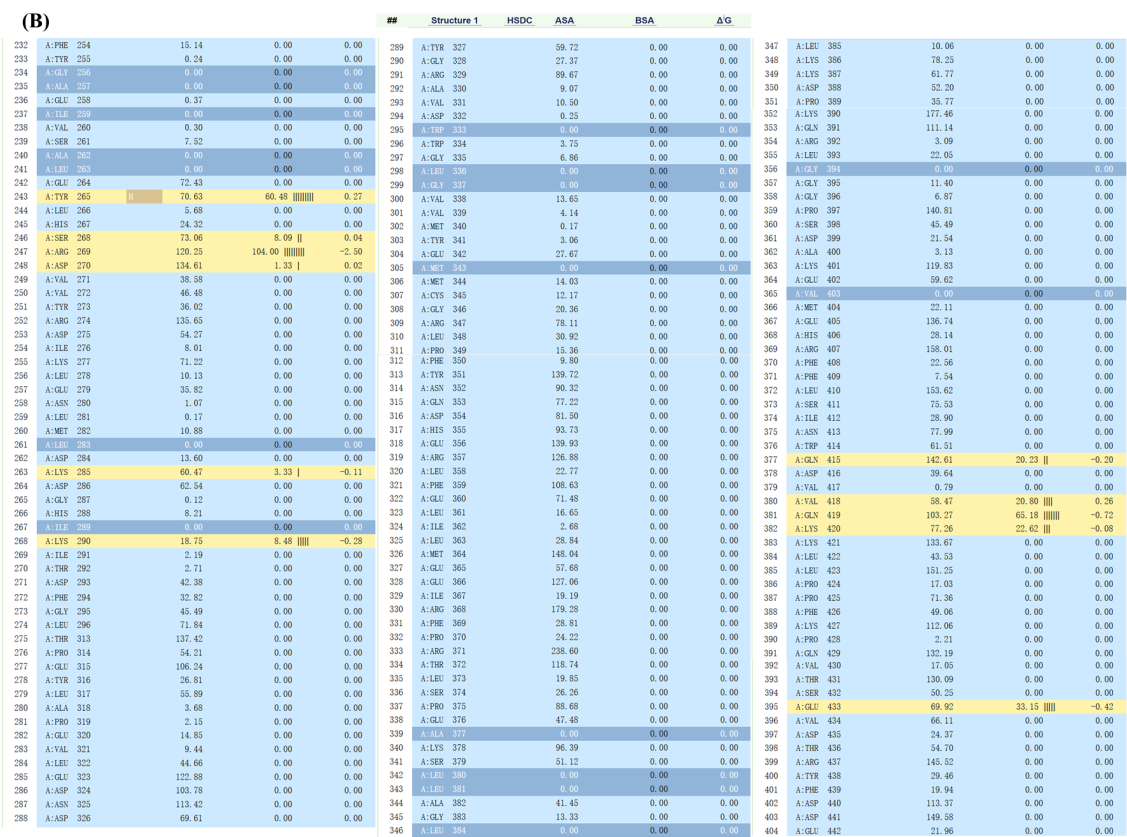
**

**Figure S43.** Molecular docking parameters of APN (6U66) + AKT (1GZN)

(B) is continuation of (A). ZDOCK is used for docking and PDBePISA is applied to analyze docking results. Available (November 2024): https://zdock.wenglab.org/; https://www.ebi.ac.uk/msd-srv/prot_int/

**
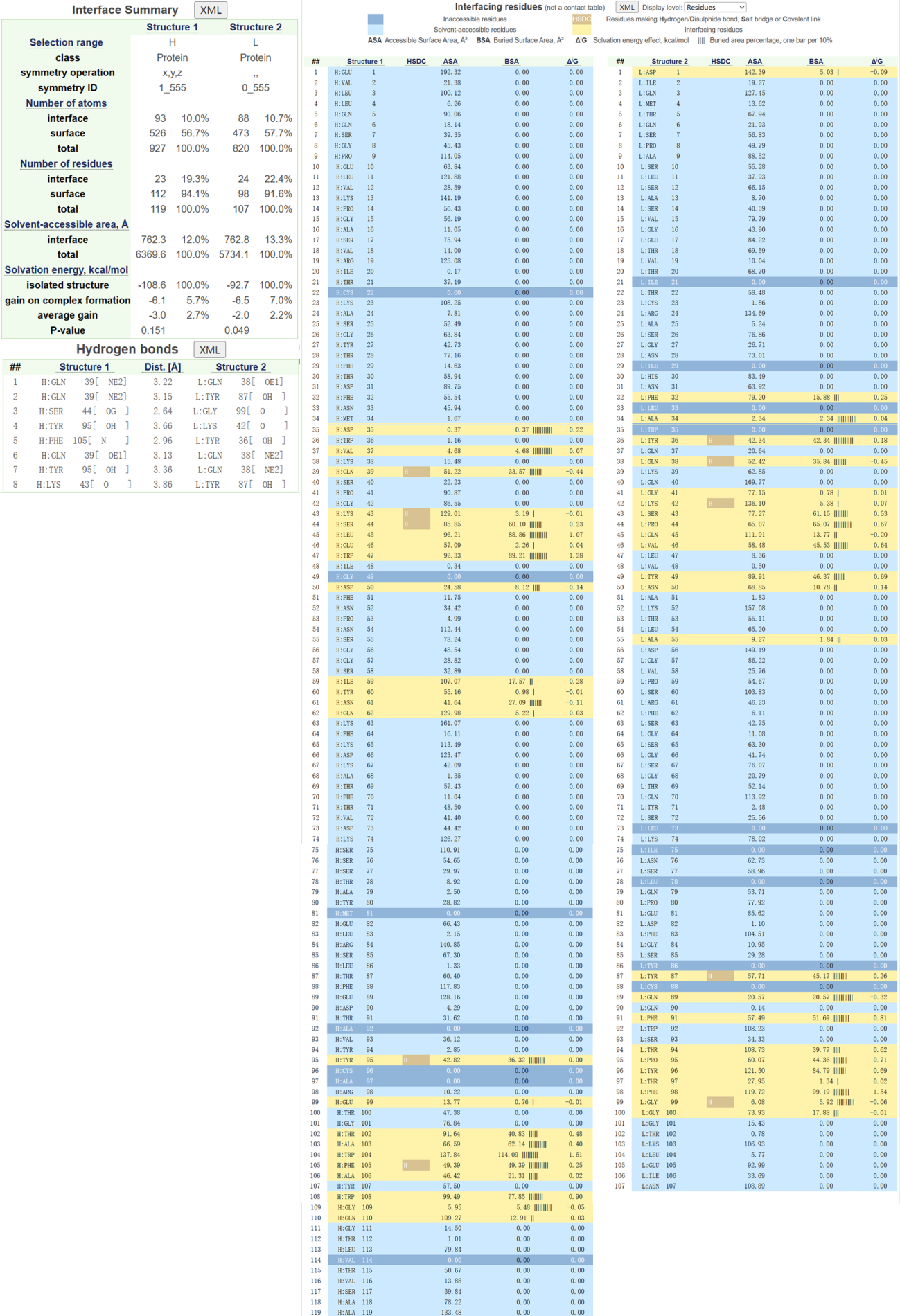
**

**Figure S44.** Molecular docking parameters of AdipoR1 (5LXG) + AKT (1GZN)

(B) is continuation of (A). ZDOCK is used for docking and PDBePISA is applied to analyze docking results. Available (November 2024): https://zdock.wenglab.org/; https://www.ebi.ac.uk/msd-srv/prot_int/

**
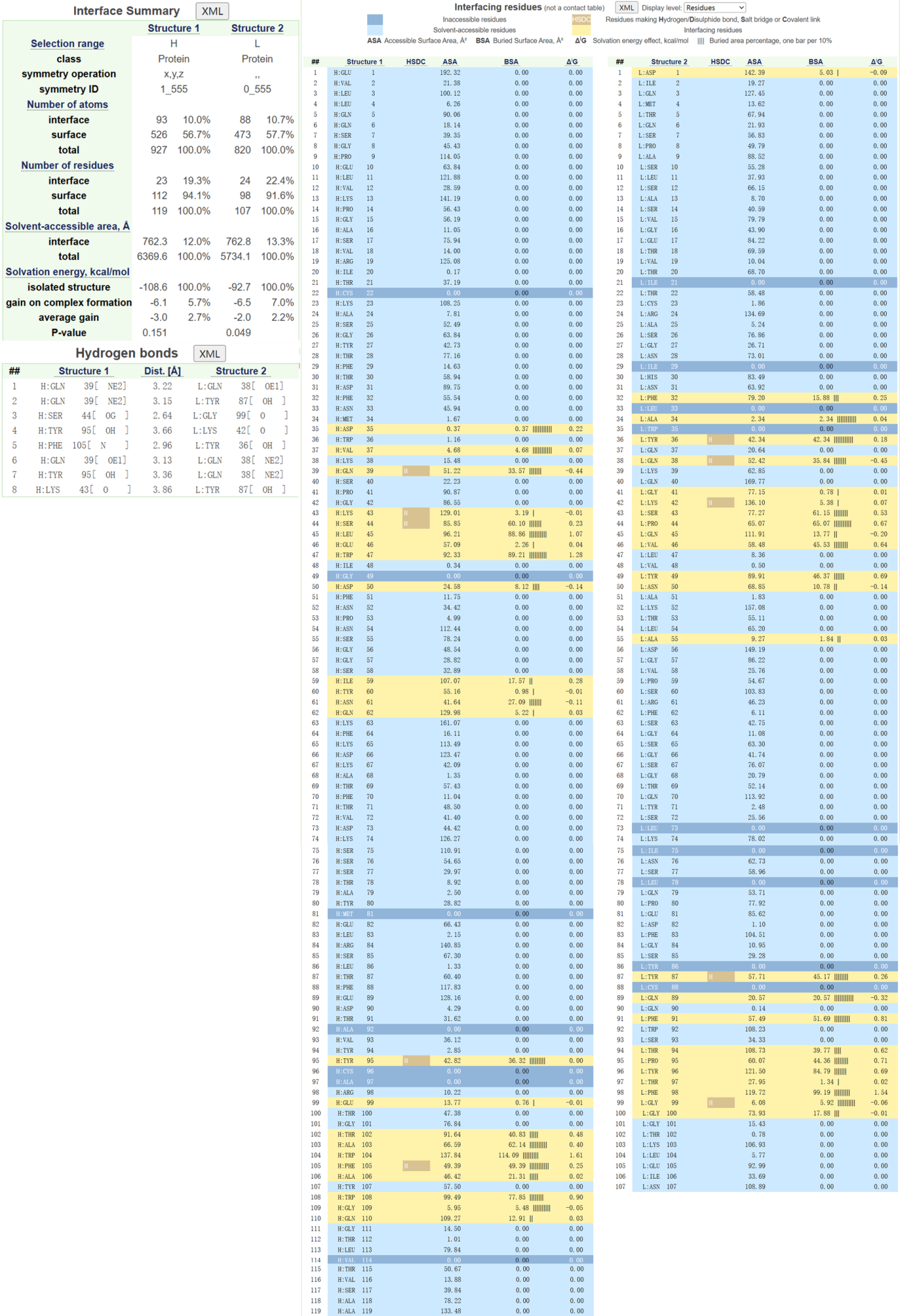
**

**Figure S45.** Molecular docking parameters of AdipoR2 (6KS1) + AKT (1GZN)

(B) is continuation of (A). ZDOCK is used for docking and PDBePISA is applied to analyze docking results. Available (November 2024): https://zdock.wenglab.org/; https://www.ebi.ac.uk/msd-srv/prot_int/

**
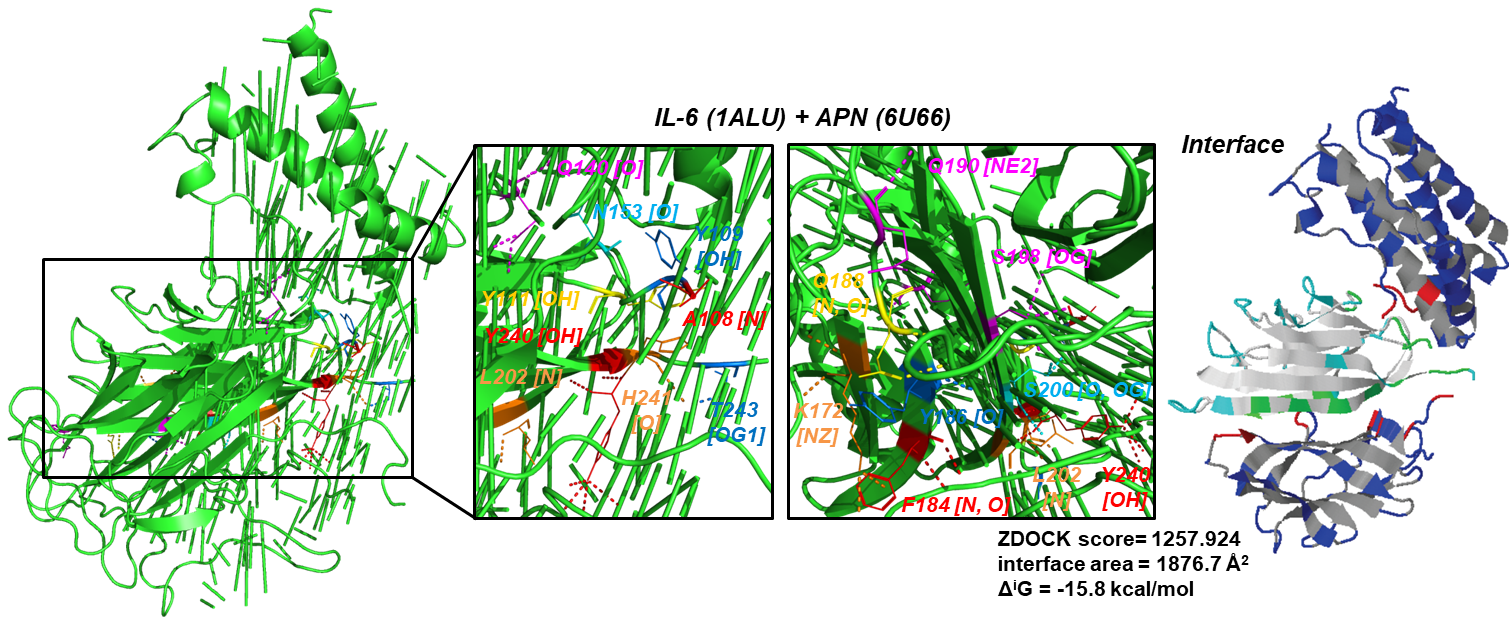
**

**Figure S46.** Amino acid sites of APN docking with IL-6

**
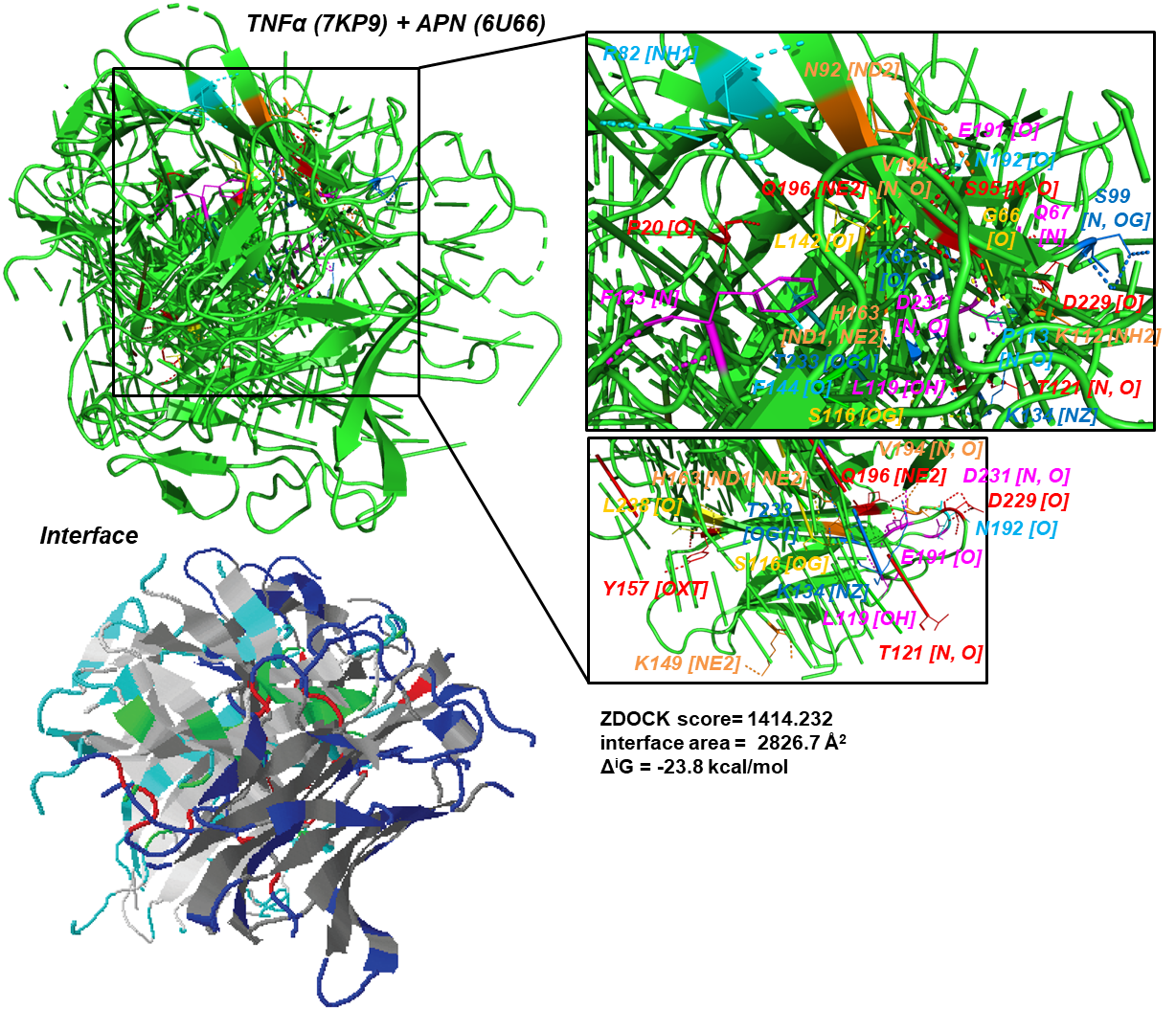
**

**Figure S47.** Amino acid sites of APN docking with TNF-α

**
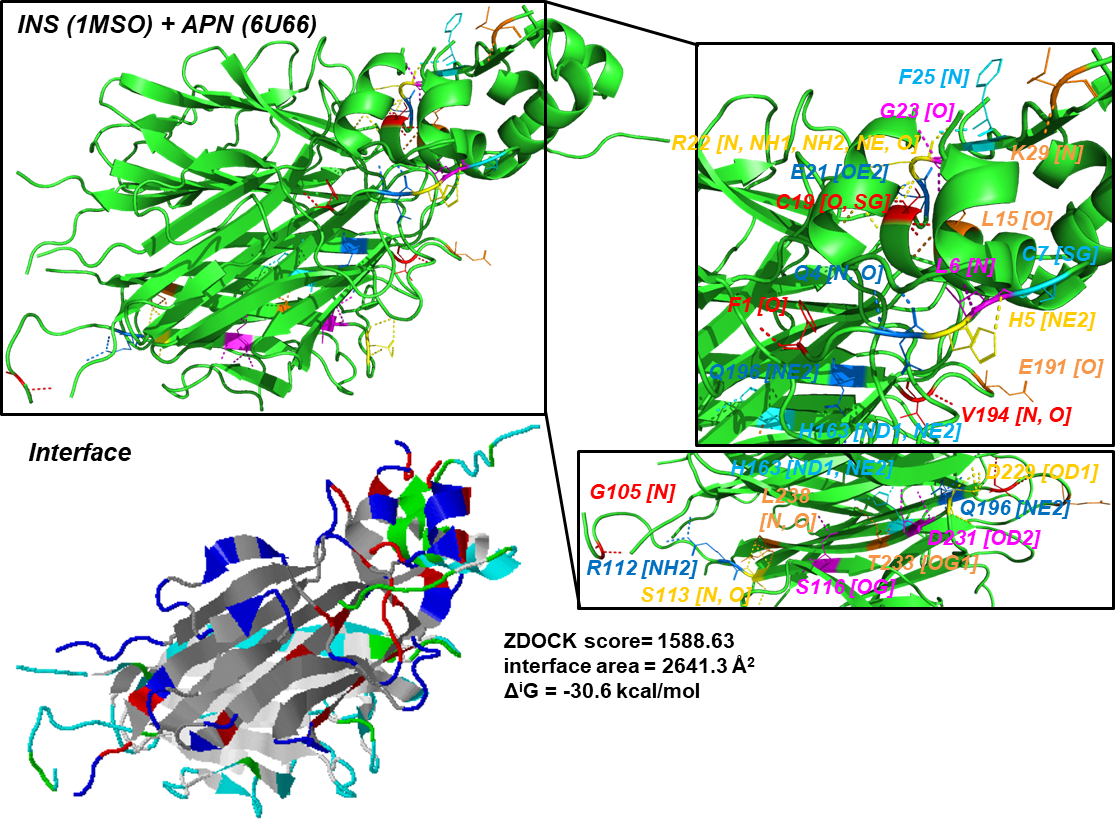
**

**Figure S48.** Amino acid sites of APN docking with INS

**
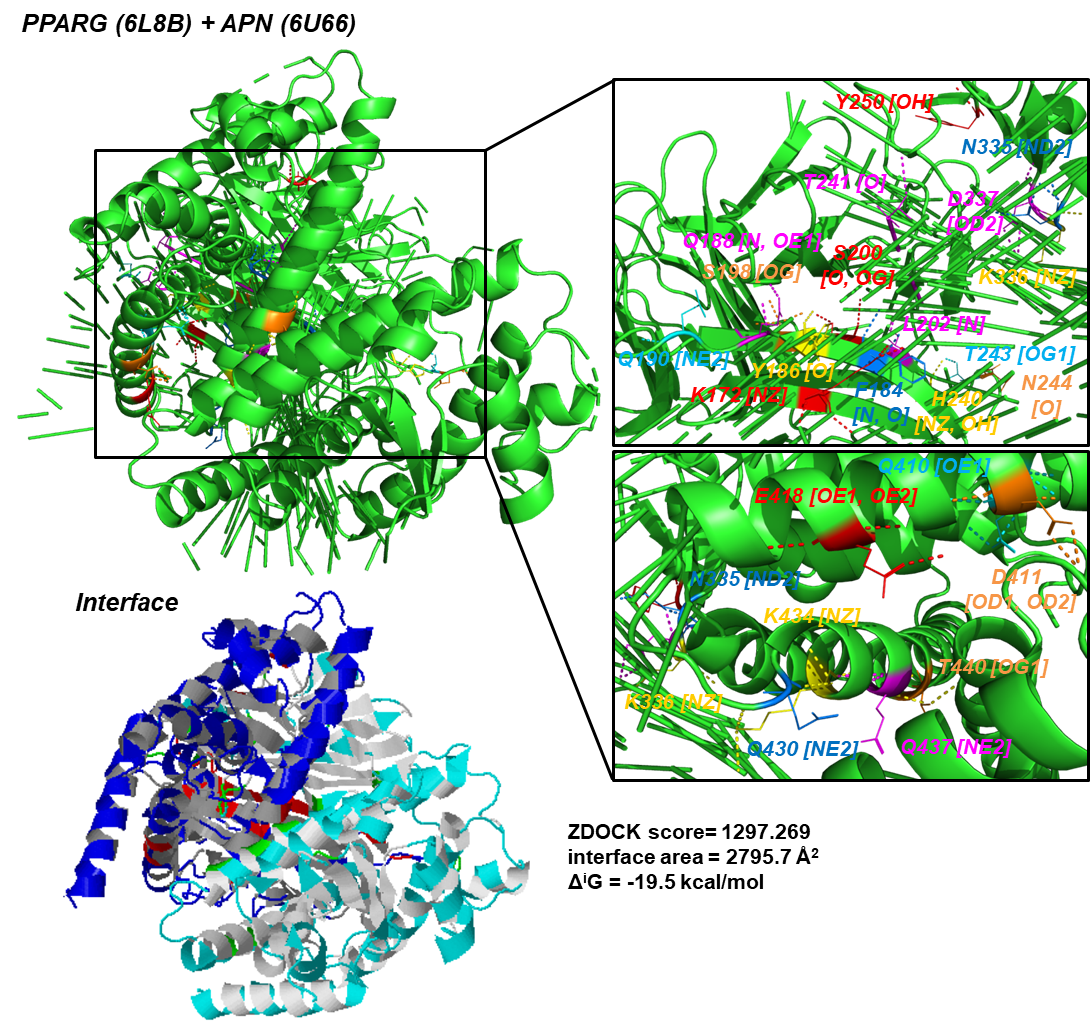
**

**Figure S49.** Amino acid sites of APN docking with PPARG

**
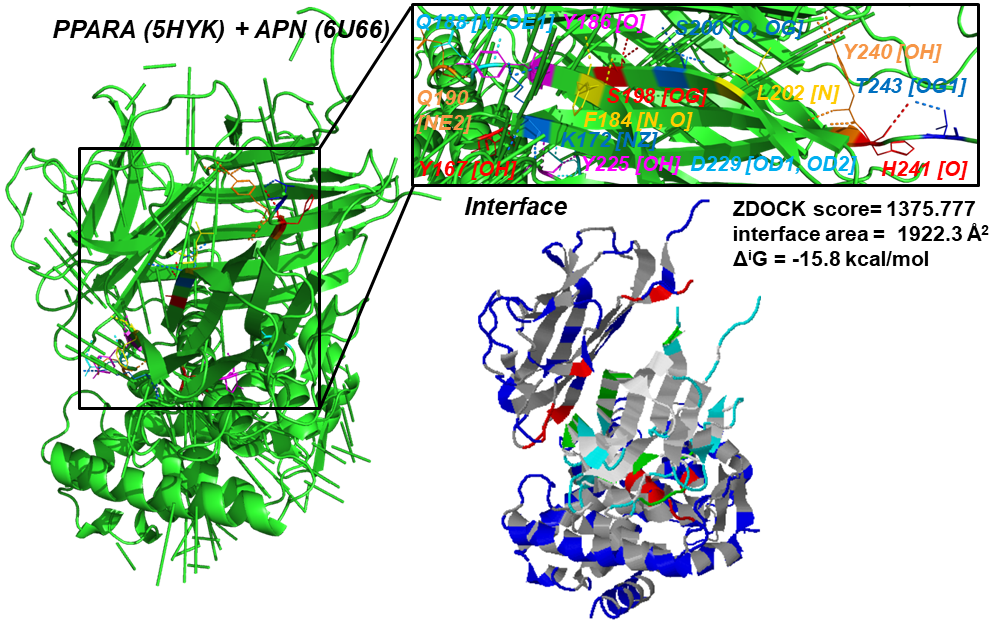
**

**Figure S50.** Amino acid sites of APN docking with PPARA

**
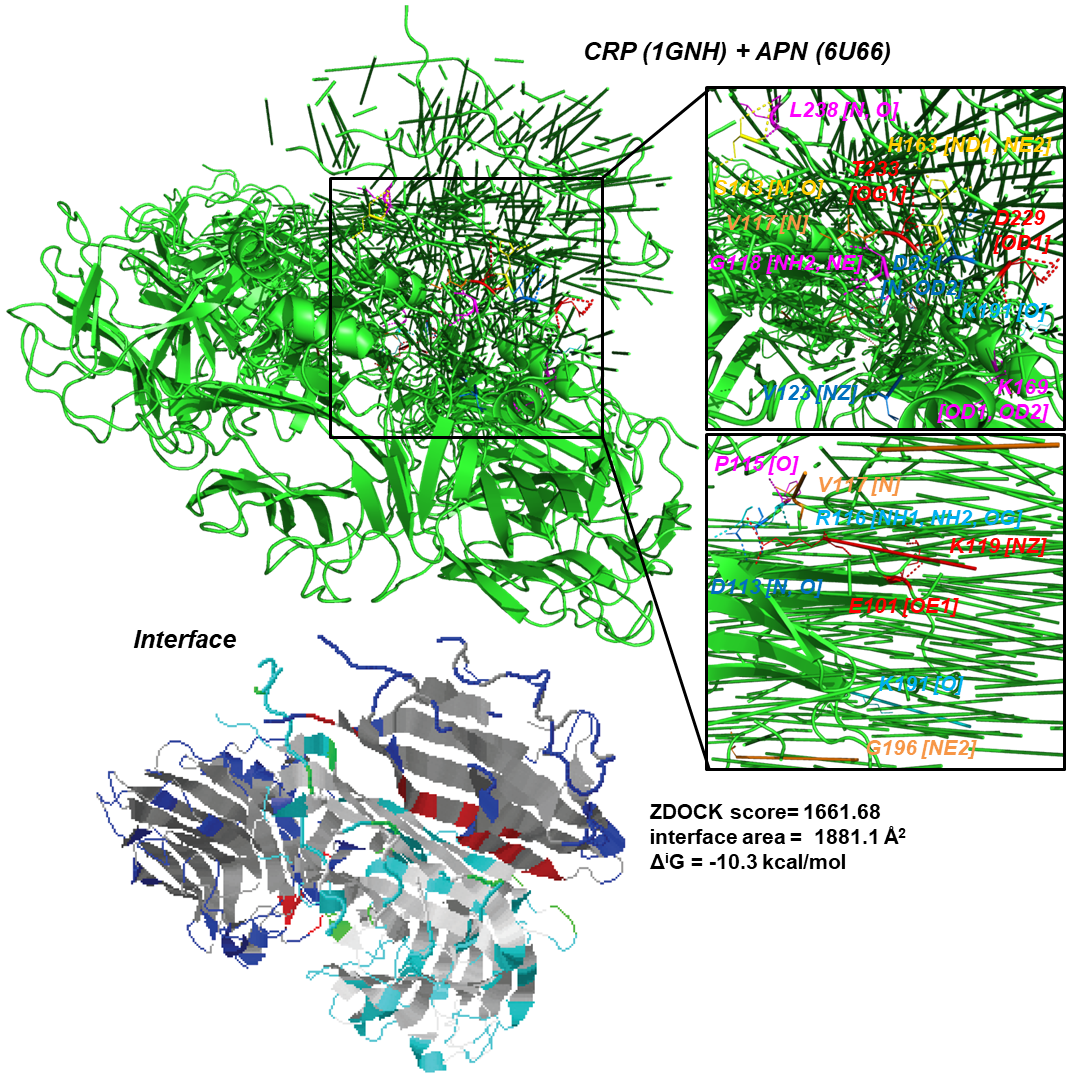
**

**Figure S51.** Amino acid sites of APN docking with CRP

**
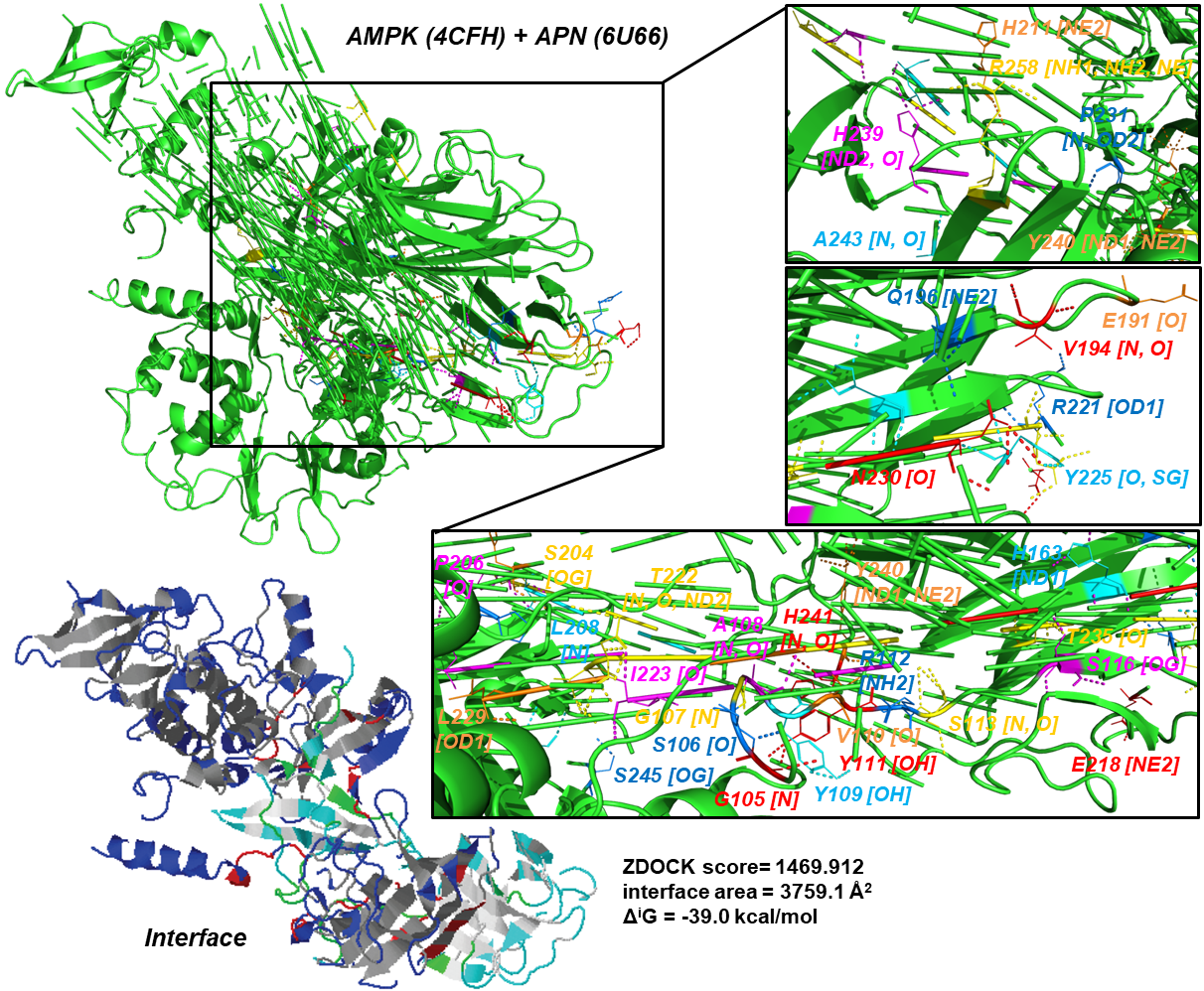
**

**Figure S52.** Amino acid sites of APN docking with AMPK

**
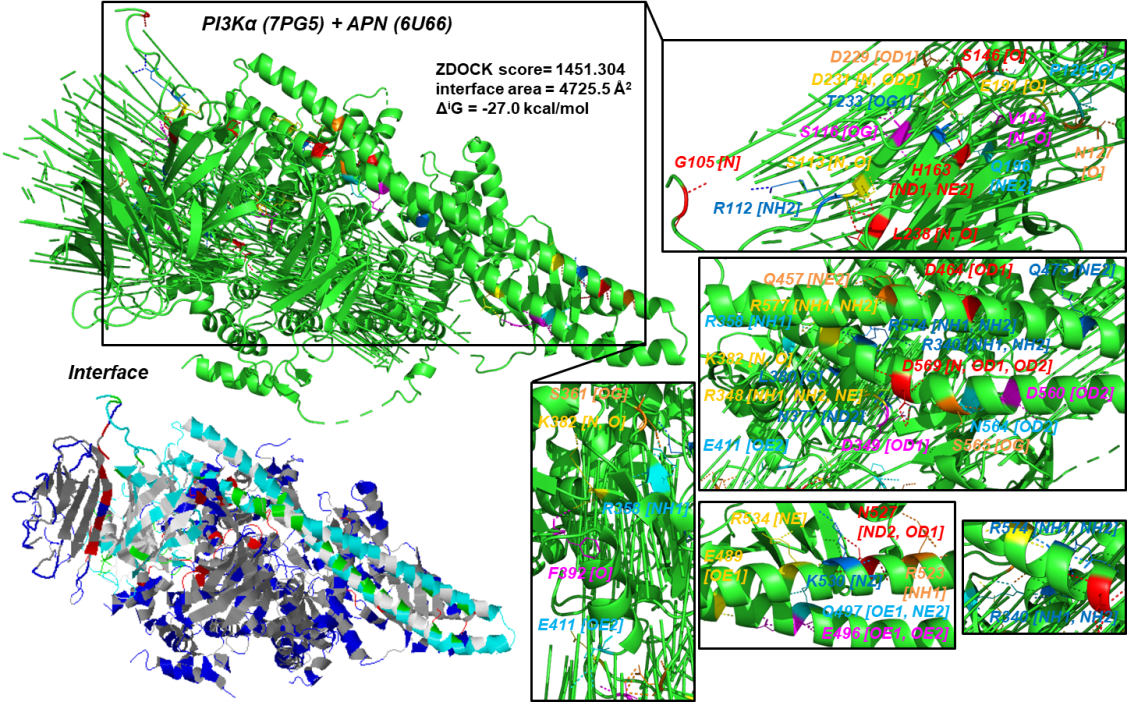
**

**Figure S53.** Amino acid sites of APN docking with PI3Kα

**
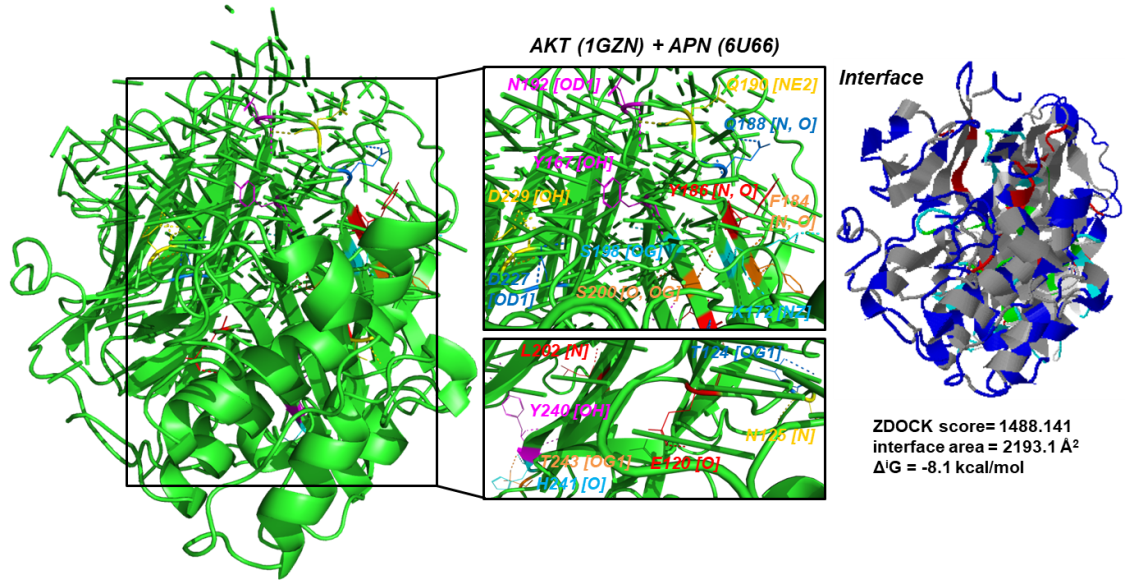
**

**Figure S54.** Amino acid sites of APN docking with AKT

**
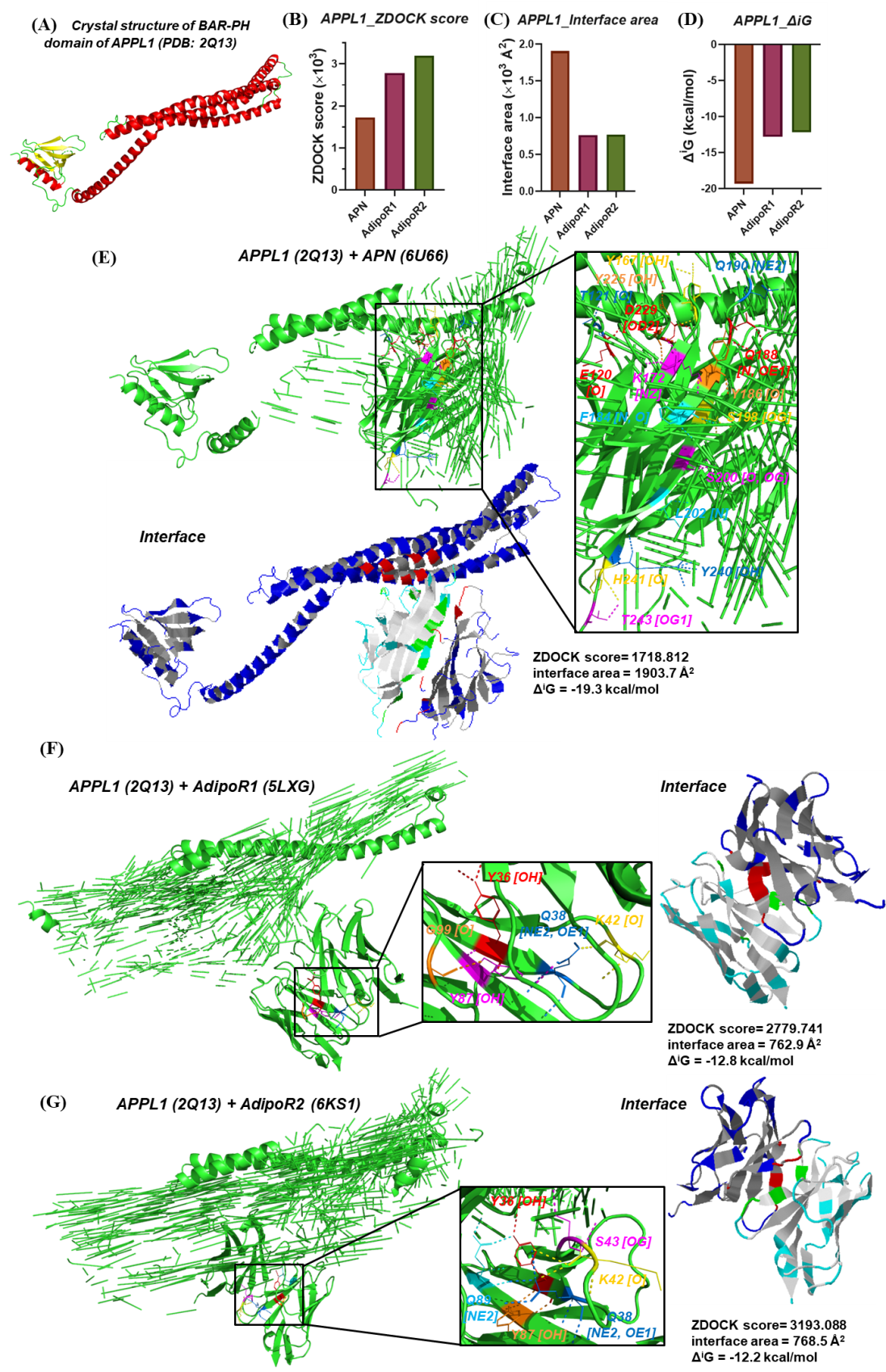
**

**Figure S55.** Molecular docking of APPL1 with APN, AdipoR1 and AdipoR2

(A) spatial structure of APPL1; (B) ZDOCK score; (C) interface area; (D) Δ^i^G; (E-G) APPL1 docking with APN, AdipoR1 and AdipoR2. APPL1: adaptor protein containing pleckstrin homology domain, phosphotyrosine binding domain, and leucine zipper motif 1; APN: adiponectin; AdipoR1: adiponectin receptor 1; AdipoR2: adiponectin receptor 2; Δ^i^G: Gibbs free energy. Available (November 2024): https://zdock.wenglab.org/; https://www.ebi.ac.uk/msd-srv/prot_int/

**
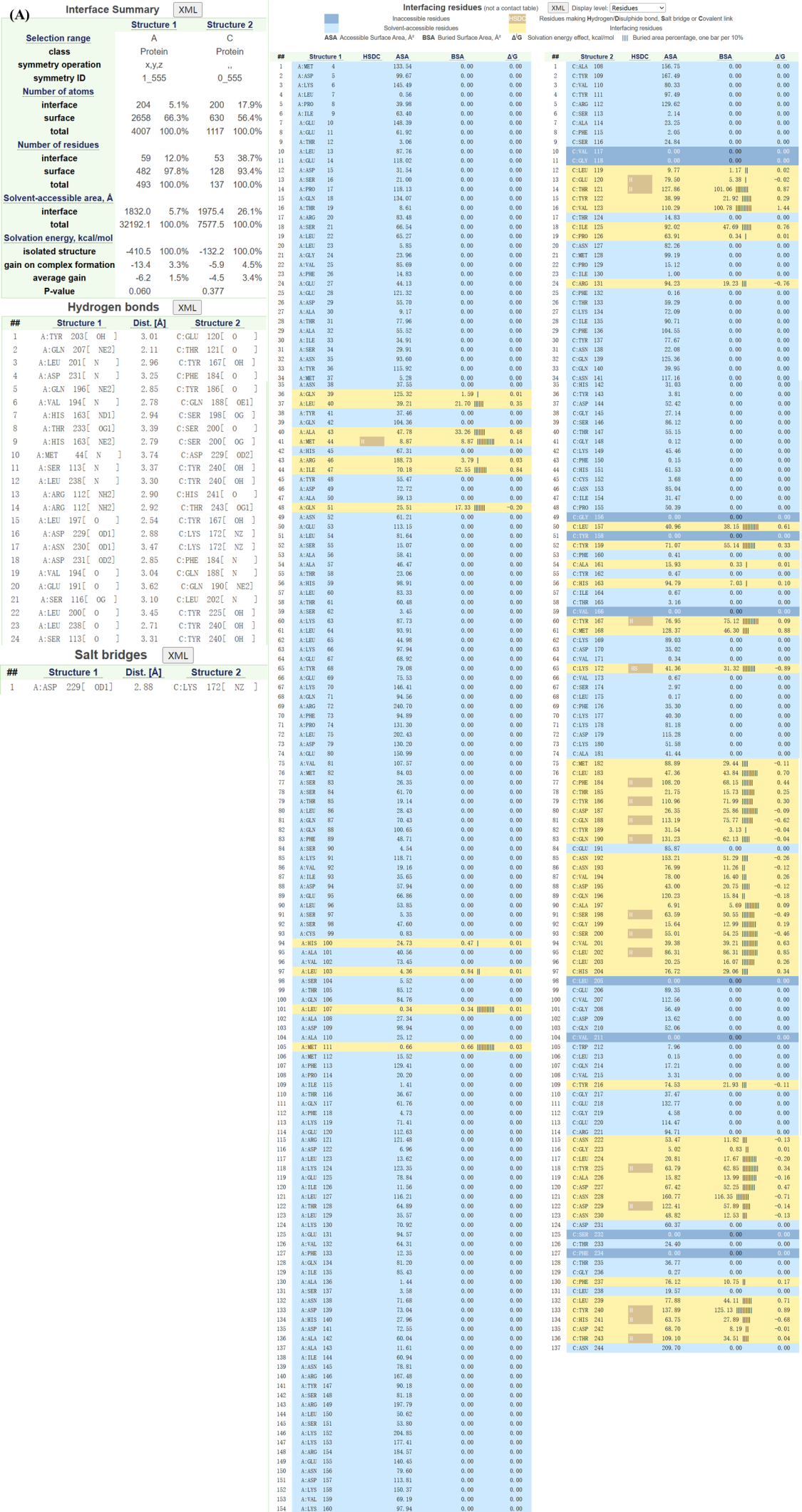
**

**
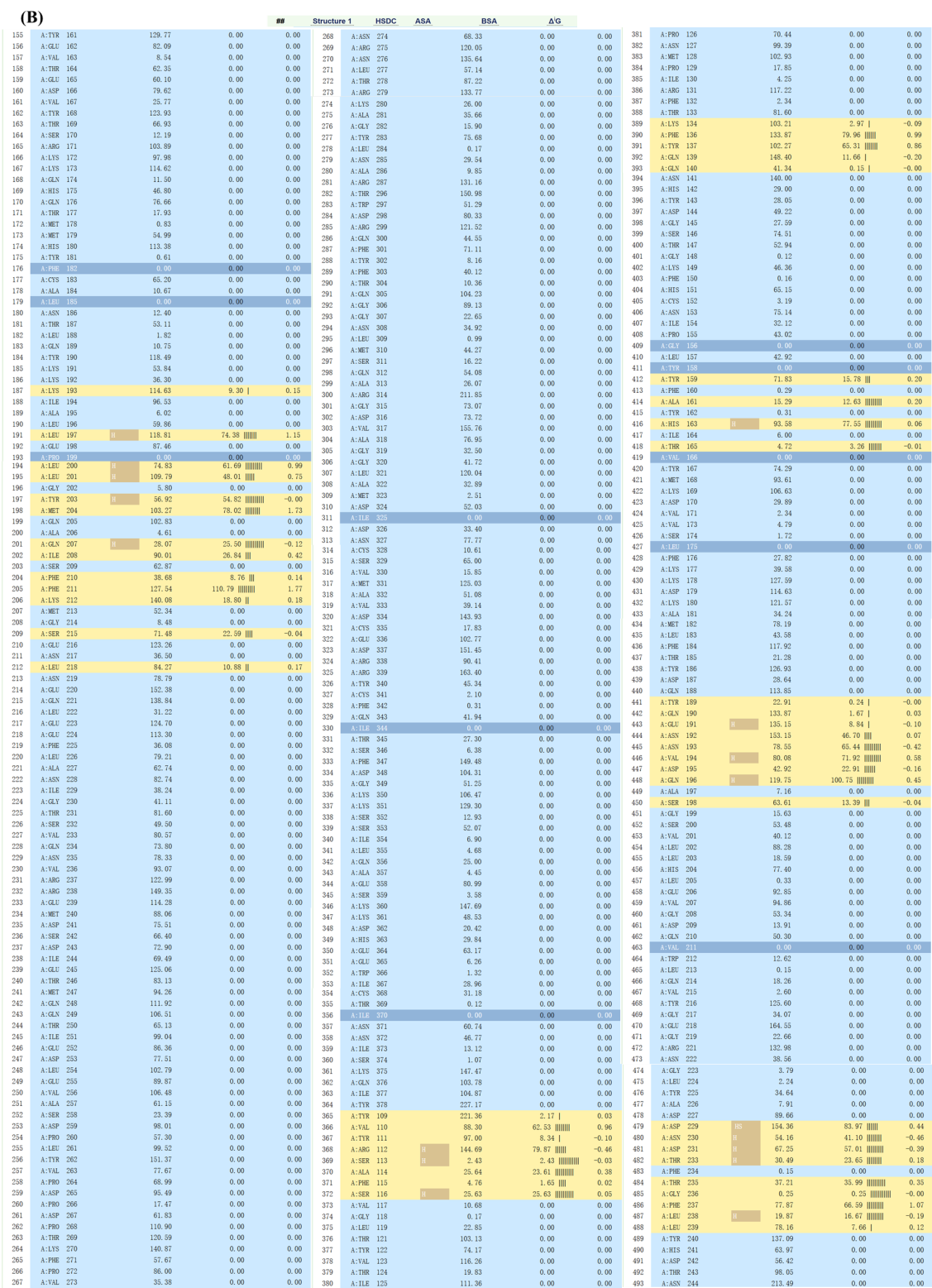
**

**Figure S56.** Molecular docking parameters of APN (6U66) + APPL1 (2Q13)

(B) is continuation of (A). ZDOCK is used for docking and PDBePISA is applied to analyze docking results. Available (November 2024): https://zdock.wenglab.org/; https://www.ebi.ac.uk/msd-srv/prot_int/

**
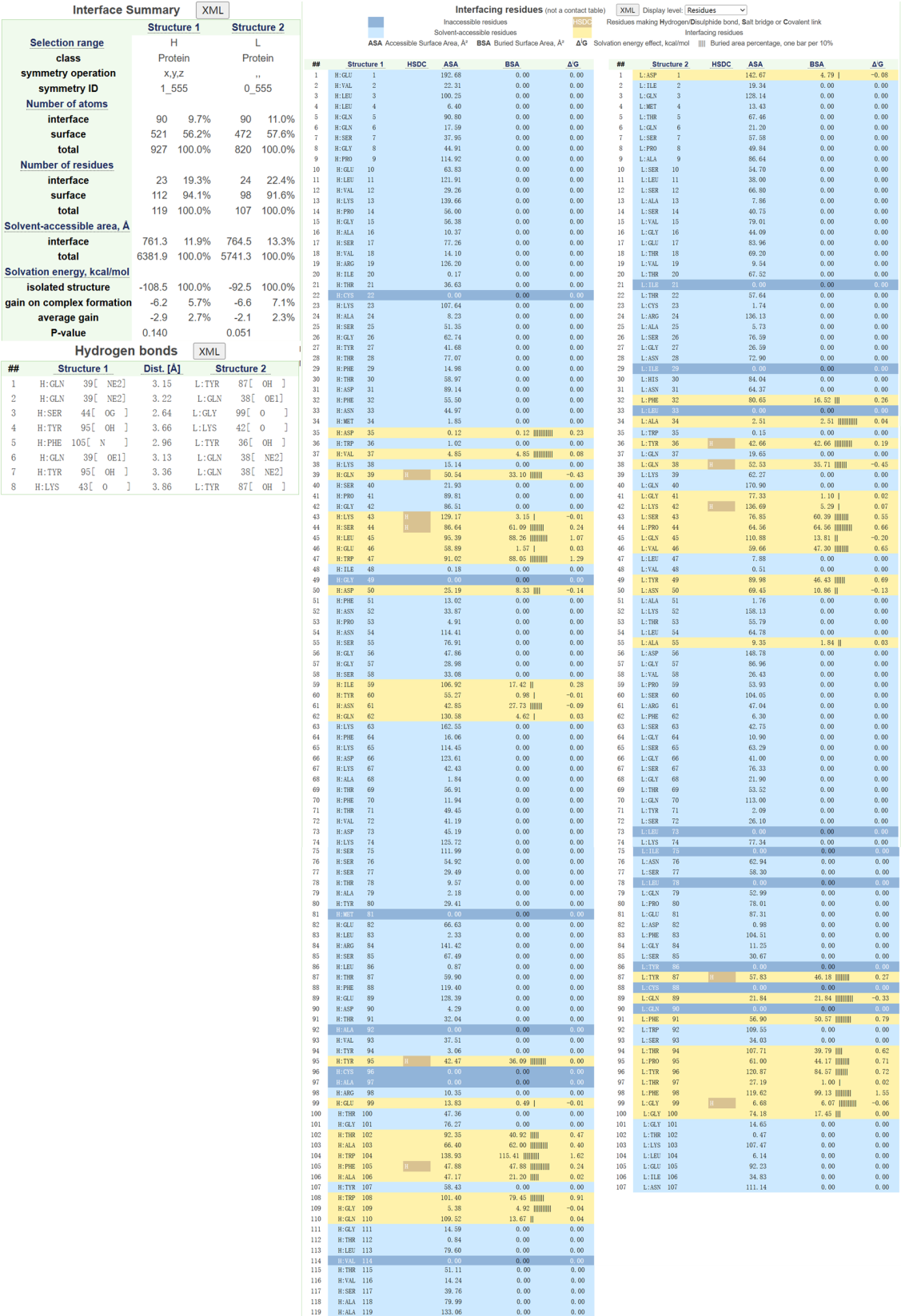
**

**Figure S57.** Molecular docking parameters of AdipoR1 (5LXG) + APPL1 (2Q13)

ZDOCK is used for docking and PDBePISA is applied to analyze docking results. Available (November 2024): https://zdock.wenglab.org/; https://www.ebi.ac.uk/msd-srv/prot_int/

**
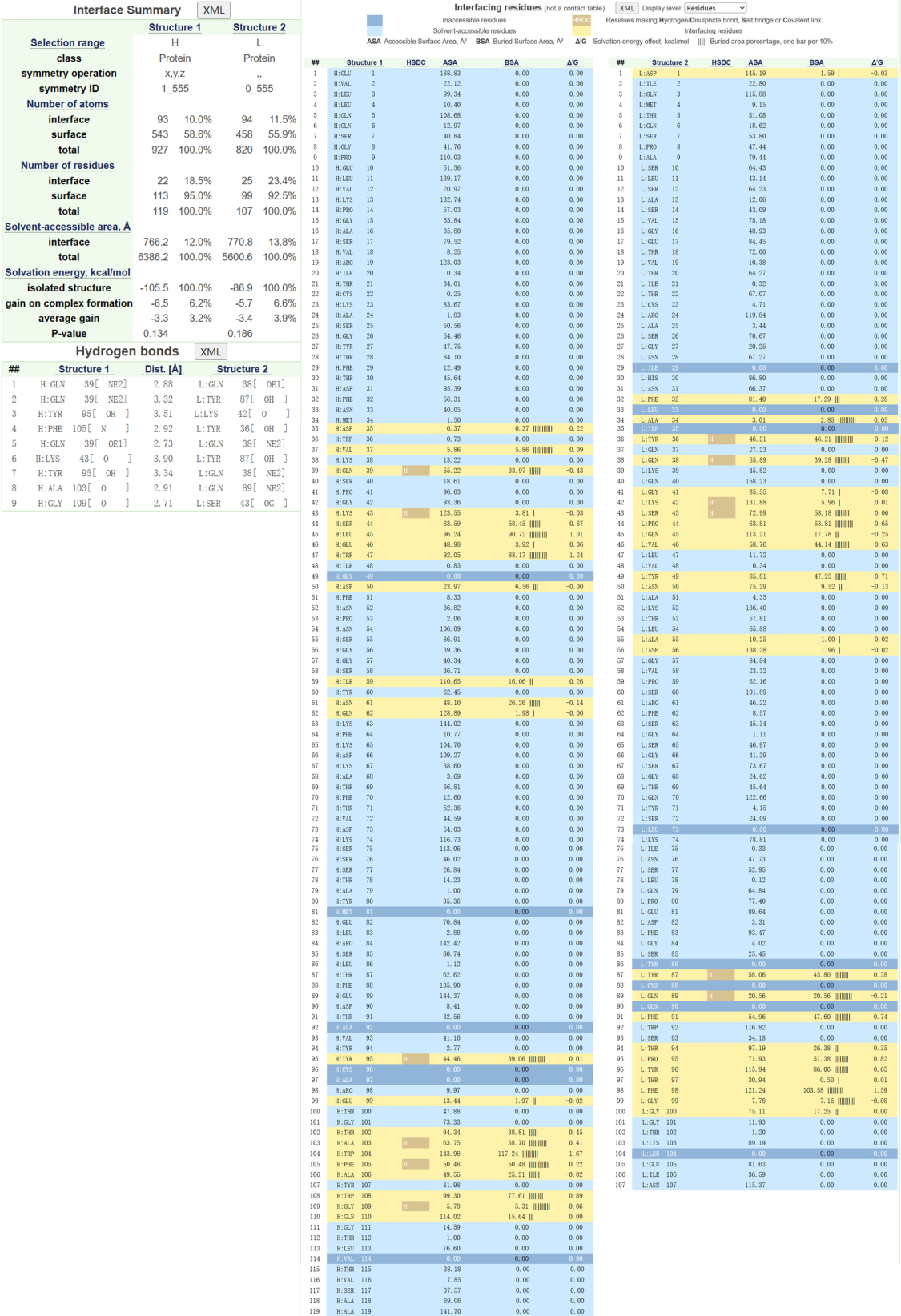
**

**Figure S58.** Molecular docking parameters of AdipoR2 (6KS1) + APPL1 (2Q13)

(B) is continuation of (A). ZDOCK is used for docking and PDBePISA is applied to analyze docking results. Available (November 2024): https://zdock.wenglab.org/; https://www.ebi.ac.uk/msd-srv/prot_int/

**
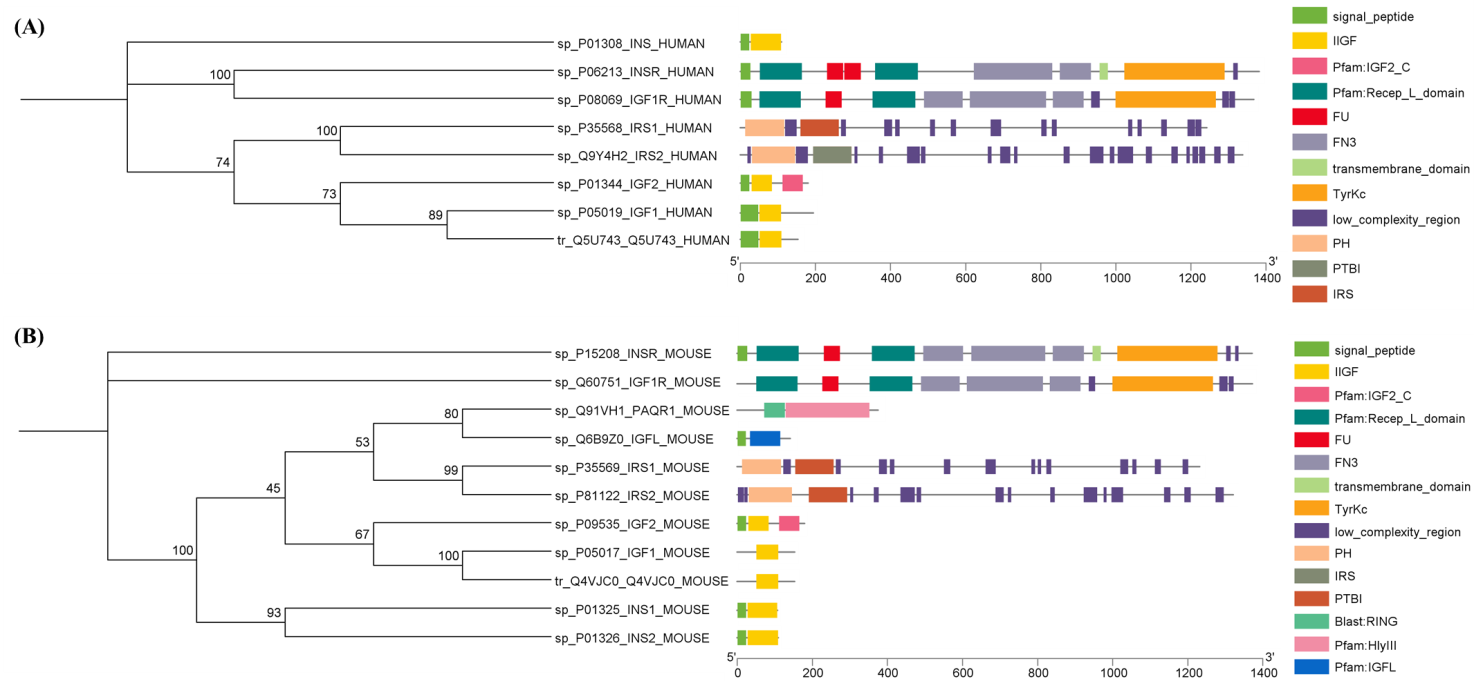
**

**Figure S59.** Phylogenetic tree and functional domains of IGF-1 and INS

(A) human; (B) mouse. INS: insulin; INSR: insulin receptor; IGF1R: insulin-like growth factor 1 receptor; IRS: insulin receptor substrate; IGF: insulin-like growth factor; Q5U743: IGF1; PAQR1: adiponectin receptor protein 1; IGFL: insulin growth factor-like family member; Q4VJC0: insulin-like growth factor 1. All human and mouse proteins domains are derived from UniProtKB database [Available (June 2025): https://www.uniprot.org/uniprotkb/]. The TBtools-II software was used for data visualization^1^.

**References**

1 Chen, C. *et al.* TBtools-II: A “one for all, all for one” bioinformatics platform for biological big-data mining. *Molecular Plant* **16**, 1733-1742, doi:10.1016/j.molp.2023.09.010 (2023).
